# Supplementary material for: Snf1/AMPK fine-tunes TORC1 signaling in response to glucose starvation
Source: eLife. 2023 Feb 7;12:e84319. doi: 10.7554/eLife.84319 (PMC9937656; doi:10.7554/eLife.84319)

# Figure 1A

Loading order:

| | WT | | | *snf1Δ* | | | *snf1^as* | | |
|---|---|---|---|---|---|---|---|---|---|
| | Exp | -C | | Exp | -C | | Exp | -C | |
| 2NM-PP1 | | - | + | | - | + | | - | + |

## Anti-Sch9-pThr737

Replica 1

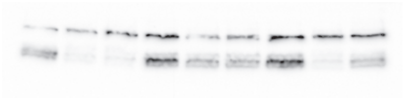

Replica 2

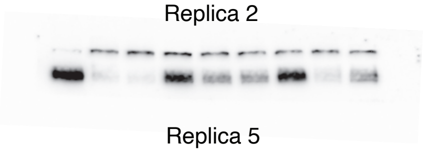

Replica 3

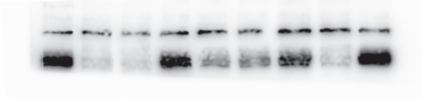

Replica 4

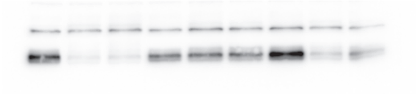

Replica 5

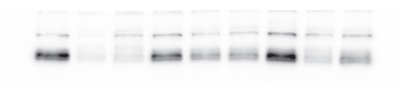

Replica 6 (Data shown in Figure 1A)

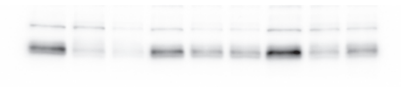

## Anti-Sch9

Replica 1

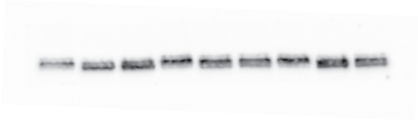

Replica 2
Replica 3

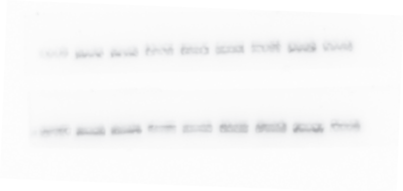

Replica 4

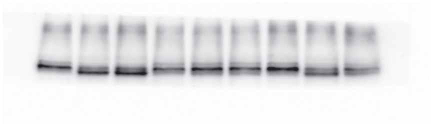

Replica 5

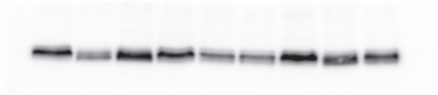

Replica 6 (Data shown in Figure 1A)

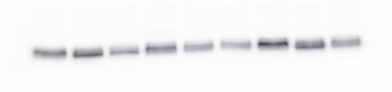

## Anti-Snf1-pThr210

Replica 1

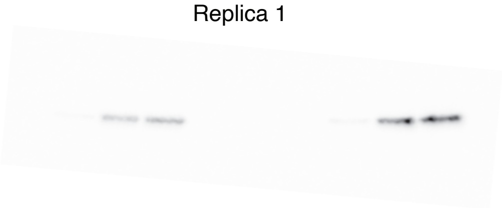

Replica 2
Replica 3

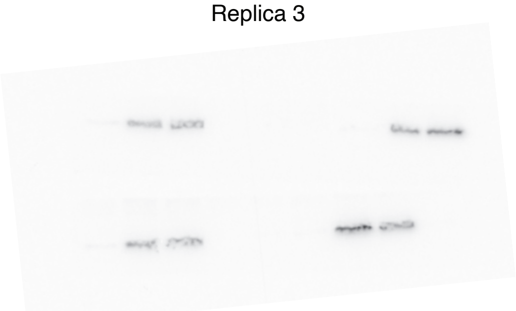

Replica 4



Replica 5

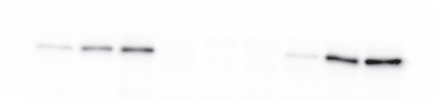

Replica 6 (Data shown in Figure 1A)



## Anti-$His_6$

Replica 1
Replica 2
Replica 3

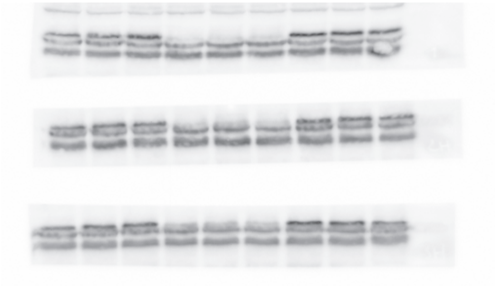

Replica 4

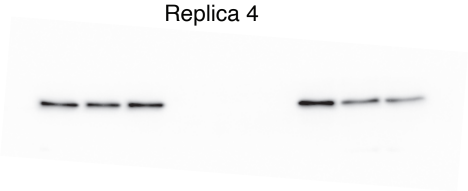

Replica 5

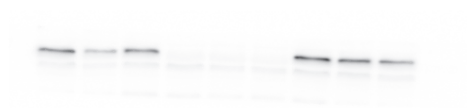

Replica 6 (Data shown in Figure 1A)

# Figure 1C

Loading order:

| | Exp | -C; DMSO |
|---|---|---|
| min | | 2 3 4 5 6 7 8 9 10 11 12 15 |

| | Exp | -C; 2NM-PP1 |
|---|---|---|
| min | | 2 3 4 5 6 7 8 9 10 11 12 15 |

## Anti-Sch9-pThr$^{737}$

Replica 1

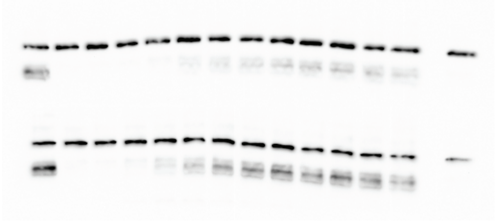

Replica 2

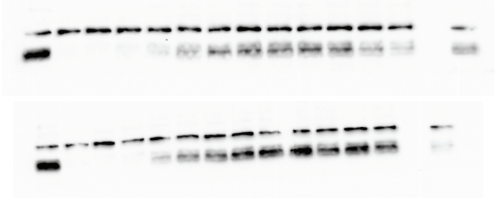

Replica 3

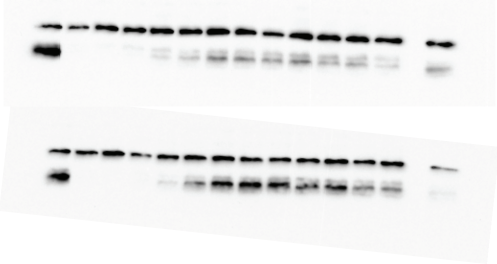

Replica 4 (Data shown in Figure 1C)

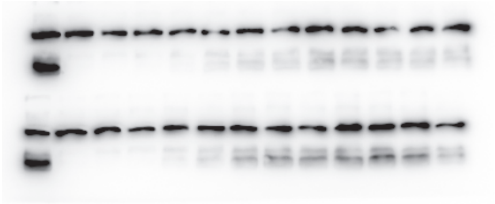

## Anti-Sch9

Replica 1

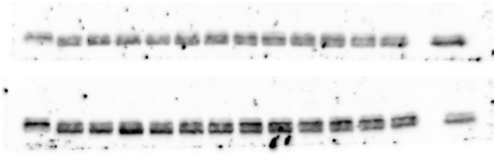

Replica 2

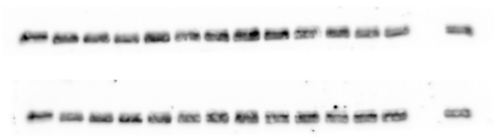

Replica 3

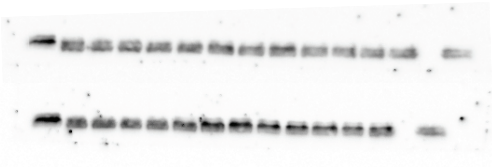

Replica 4 (Data shown in Figure 1C)

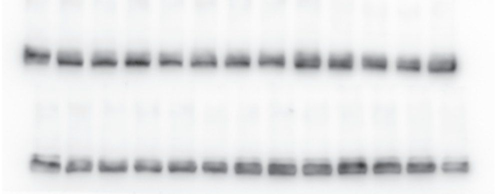

## Anti-Snf1-pThr$^{210}$

Replica 1

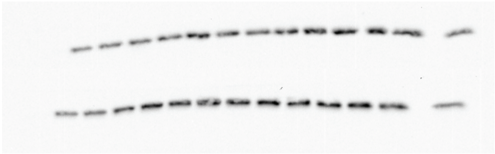

Replica 2

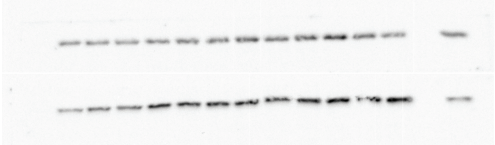

Replica 3

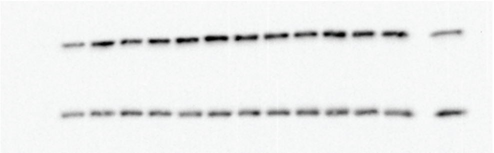

Replica 4 (Data shown in Figure 1C)

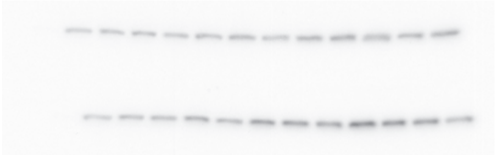

## Anti-His$_6$

Replica 1

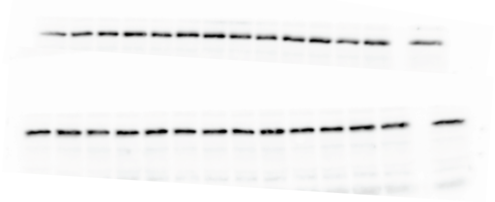

Replica 2

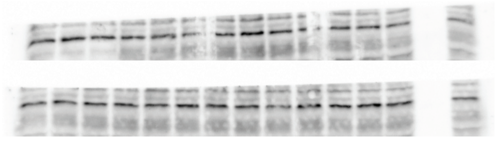

Replica 3

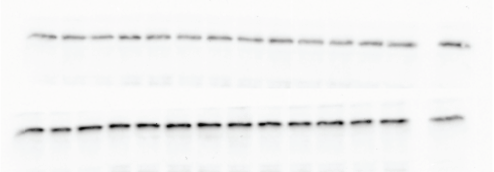

Replica 4 (Data shown in Figure 1C)

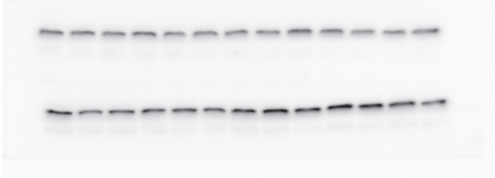

# Figure 1E

Loading order: WT *reg1Δ*

Replica 4 (Data shown in Figure 1E)

Anti-Sch9-pThr$^{737}$

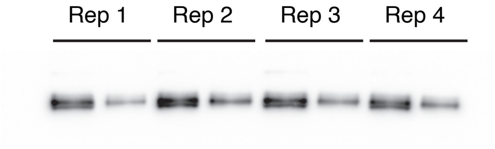


Anti-Sch9

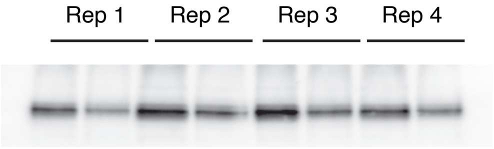


Anti-Snf1-pThr$^{210}$

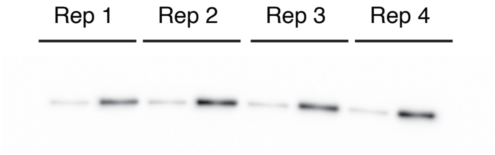


Anti-$His_6$

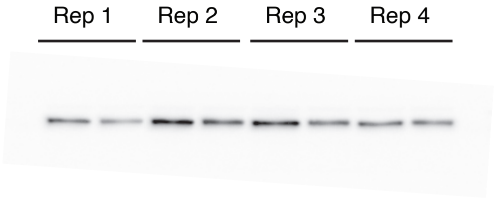

# Figure 1F

Spotting order:

WT
*gtr1Δ*
*snf1Δ*
*snf1*$^{as}$
*reg1Δ*

## Control

Replica 1

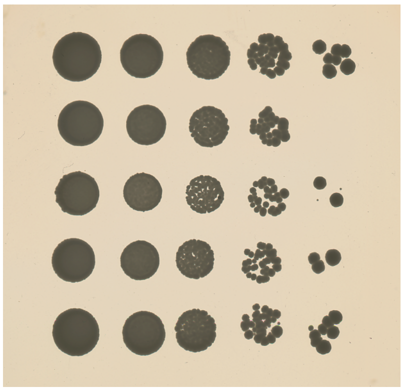

Replica 2 (Data shown in Figure 1F)

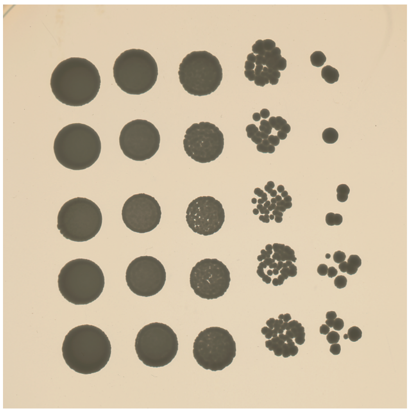

Replica 3

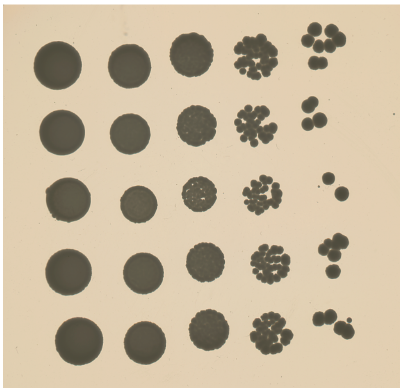

## 3 nM Rapamycin

Replica 1

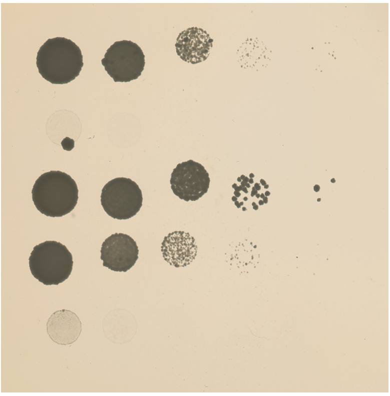

Replica 2 (Data shown in Figure 1F)

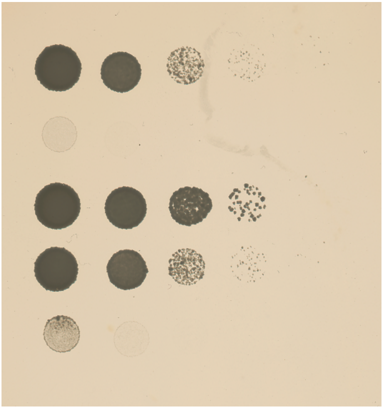

Replica 3

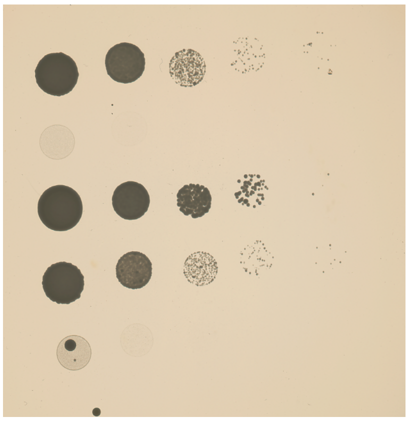

Supplement: Figure 1—source data 2. [file elife-84319-fig1-data2.pdf]
