# Supplementary figures and images for: Snf1/AMPK fine-tunes TORC1 signaling in response to glucose starvation (part 1 of 2)
Source: eLife. 2023 Feb 7;12:e84319. doi: 10.7554/eLife.84319 (PMC9937656; doi:10.7554/eLife.84319)

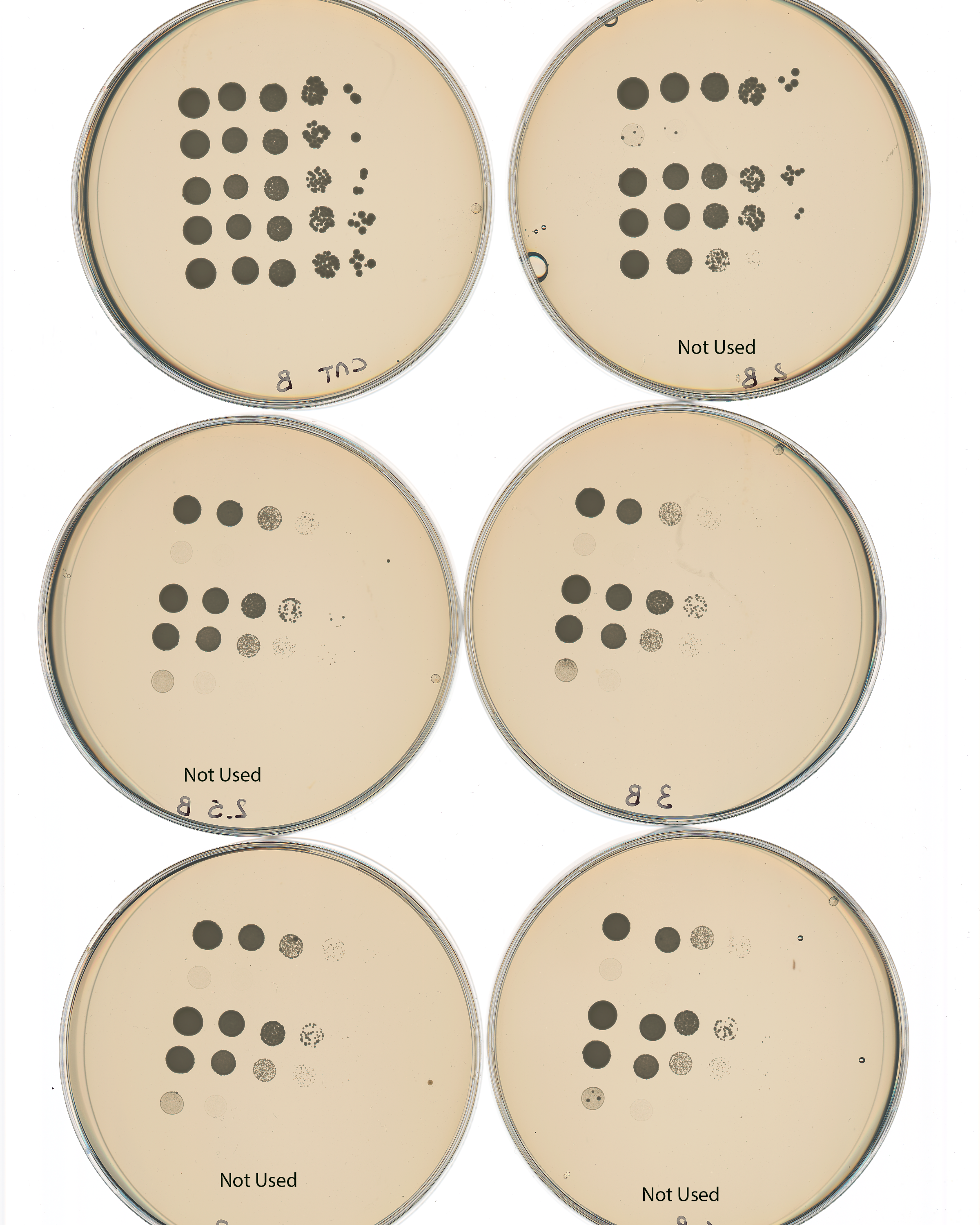

Supplement: Figure 1—source data 3. [file elife-84319-fig1-data3.zip › Figure 1ΓÇôSource Data 3/Figure 1F/Replica 2.tif]

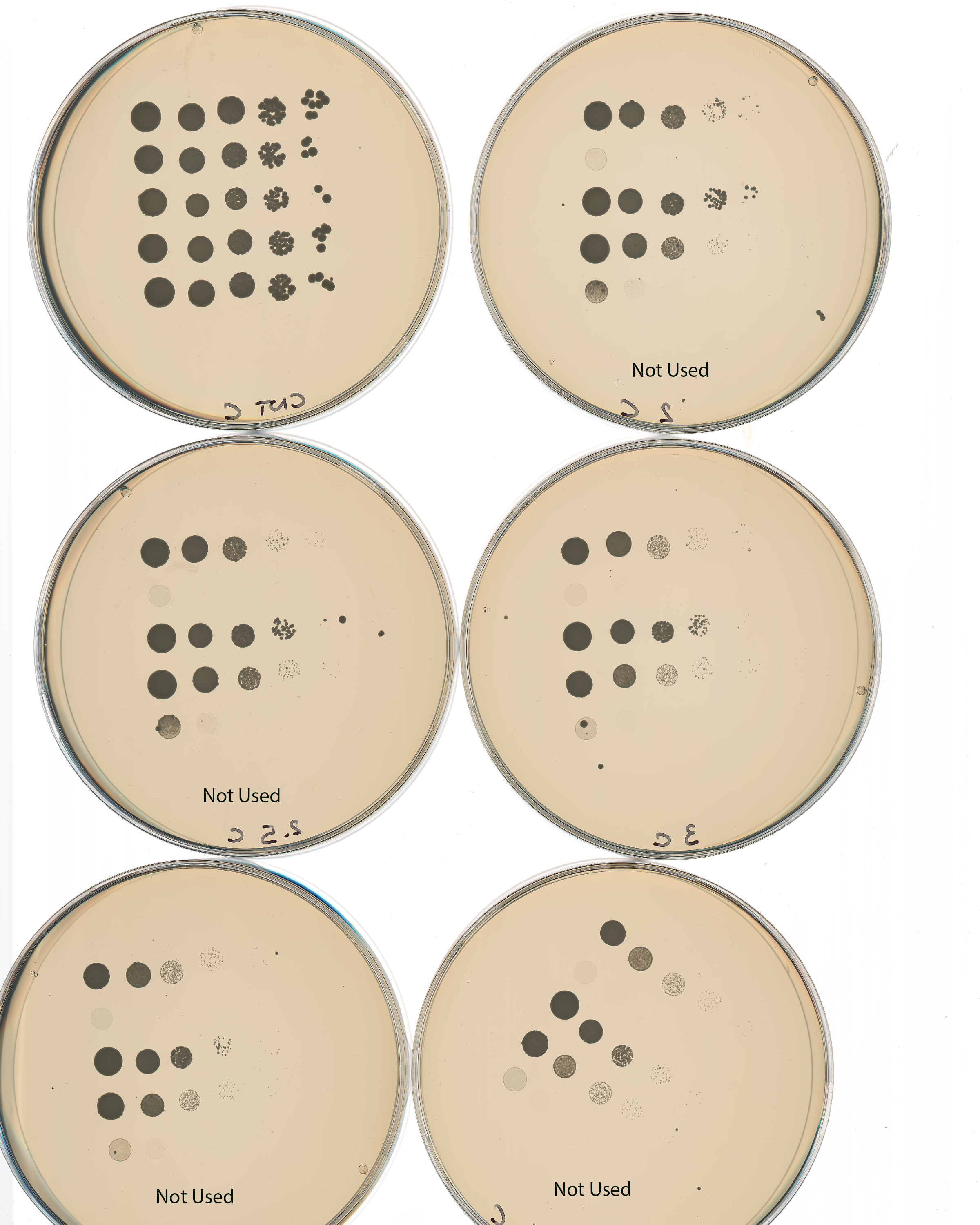

Supplement: Figure 1—source data 3. [file elife-84319-fig1-data3.zip › Figure 1ΓÇôSource Data 3/Figure 1F/Replica 3.tif]

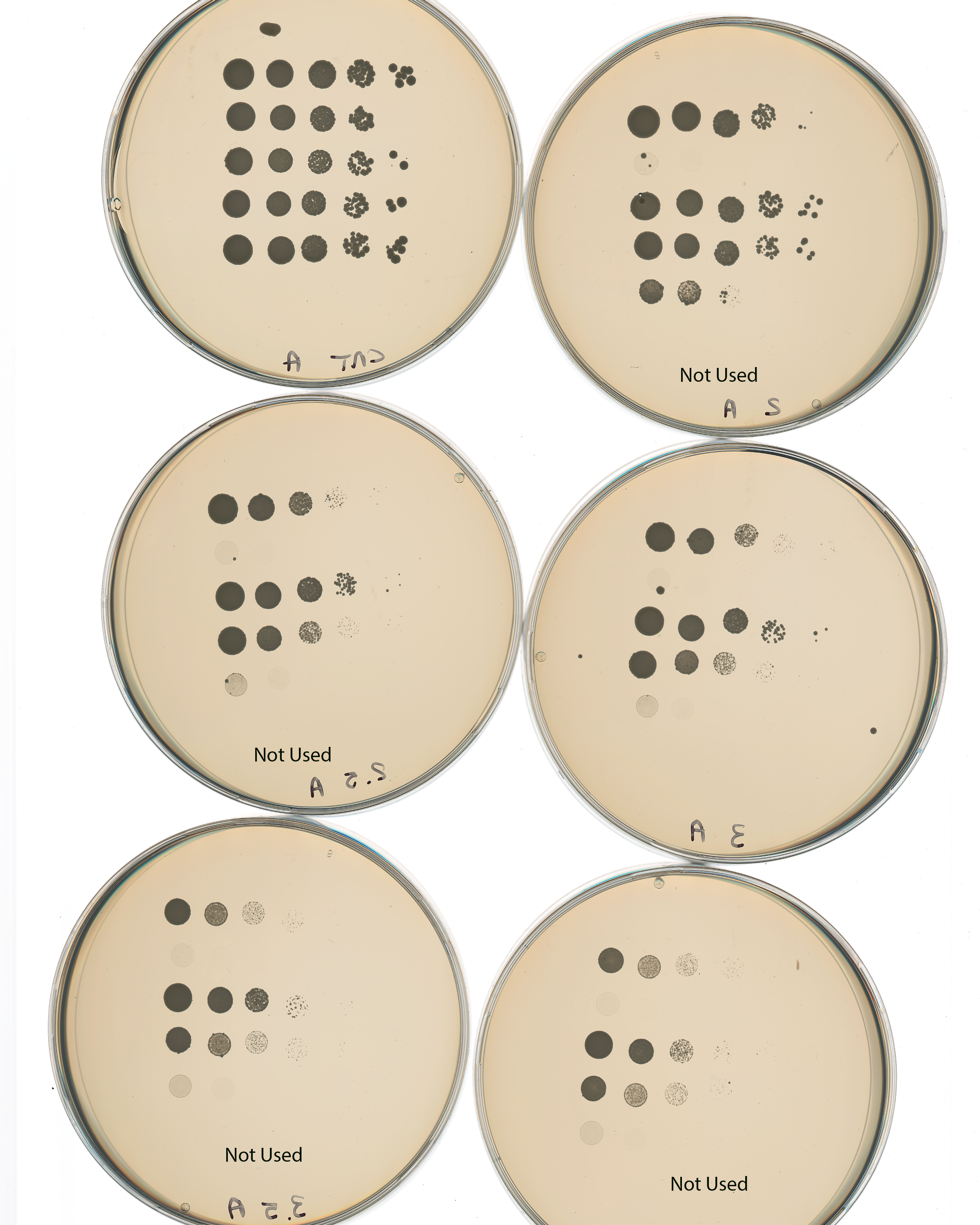

Supplement: Figure 1—source data 3. [file elife-84319-fig1-data3.zip › Figure 1ΓÇôSource Data 3/Figure 1F/Replica 1.tif]

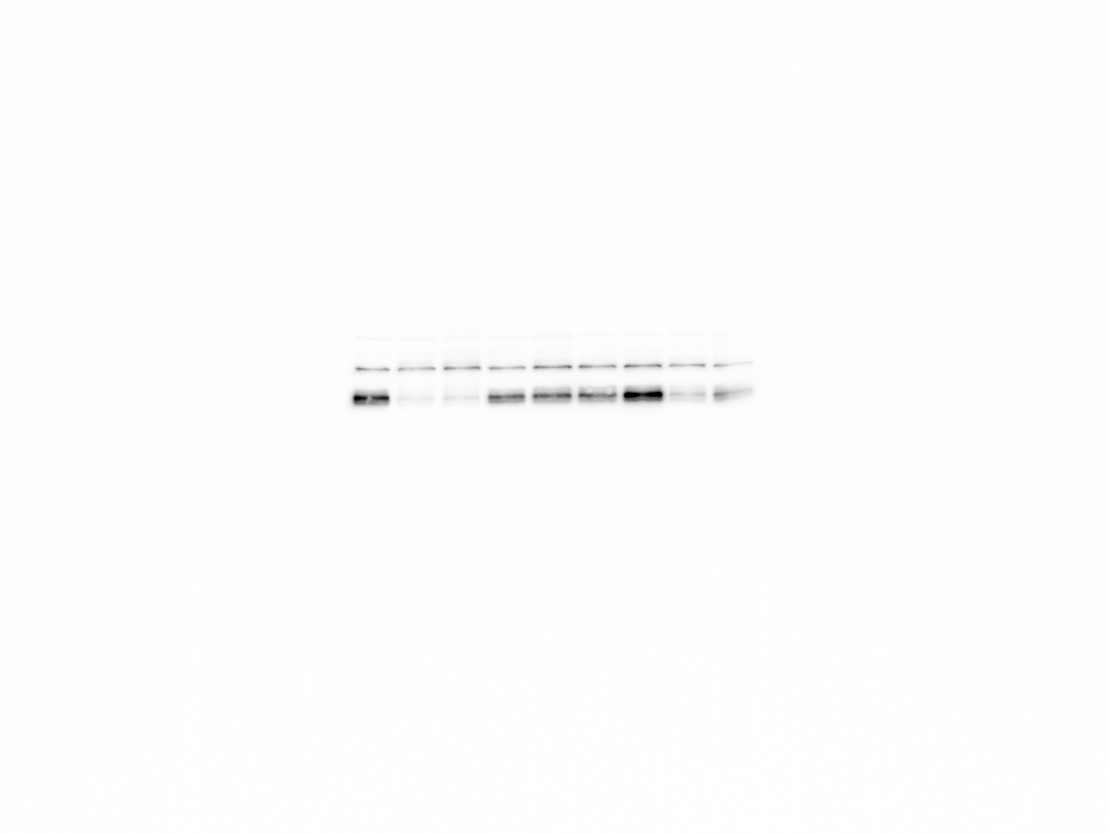

Supplement: Figure 1—source data 3. [file elife-84319-fig1-data3.zip › Figure 1ΓÇôSource Data 3/Figure 1A/Sch9-pThr737/Replica 4.tif]

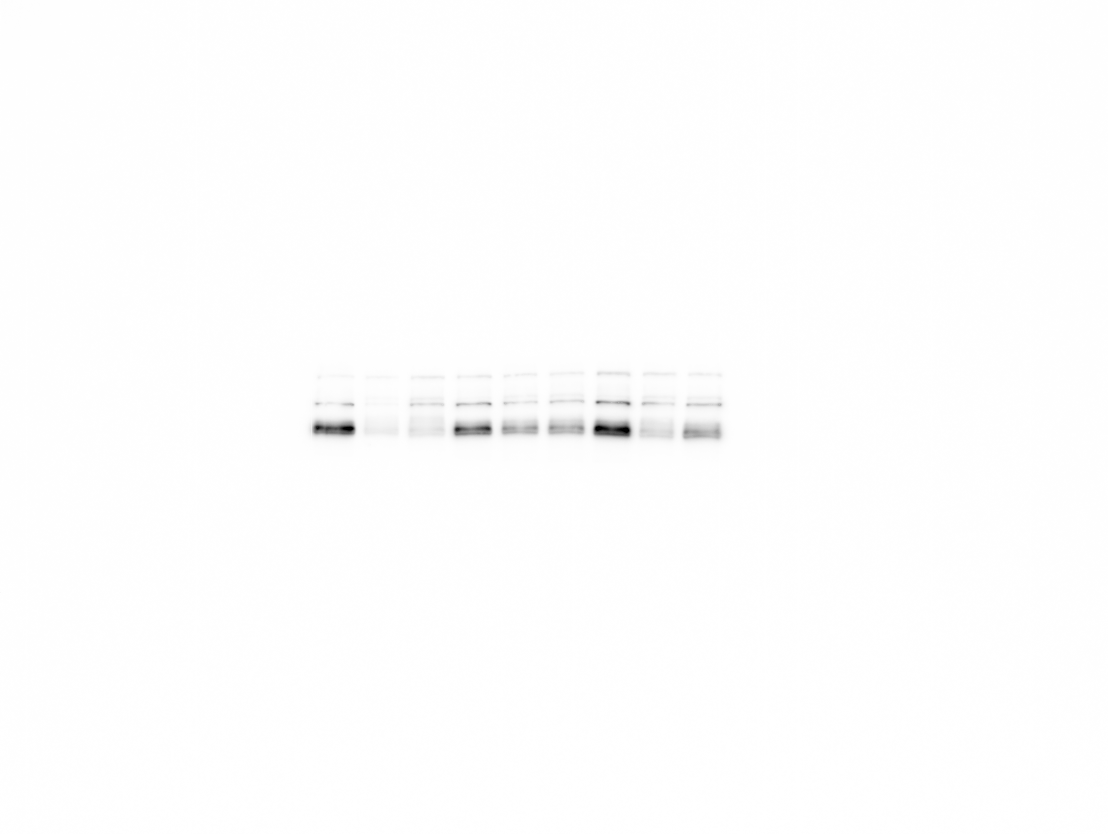

Supplement: Figure 1—source data 3. [file elife-84319-fig1-data3.zip › Figure 1ΓÇôSource Data 3/Figure 1A/Sch9-pThr737/Replica 5.tif]

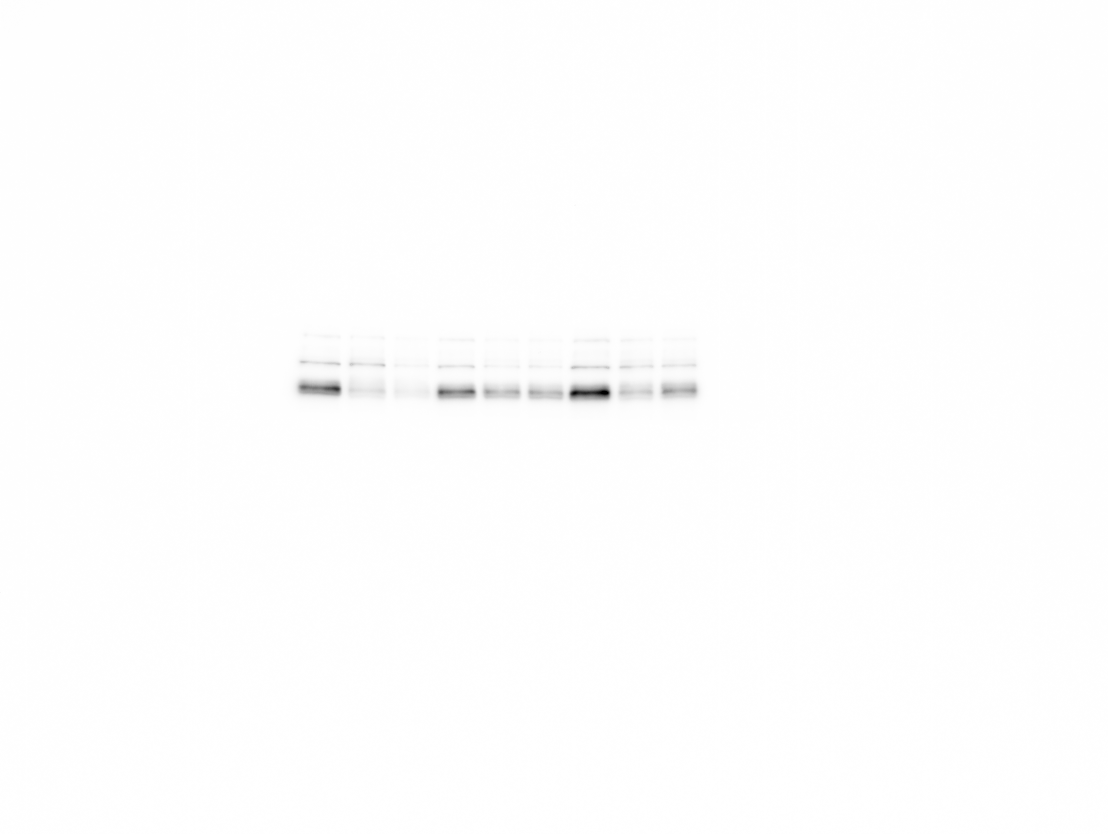

Supplement: Figure 1—source data 3. [file elife-84319-fig1-data3.zip › Figure 1ΓÇôSource Data 3/Figure 1A/Sch9-pThr737/Replica 6.tif]

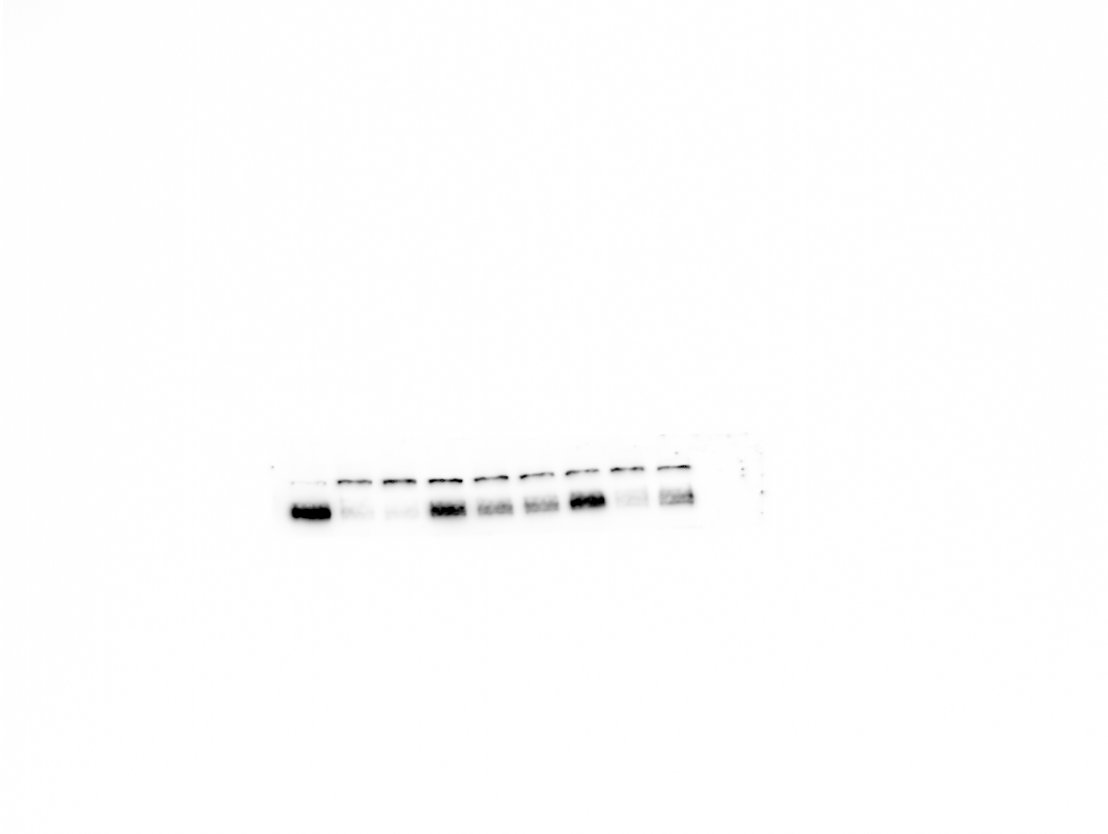

Supplement: Figure 1—source data 3. [file elife-84319-fig1-data3.zip › Figure 1ΓÇôSource Data 3/Figure 1A/Sch9-pThr737/Replica 2.tif]

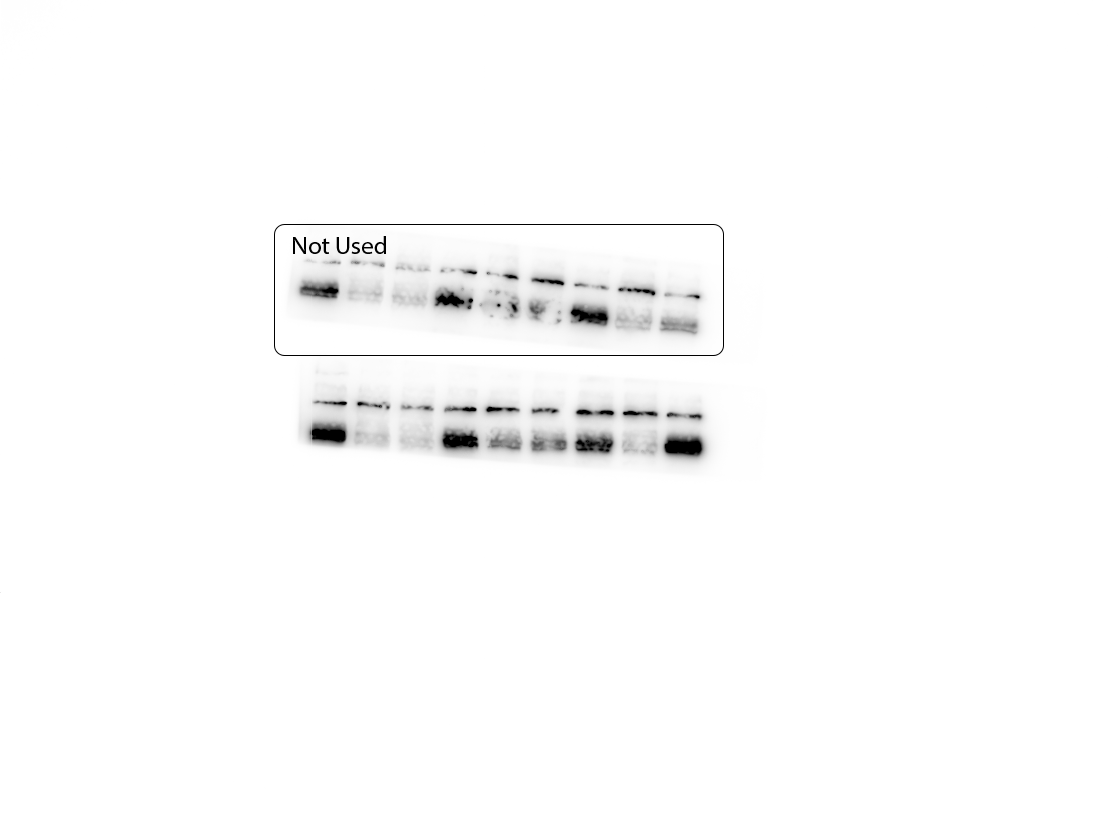

Supplement: Figure 1—source data 3. [file elife-84319-fig1-data3.zip › Figure 1ΓÇôSource Data 3/Figure 1A/Sch9-pThr737/Replica 3.tif]

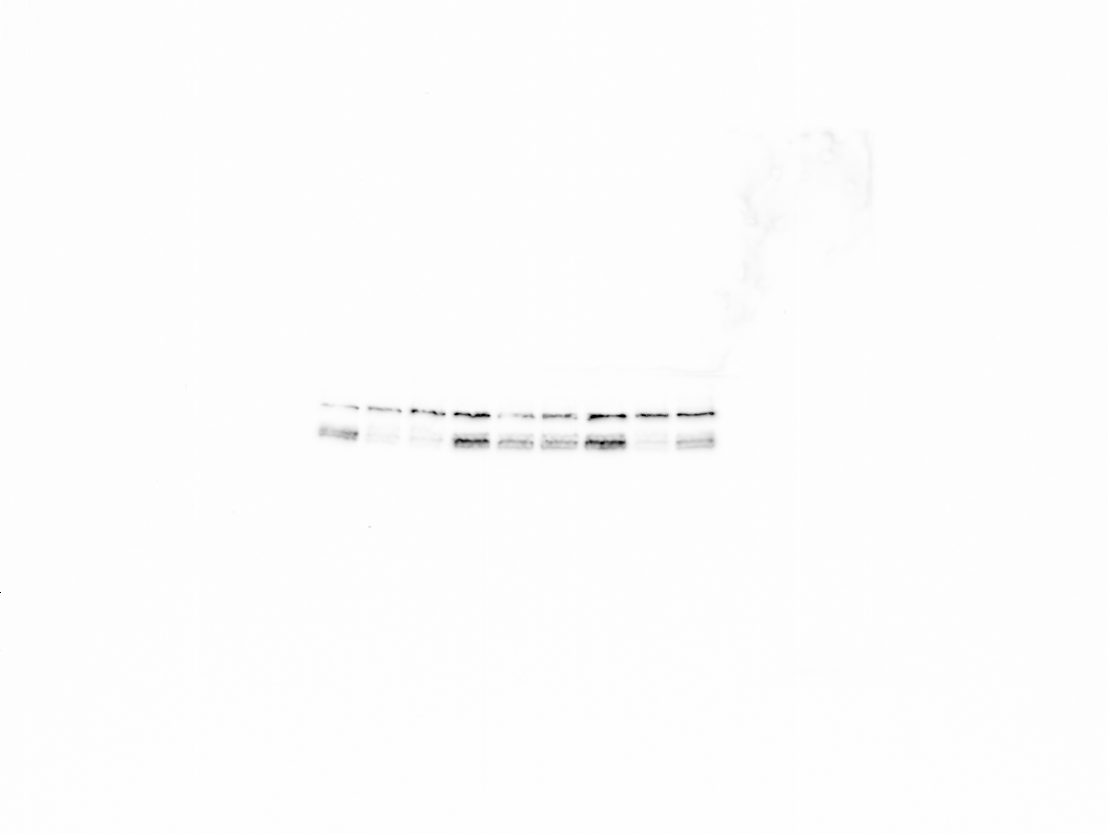

Supplement: Figure 1—source data 3. [file elife-84319-fig1-data3.zip › Figure 1ΓÇôSource Data 3/Figure 1A/Sch9-pThr737/Replica 1.tif]

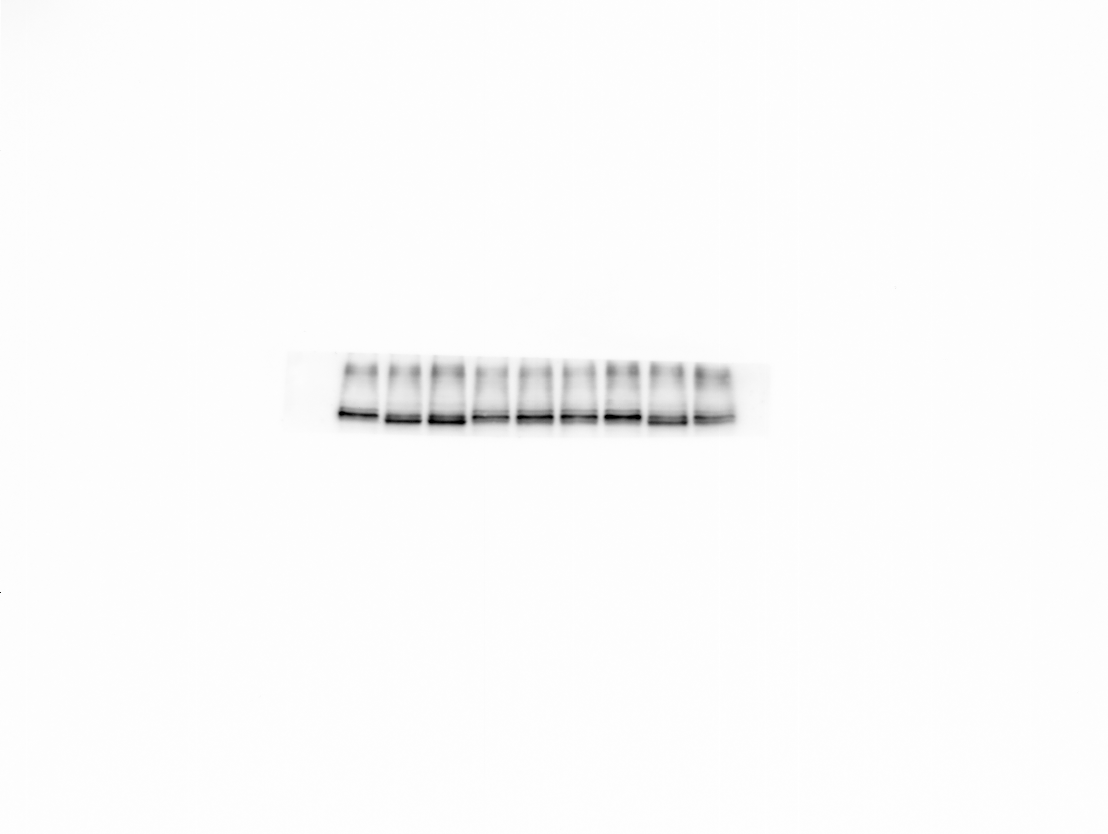

Supplement: Figure 1—source data 3. [file elife-84319-fig1-data3.zip › Figure 1ΓÇôSource Data 3/Figure 1A/Sch9/Replica 4.tif]

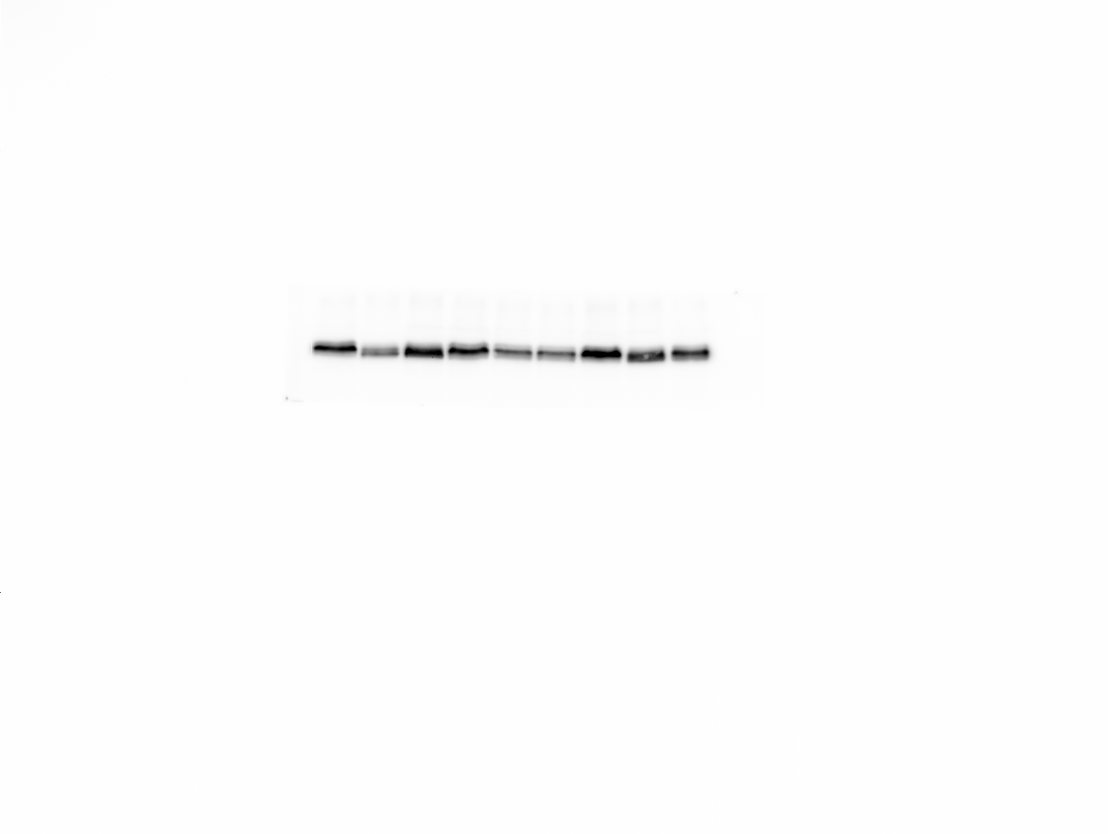

Supplement: Figure 1—source data 3. [file elife-84319-fig1-data3.zip › Figure 1ΓÇôSource Data 3/Figure 1A/Sch9/Replica 5.tif]

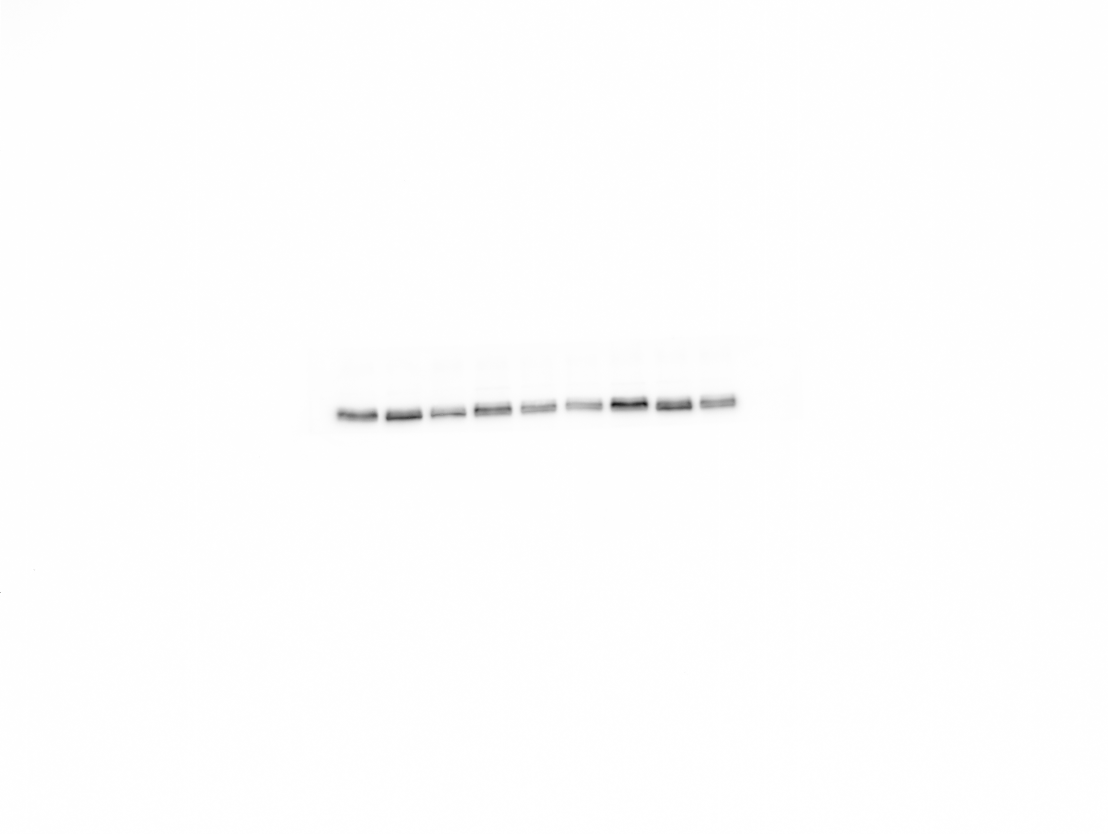

Supplement: Figure 1—source data 3. [file elife-84319-fig1-data3.zip › Figure 1ΓÇôSource Data 3/Figure 1A/Sch9/Replica 6.tif]

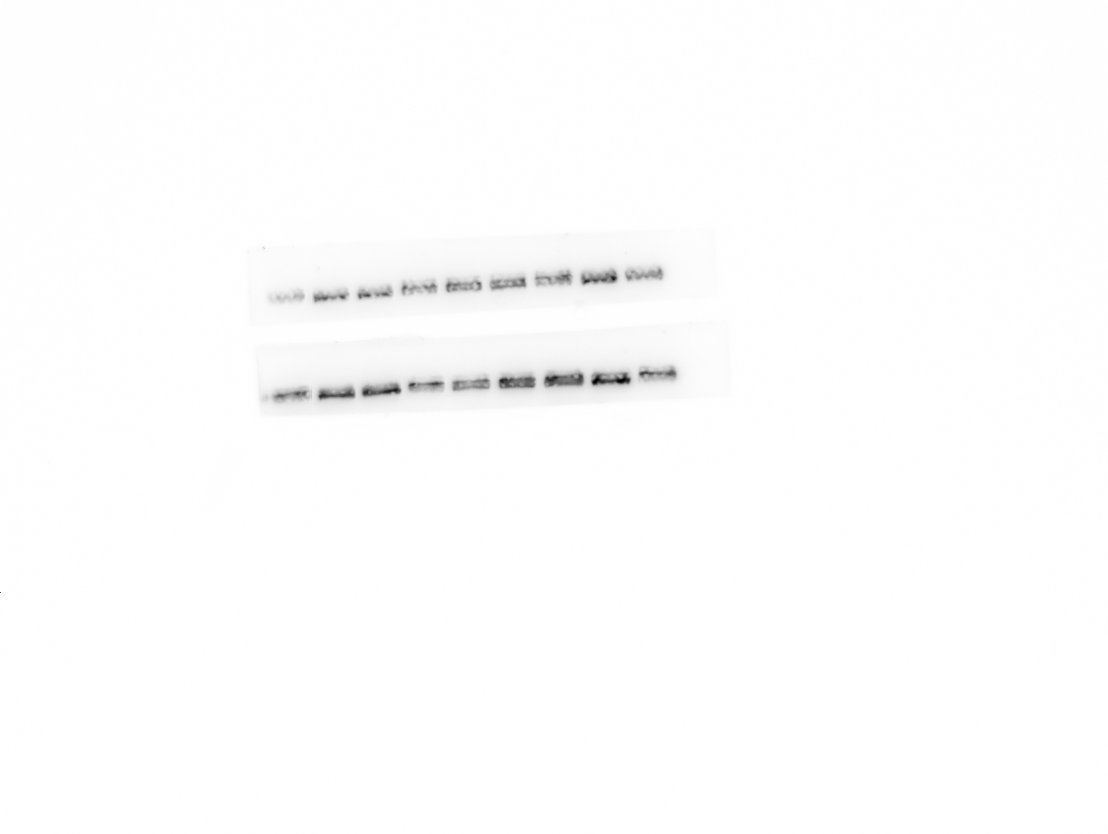

Supplement: Figure 1—source data 3. [file elife-84319-fig1-data3.zip › Figure 1ΓÇôSource Data 3/Figure 1A/Sch9/Replica 2_3.tif]

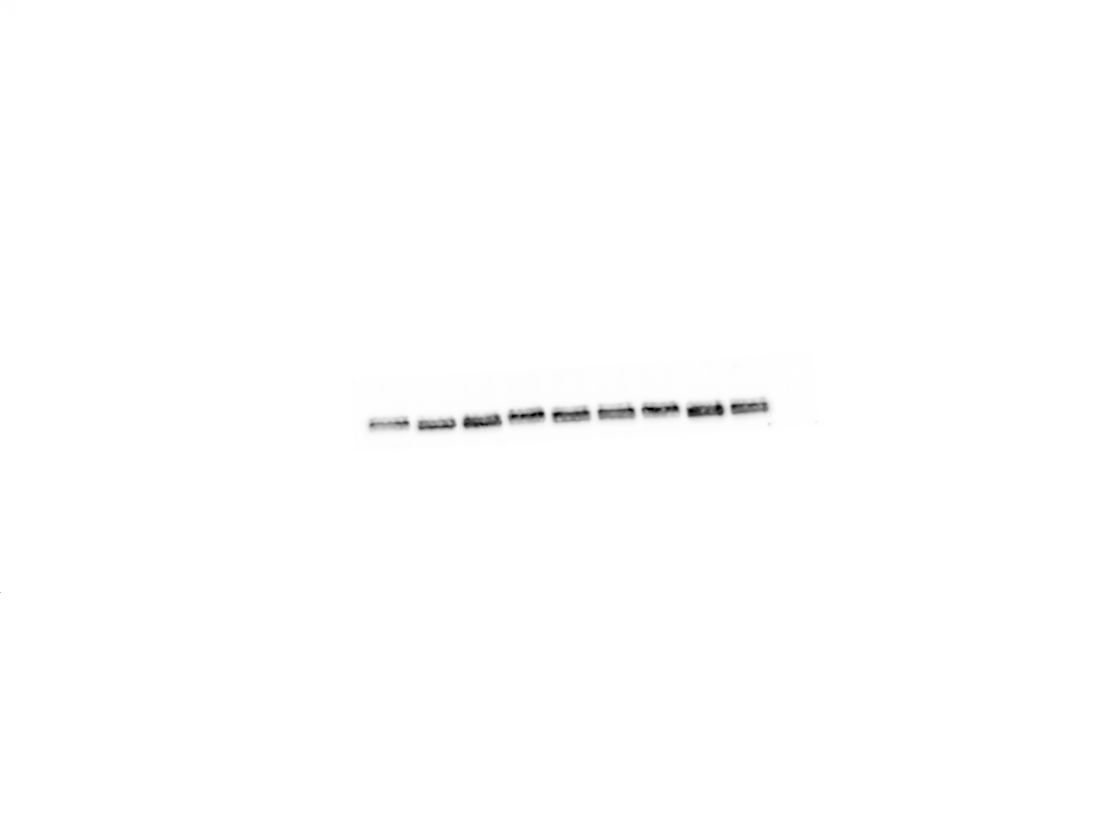

Supplement: Figure 1—source data 3. [file elife-84319-fig1-data3.zip › Figure 1ΓÇôSource Data 3/Figure 1A/Sch9/Replica 1.tif]

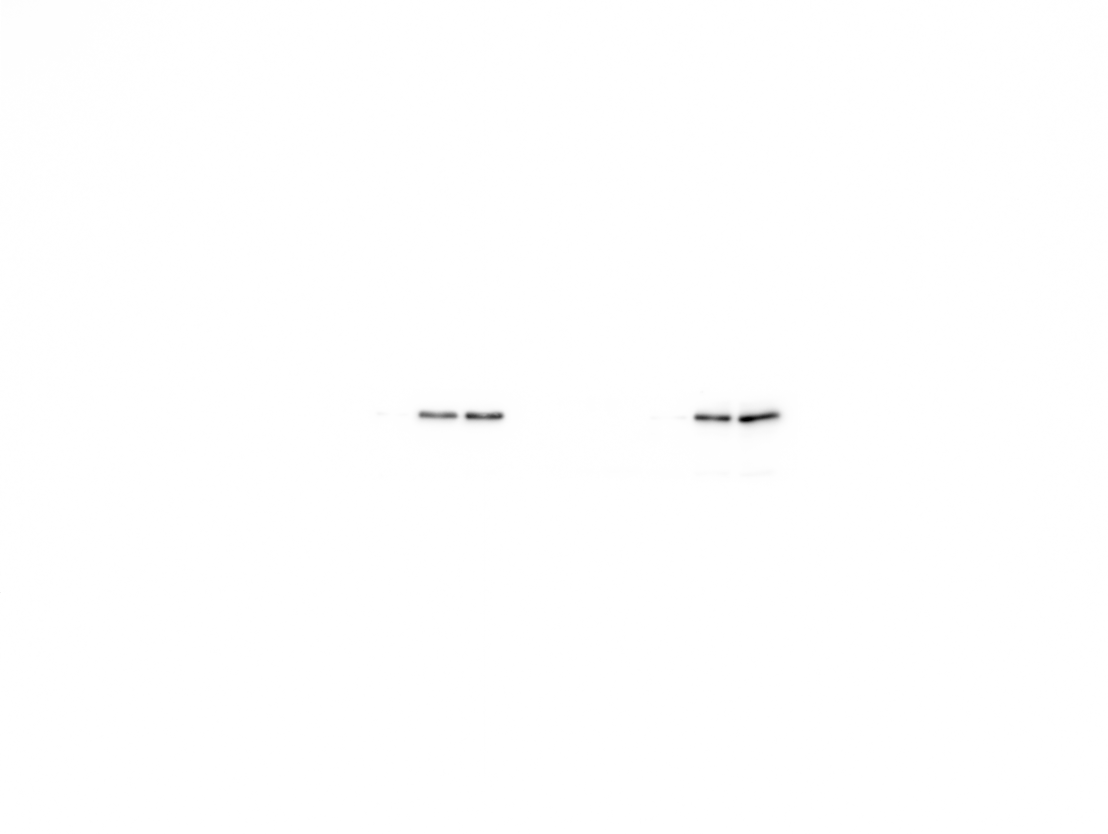

Supplement: Figure 1—source data 3. [file elife-84319-fig1-data3.zip › Figure 1ΓÇôSource Data 3/Figure 1A/Snf1-pThr210/Replica 4.tif]

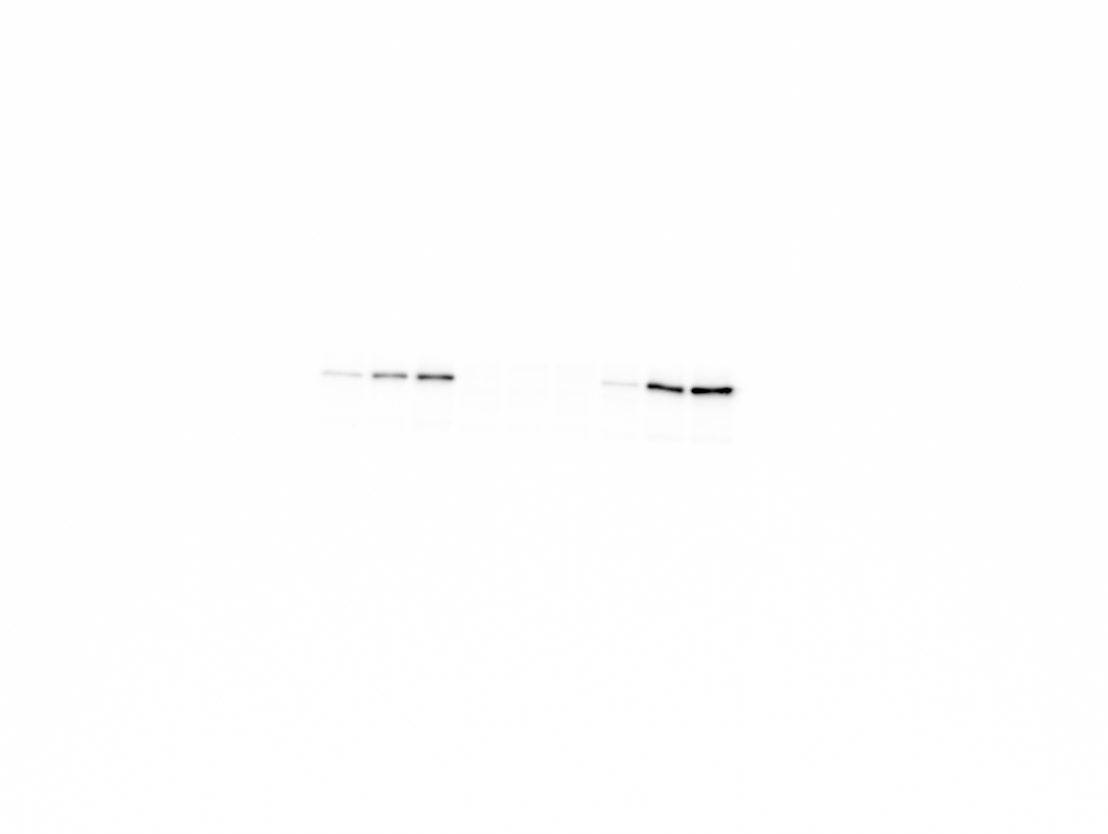

Supplement: Figure 1—source data 3. [file elife-84319-fig1-data3.zip › Figure 1ΓÇôSource Data 3/Figure 1A/Snf1-pThr210/Replica 5.tif]

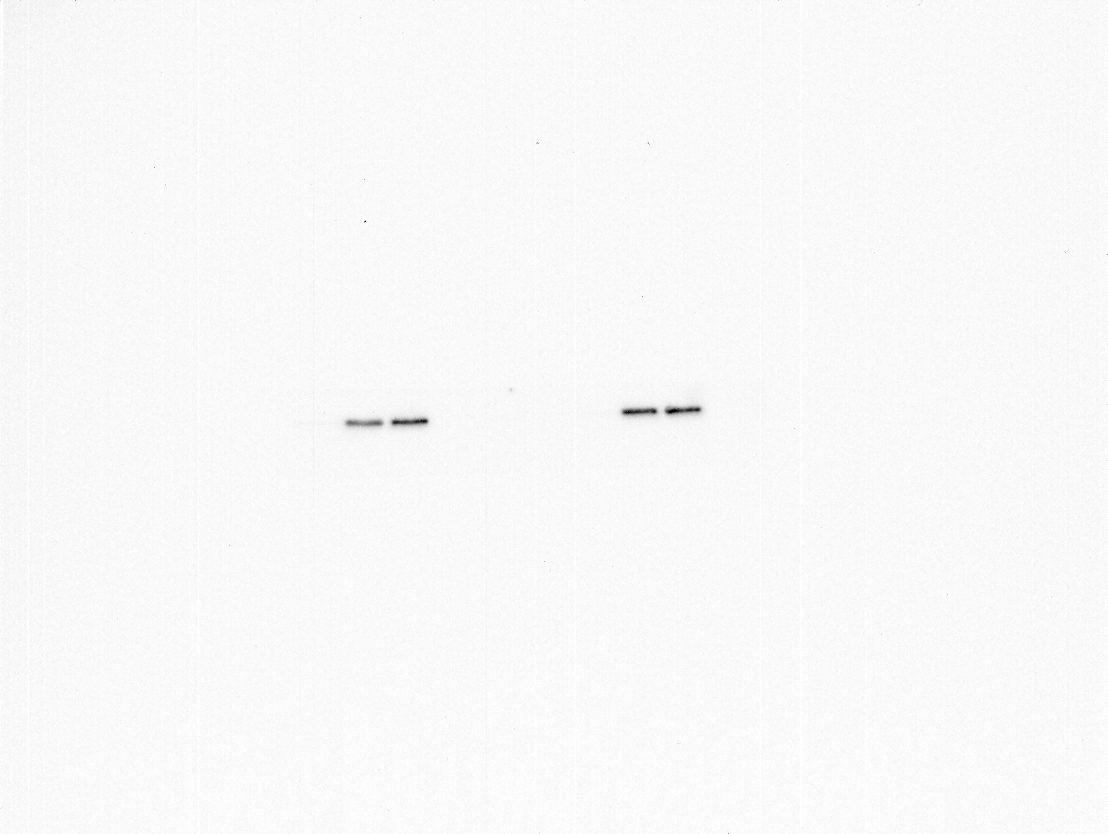

Supplement: Figure 1—source data 3. [file elife-84319-fig1-data3.zip › Figure 1ΓÇôSource Data 3/Figure 1A/Snf1-pThr210/Replica 2.tif]

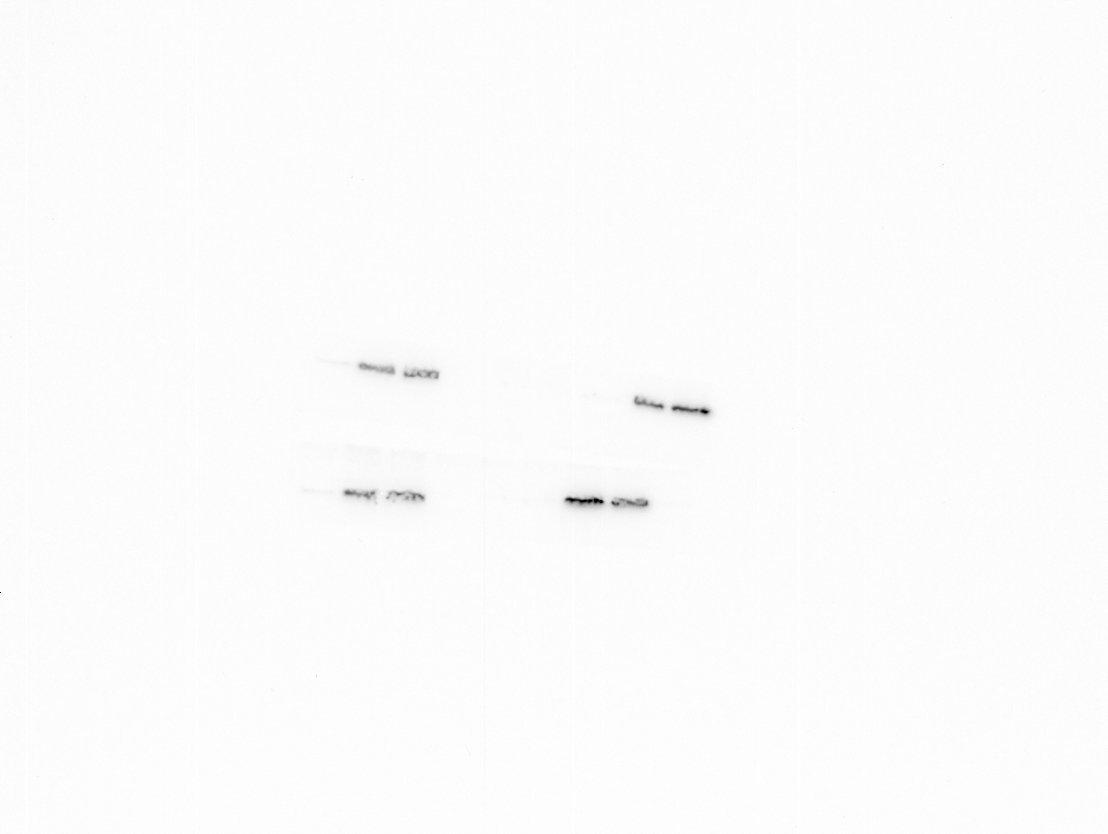

Supplement: Figure 1—source data 3. [file elife-84319-fig1-data3.zip › Figure 1ΓÇôSource Data 3/Figure 1A/Snf1-pThr210/Replica 3.tif]

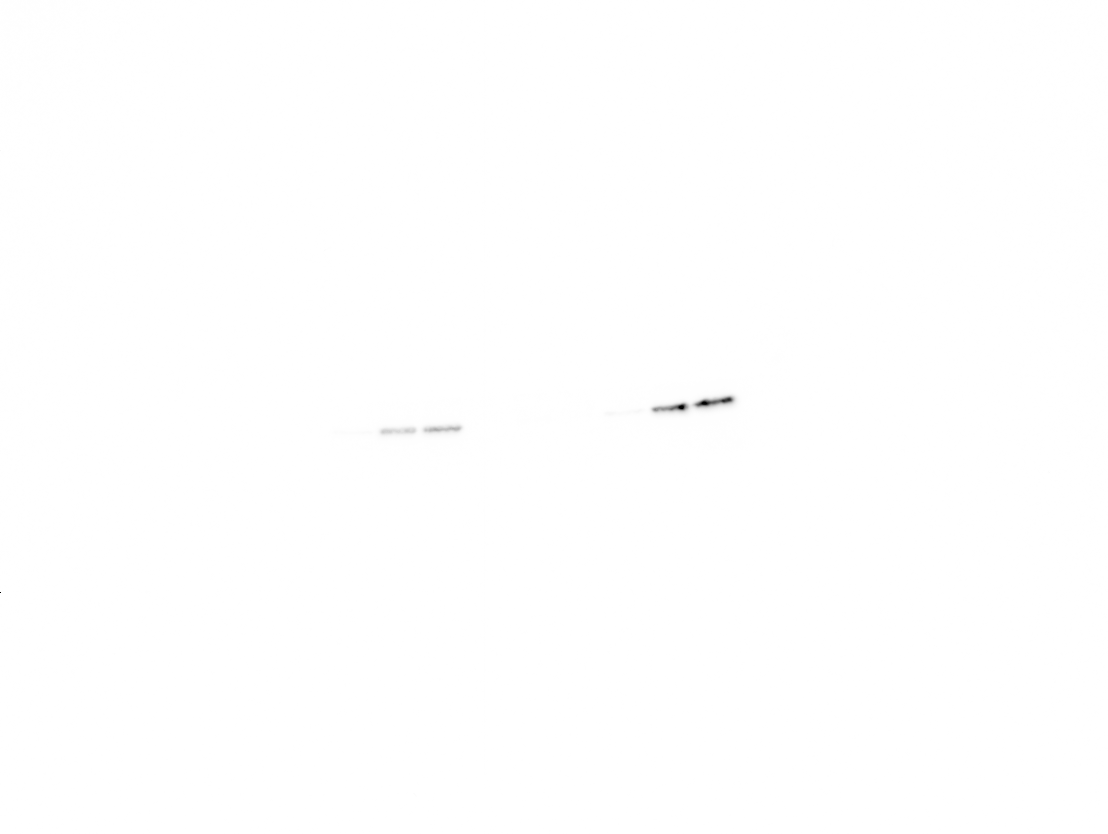

Supplement: Figure 1—source data 3. [file elife-84319-fig1-data3.zip › Figure 1ΓÇôSource Data 3/Figure 1A/Snf1-pThr210/Replica 1.tif]

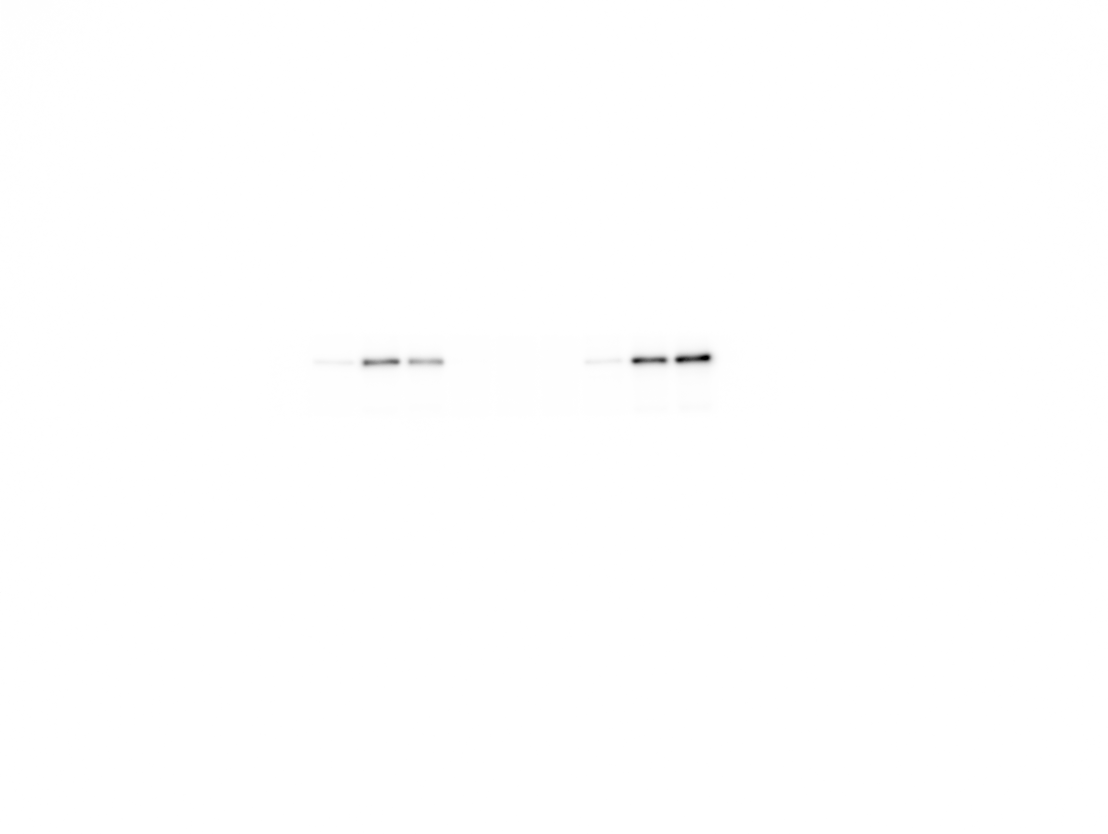

Supplement: Figure 1—source data 3. [file elife-84319-fig1-data3.zip › Figure 1ΓÇôSource Data 3/Figure 1A/Snf1-pThr210/Replica 6.tif.tif]

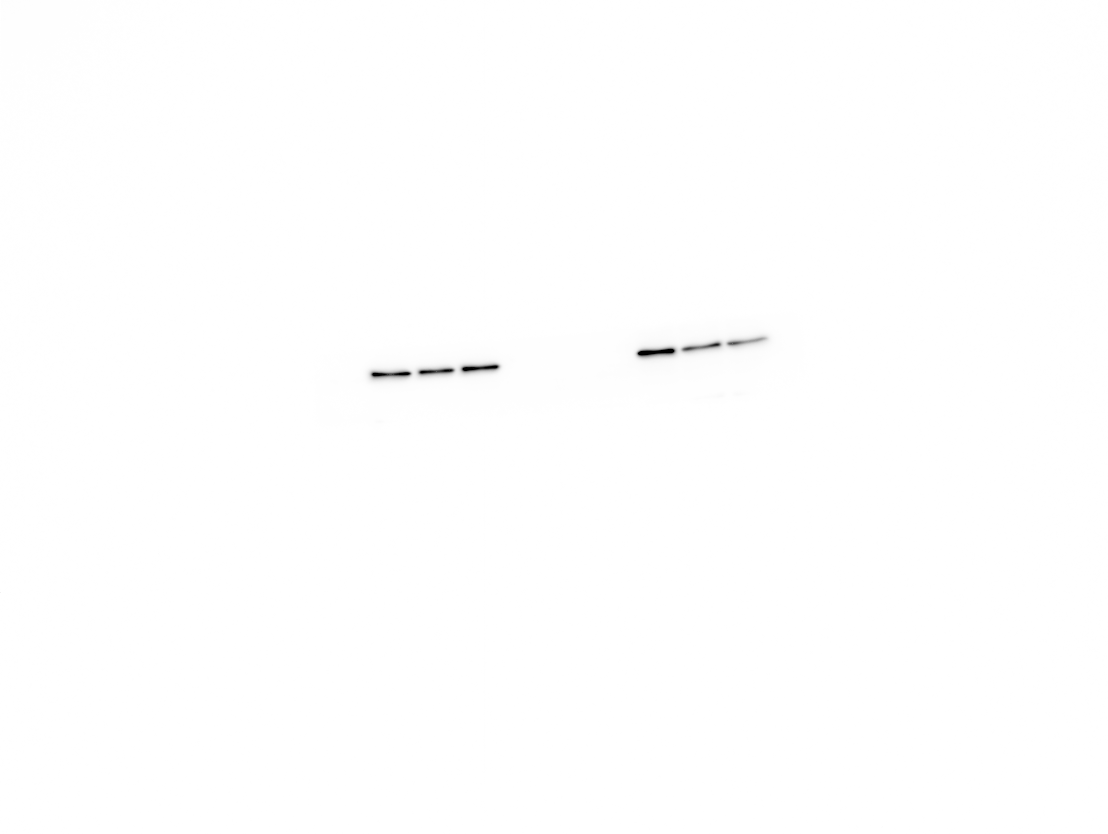

Supplement: Figure 1—source data 3. [file elife-84319-fig1-data3.zip › Figure 1ΓÇôSource Data 3/Figure 1A/His6/Replica 4.tif]

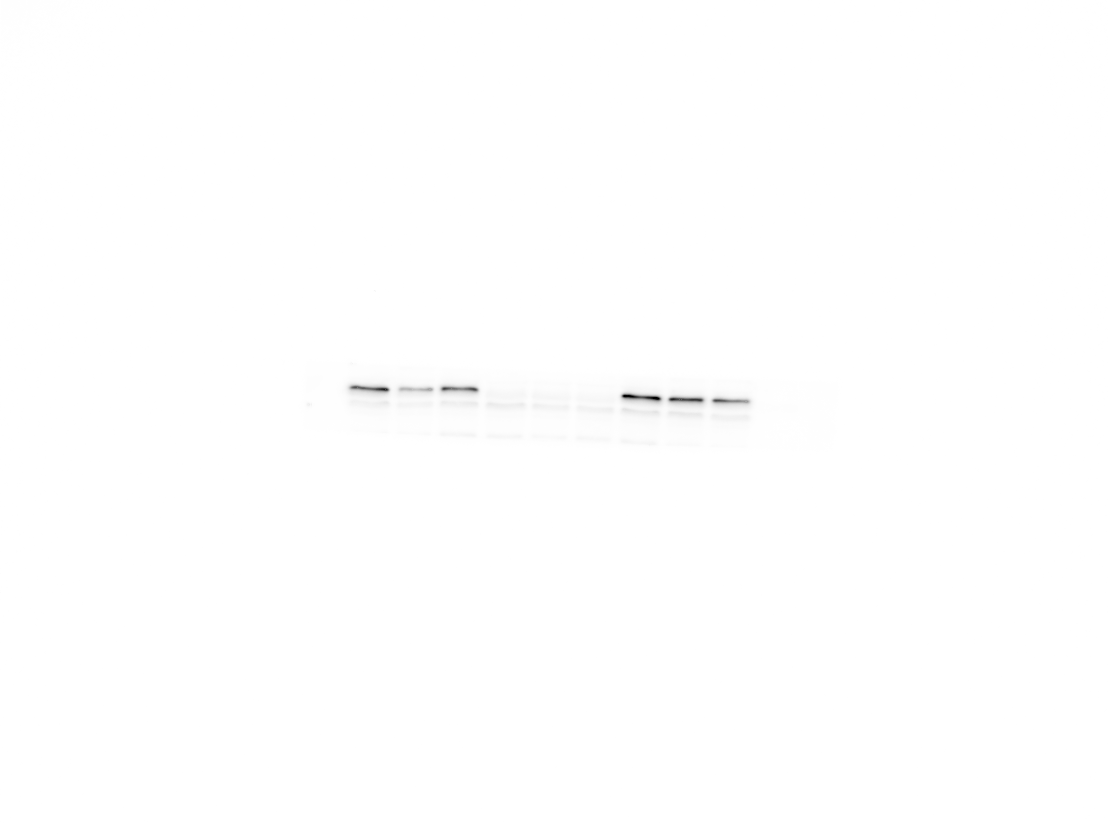

Supplement: Figure 1—source data 3. [file elife-84319-fig1-data3.zip › Figure 1ΓÇôSource Data 3/Figure 1A/His6/Replica 5.tif]

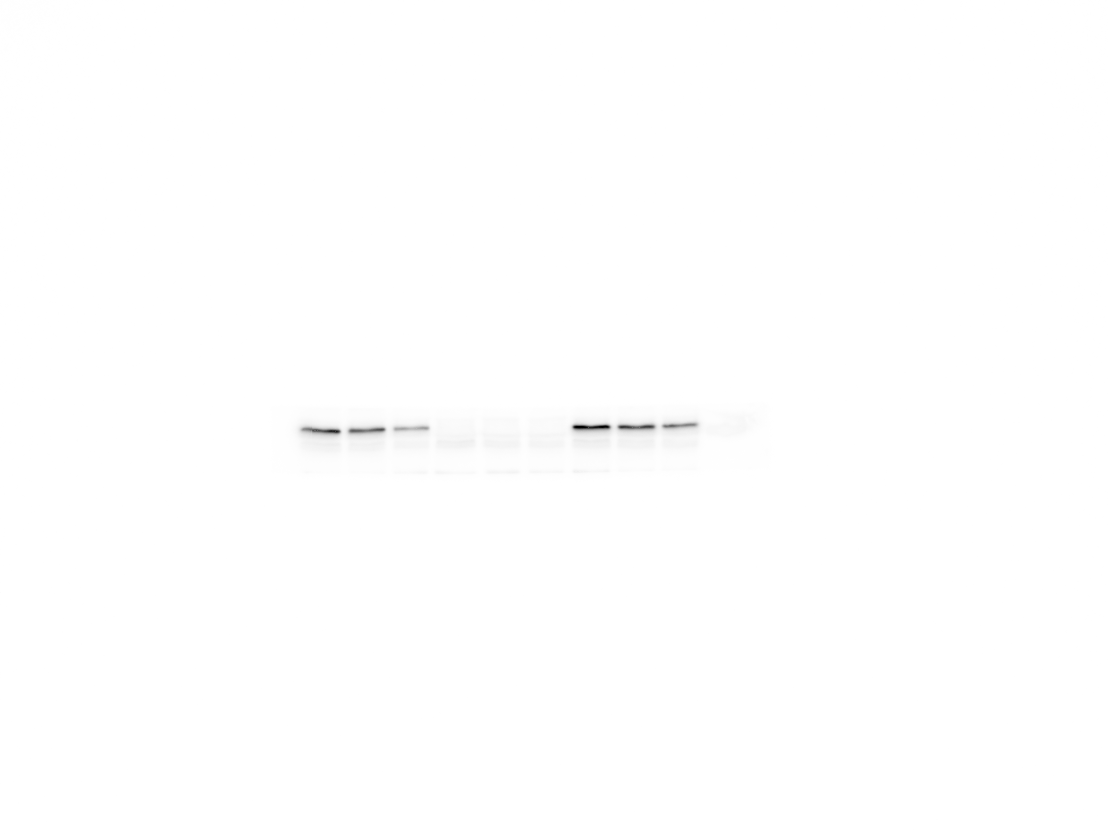

Supplement: Figure 1—source data 3. [file elife-84319-fig1-data3.zip › Figure 1ΓÇôSource Data 3/Figure 1A/His6/Replica 6.tif]

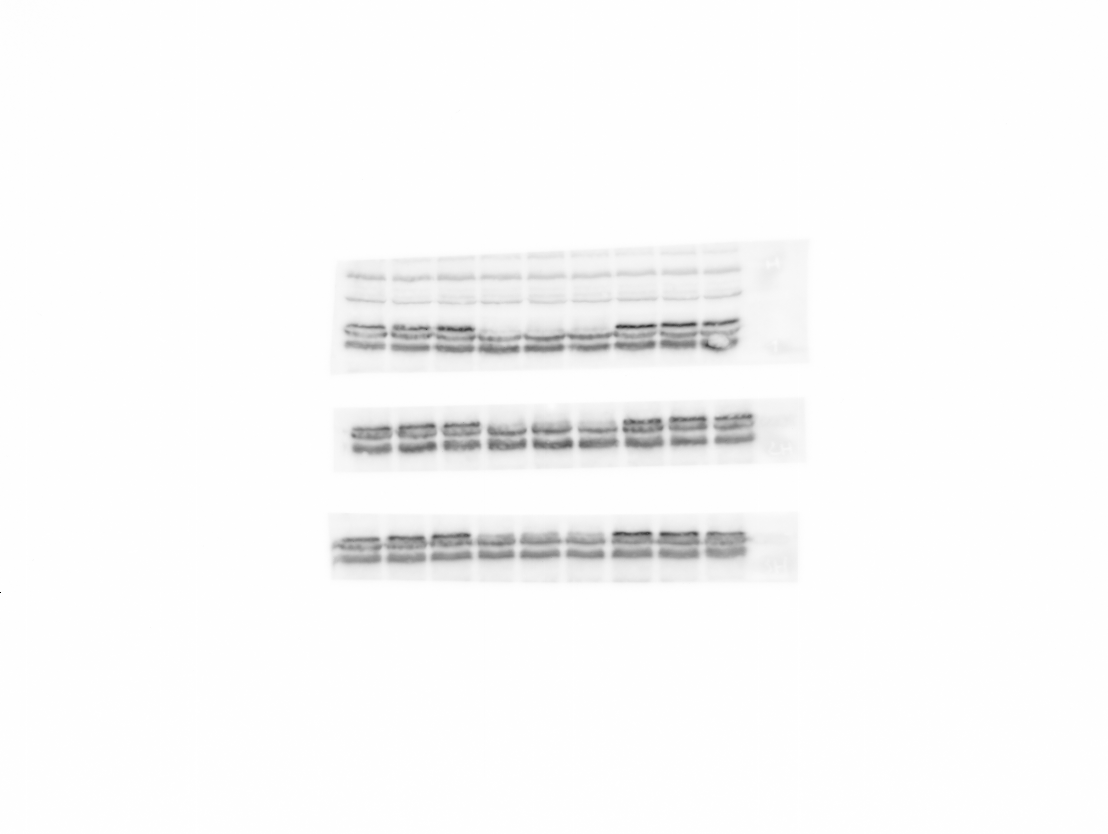

Supplement: Figure 1—source data 3. [file elife-84319-fig1-data3.zip › Figure 1ΓÇôSource Data 3/Figure 1A/His6/Replica 1_2_3.tif]

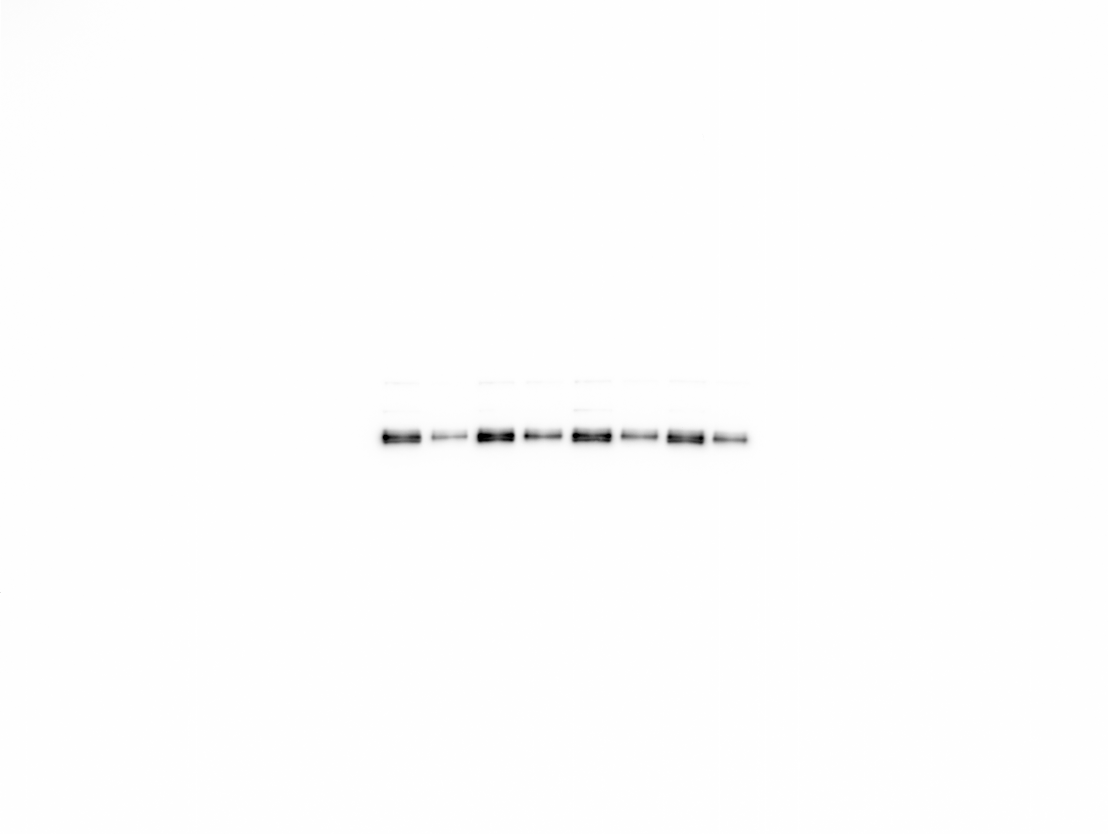

Supplement: Figure 1—source data 3. [file elife-84319-fig1-data3.zip › Figure 1ΓÇôSource Data 3/Figure 1E/Sch9-pThr737/Replica 1_4.tif]

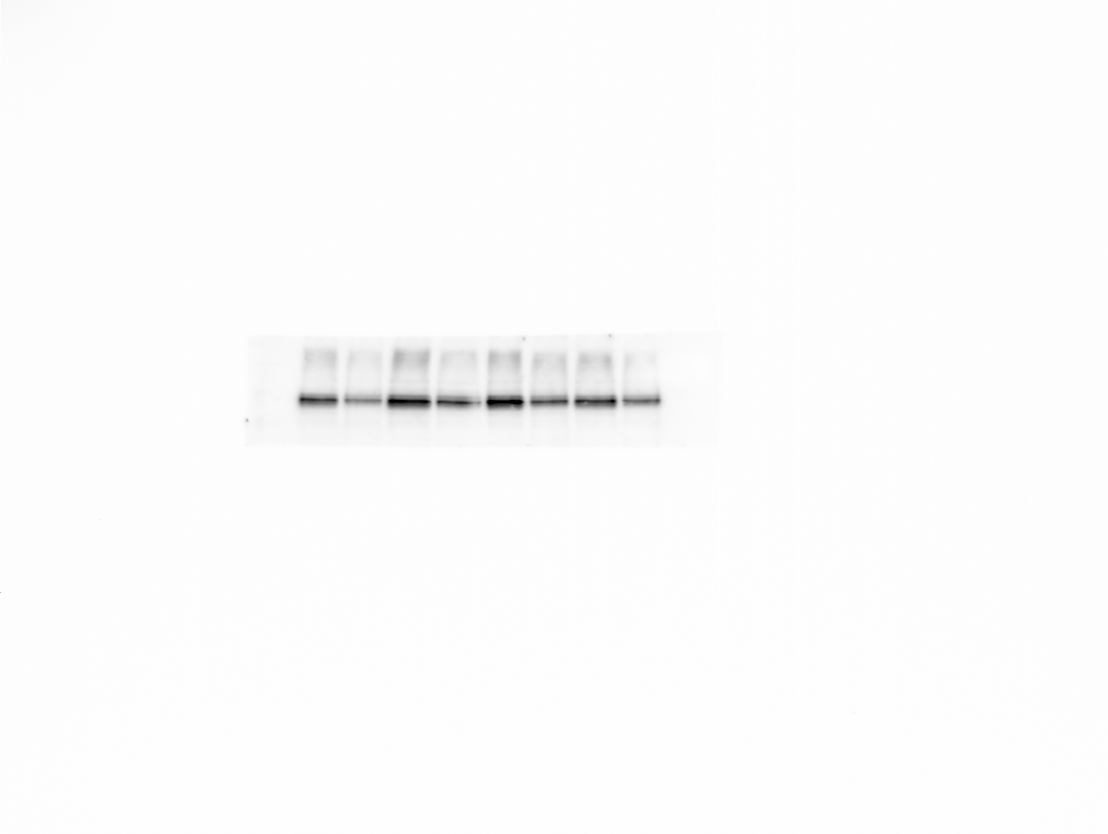

Supplement: Figure 1—source data 3. [file elife-84319-fig1-data3.zip › Figure 1ΓÇôSource Data 3/Figure 1E/Sch9/Replica 1_4.tif]

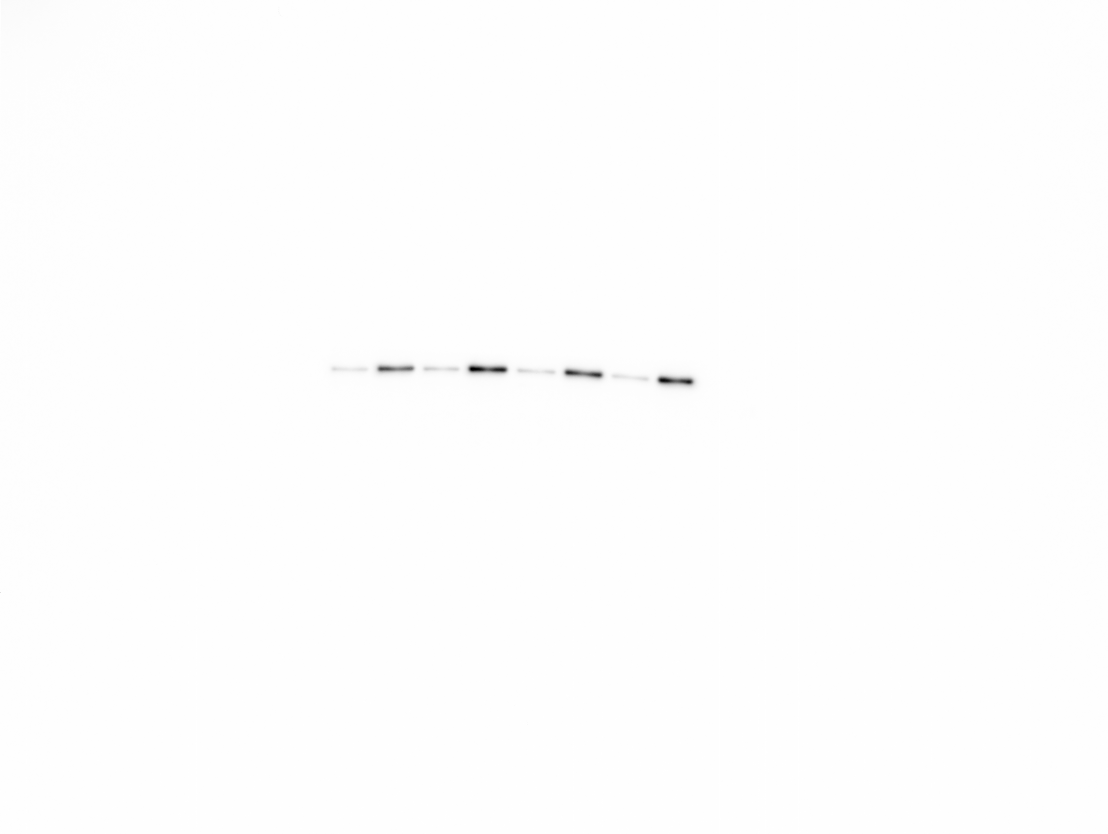

Supplement: Figure 1—source data 3. [file elife-84319-fig1-data3.zip › Figure 1ΓÇôSource Data 3/Figure 1E/Snf1-pThr210/Replica 1_4.tif]

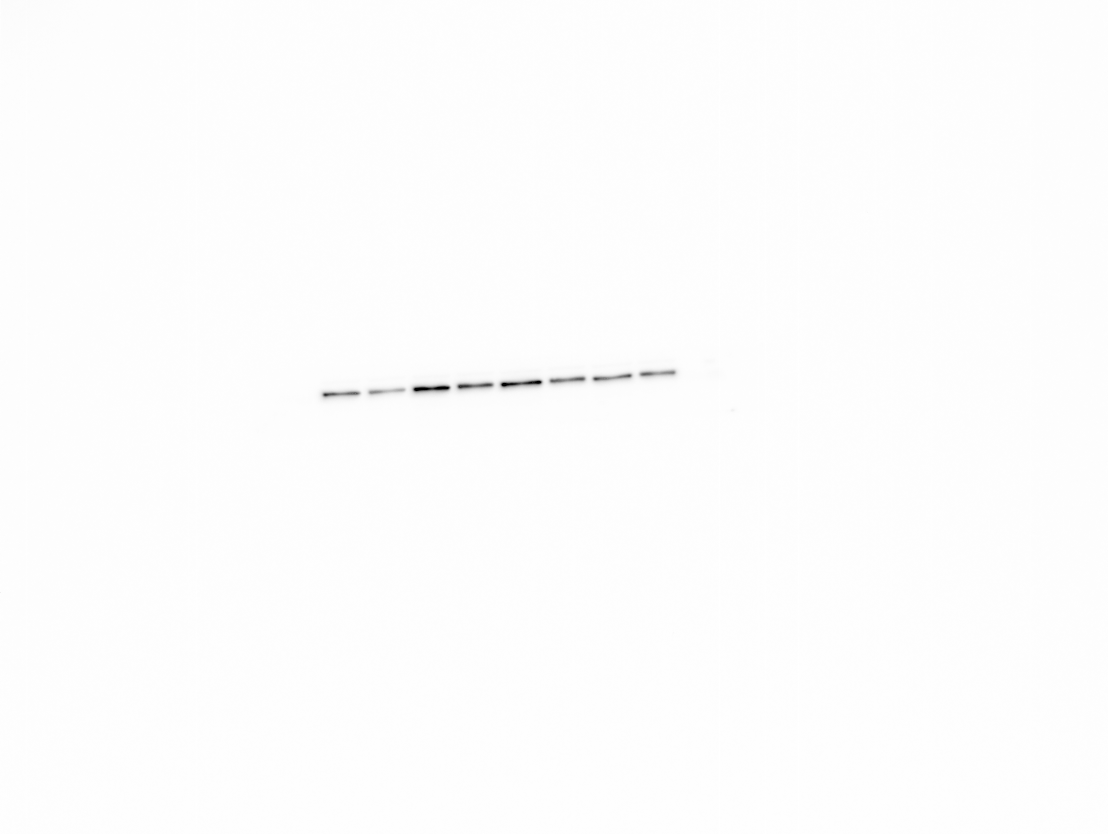

Supplement: Figure 1—source data 3. [file elife-84319-fig1-data3.zip › Figure 1ΓÇôSource Data 3/Figure 1E/His6/Replica 1_4.tif]

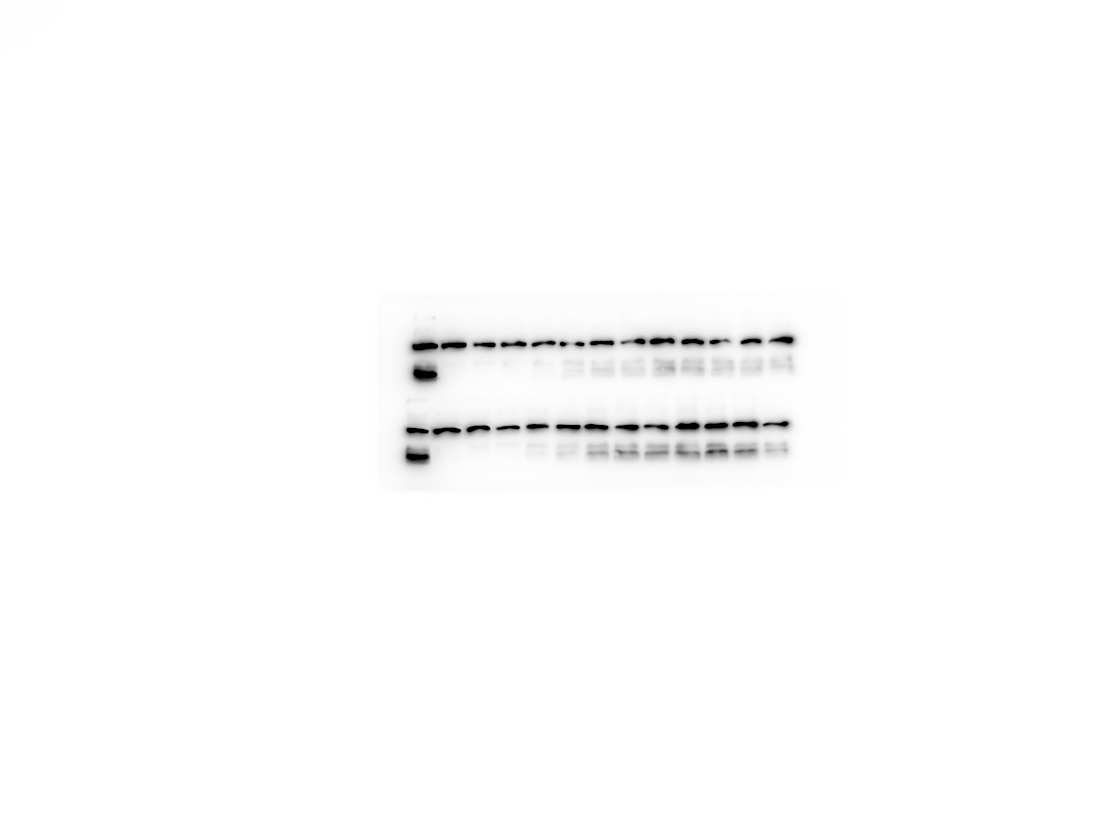

Supplement: Figure 1—source data 3. [file elife-84319-fig1-data3.zip › Figure 1ΓÇôSource Data 3/Figure 1C/Sch9-pThr737/Replica 4.tif]

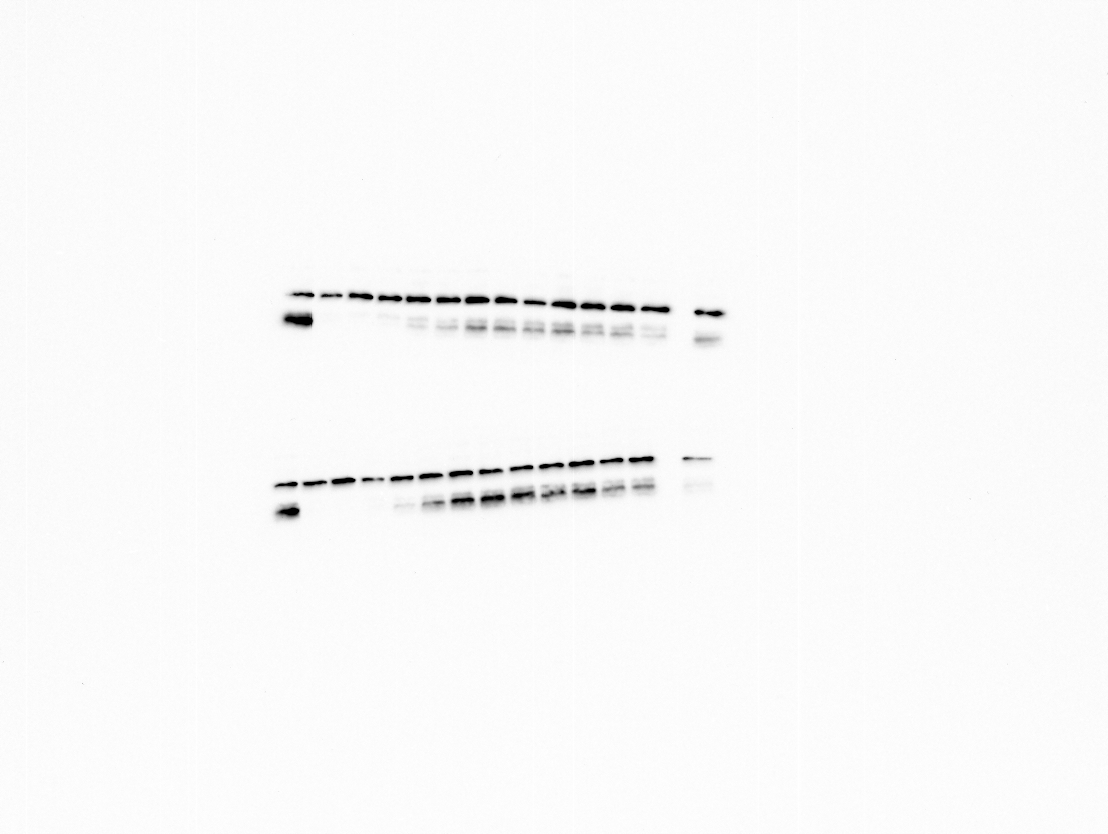

Supplement: Figure 1—source data 3. [file elife-84319-fig1-data3.zip › Figure 1ΓÇôSource Data 3/Figure 1C/Sch9-pThr737/Replica 3.jpg]

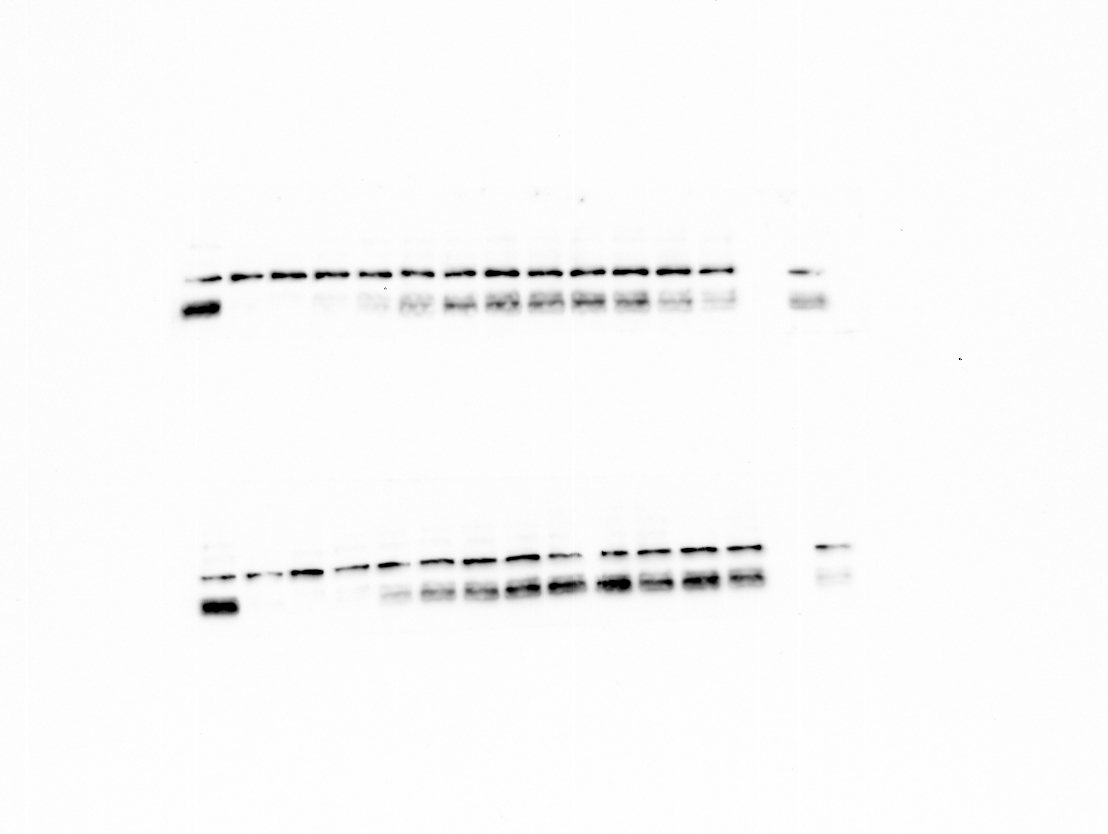

Supplement: Figure 1—source data 3. [file elife-84319-fig1-data3.zip › Figure 1ΓÇôSource Data 3/Figure 1C/Sch9-pThr737/Replica 2.jpg]

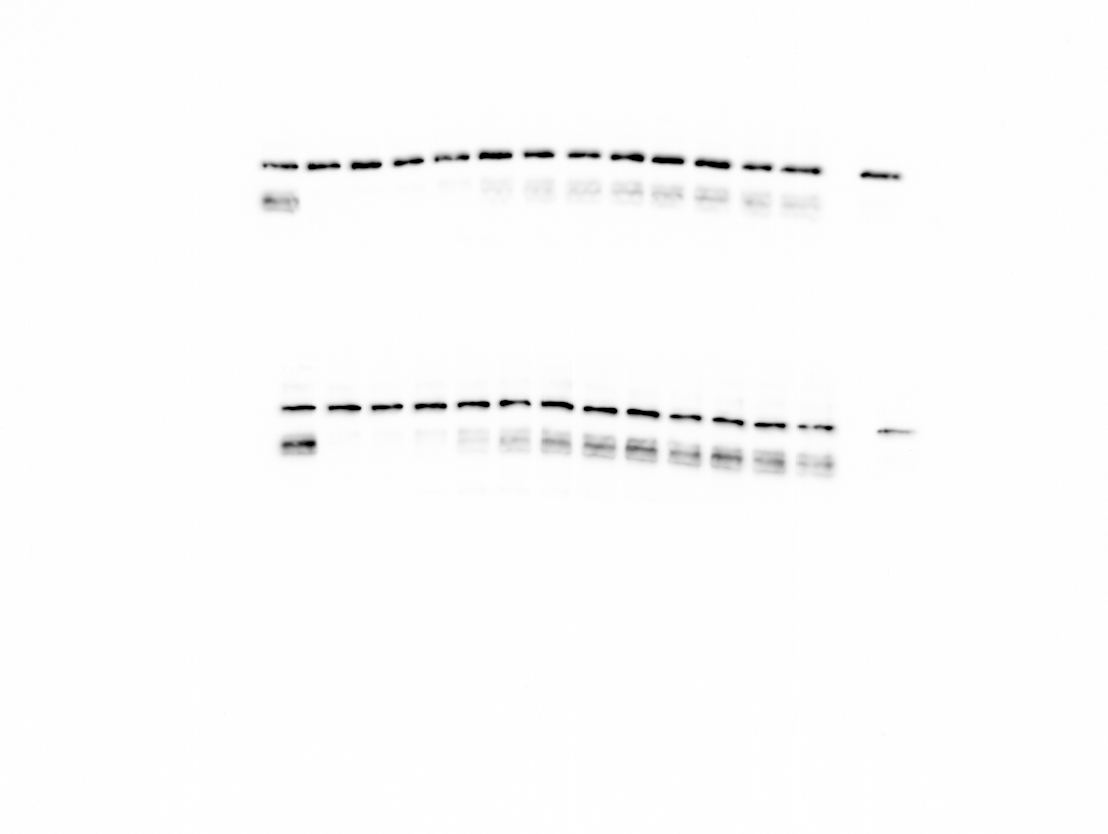

Supplement: Figure 1—source data 3. [file elife-84319-fig1-data3.zip › Figure 1ΓÇôSource Data 3/Figure 1C/Sch9-pThr737/Replica 1.jpg]

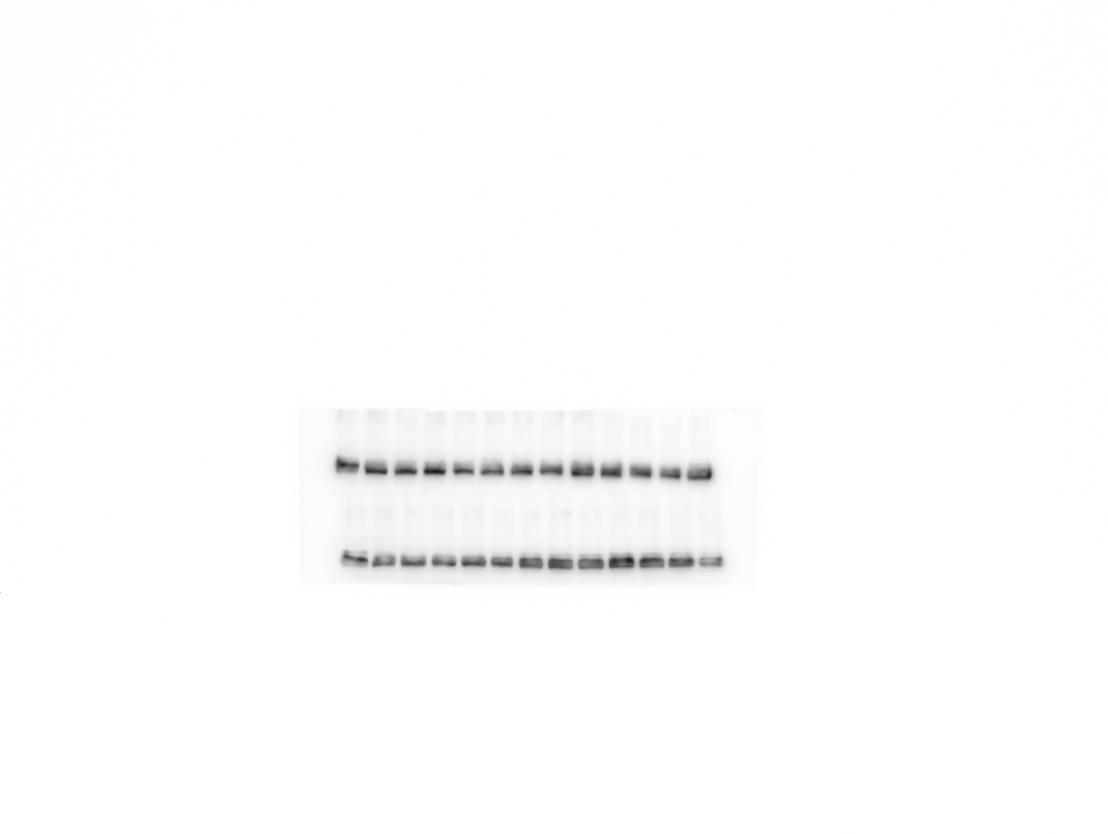

Supplement: Figure 1—source data 3. [file elife-84319-fig1-data3.zip › Figure 1ΓÇôSource Data 3/Figure 1C/Sch9/Replica 4.tif]

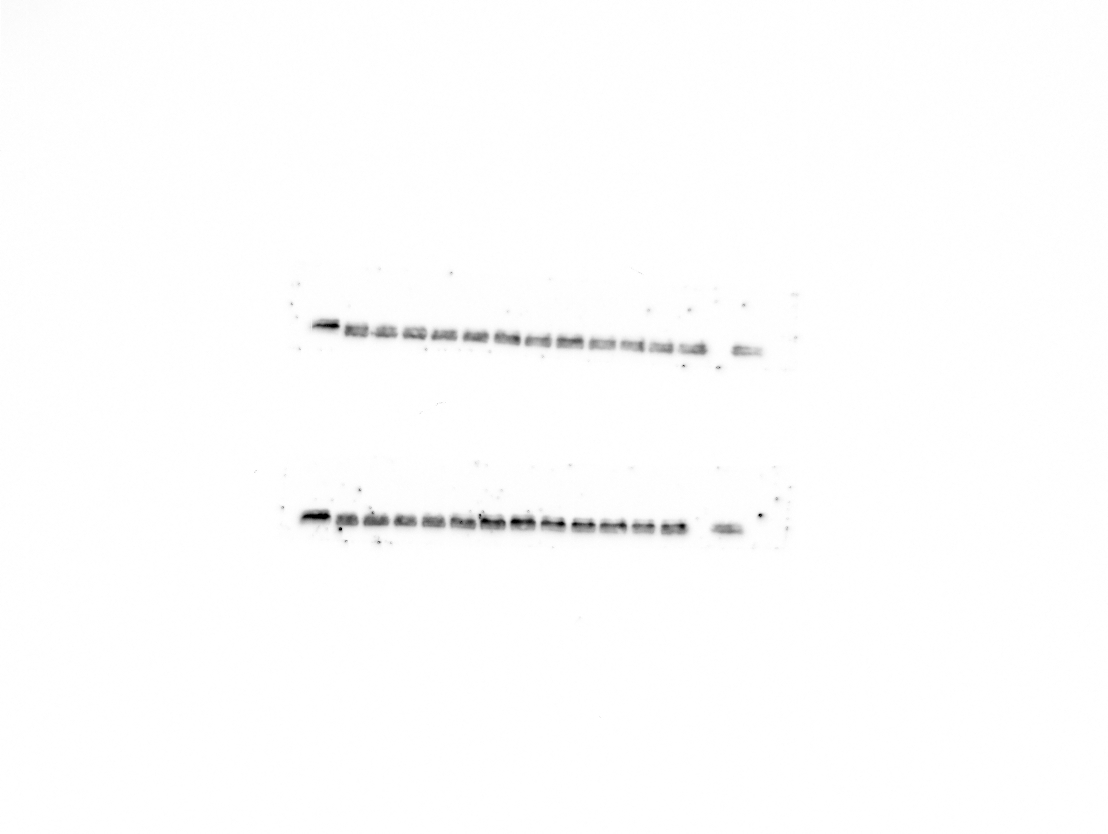

Supplement: Figure 1—source data 3. [file elife-84319-fig1-data3.zip › Figure 1ΓÇôSource Data 3/Figure 1C/Sch9/Replica 3.jpg]

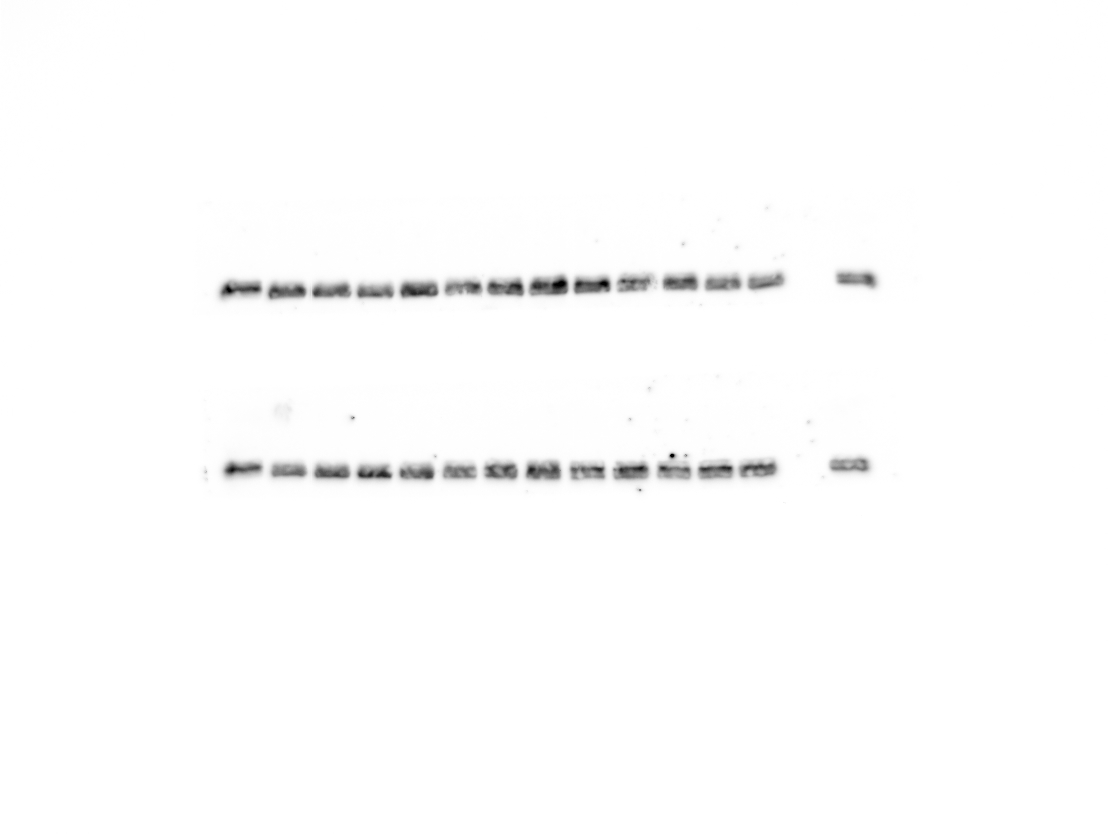

Supplement: Figure 1—source data 3. [file elife-84319-fig1-data3.zip › Figure 1ΓÇôSource Data 3/Figure 1C/Sch9/Replica 2.jpg]

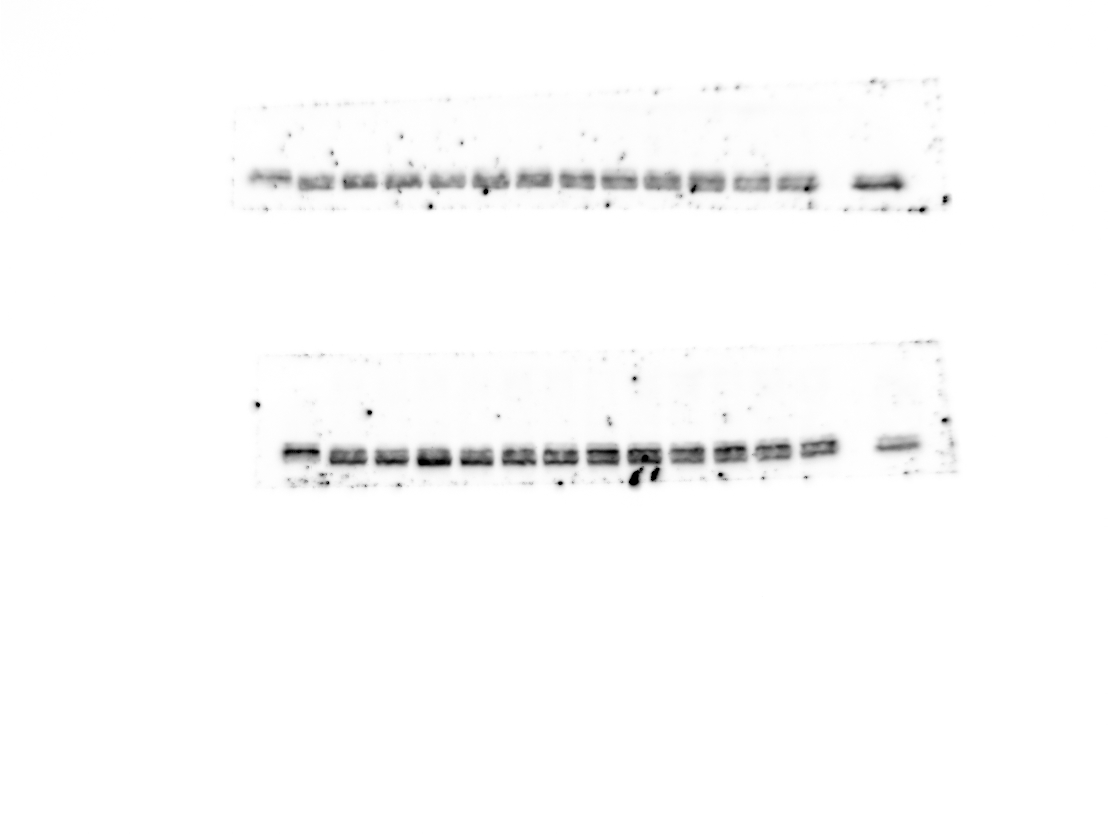

Supplement: Figure 1—source data 3. [file elife-84319-fig1-data3.zip › Figure 1ΓÇôSource Data 3/Figure 1C/Sch9/Replica 1.jpg]

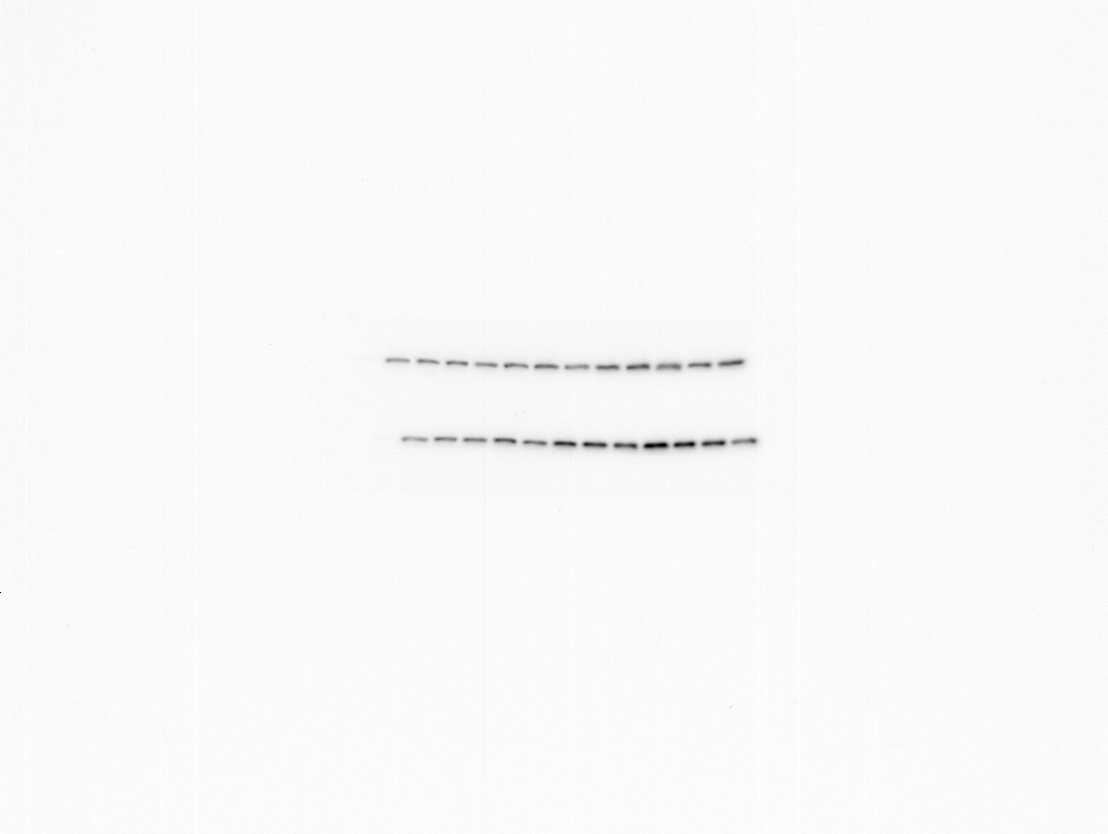

Supplement: Figure 1—source data 3. [file elife-84319-fig1-data3.zip › Figure 1ΓÇôSource Data 3/Figure 1C/Snf1-pThr210/Replica 4.tif]

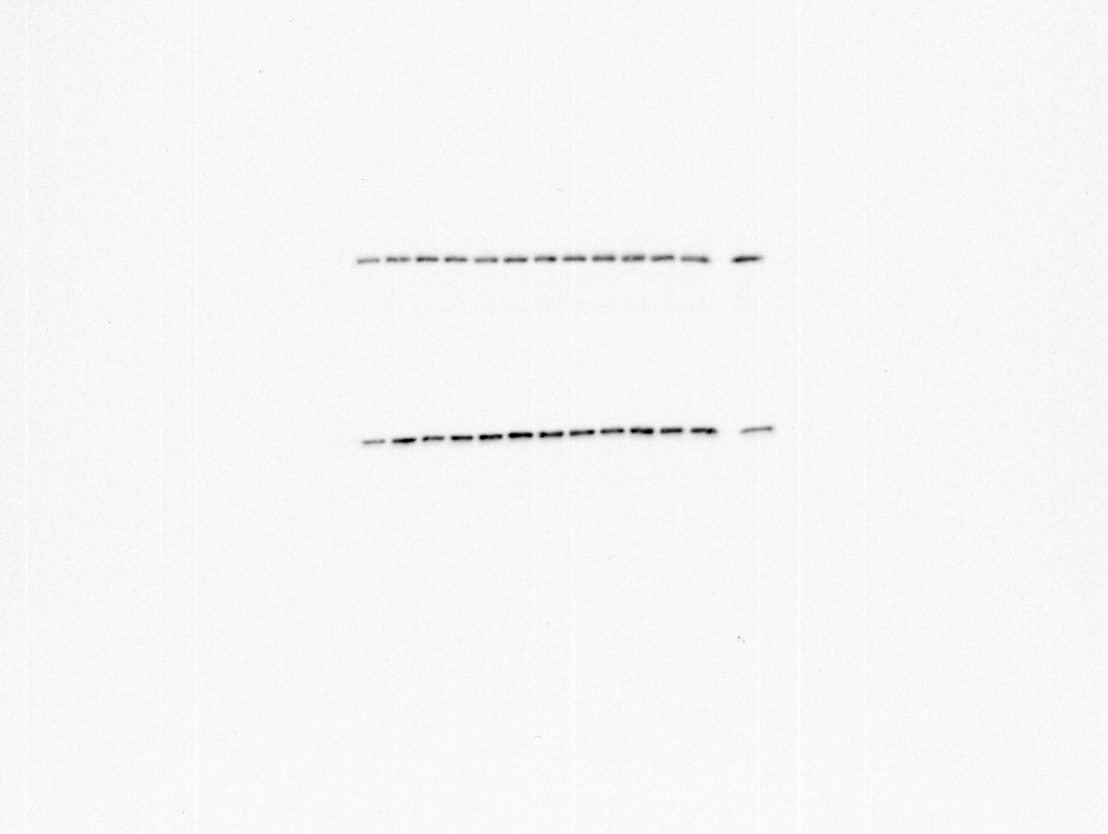

Supplement: Figure 1—source data 3. [file elife-84319-fig1-data3.zip › Figure 1ΓÇôSource Data 3/Figure 1C/Snf1-pThr210/Replica 3.jpg]

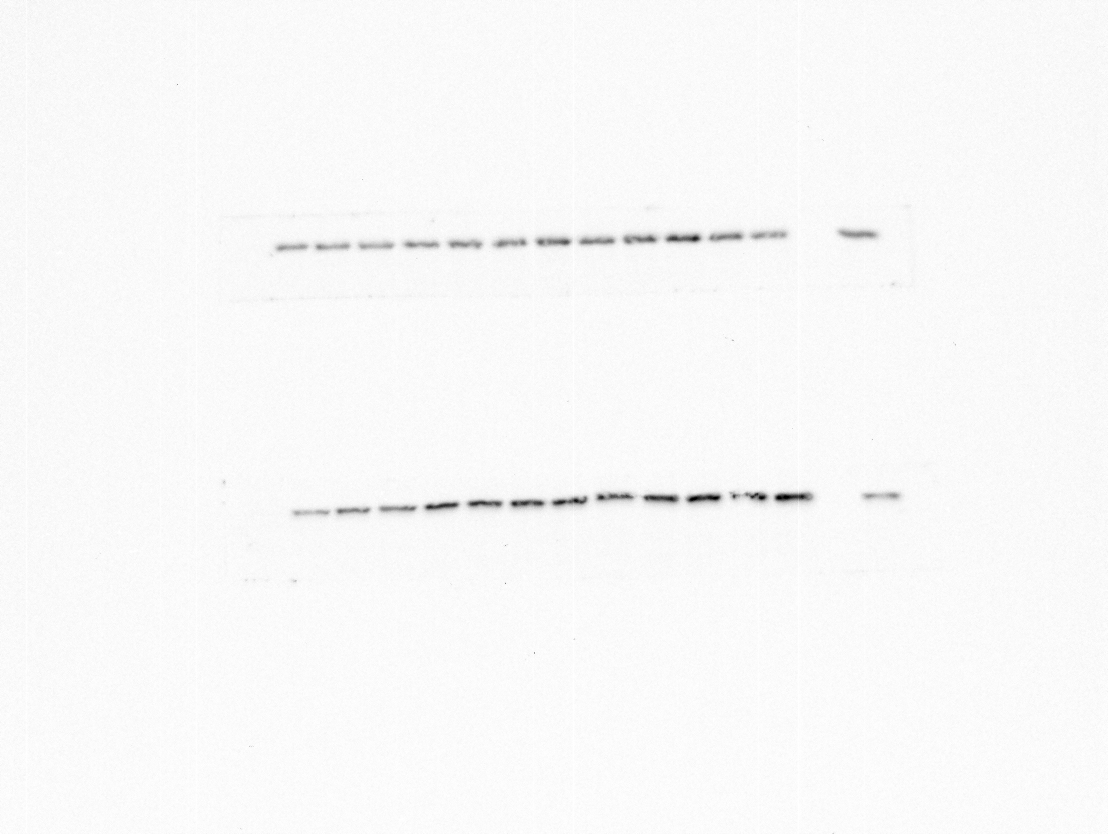

Supplement: Figure 1—source data 3. [file elife-84319-fig1-data3.zip › Figure 1ΓÇôSource Data 3/Figure 1C/Snf1-pThr210/Replica 2.jpg]

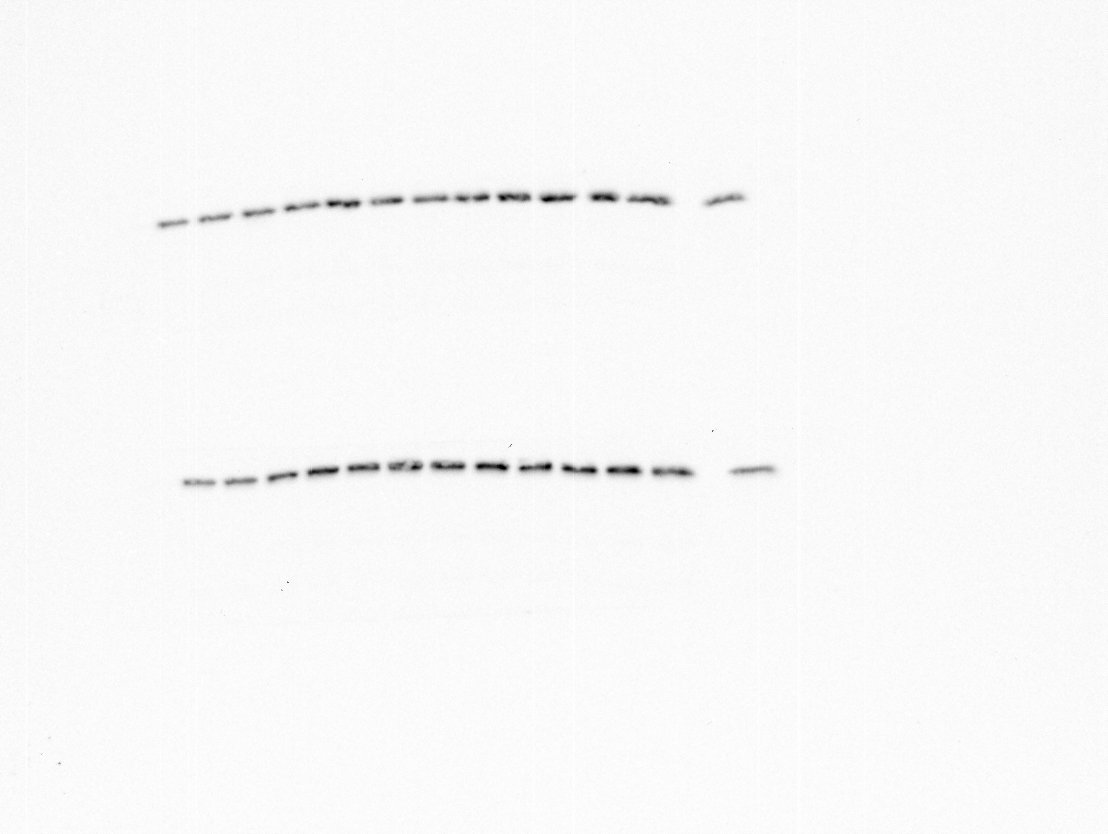

Supplement: Figure 1—source data 3. [file elife-84319-fig1-data3.zip › Figure 1ΓÇôSource Data 3/Figure 1C/Snf1-pThr210/Replica 1.jpg]

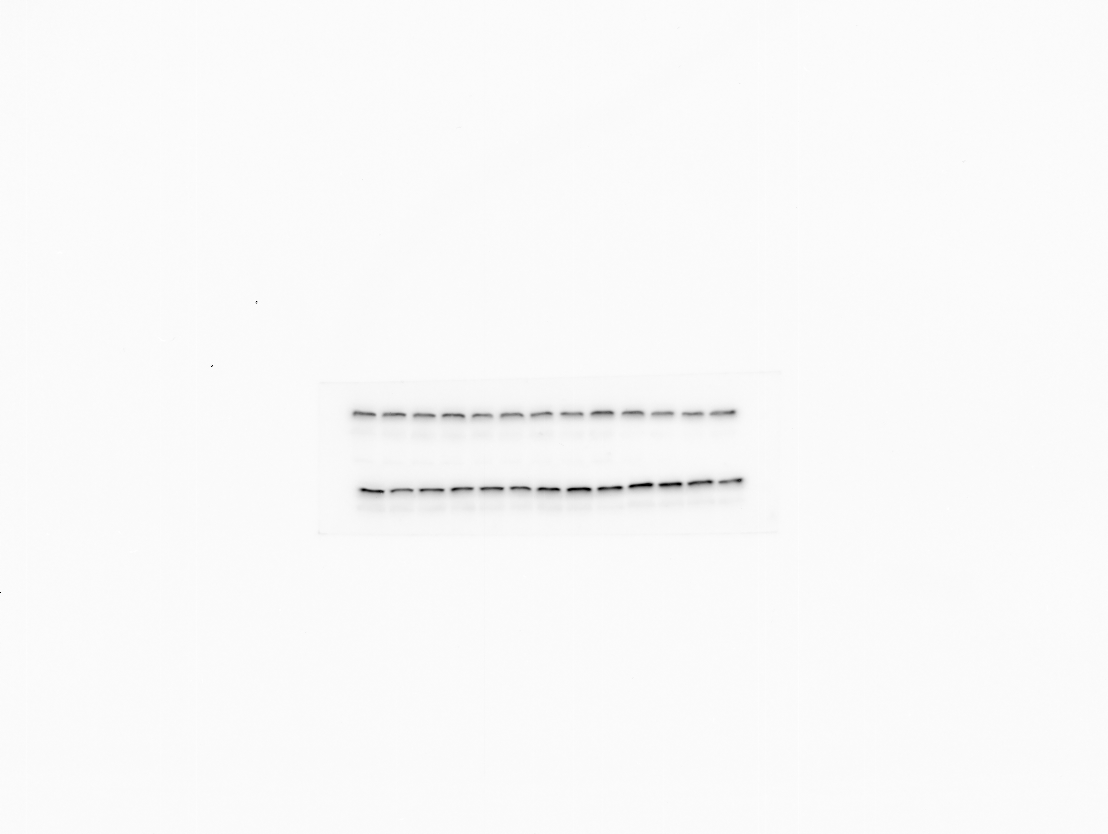

Supplement: Figure 1—source data 3. [file elife-84319-fig1-data3.zip › Figure 1ΓÇôSource Data 3/Figure 1C/His6/Replica 4.tif]

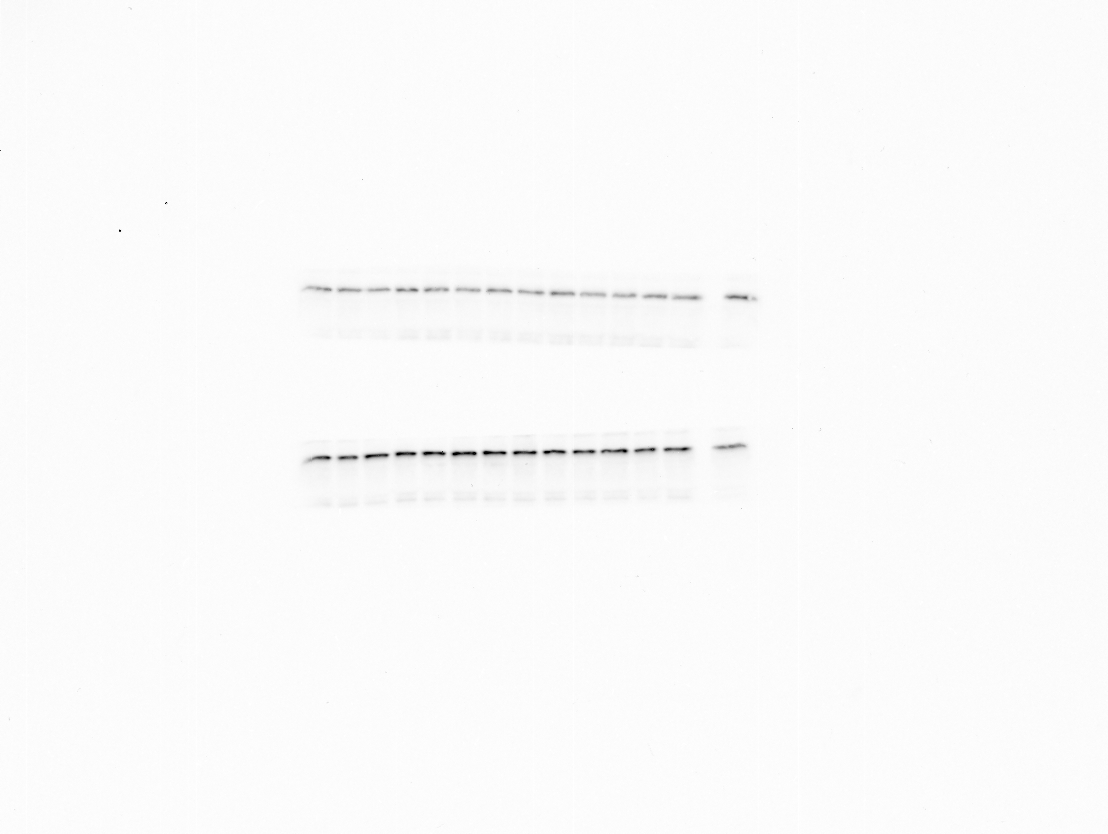

Supplement: Figure 1—source data 3. [file elife-84319-fig1-data3.zip › Figure 1ΓÇôSource Data 3/Figure 1C/His6/Replica 3.jpg]

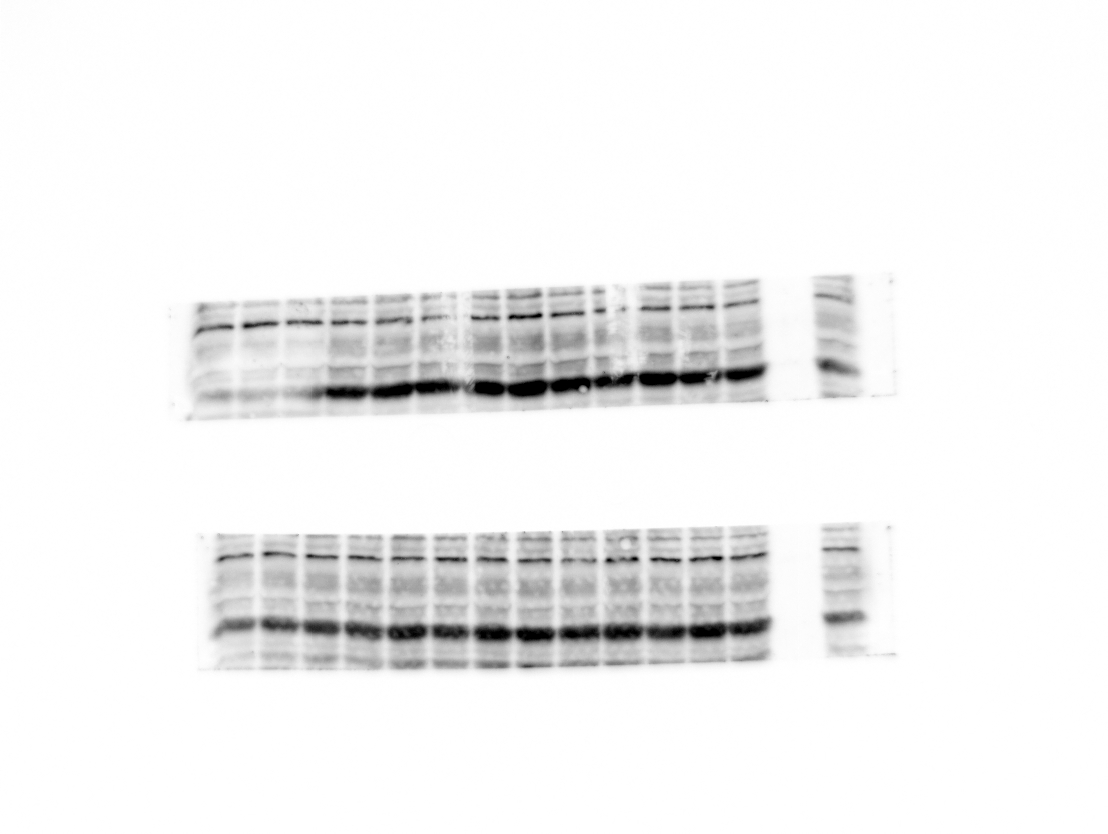

Supplement: Figure 1—source data 3. [file elife-84319-fig1-data3.zip › Figure 1ΓÇôSource Data 3/Figure 1C/His6/Replica 2.jpg]

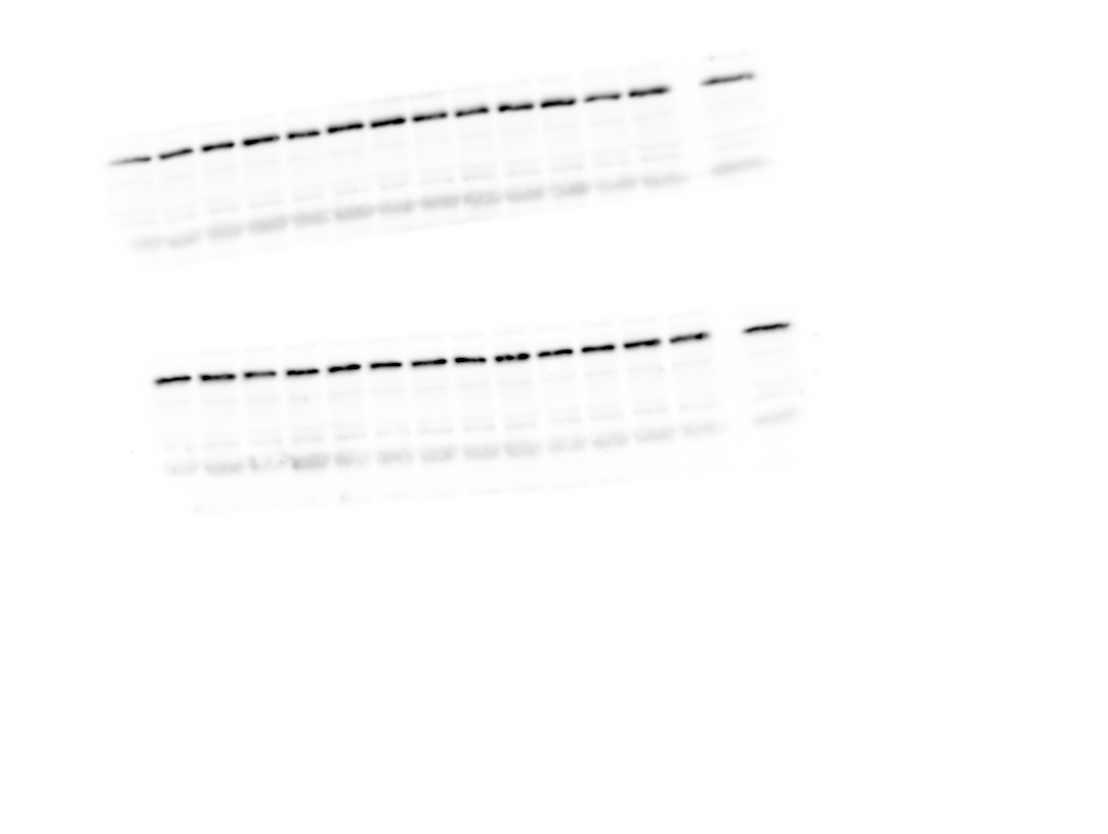

Supplement: Figure 1—source data 3. [file elife-84319-fig1-data3.zip › Figure 1ΓÇôSource Data 3/Figure 1C/His6/Replica 1.jpg]

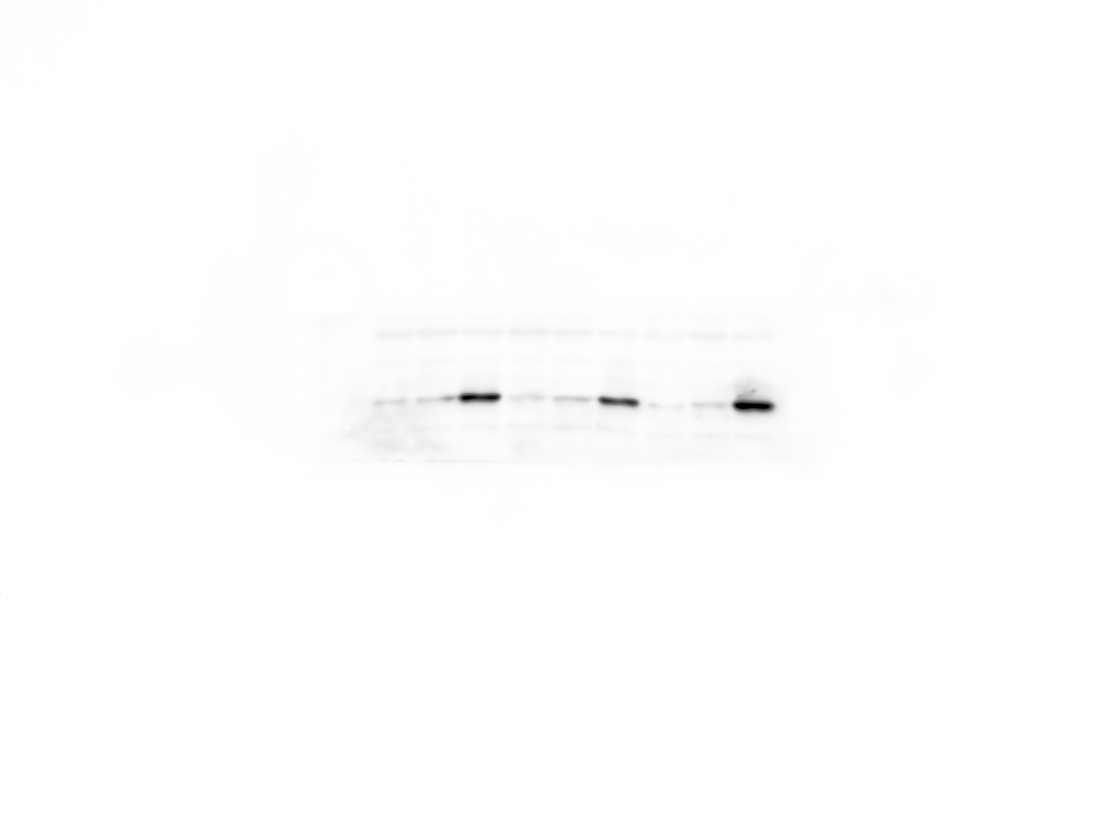

Supplement: Figure 1—figure supplement 1—source data 3. [file elife-84319-fig1-figsupp1-data3.zip › Figure 1ΓÇôSupplementary figure 1-Source Data 2/Supplementary figure 1B/Acc1-pSer79/Replica 1_2_3.tif]

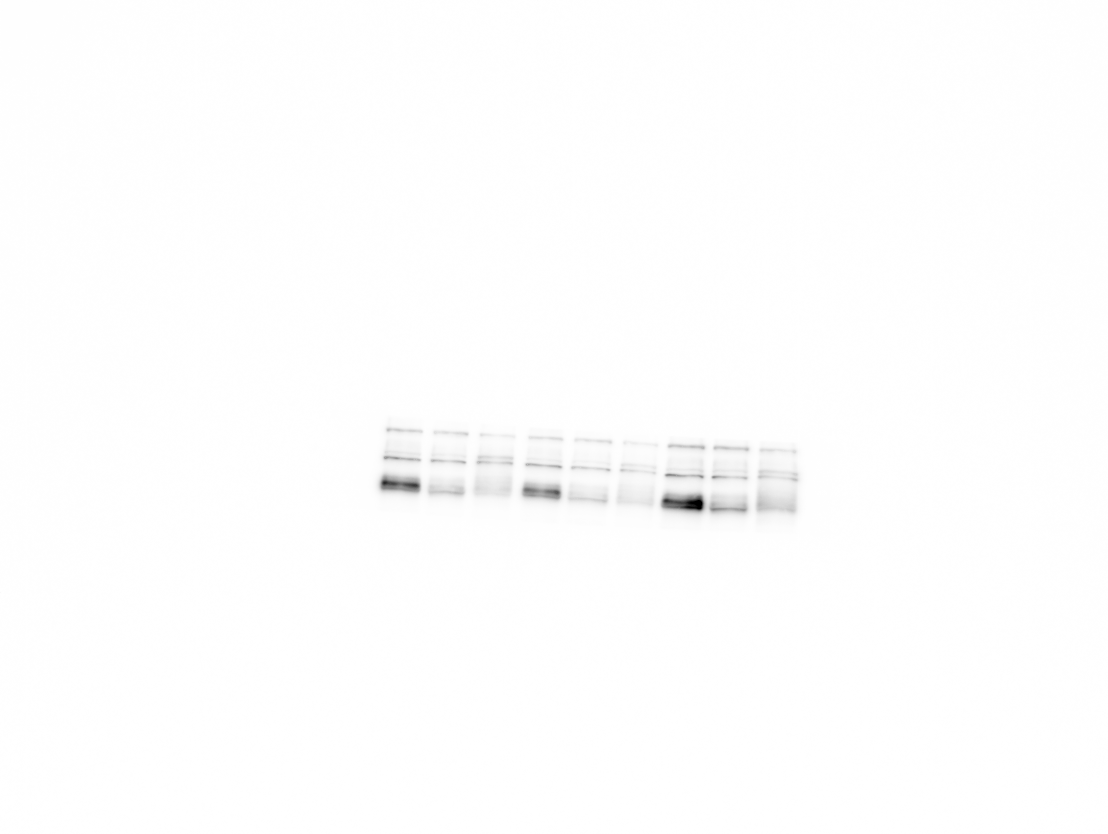

Supplement: Figure 1—figure supplement 1—source data 3. [file elife-84319-fig1-figsupp1-data3.zip › Figure 1ΓÇôSupplementary figure 1-Source Data 2/Supplementary figure 1B/Sch9-pThr737/Replica 1_2_3.tif]

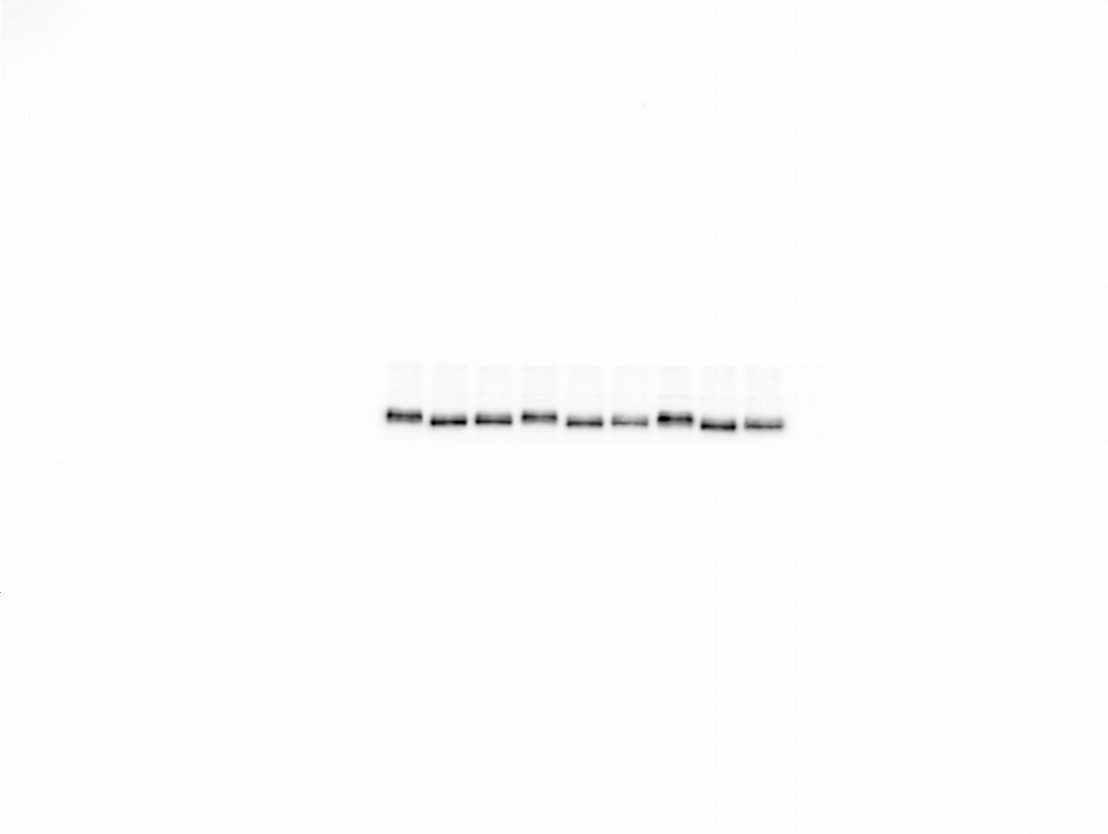

Supplement: Figure 1—figure supplement 1—source data 3. [file elife-84319-fig1-figsupp1-data3.zip › Figure 1ΓÇôSupplementary figure 1-Source Data 2/Supplementary figure 1B/Sch9/Replica 1_2_3.tif]

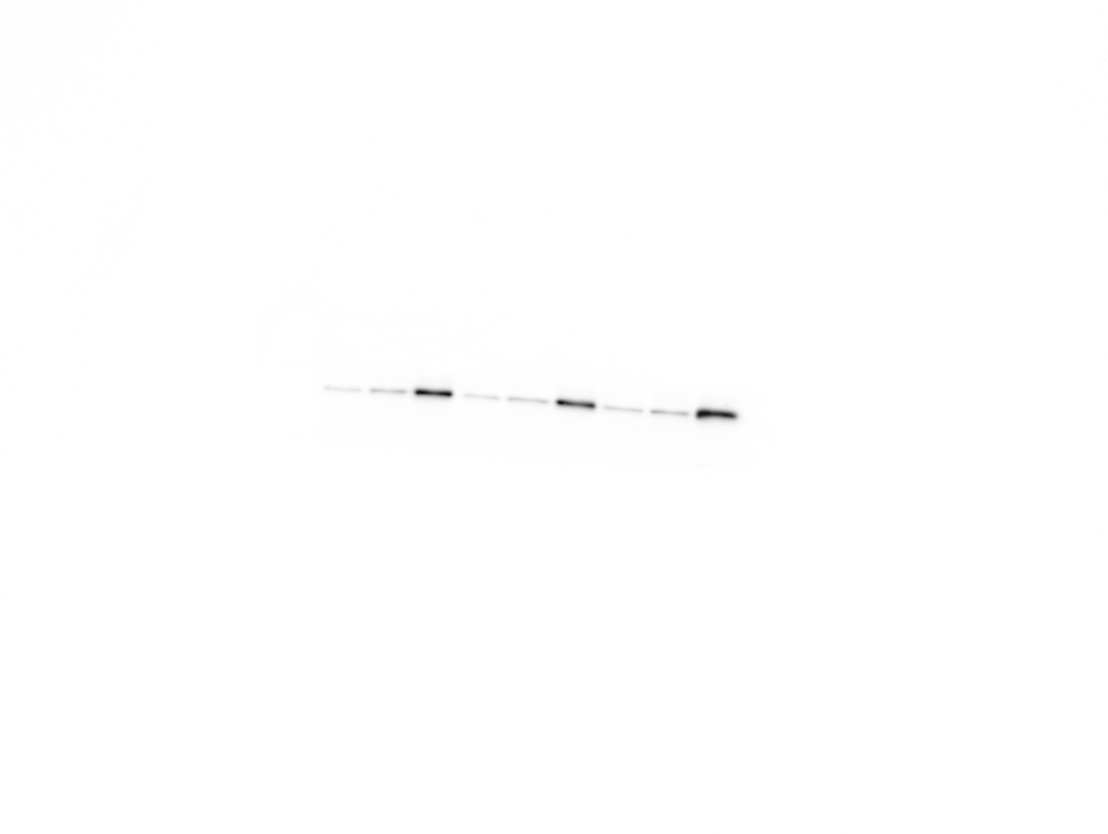

Supplement: Figure 1—figure supplement 1—source data 3. [file elife-84319-fig1-figsupp1-data3.zip › Figure 1ΓÇôSupplementary figure 1-Source Data 2/Supplementary figure 1B/Snf1-pThr210/Replica 1_2_3.tif]

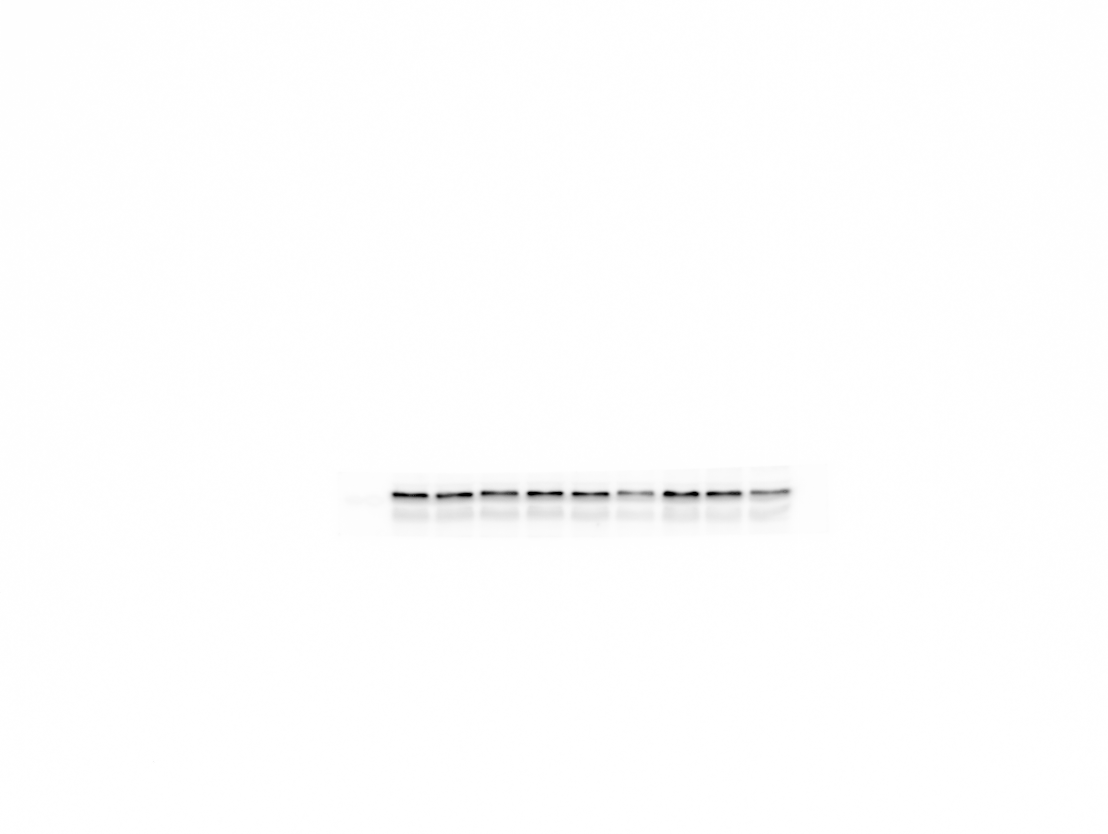

Supplement: Figure 1—figure supplement 1—source data 3. [file elife-84319-fig1-figsupp1-data3.zip › Figure 1ΓÇôSupplementary figure 1-Source Data 2/Supplementary figure 1B/His6/Replica 1_2_3.tif]

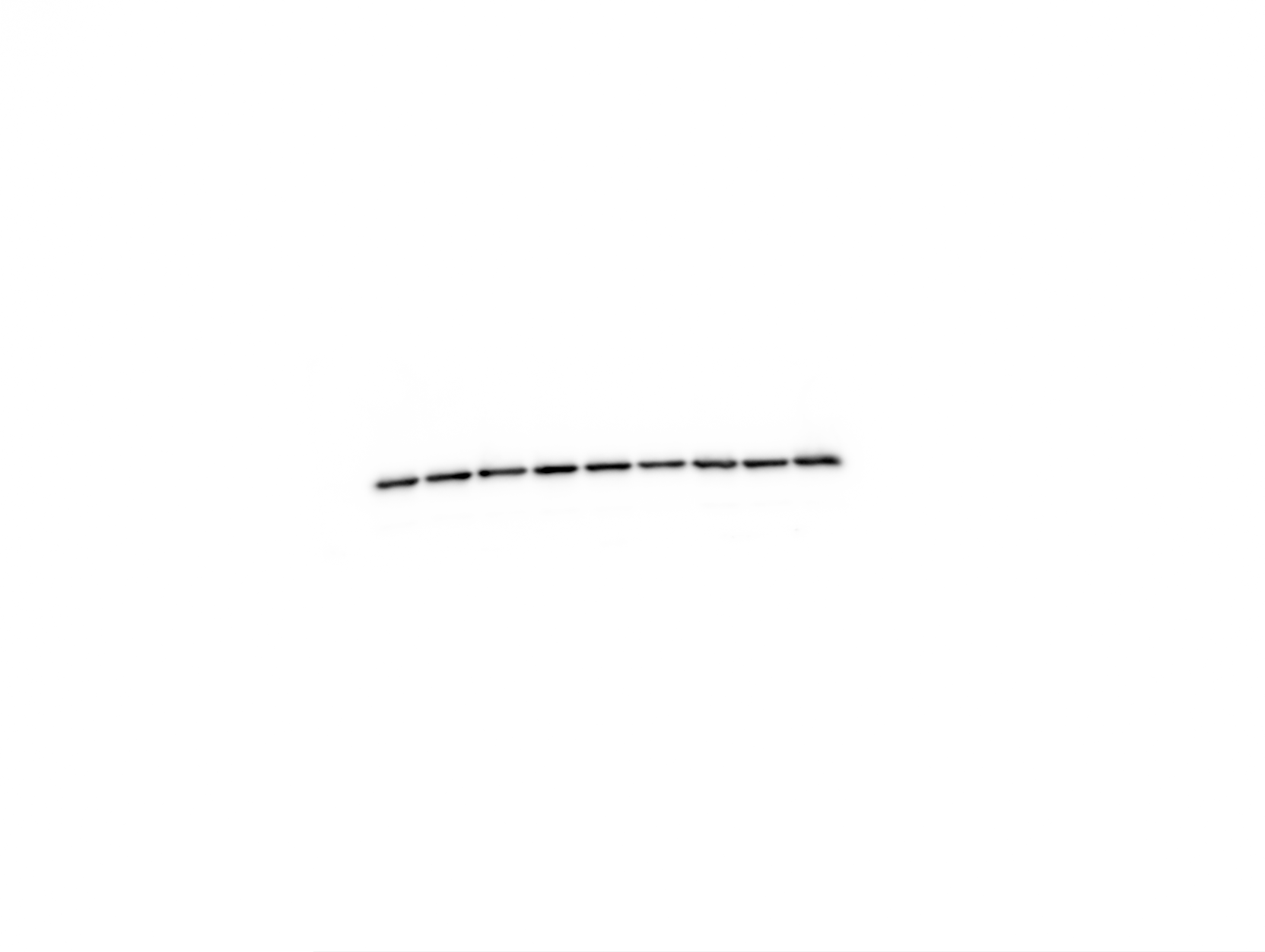

Supplement: Figure 1—figure supplement 1—source data 3. [file elife-84319-fig1-figsupp1-data3.zip › Figure 1ΓÇôSupplementary figure 1-Source Data 2/Supplementary figure 1B/GFP/Replica 1_2_3.tif]

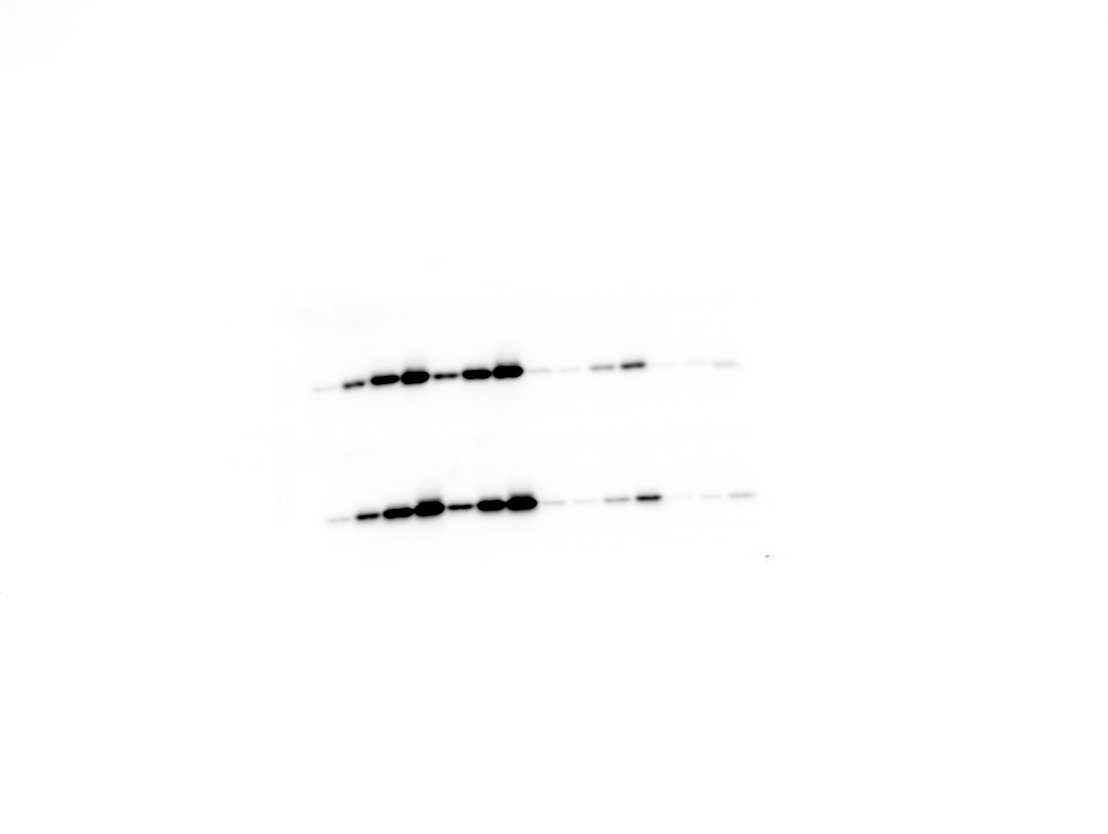

Supplement: Figure 1—figure supplement 1—source data 3. [file elife-84319-fig1-figsupp1-data3.zip › Figure 1ΓÇôSupplementary figure 1-Source Data 2/Supplementary figure 1A/Acc1-pSer79/Replica 2_3.tif]

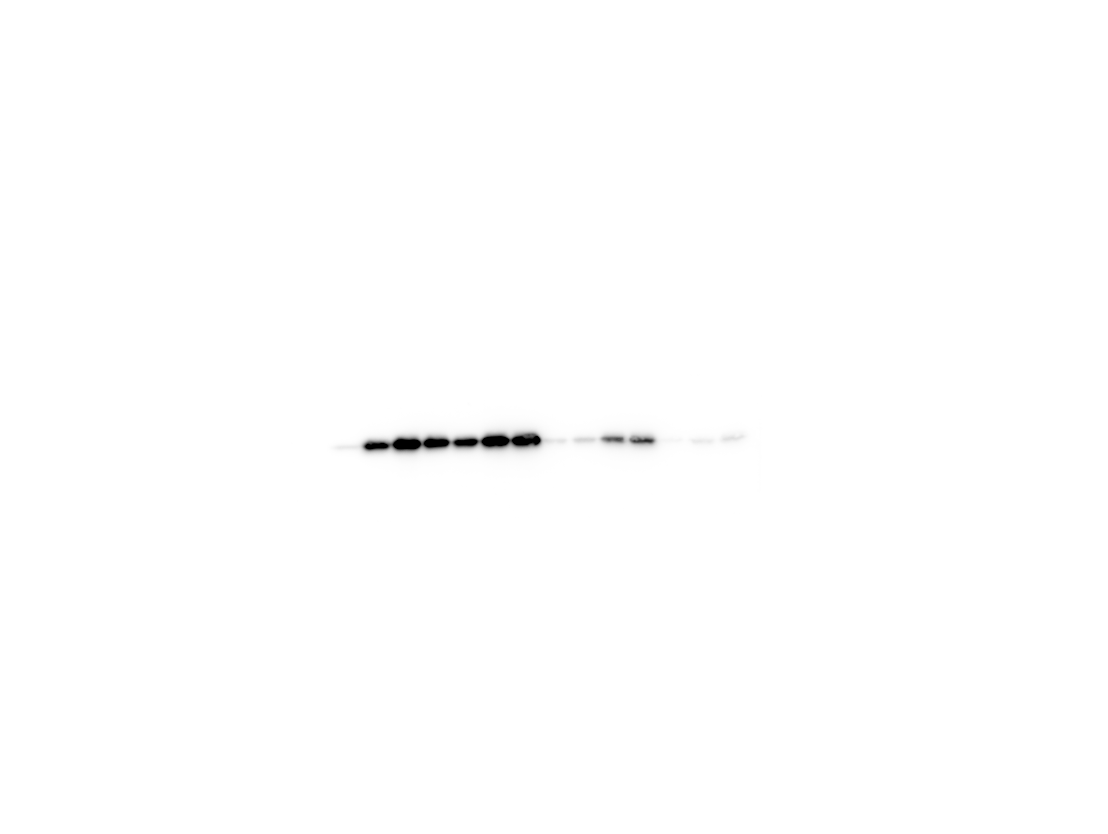

Supplement: Figure 1—figure supplement 1—source data 3. [file elife-84319-fig1-figsupp1-data3.zip › Figure 1ΓÇôSupplementary figure 1-Source Data 2/Supplementary figure 1A/Acc1-pSer79/Replica 1.bmp]

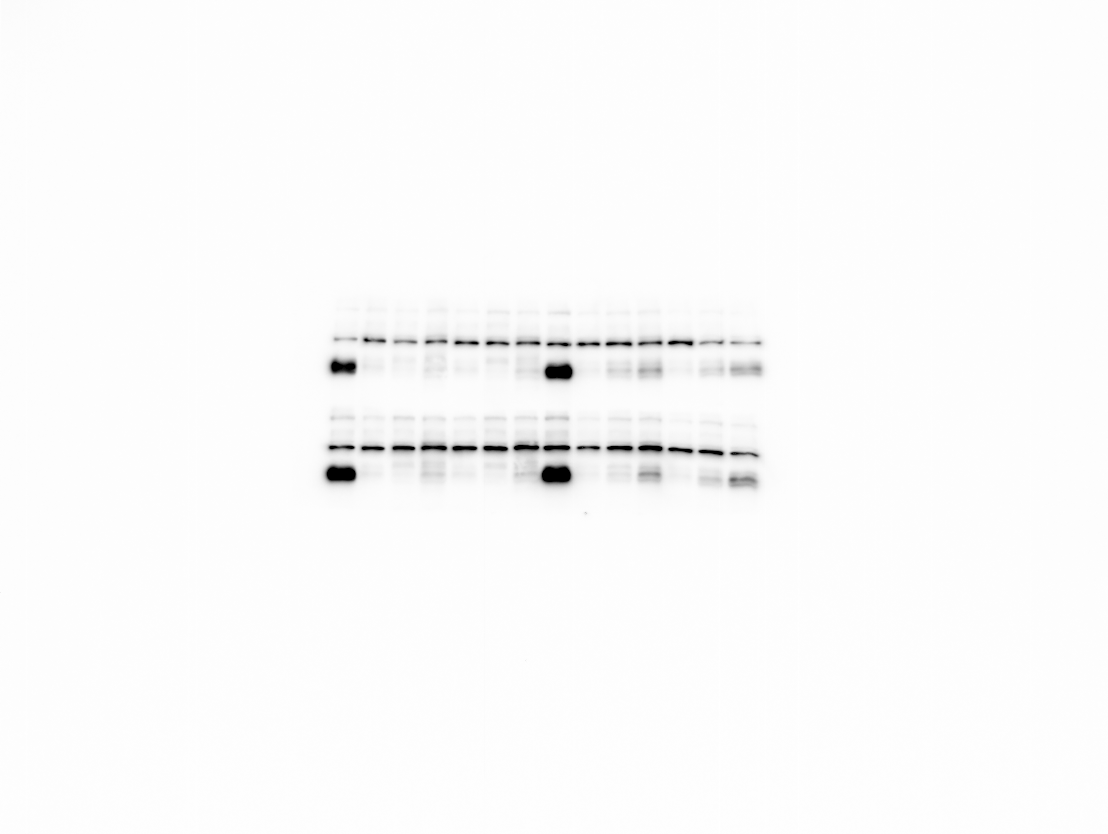

Supplement: Figure 1—figure supplement 1—source data 3. [file elife-84319-fig1-figsupp1-data3.zip › Figure 1ΓÇôSupplementary figure 1-Source Data 2/Supplementary figure 1A/Sch9-pThr737/Replica 2_3.tif]

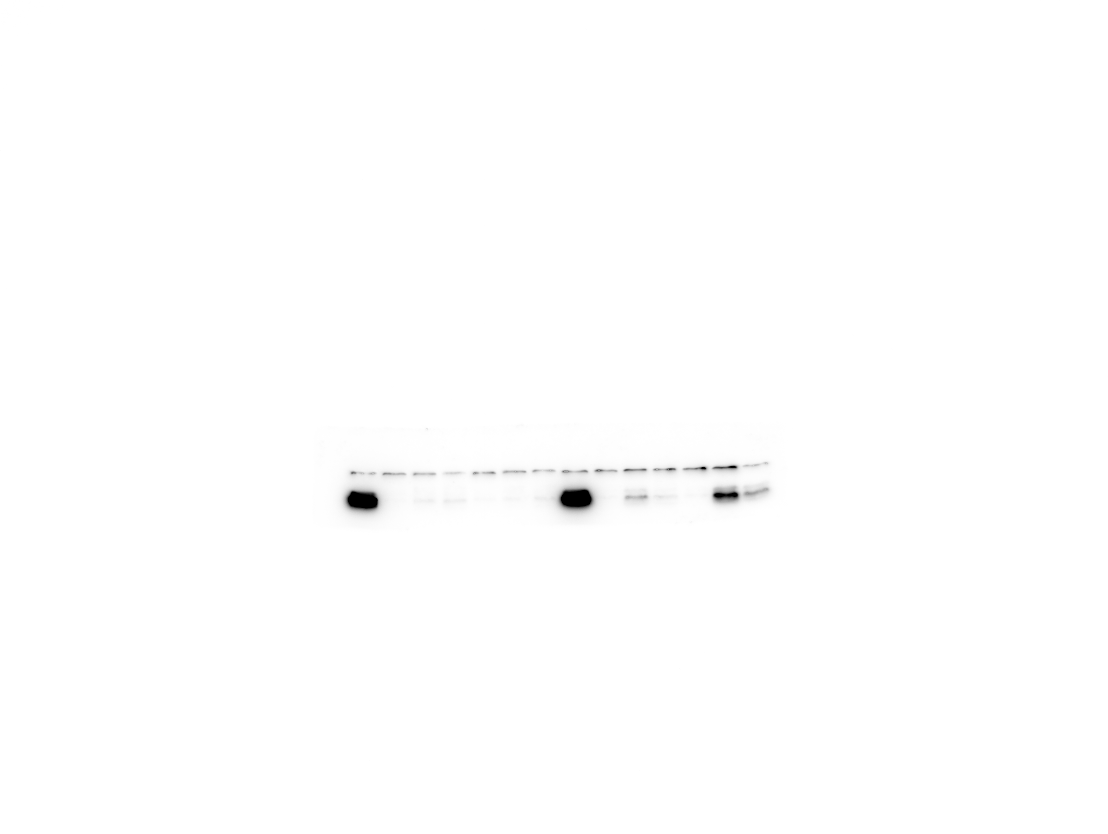

Supplement: Figure 1—figure supplement 1—source data 3. [file elife-84319-fig1-figsupp1-data3.zip › Figure 1ΓÇôSupplementary figure 1-Source Data 2/Supplementary figure 1A/Sch9-pThr737/Replica 1.bmp]

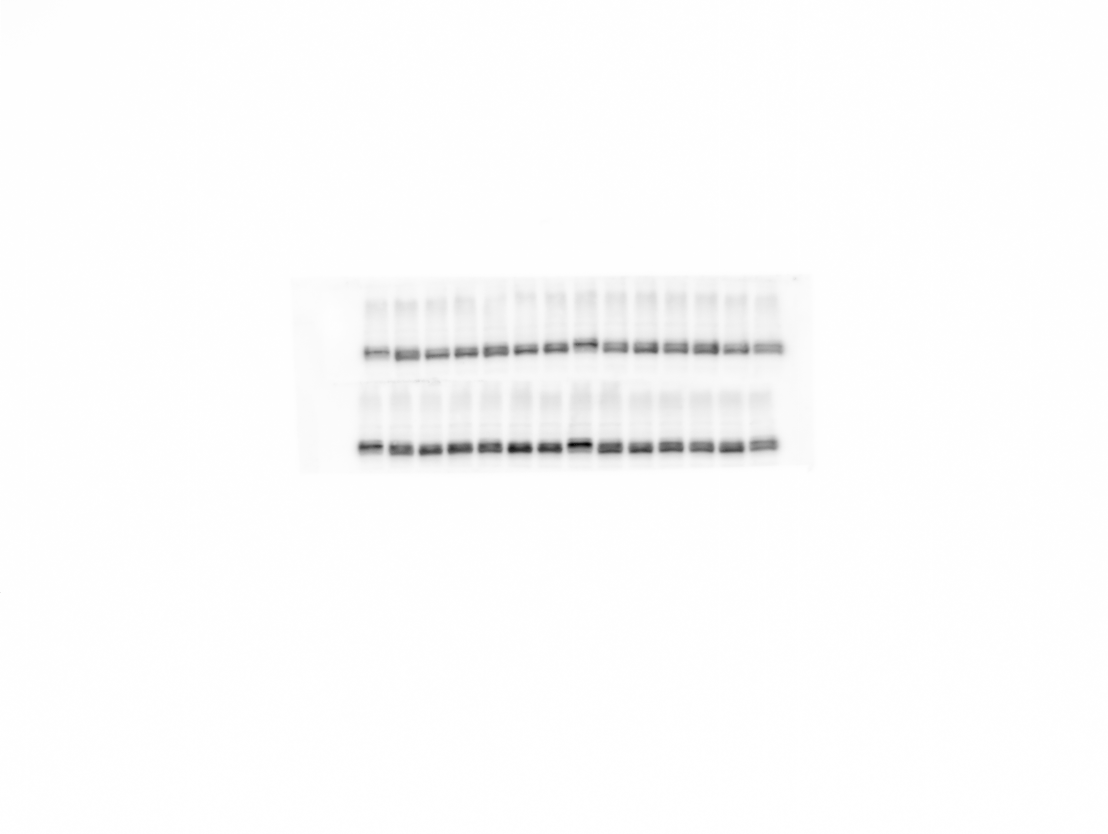

Supplement: Figure 1—figure supplement 1—source data 3. [file elife-84319-fig1-figsupp1-data3.zip › Figure 1ΓÇôSupplementary figure 1-Source Data 2/Supplementary figure 1A/Sch9/Replica 2_3.tif]

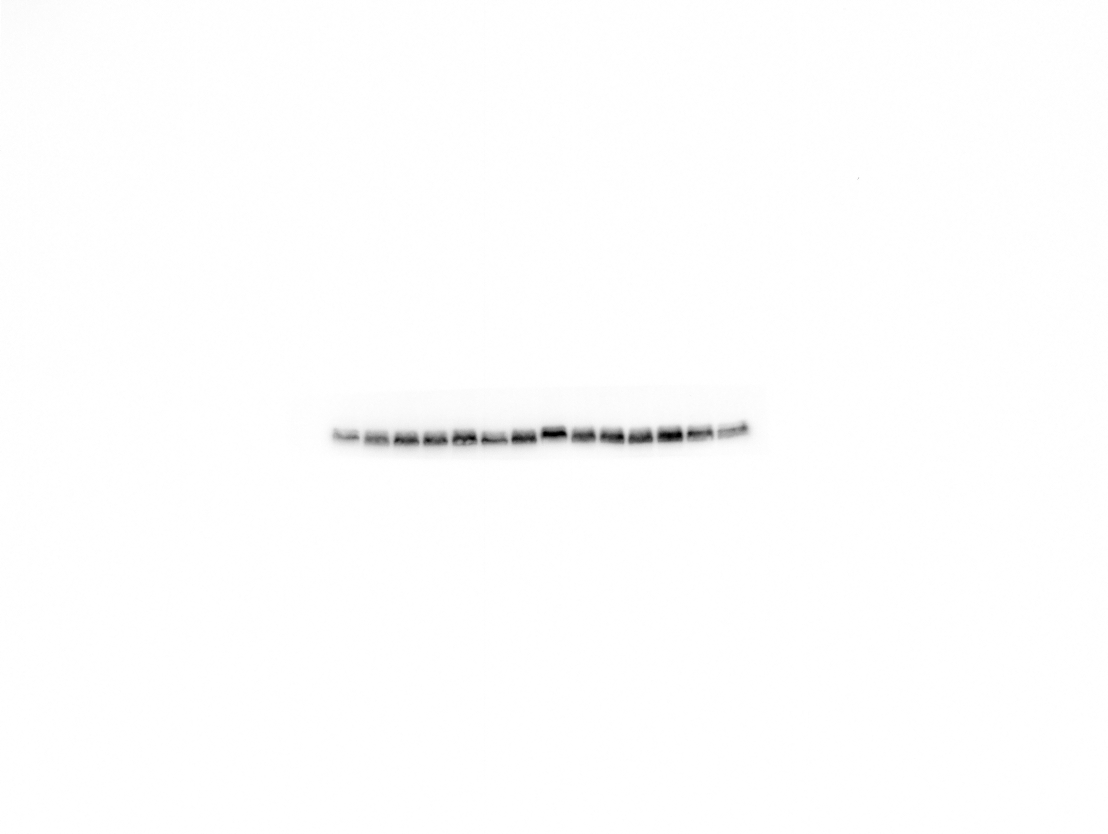

Supplement: Figure 1—figure supplement 1—source data 3. [file elife-84319-fig1-figsupp1-data3.zip › Figure 1ΓÇôSupplementary figure 1-Source Data 2/Supplementary figure 1A/Sch9/Replica 1.bmp]

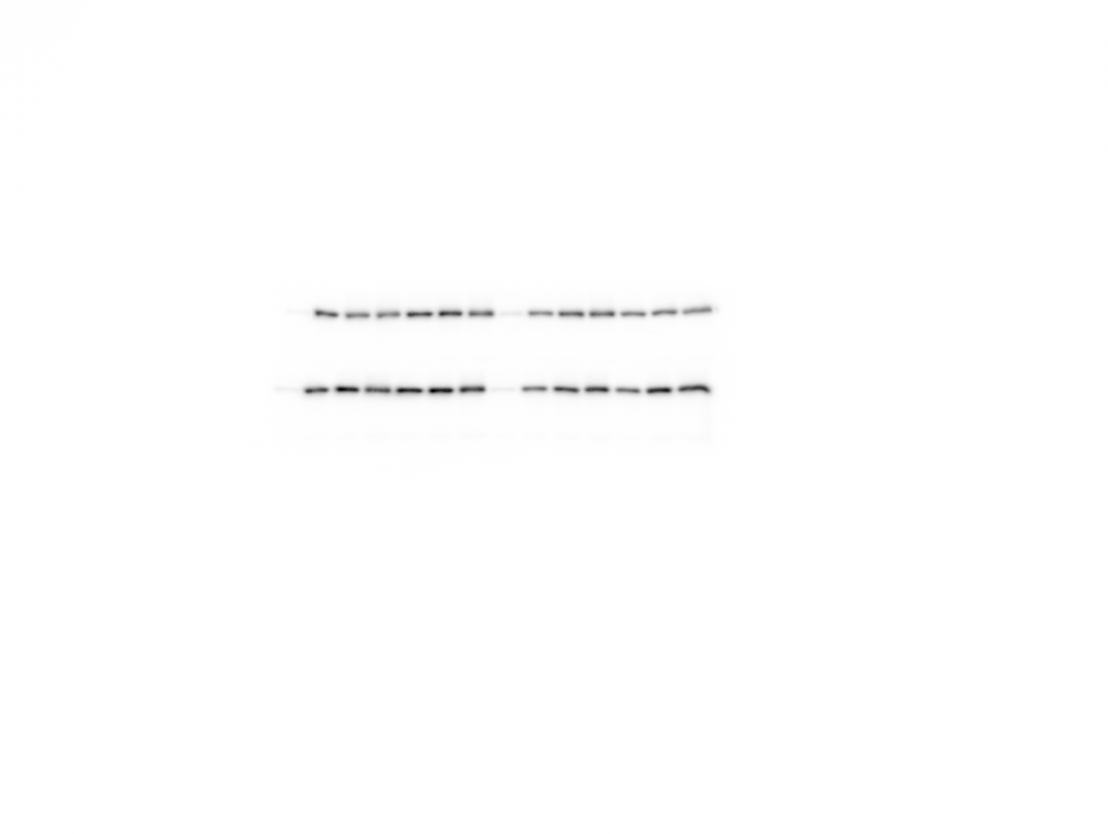

Supplement: Figure 1—figure supplement 1—source data 3. [file elife-84319-fig1-figsupp1-data3.zip › Figure 1ΓÇôSupplementary figure 1-Source Data 2/Supplementary figure 1A/Snf1-pThr210/Replica 2_3.tif]

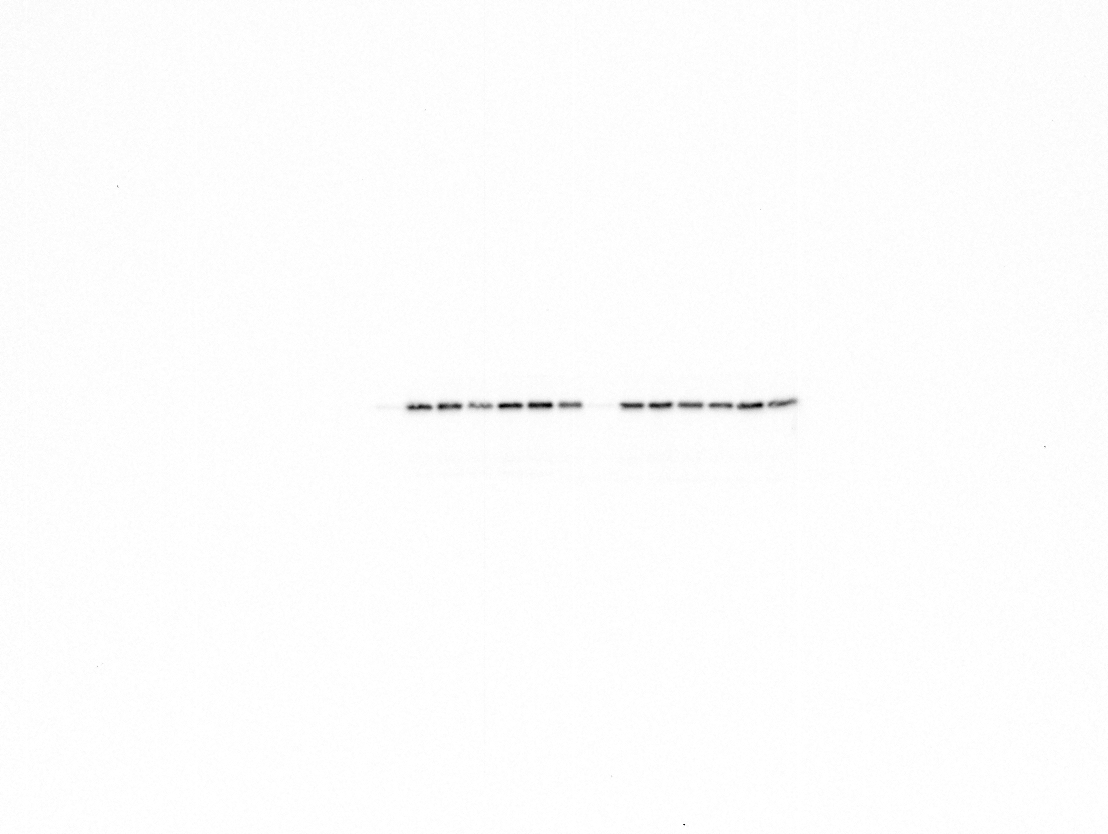

Supplement: Figure 1—figure supplement 1—source data 3. [file elife-84319-fig1-figsupp1-data3.zip › Figure 1ΓÇôSupplementary figure 1-Source Data 2/Supplementary figure 1A/Snf1-pThr210/Replica 1.bmp]

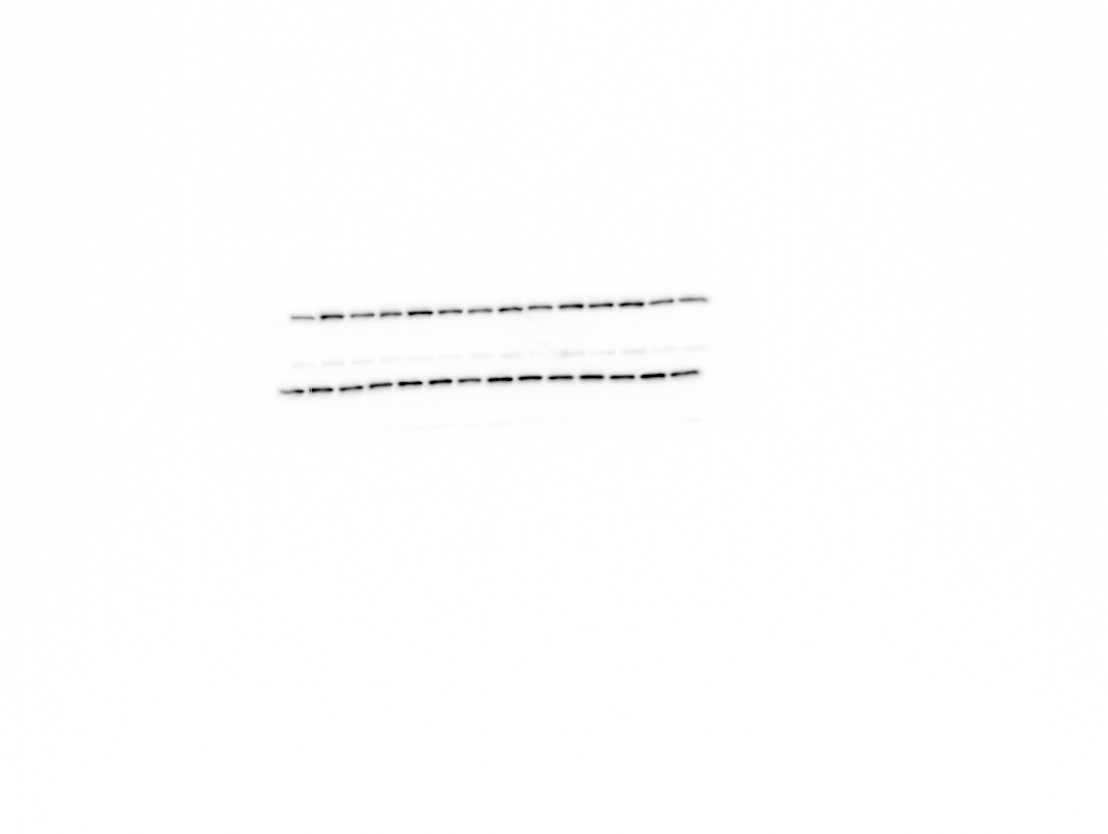

Supplement: Figure 1—figure supplement 1—source data 3. [file elife-84319-fig1-figsupp1-data3.zip › Figure 1ΓÇôSupplementary figure 1-Source Data 2/Supplementary figure 1A/His6/Replica 2_3.tif]

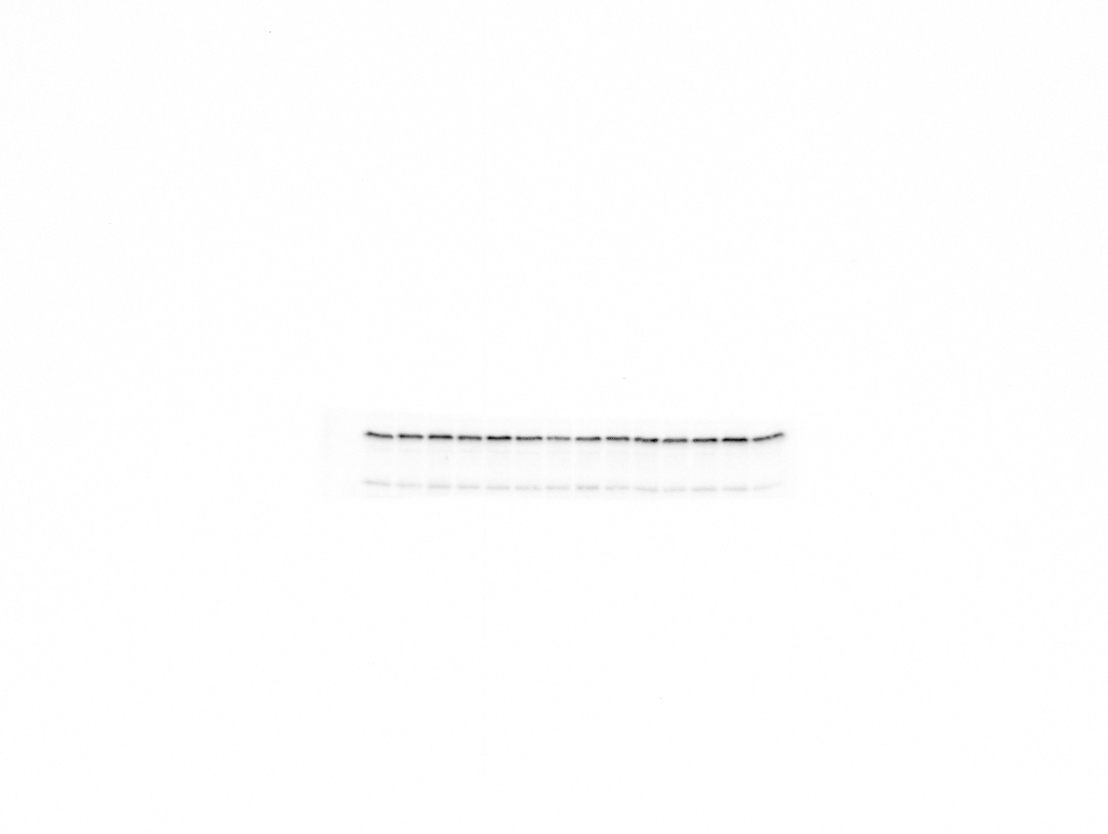

Supplement: Figure 1—figure supplement 1—source data 3. [file elife-84319-fig1-figsupp1-data3.zip › Figure 1ΓÇôSupplementary figure 1-Source Data 2/Supplementary figure 1A/His6/Replica 1.bmp]

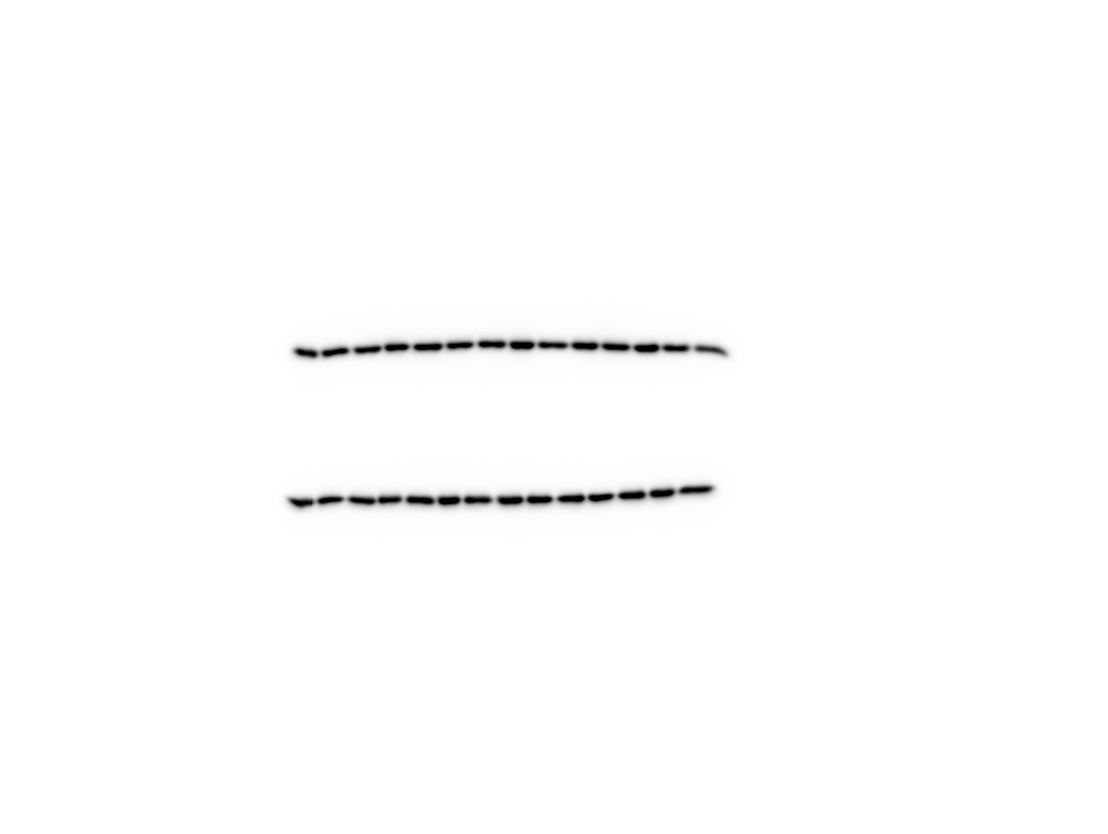

Supplement: Figure 1—figure supplement 1—source data 3. [file elife-84319-fig1-figsupp1-data3.zip › Figure 1ΓÇôSupplementary figure 1-Source Data 2/Supplementary figure 1A/GFP/Replica 2_3.tif]

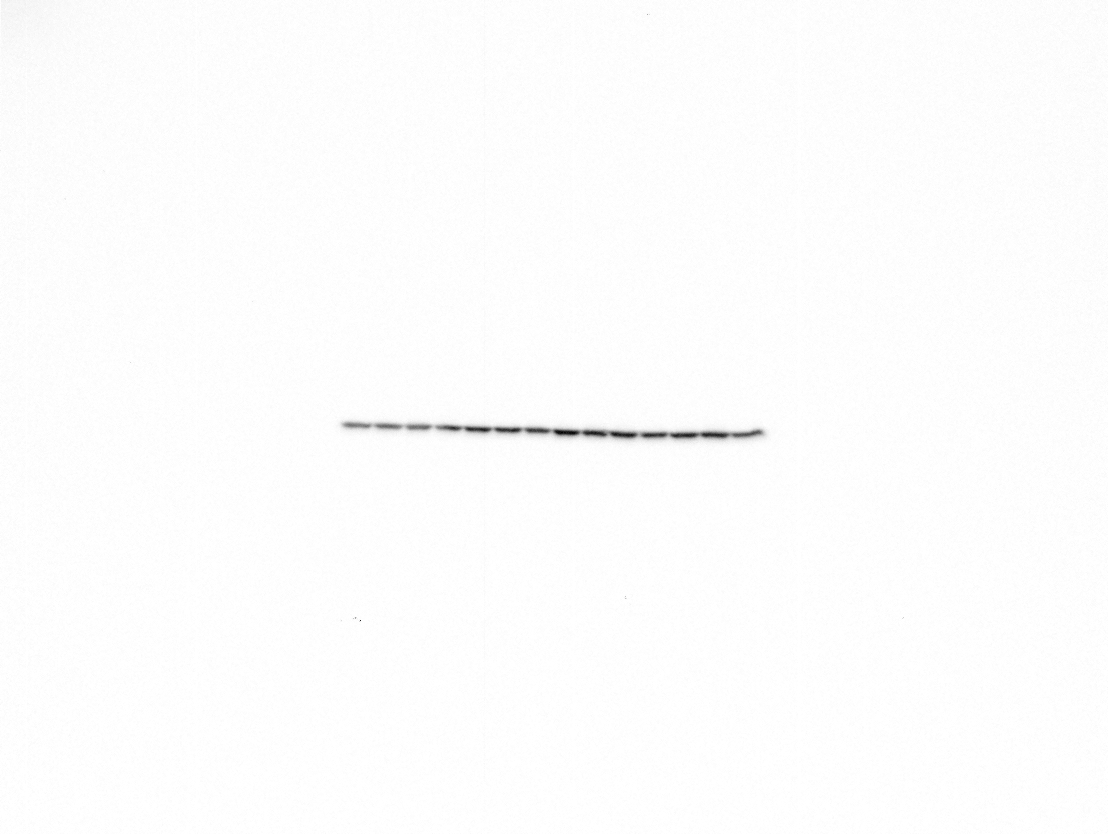

Supplement: Figure 1—figure supplement 1—source data 3. [file elife-84319-fig1-figsupp1-data3.zip › Figure 1ΓÇôSupplementary figure 1-Source Data 2/Supplementary figure 1A/GFP/Replica 1.bmp]

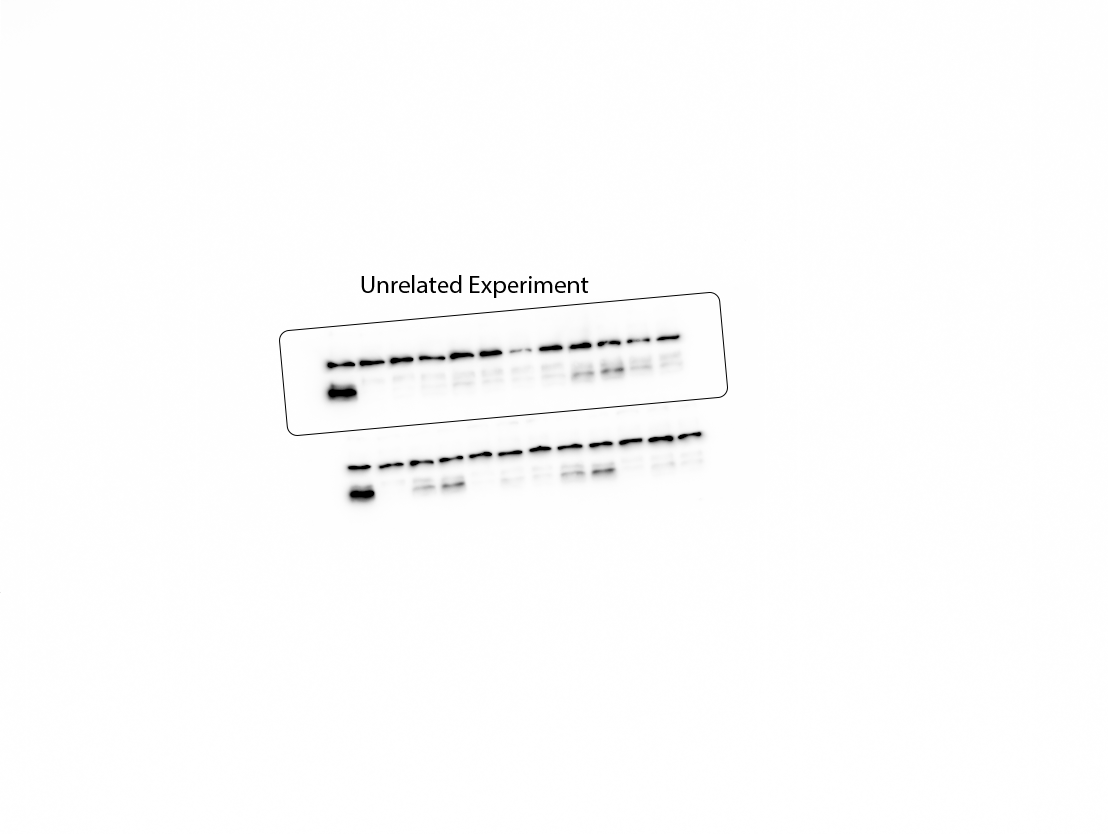

Supplement: Figure 2—source data 3. [file elife-84319-fig2-data3.zip › Figure 2ΓÇôSource Data 3/Figure 2A/Sch9-pThr737/Replica 3.tif]

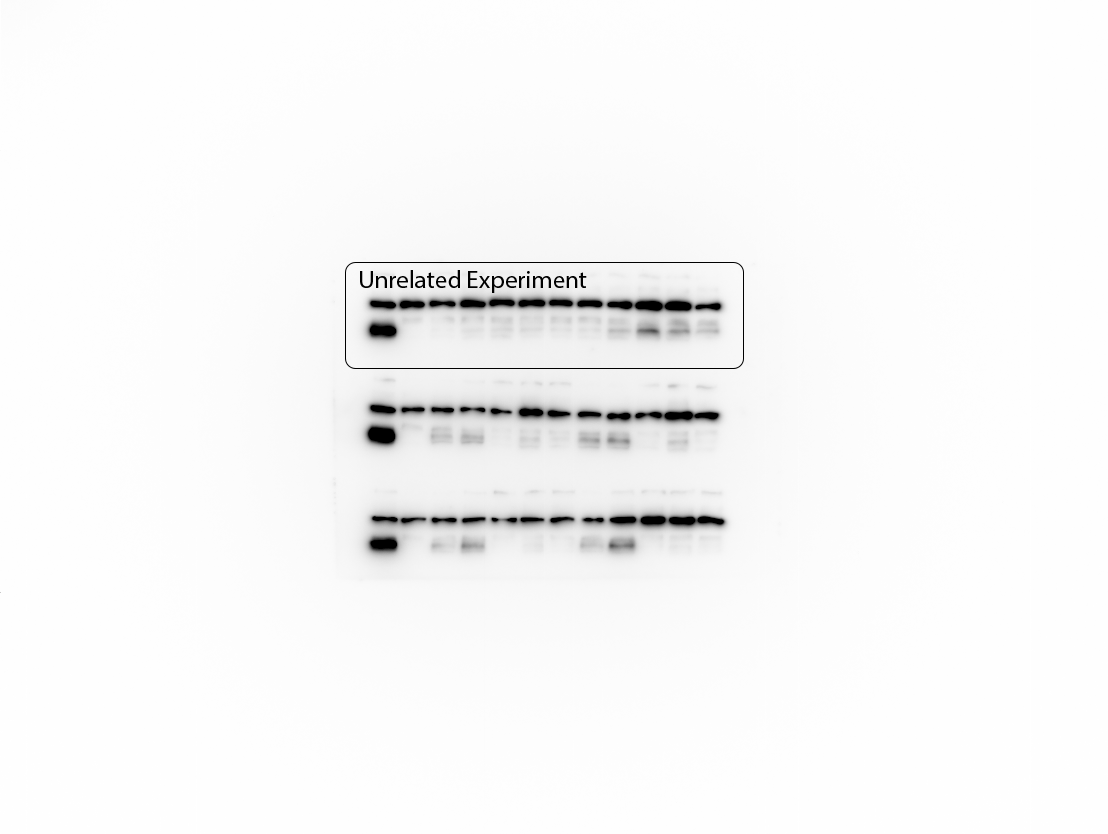

Supplement: Figure 2—source data 3. [file elife-84319-fig2-data3.zip › Figure 2ΓÇôSource Data 3/Figure 2A/Sch9-pThr737/Replica 1_2.tif]

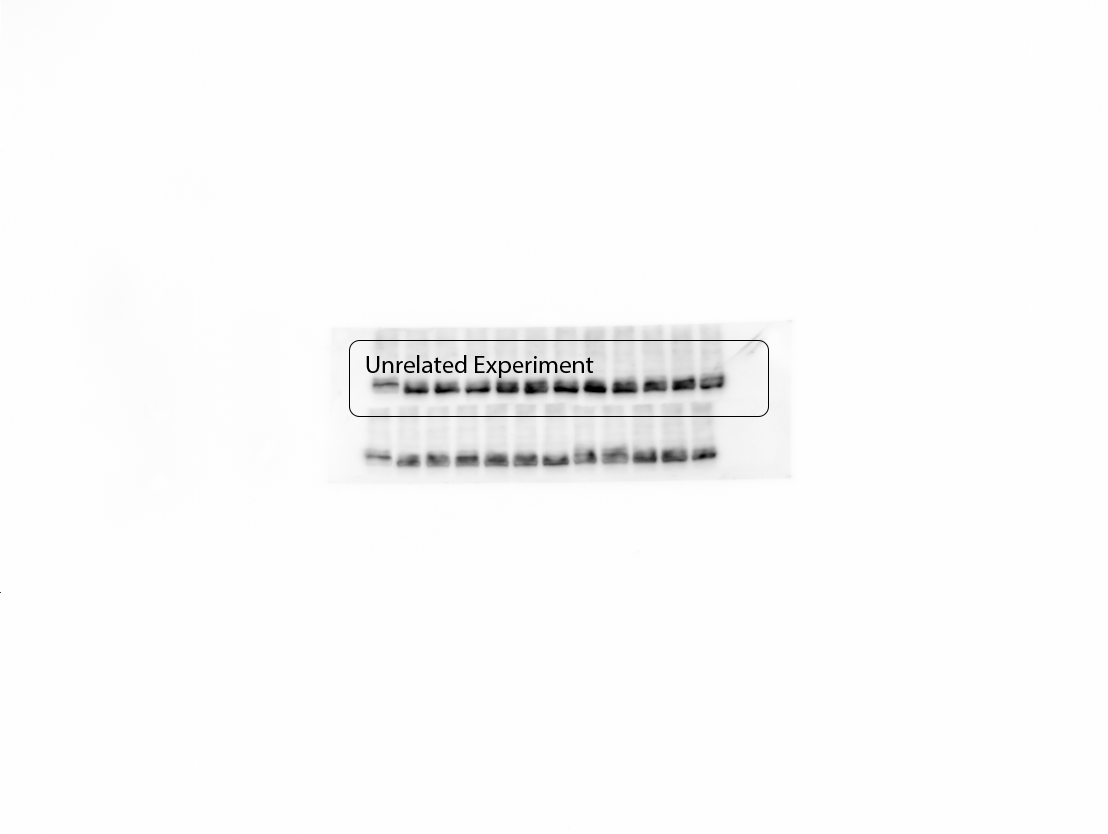

Supplement: Figure 2—source data 3. [file elife-84319-fig2-data3.zip › Figure 2ΓÇôSource Data 3/Figure 2A/Sch9/Replica 3.tif]

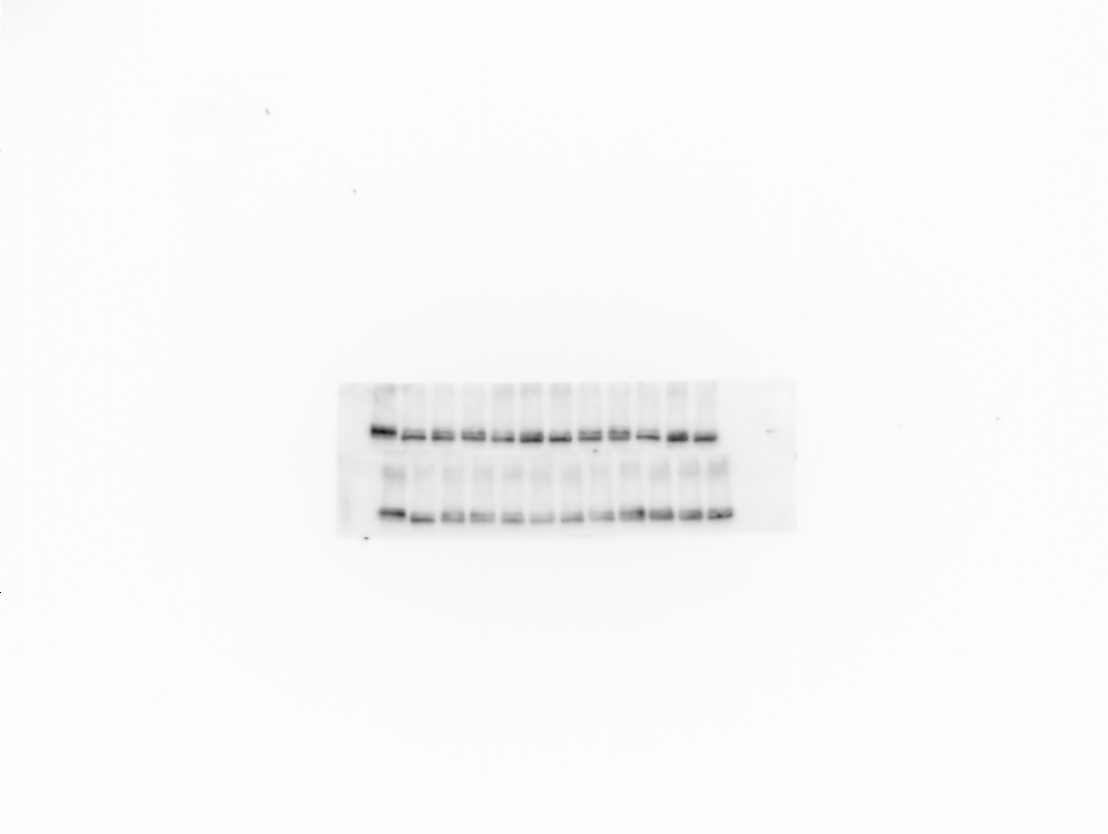

Supplement: Figure 2—source data 3. [file elife-84319-fig2-data3.zip › Figure 2ΓÇôSource Data 3/Figure 2A/Sch9/Replica 1_2.tif]

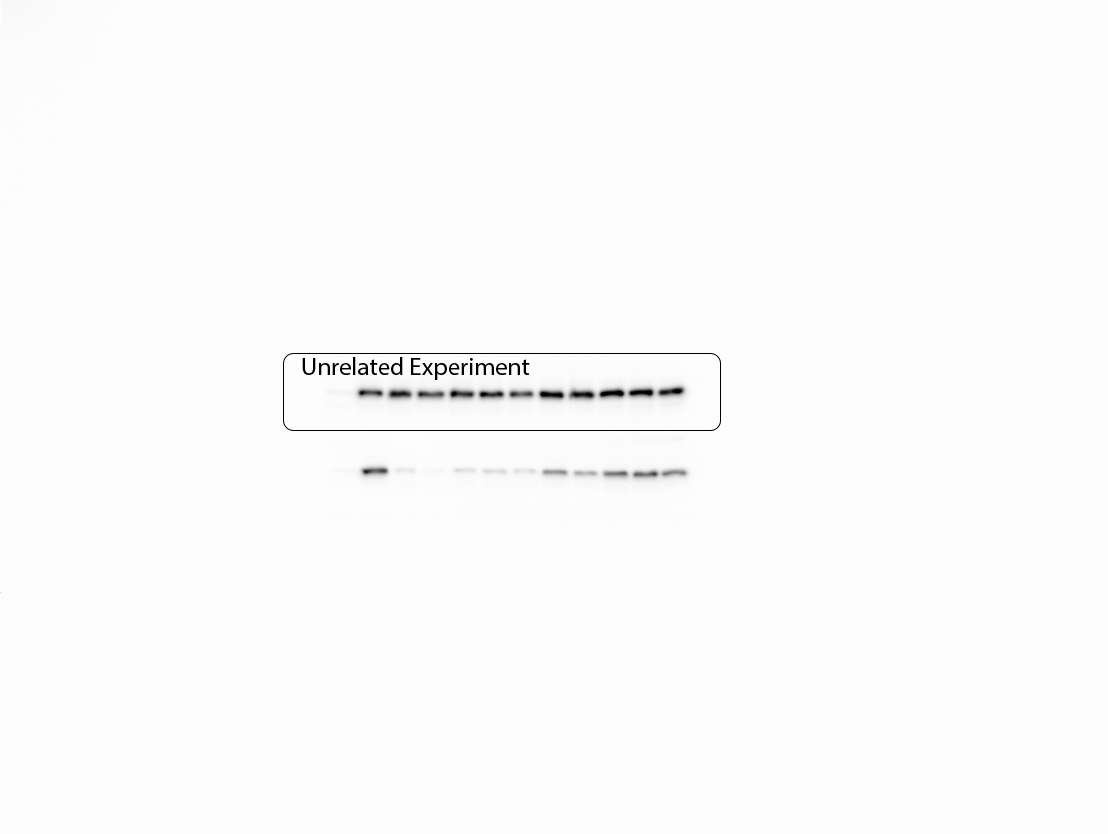

Supplement: Figure 2—source data 3. [file elife-84319-fig2-data3.zip › Figure 2ΓÇôSource Data 3/Figure 2A/Snf1-pThr210/Replica 3.tif]

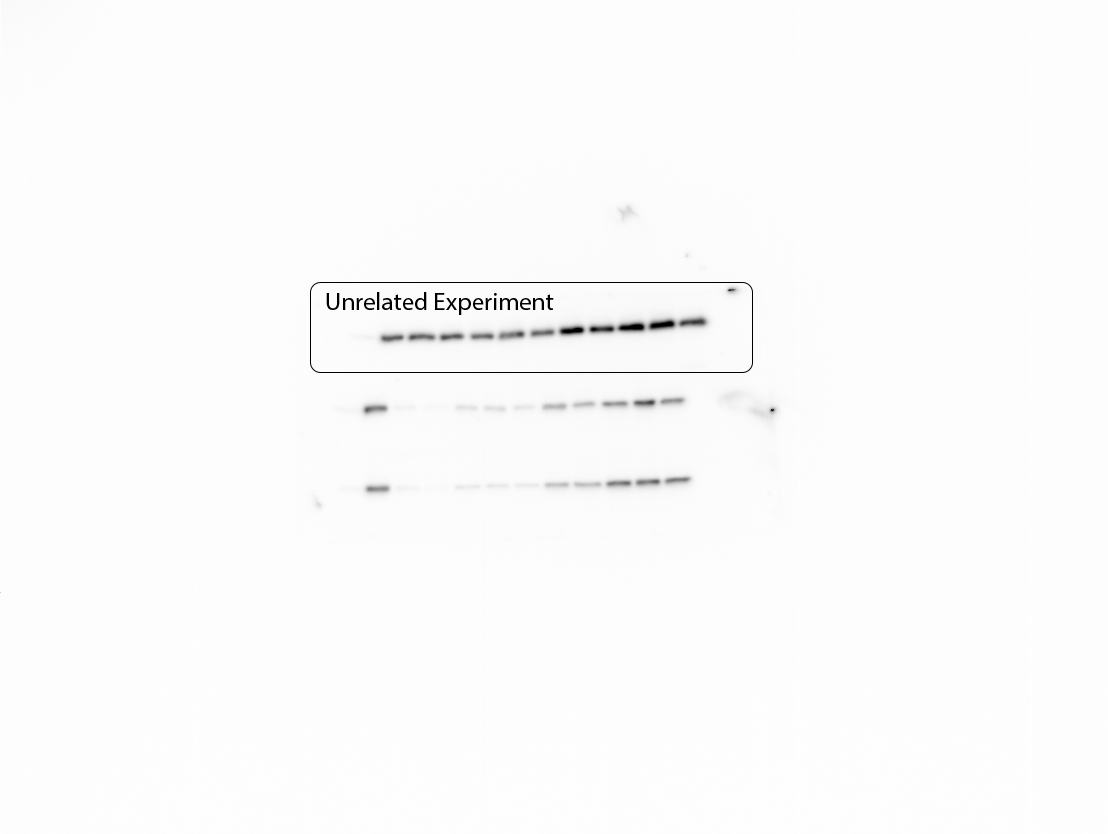

Supplement: Figure 2—source data 3. [file elife-84319-fig2-data3.zip › Figure 2ΓÇôSource Data 3/Figure 2A/Snf1-pThr210/Replica 1_2.tif]

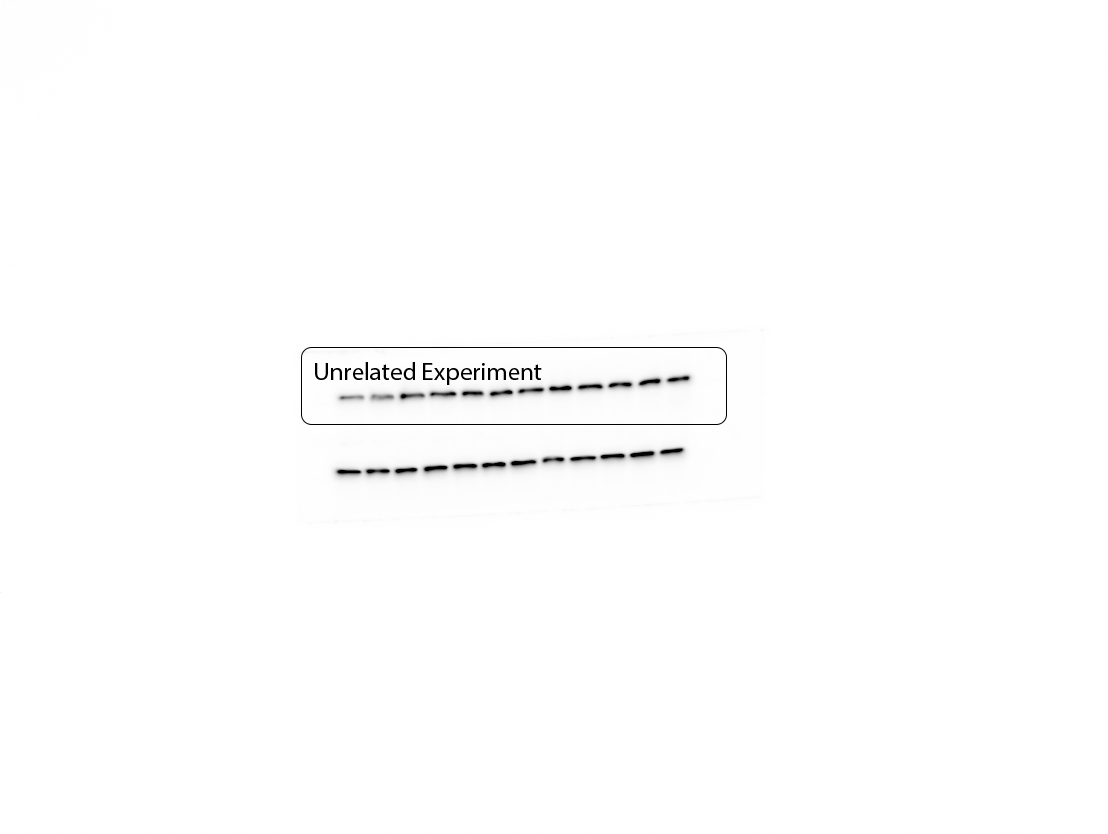

Supplement: Figure 2—source data 3. [file elife-84319-fig2-data3.zip › Figure 2ΓÇôSource Data 3/Figure 2A/His6/Replica 3.tif]

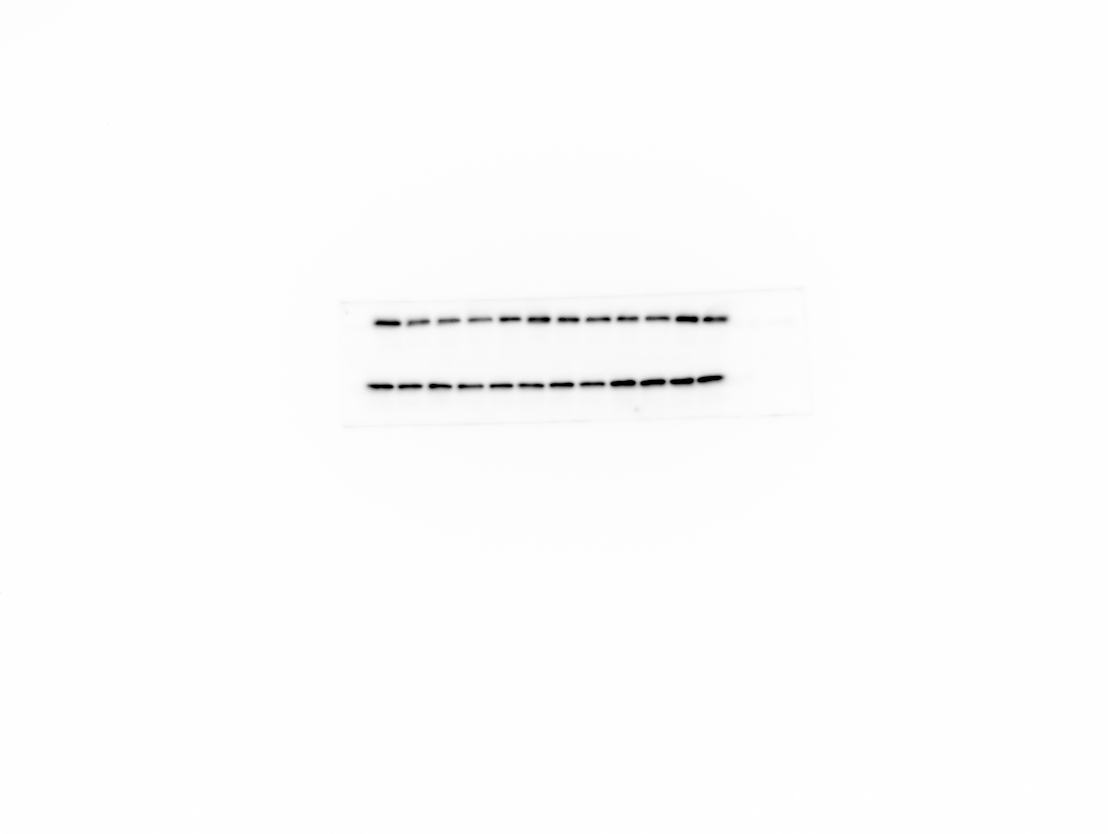

Supplement: Figure 2—source data 3. [file elife-84319-fig2-data3.zip › Figure 2ΓÇôSource Data 3/Figure 2A/His6/Replica 1_2.tif]

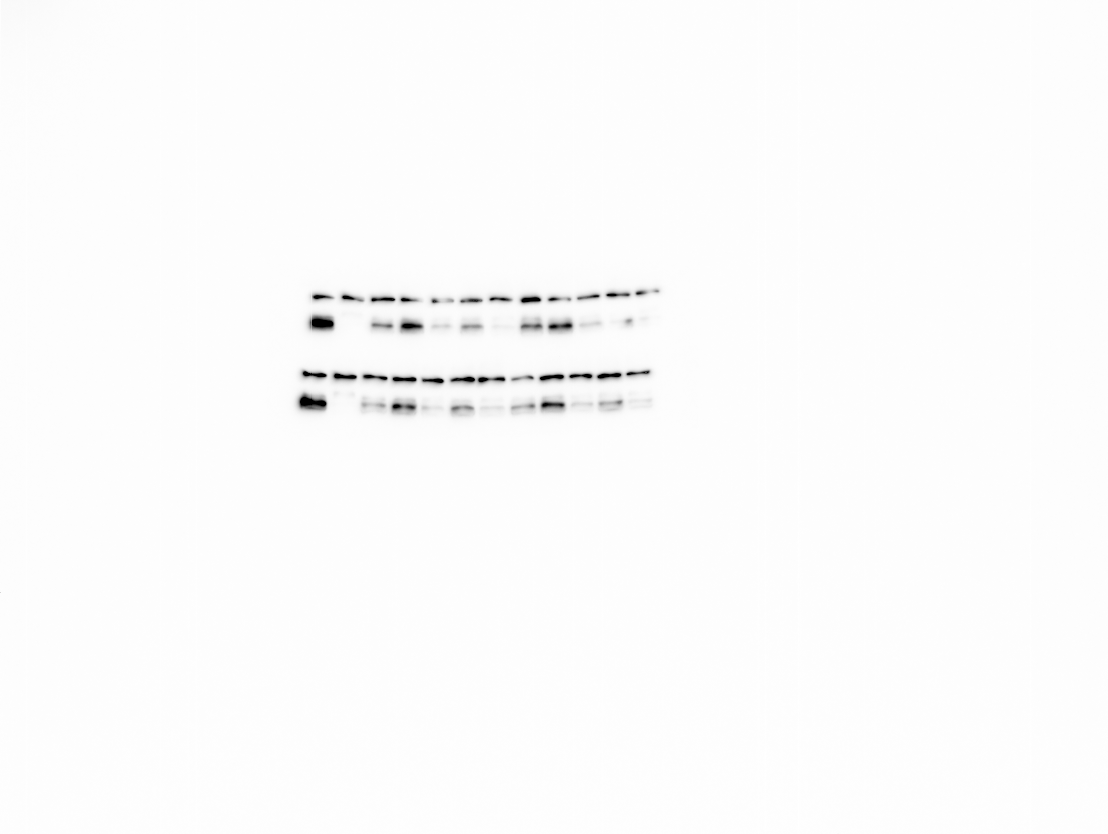

Supplement: Figure 2—source data 3. [file elife-84319-fig2-data3.zip › Figure 2ΓÇôSource Data 3/Figure 2C/Sch9-pThr737/Replica 2_3.tif]

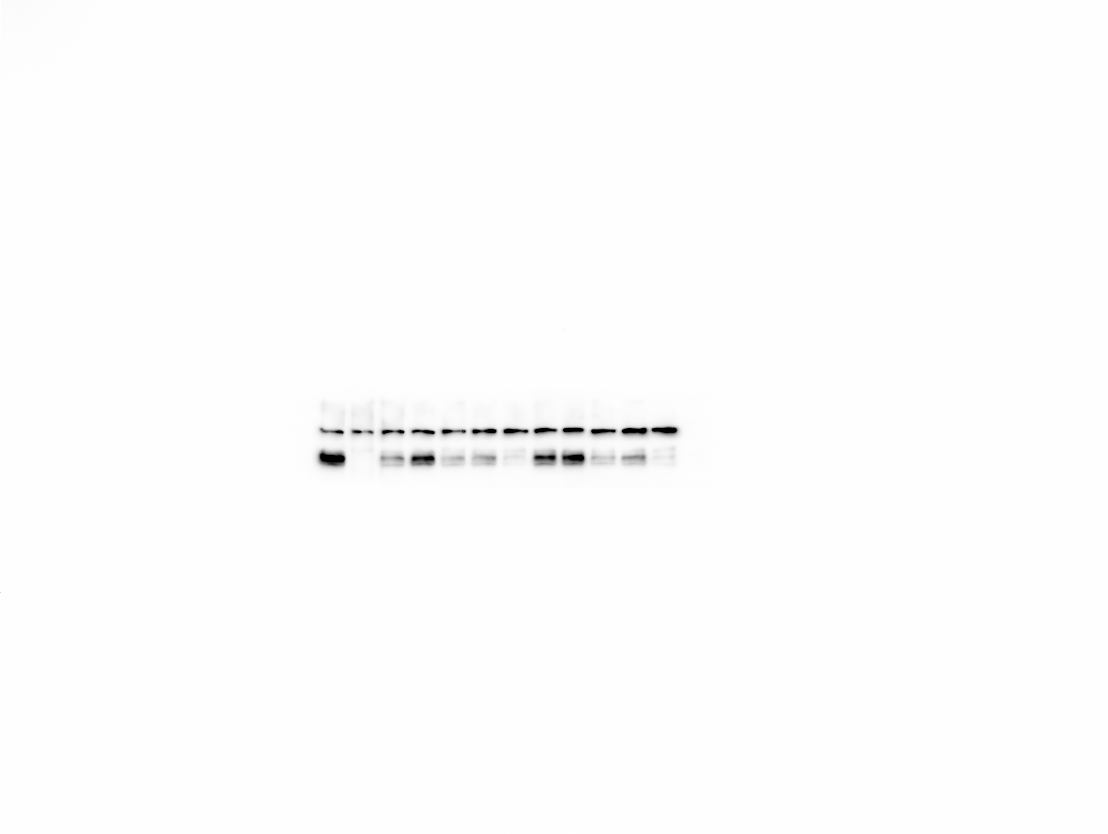

Supplement: Figure 2—source data 3. [file elife-84319-fig2-data3.zip › Figure 2ΓÇôSource Data 3/Figure 2C/Sch9-pThr737/Replica 1.tif]

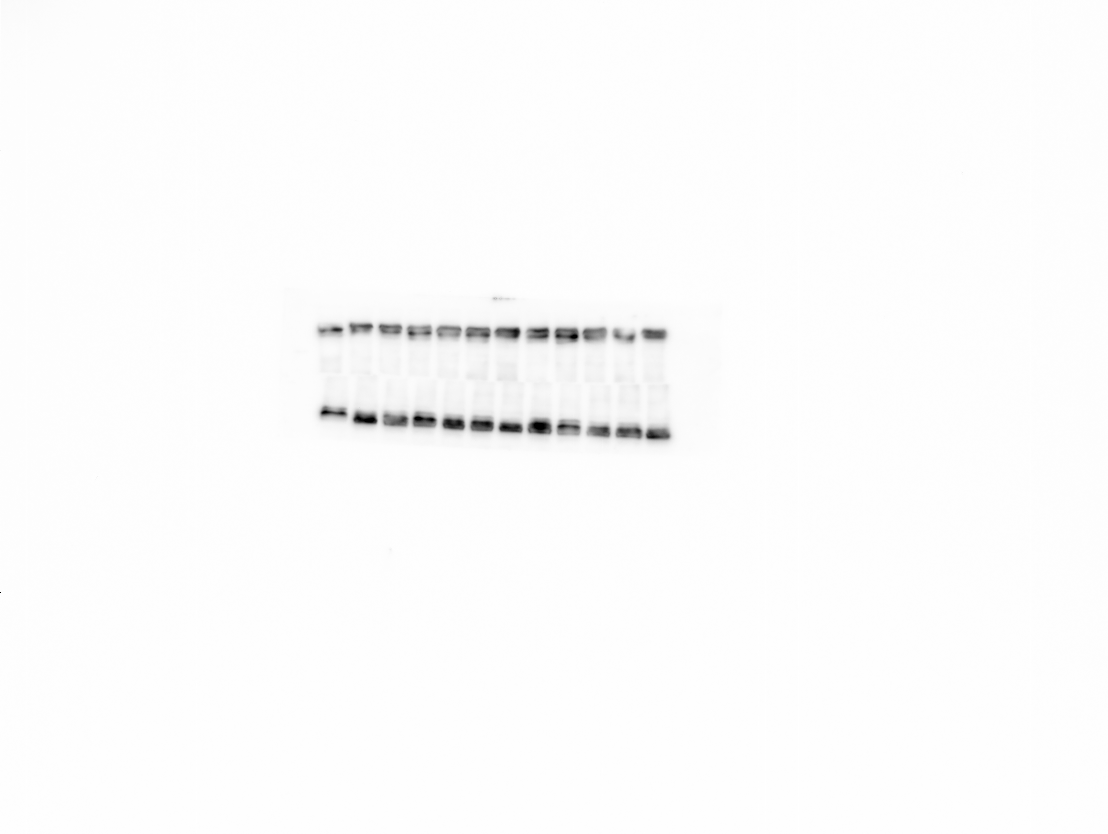

Supplement: Figure 2—source data 3. [file elife-84319-fig2-data3.zip › Figure 2ΓÇôSource Data 3/Figure 2C/Sch9/Replica 2_3.tif]

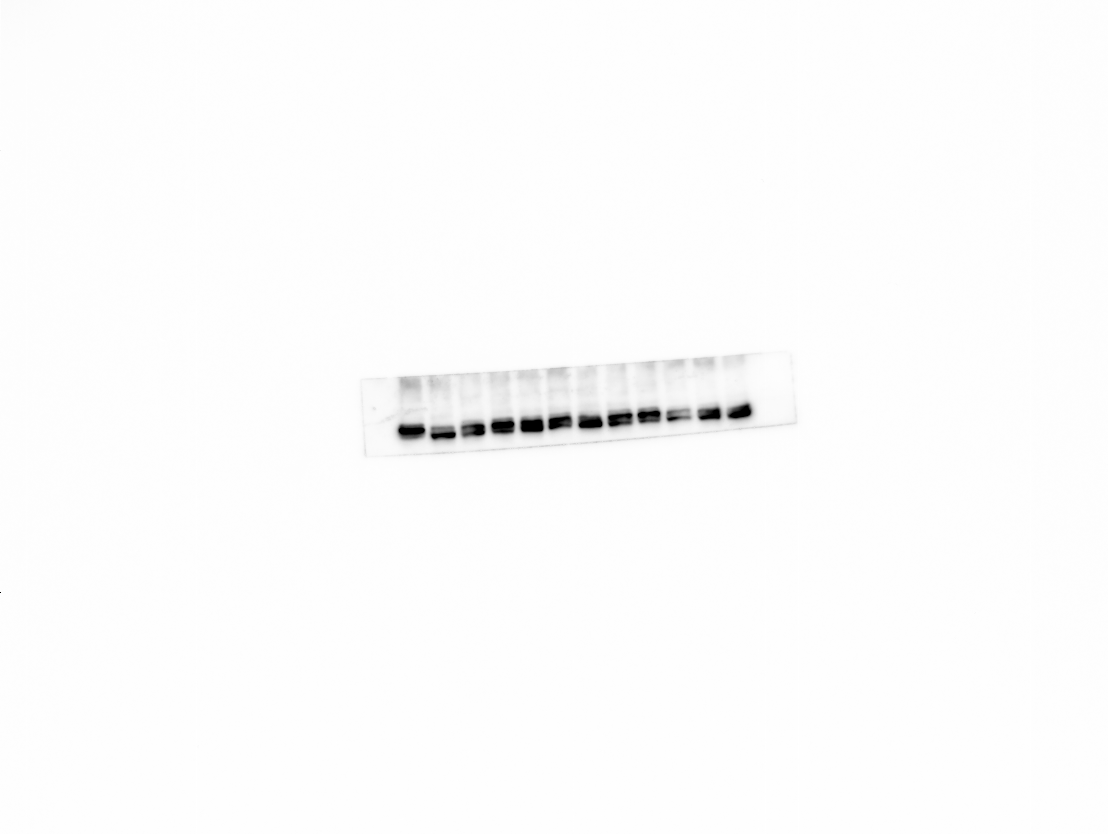

Supplement: Figure 2—source data 3. [file elife-84319-fig2-data3.zip › Figure 2ΓÇôSource Data 3/Figure 2C/Sch9/Replica 1.tif]

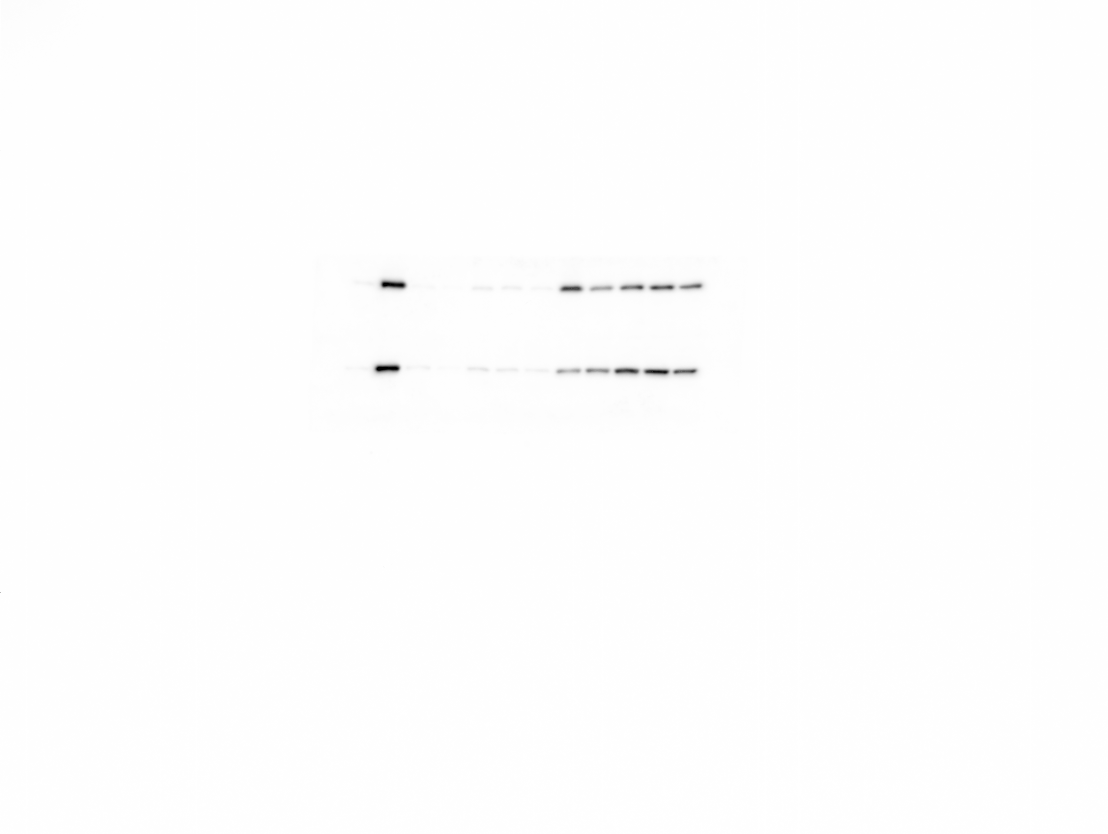

Supplement: Figure 2—source data 3. [file elife-84319-fig2-data3.zip › Figure 2ΓÇôSource Data 3/Figure 2C/Snf1-pThr210/Replica 2_3.tif]

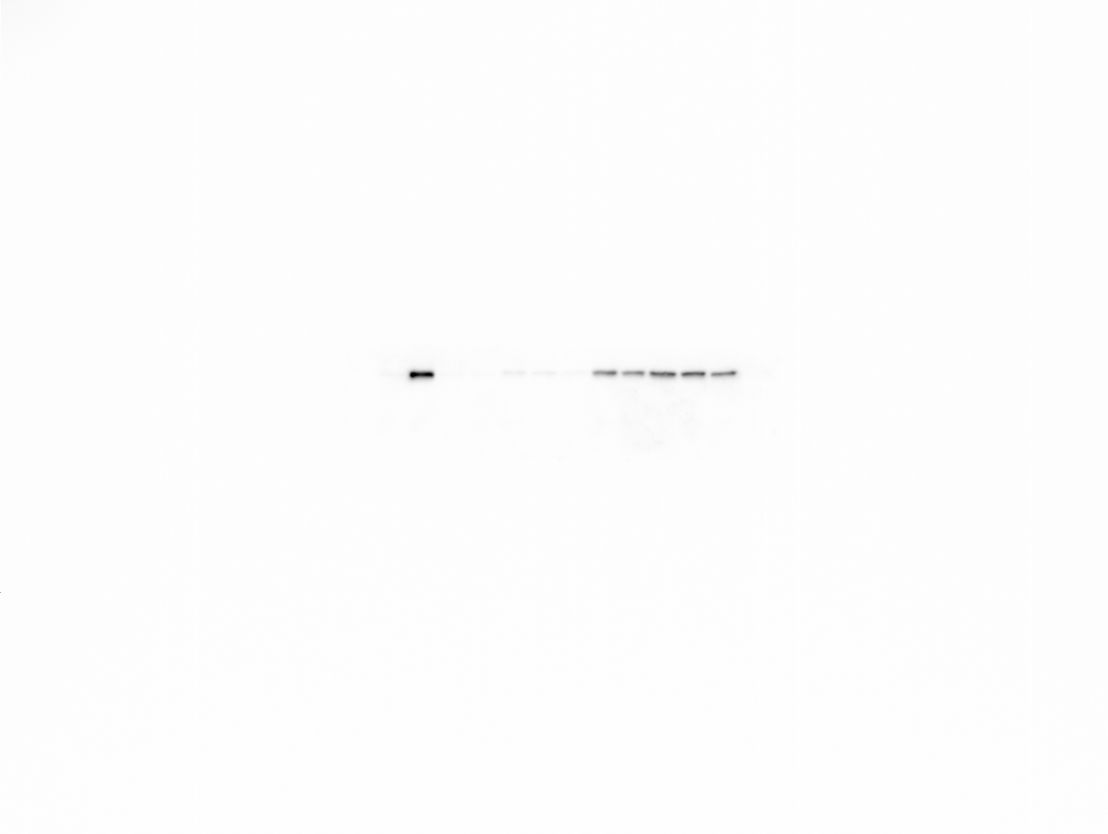

Supplement: Figure 2—source data 3. [file elife-84319-fig2-data3.zip › Figure 2ΓÇôSource Data 3/Figure 2C/Snf1-pThr210/Replica 1.tif]

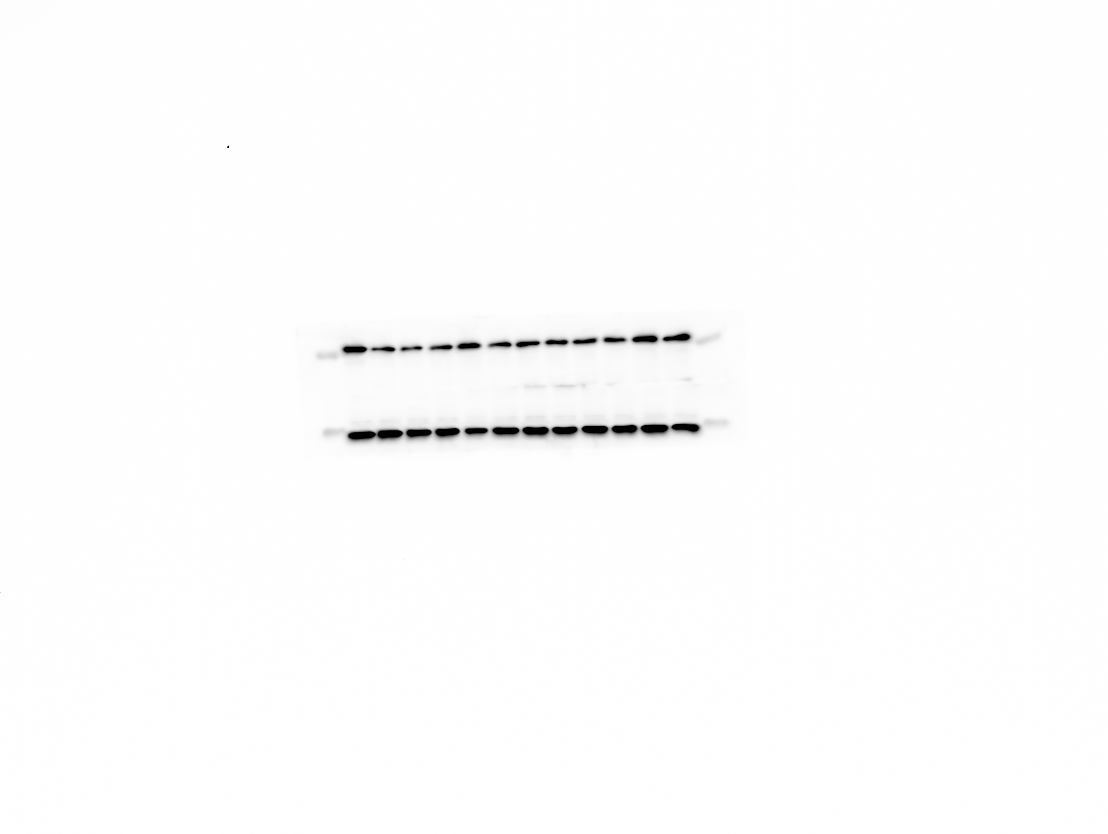

Supplement: Figure 2—source data 3. [file elife-84319-fig2-data3.zip › Figure 2ΓÇôSource Data 3/Figure 2C/His6/Replica 2_3.tif]

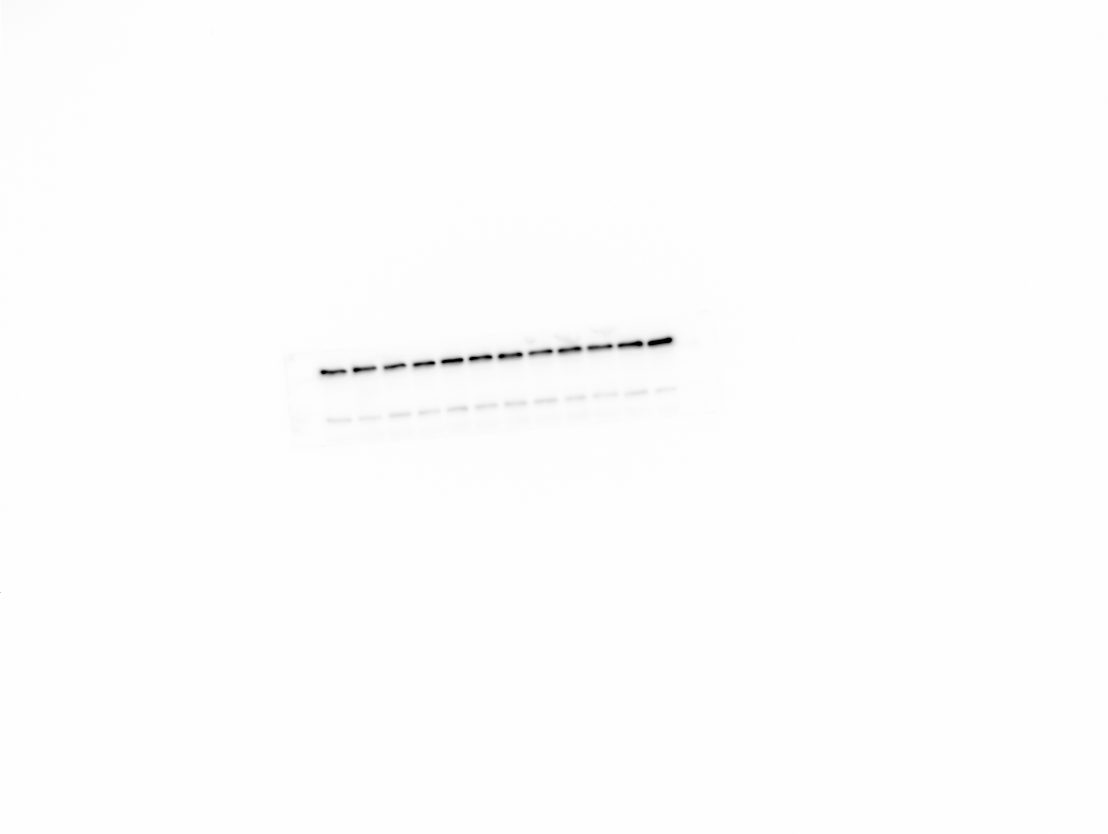

Supplement: Figure 2—source data 3. [file elife-84319-fig2-data3.zip › Figure 2ΓÇôSource Data 3/Figure 2C/His6/Replica 1.tif]

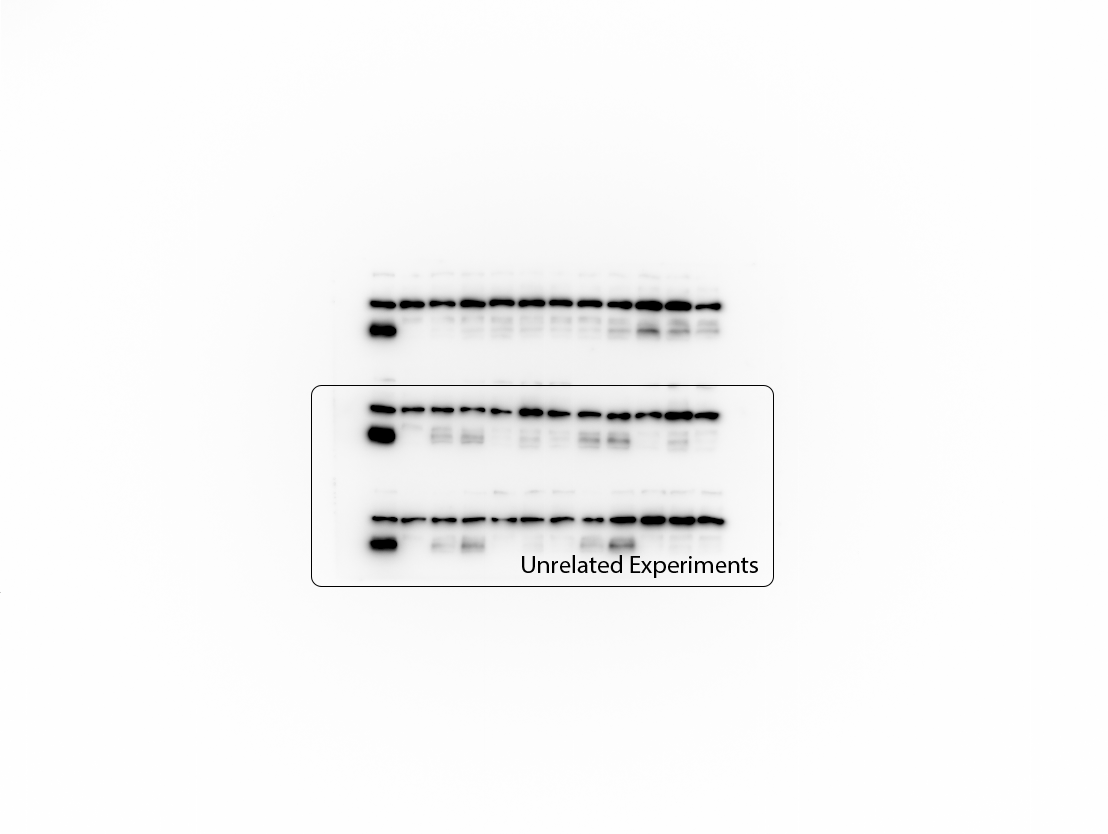

Supplement: Figure 2—source data 3. [file elife-84319-fig2-data3.zip › Figure 2ΓÇôSource Data 3/Figure 2E/Sch9-pThr737/Replica 2.tif]

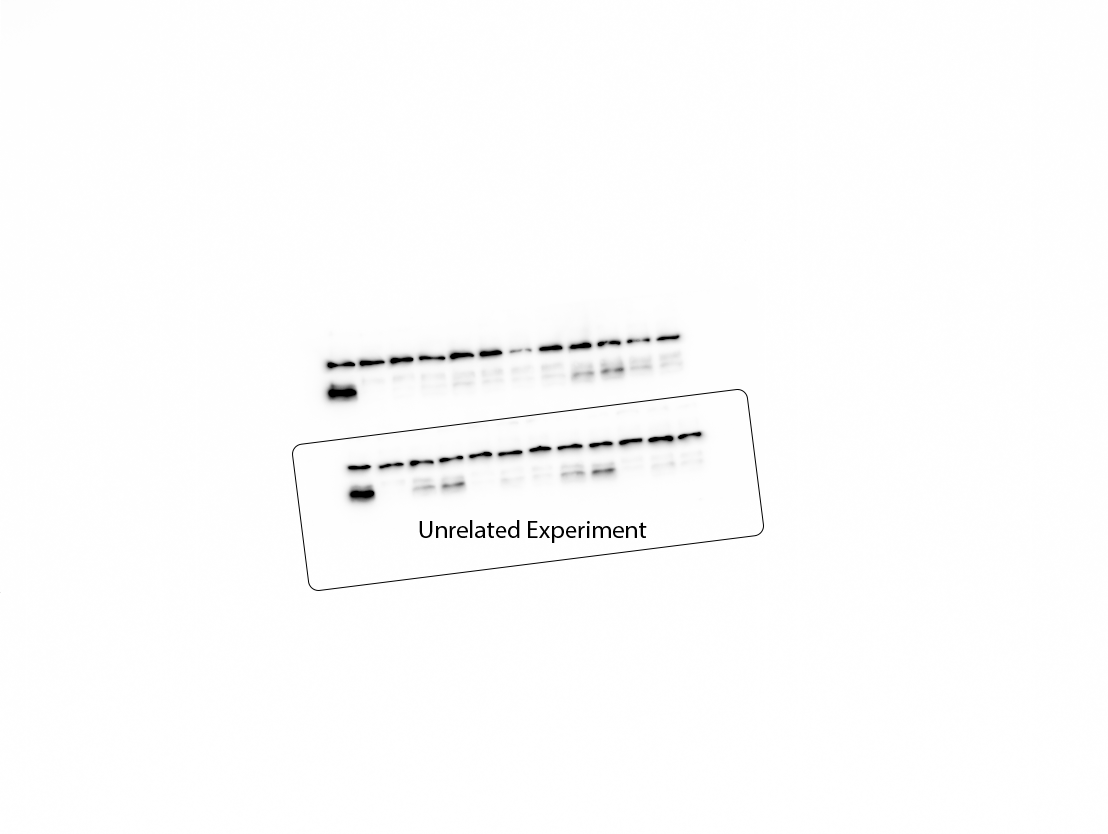

Supplement: Figure 2—source data 3. [file elife-84319-fig2-data3.zip › Figure 2ΓÇôSource Data 3/Figure 2E/Sch9-pThr737/Replica 3.tif]

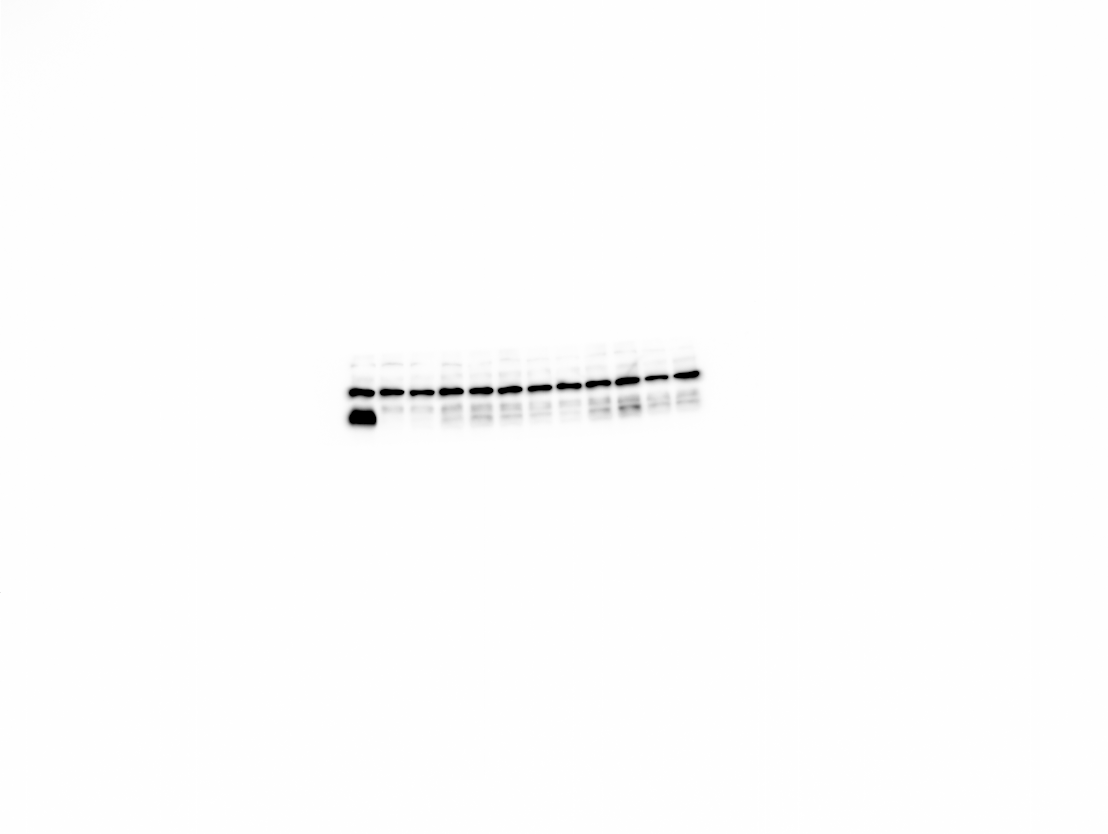

Supplement: Figure 2—source data 3. [file elife-84319-fig2-data3.zip › Figure 2ΓÇôSource Data 3/Figure 2E/Sch9-pThr737/Replica 1.tif]

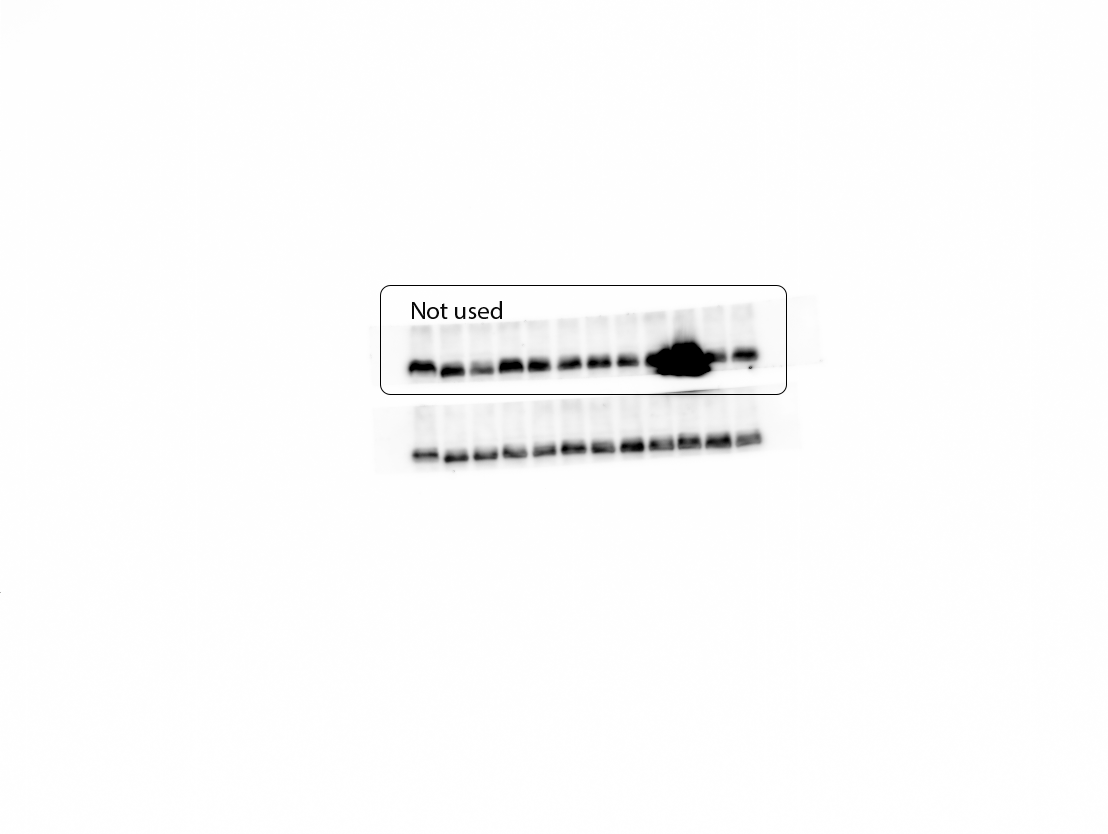

Supplement: Figure 2—source data 3. [file elife-84319-fig2-data3.zip › Figure 2ΓÇôSource Data 3/Figure 2E/Sch9/Replica 2.tif]

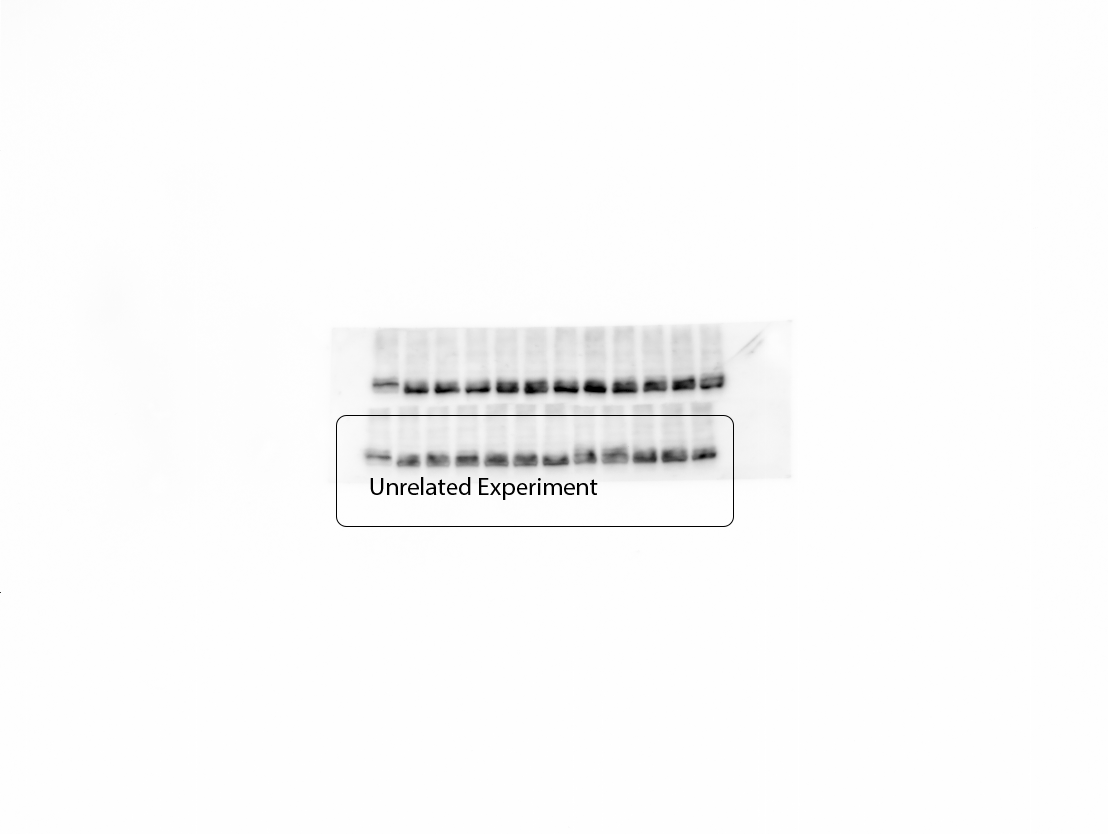

Supplement: Figure 2—source data 3. [file elife-84319-fig2-data3.zip › Figure 2ΓÇôSource Data 3/Figure 2E/Sch9/Replica 3.tif]

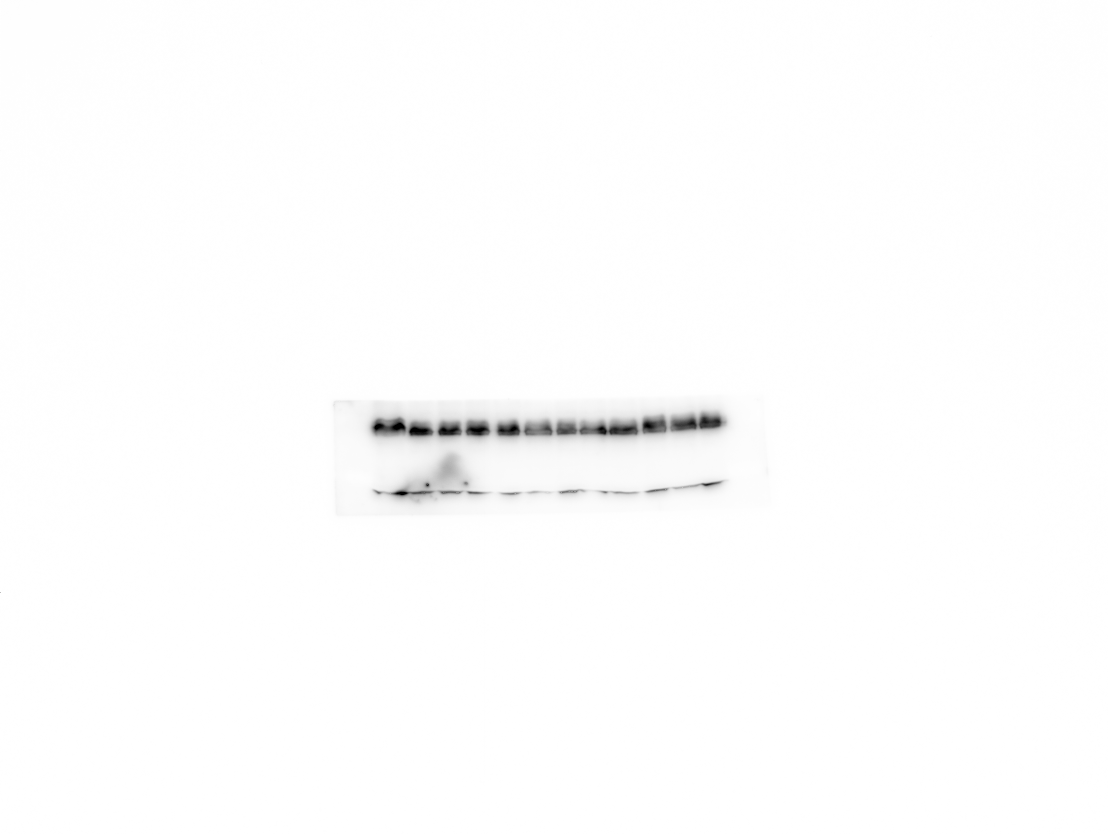

Supplement: Figure 2—source data 3. [file elife-84319-fig2-data3.zip › Figure 2ΓÇôSource Data 3/Figure 2E/Sch9/Replica 1.tif]

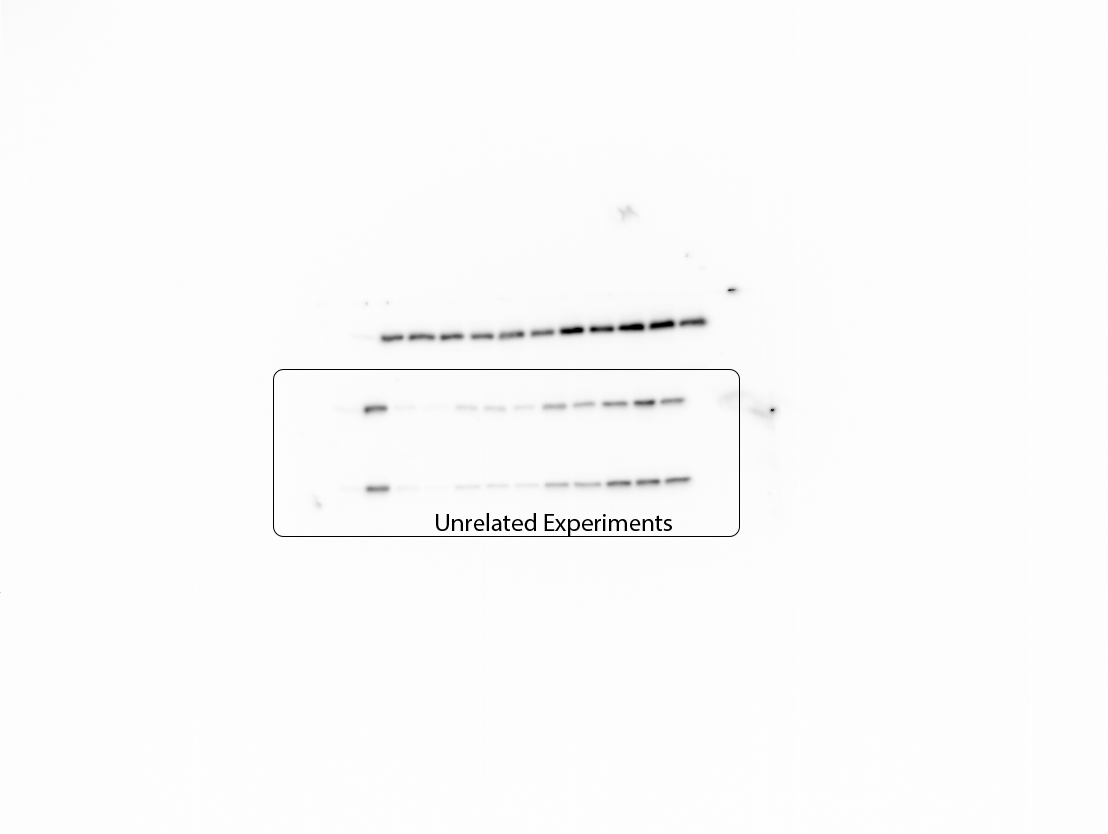

Supplement: Figure 2—source data 3. [file elife-84319-fig2-data3.zip › Figure 2ΓÇôSource Data 3/Figure 2E/Snf1-pThr210/Replica 2.tif]

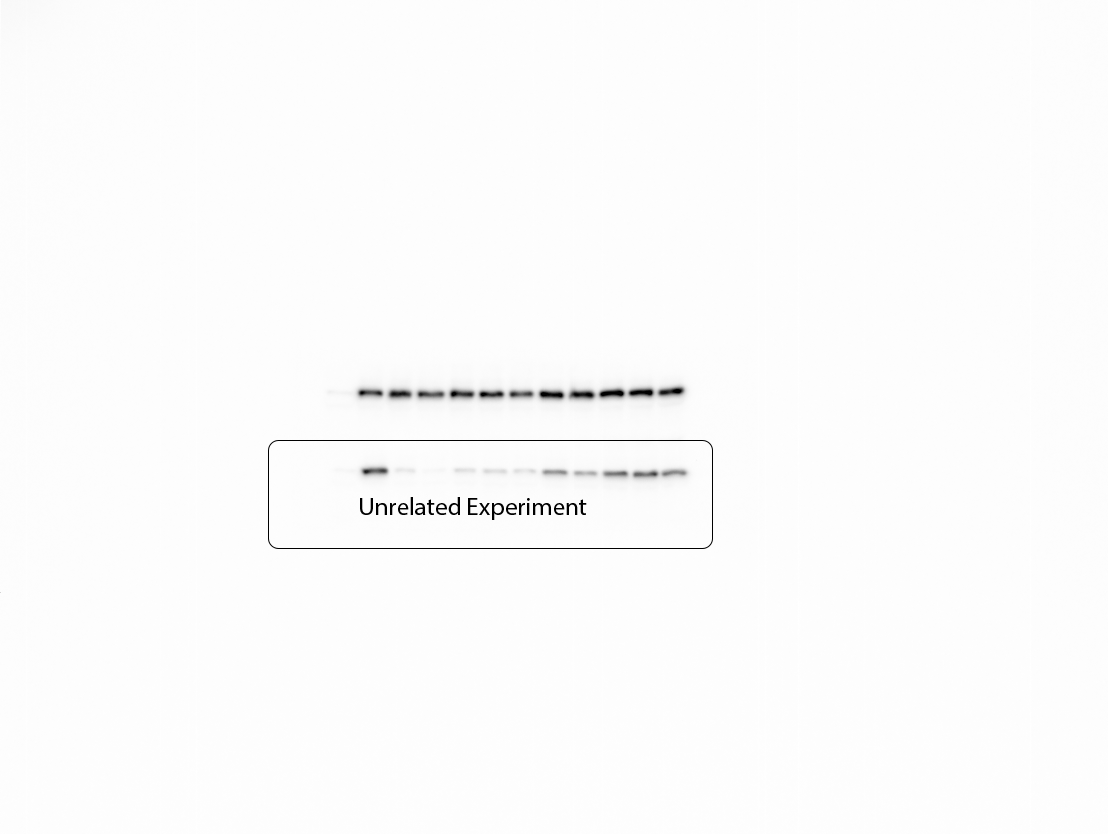

Supplement: Figure 2—source data 3. [file elife-84319-fig2-data3.zip › Figure 2ΓÇôSource Data 3/Figure 2E/Snf1-pThr210/Replica 3.tif]

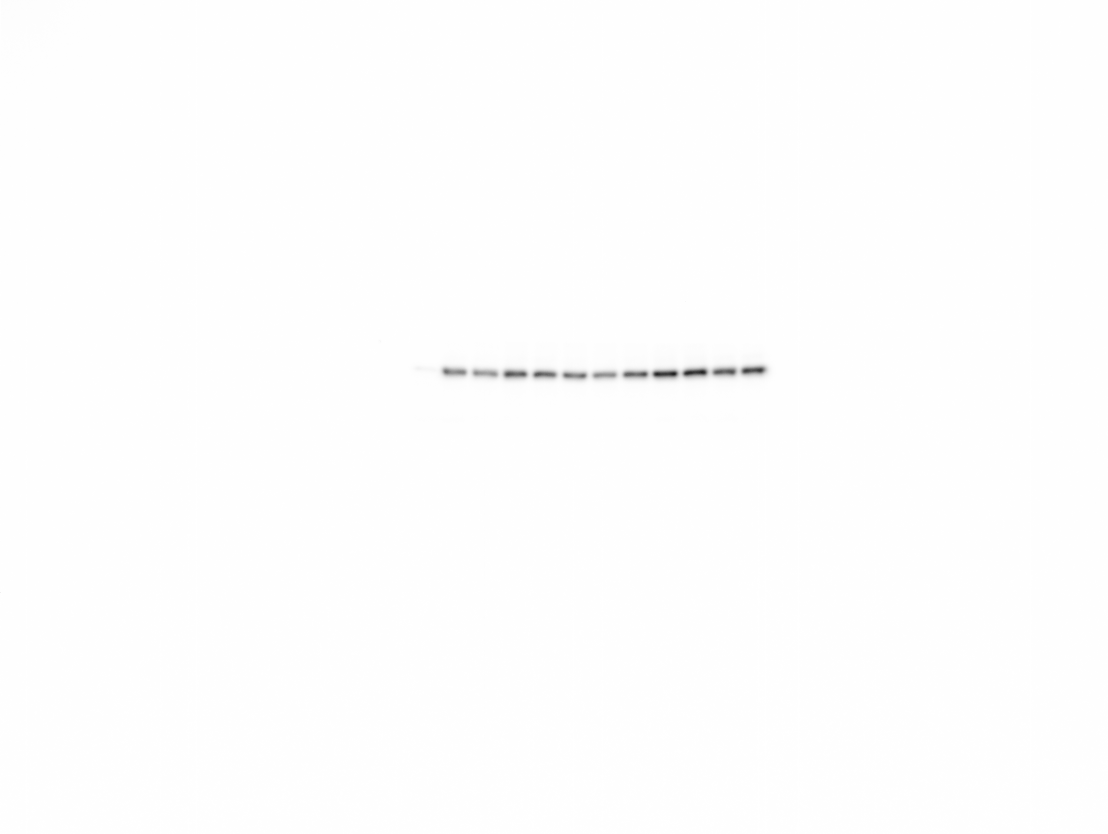

Supplement: Figure 2—source data 3. [file elife-84319-fig2-data3.zip › Figure 2ΓÇôSource Data 3/Figure 2E/Snf1-pThr210/Replica 1.tif]

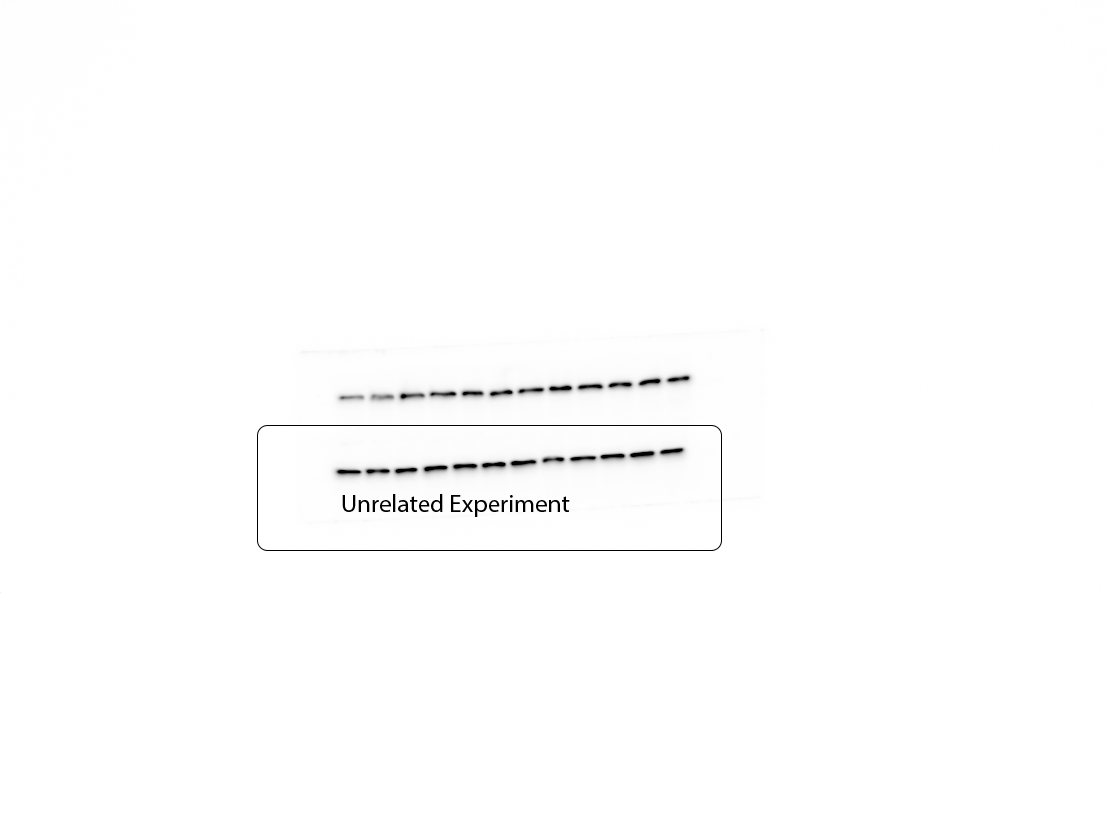

Supplement: Figure 2—source data 3. [file elife-84319-fig2-data3.zip › Figure 2ΓÇôSource Data 3/Figure 2E/His6/Replica 3.tif]

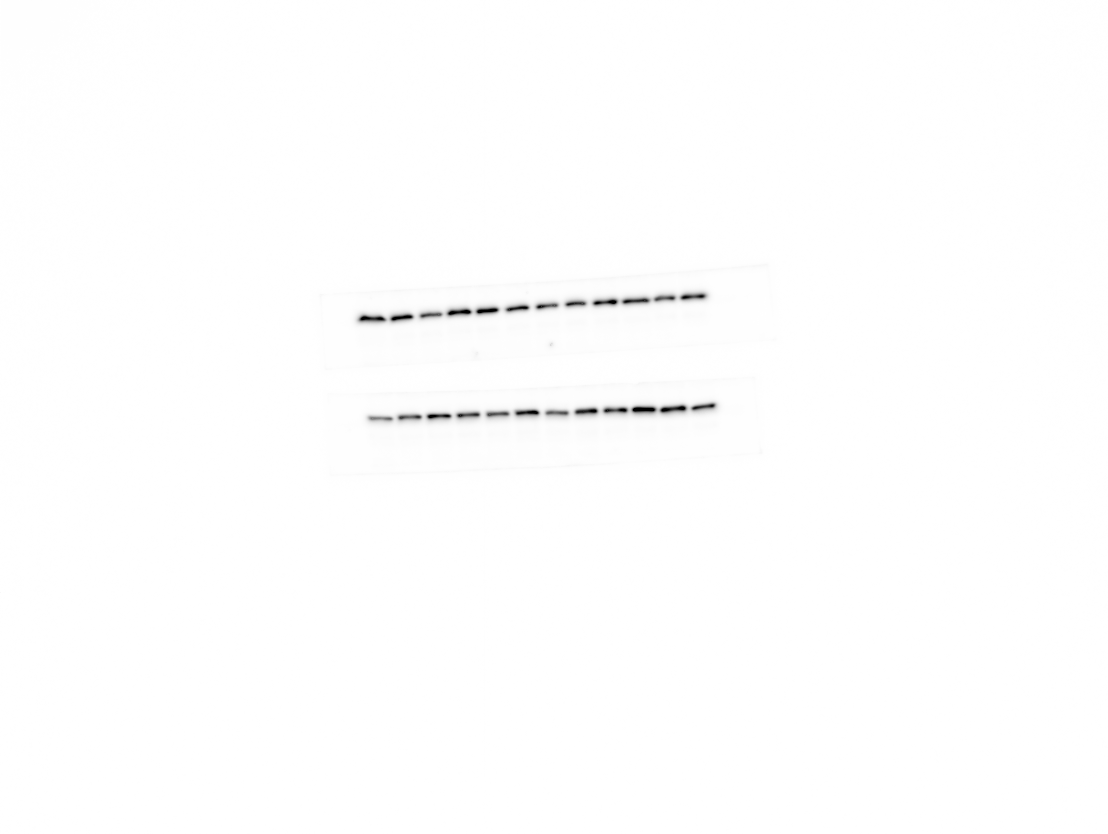

Supplement: Figure 2—source data 3. [file elife-84319-fig2-data3.zip › Figure 2ΓÇôSource Data 3/Figure 2E/His6/Replica 1_2.tif]

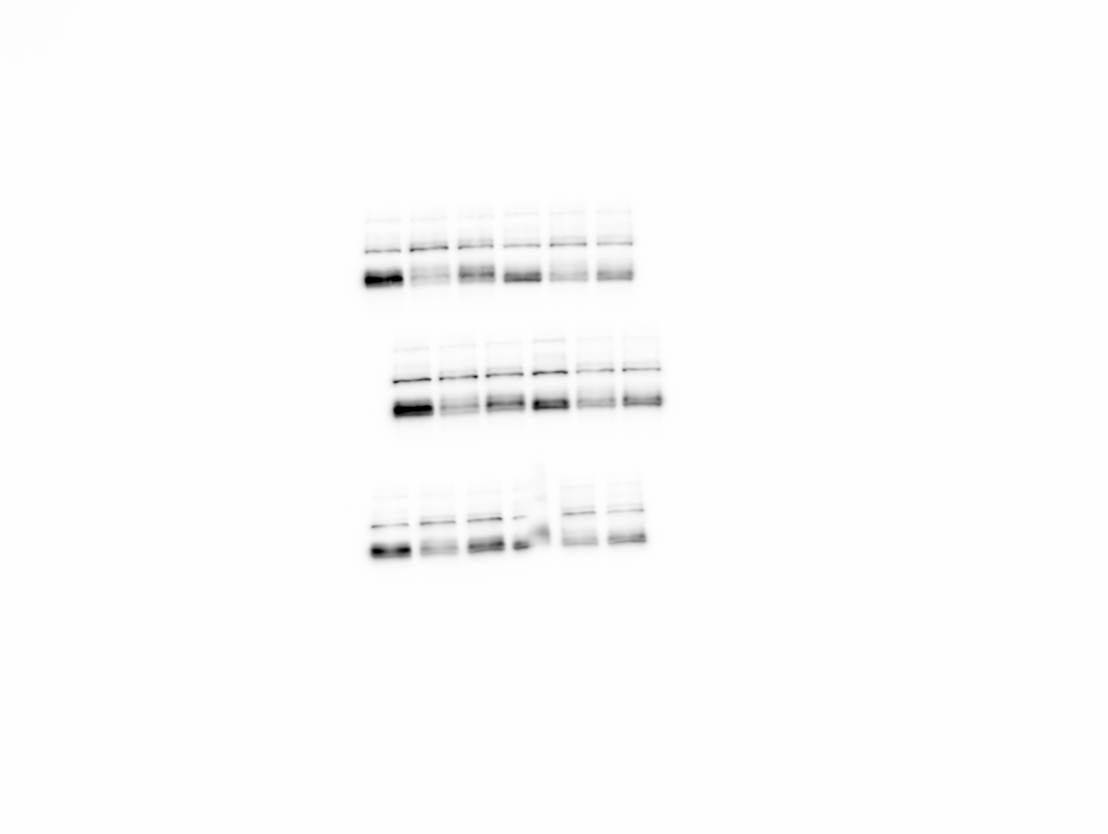

Supplement: Figure 3—figure supplement 1—source data 3. [file elife-84319-fig3-figsupp1-data3.zip › Figure 3ΓÇôfigure supplement 1-source data 3/Figure 3-figure supplement 1C/Sch9-pThr737/Replica 1_2_3.tif]

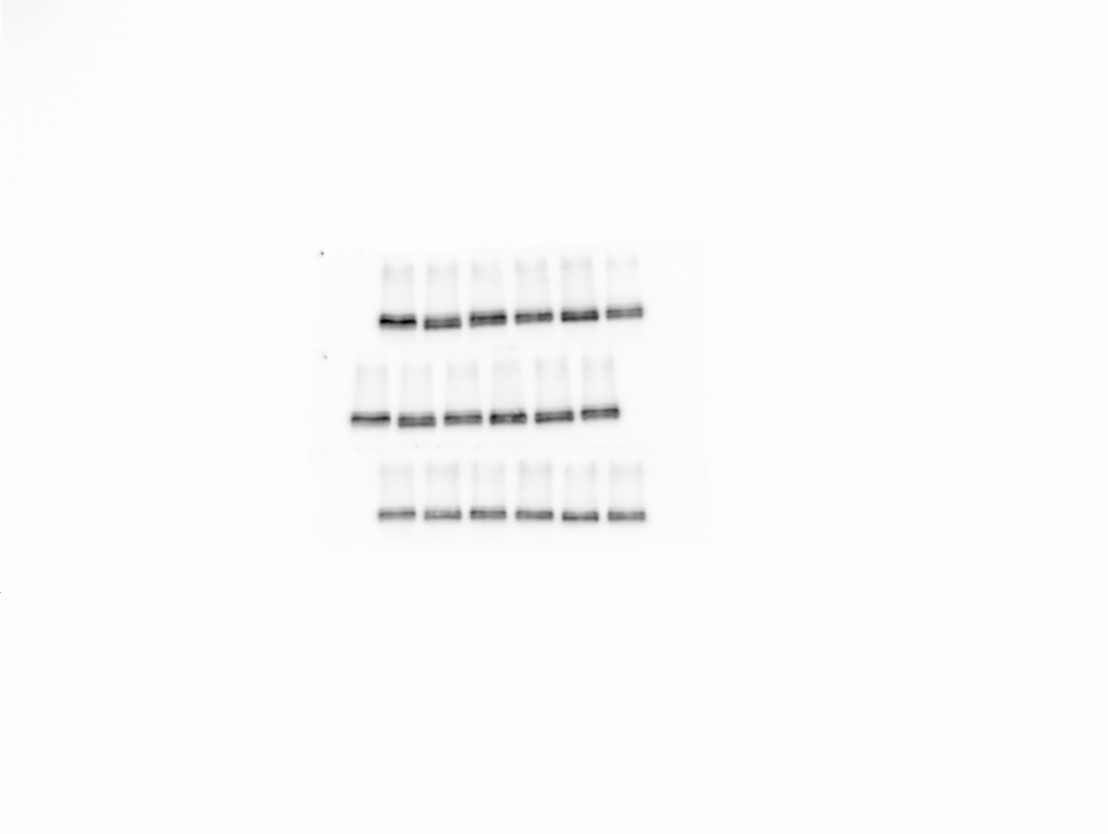

Supplement: Figure 3—figure supplement 1—source data 3. [file elife-84319-fig3-figsupp1-data3.zip › Figure 3ΓÇôfigure supplement 1-source data 3/Figure 3-figure supplement 1C/Sch9/Replica 1_2_3.tif]

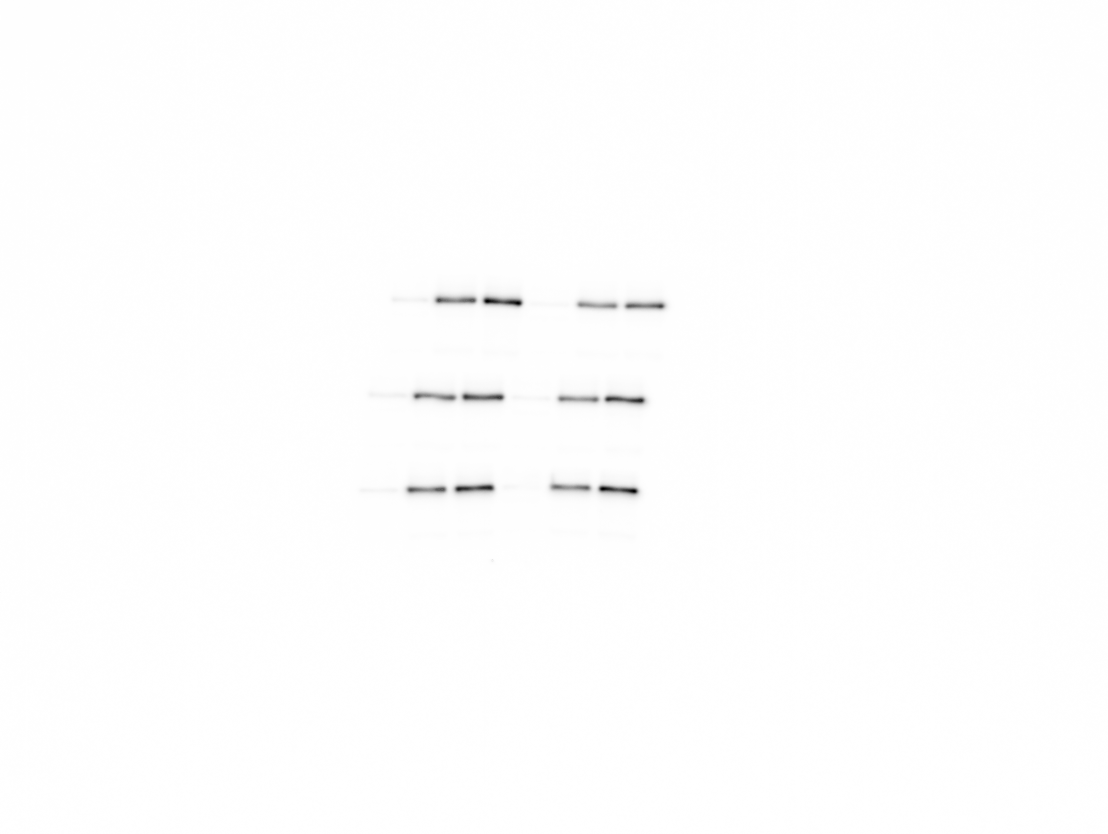

Supplement: Figure 3—figure supplement 1—source data 3. [file elife-84319-fig3-figsupp1-data3.zip › Figure 3ΓÇôfigure supplement 1-source data 3/Figure 3-figure supplement 1C/Snf1-pThr210/Replica 1_2_3.tif]

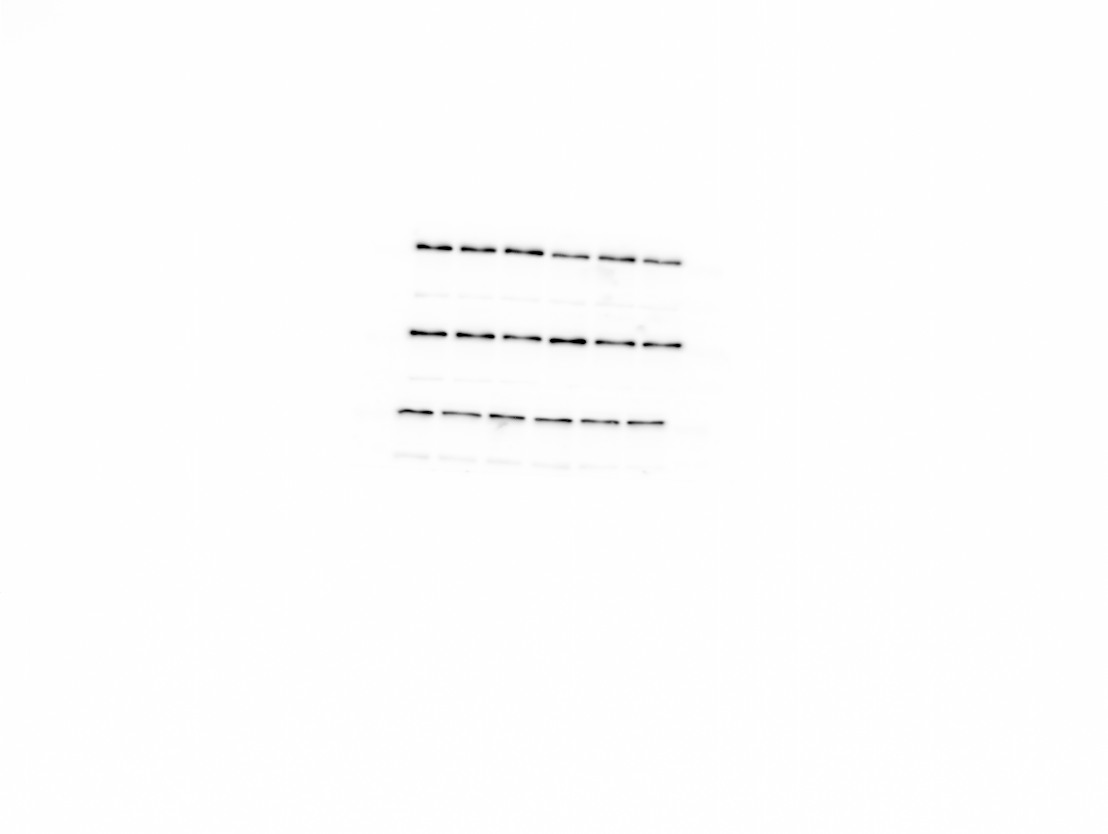

Supplement: Figure 3—figure supplement 1—source data 3. [file elife-84319-fig3-figsupp1-data3.zip › Figure 3ΓÇôfigure supplement 1-source data 3/Figure 3-figure supplement 1C/His6/Replica 1_2_3.tif]

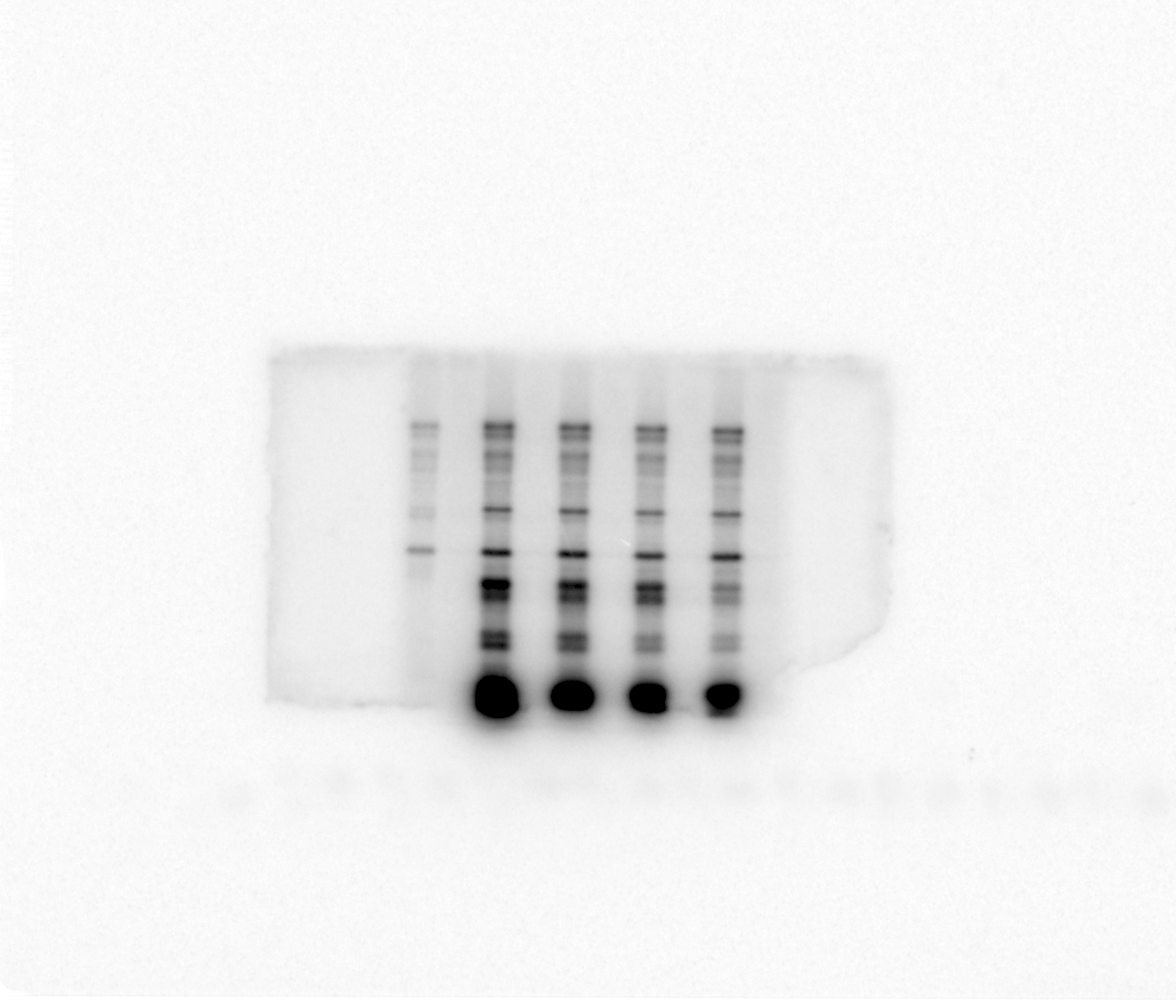

Supplement: Figure 4—source data 3. [file elife-84319-fig4-data3.zip › Figure 4ΓÇôSource Data 3/Figure 4D/32P/Replica 2.tif]

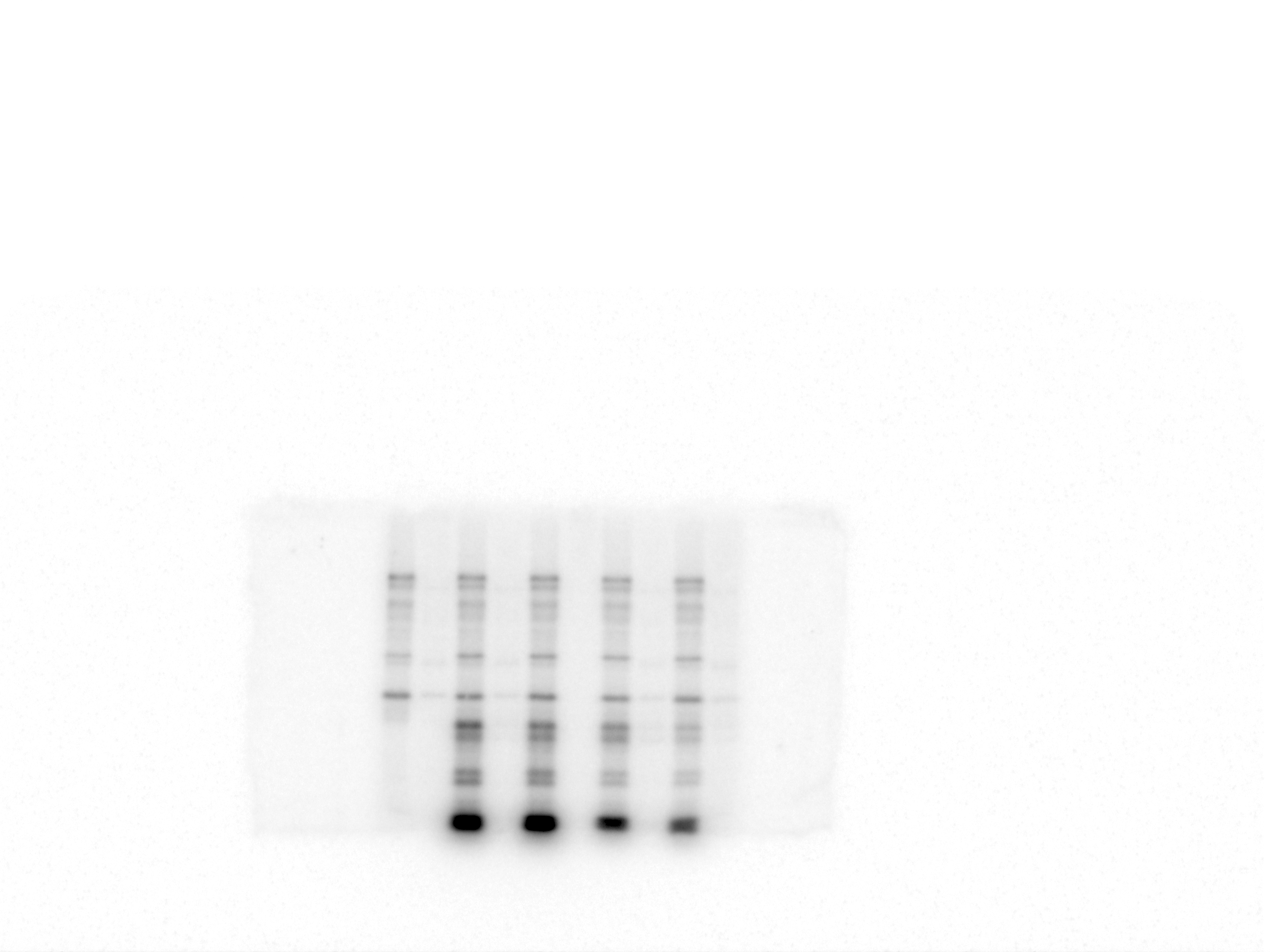

Supplement: Figure 4—source data 3. [file elife-84319-fig4-data3.zip › Figure 4ΓÇôSource Data 3/Figure 4D/32P/Replica 3.tif]

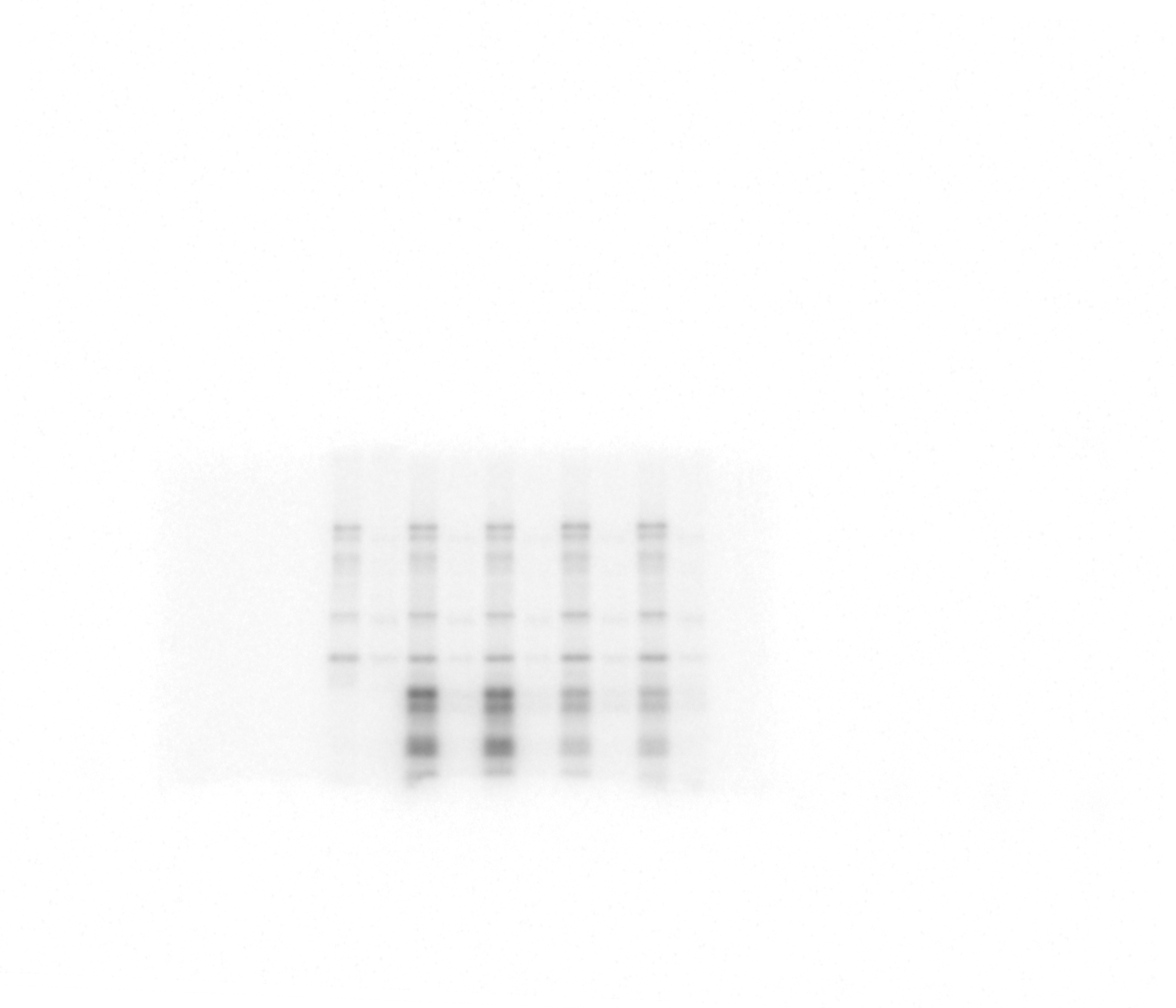

Supplement: Figure 4—source data 3. [file elife-84319-fig4-data3.zip › Figure 4ΓÇôSource Data 3/Figure 4D/32P/Replica 1.tif]

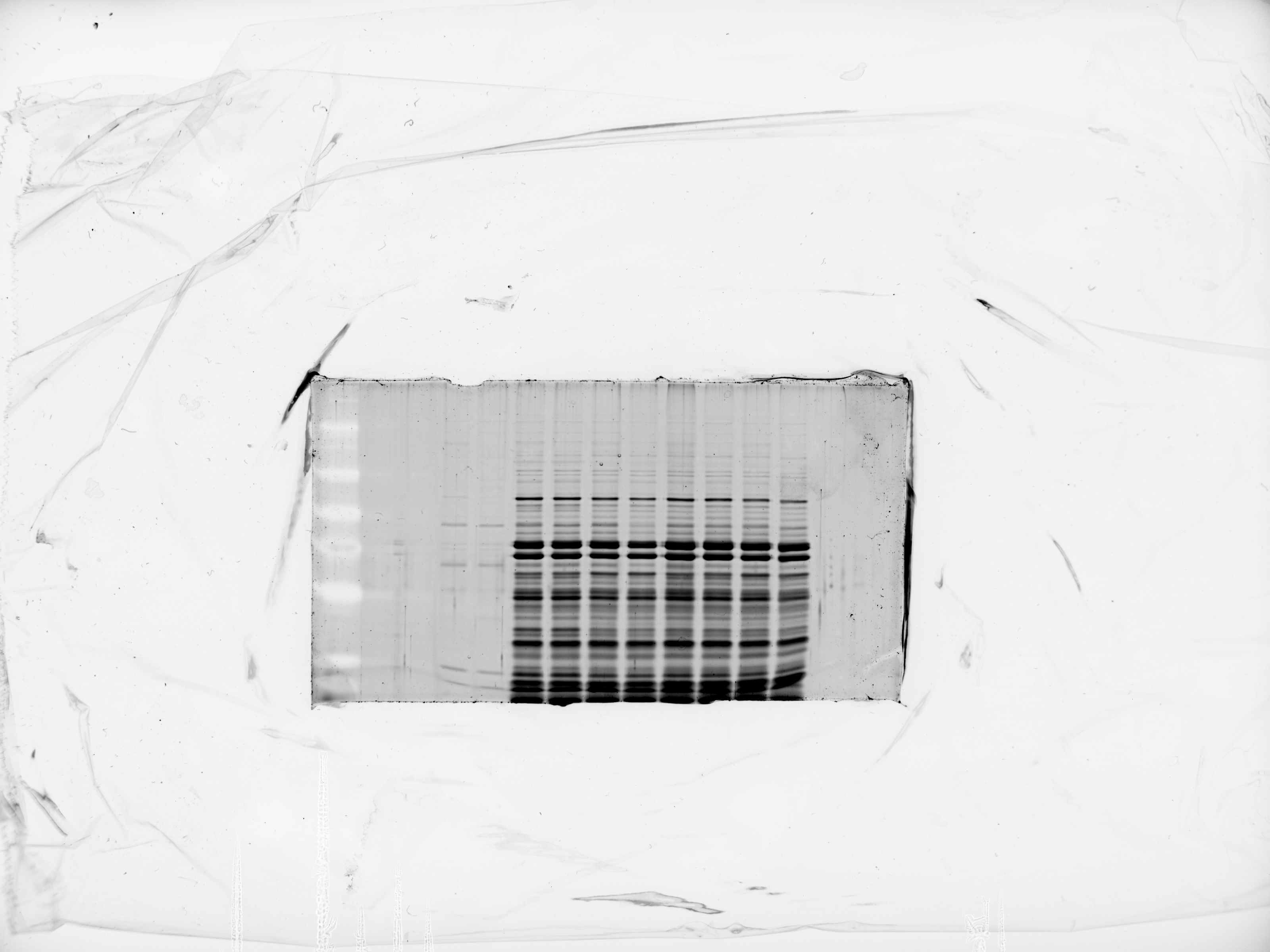

Supplement: Figure 4—source data 3. [file elife-84319-fig4-data3.zip › Figure 4ΓÇôSource Data 3/Figure 4D/Sypro Ruby/Replica 2.tif]

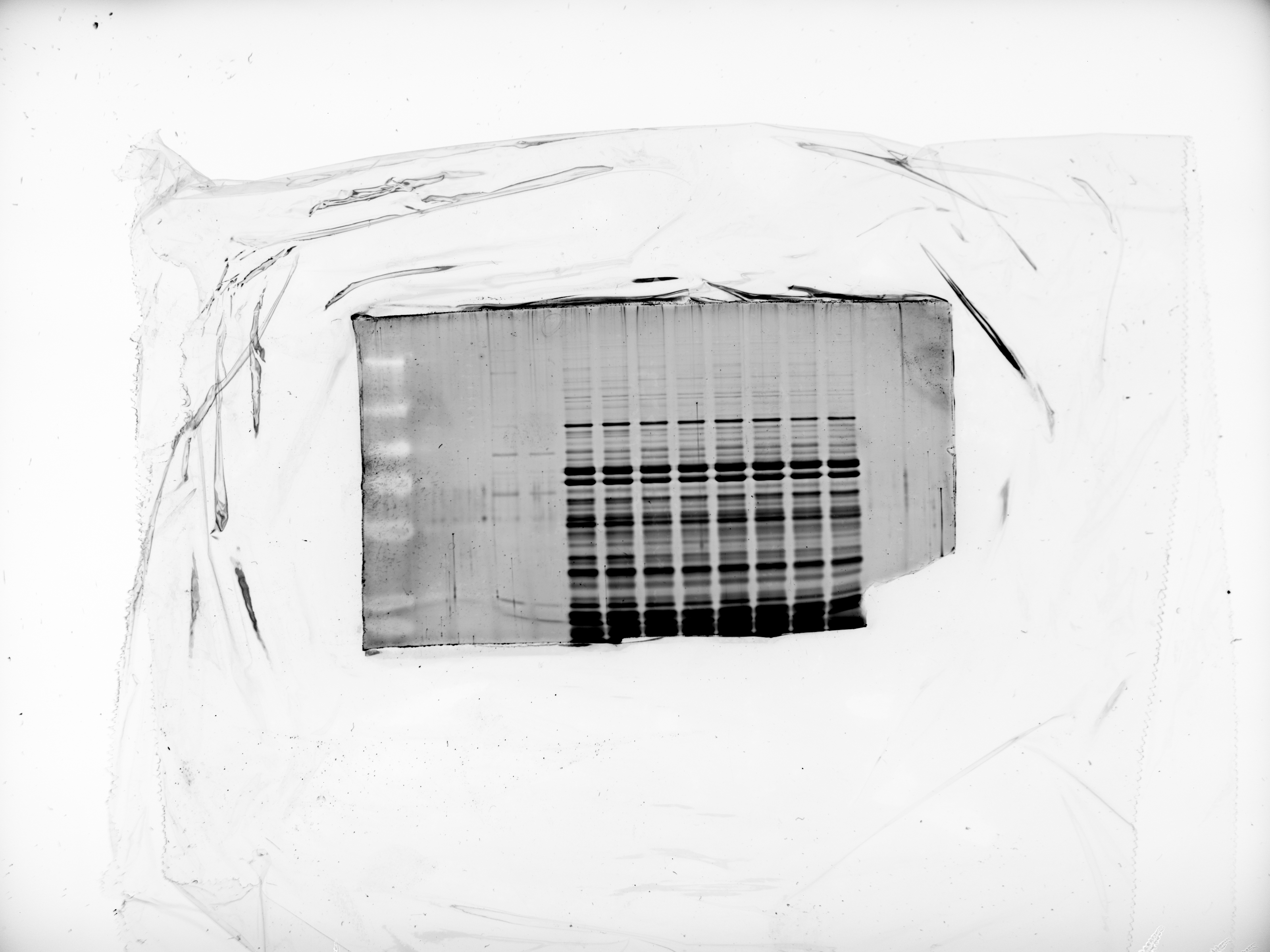

Supplement: Figure 4—source data 3. [file elife-84319-fig4-data3.zip › Figure 4ΓÇôSource Data 3/Figure 4D/Sypro Ruby/Replica 3.tif]

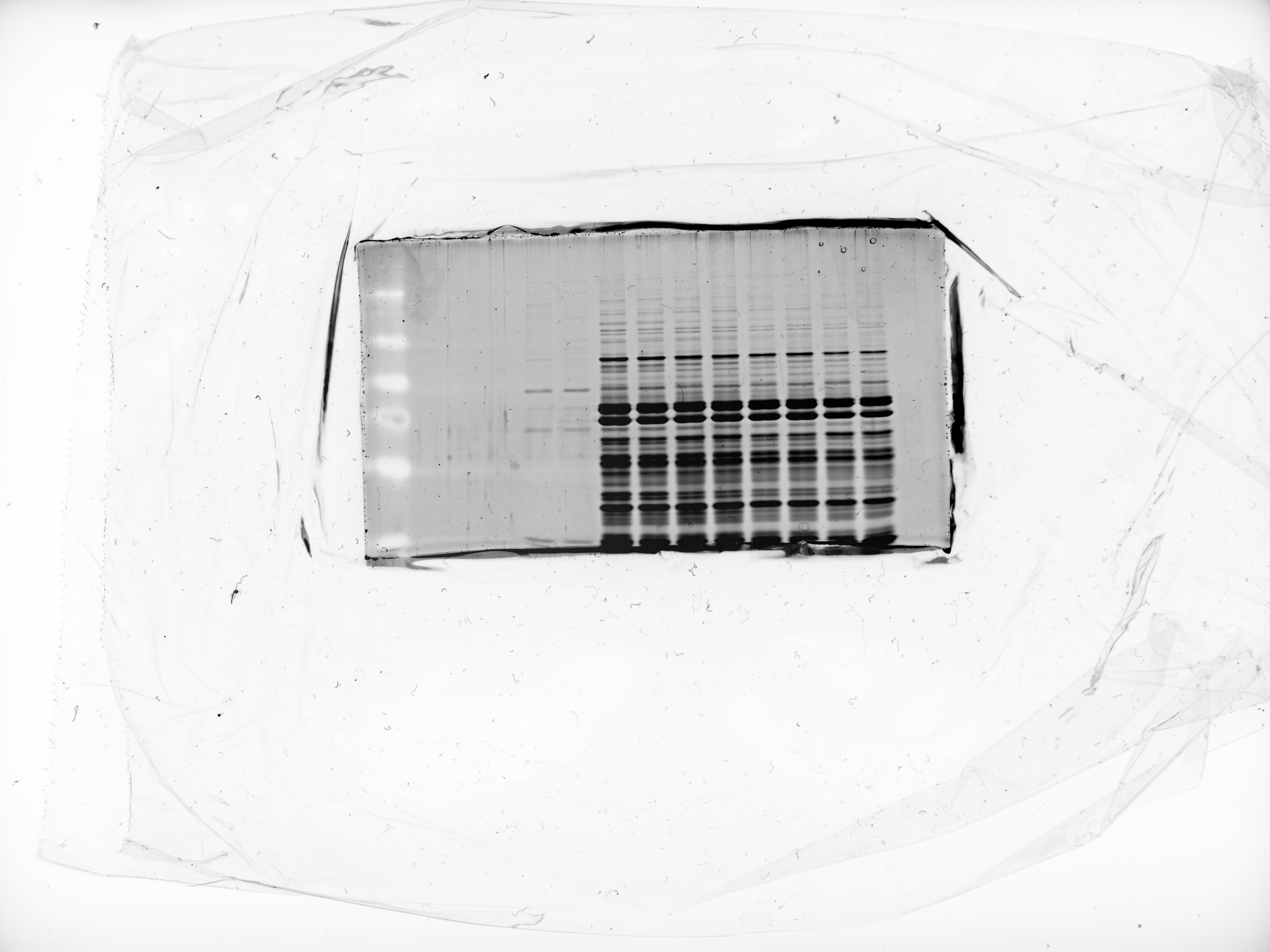

Supplement: Figure 4—source data 3. [file elife-84319-fig4-data3.zip › Figure 4ΓÇôSource Data 3/Figure 4D/Sypro Ruby/Replica 1.tif]

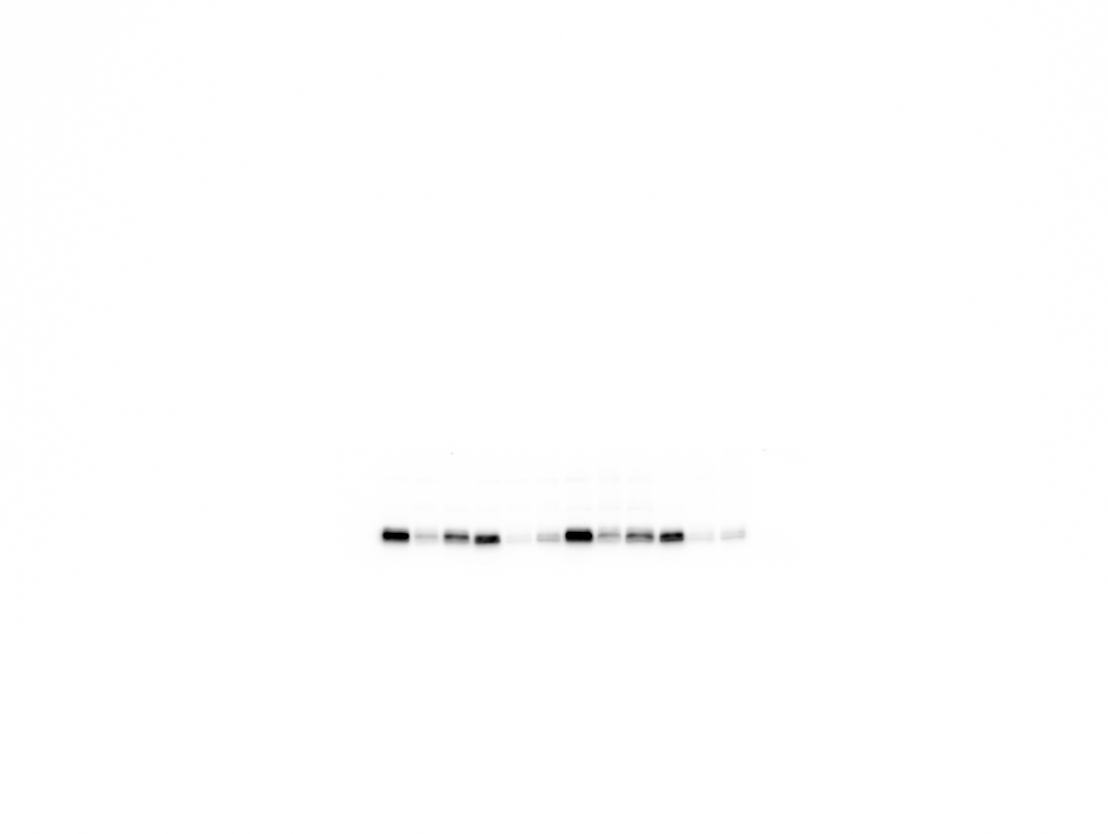

Supplement: Figure 4—source data 3. [file elife-84319-fig4-data3.zip › Figure 4ΓÇôSource Data 3/Figure 4F/Sch9-pThr737/Replica 4.tif]
