# Supplementary material for: Snf1/AMPK fine-tunes TORC1 signaling in response to glucose starvation
Source: eLife. 2023 Feb 7;12:e84319. doi: 10.7554/eLife.84319 (PMC9937656; doi:10.7554/eLife.84319)

Figure 1-figure supplement 1A

Loading order:

	WT			*snf1Δ*			*snf1^as^*		
	Exp	-C		Exp	-C		Exp	-C	
2NM-PP1		-	+		-	+		-	+

Anti-Sch9-pThr^{737}

Replica 1 (Data shown in Figure 1-figure supplement 1A)

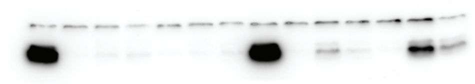

Replica 2
Replica 3

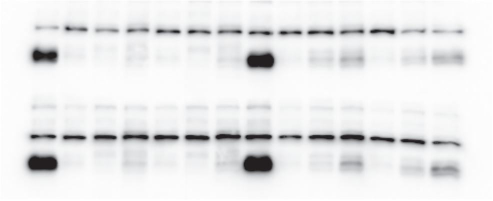

Anti-Sch9

Replica 1 (Data shown in Figure 1-figure supplement 1A)

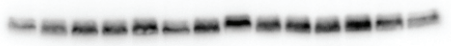

Replica 2
Replica 3

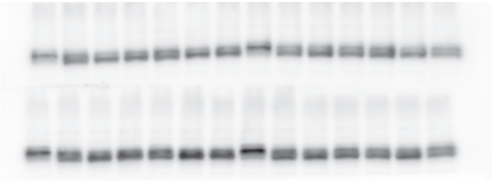

Anti-Snf1-pThr^{210}

Replica 1 (Data shown in Figure 1-figure supplement 1A)

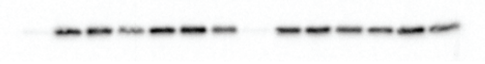

Replica 2
Replica 3

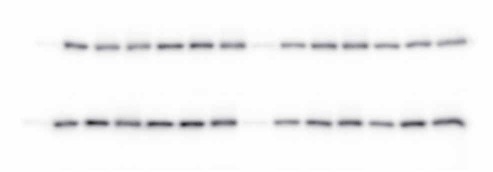

Anti-His_6

Replica 1 (Data shown in Figure 1-figure supplement 1A)

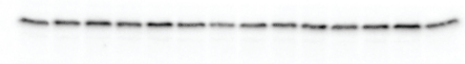

Replica 2
Replica 3

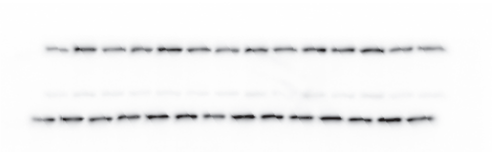

Anti-Acc1-pSer^{79}

Replica 1 (Data shown in Figure 1-figure supplement 1A)

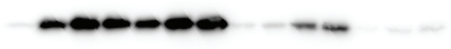

Replica 2

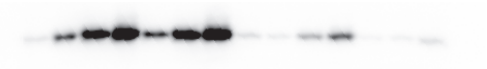

Replica 3

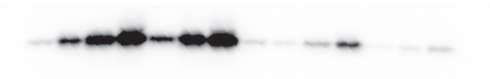

Anti-GFP

Replica 1 (Data shown in Figure 1-figure supplement 1A)

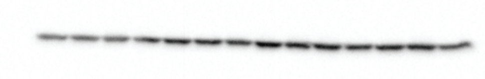

Replica 2

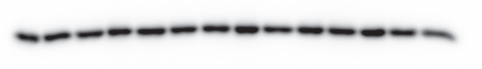

Replica 3

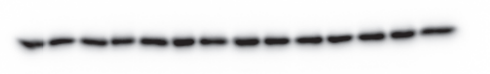

Figure 1-figure supplement 1B

Loading order: WT — Exp -N -C

Replica 1 (Data shown in Figure 1-figure supplement 1B)

Anti-Sch9-pThr[737]

Rep 1 Rep 2 Rep 3

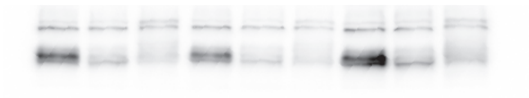

Anti-Sch9

Rep 1 Rep 2 Rep 3

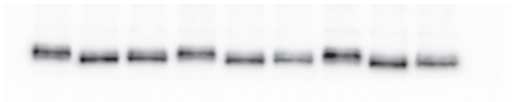

Anti-Snf1-pThr[210]

Rep 1 Rep 2 Rep 3

Anti-His_6

Rep 1 Rep 2 Rep 3

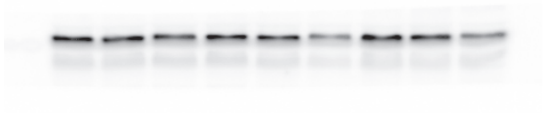

Anti-Acc1-pSer[79]

Rep 1 Rep 2 Rep 3

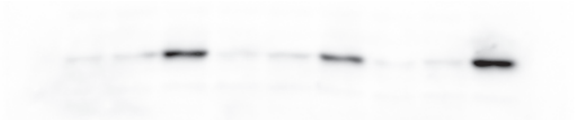

Anti-GFP

Rep 1 Rep 2 Rep 3

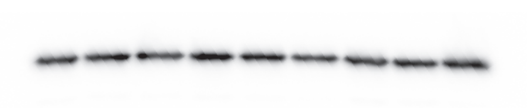

Supplement: Figure 1—figure supplement 1—source data 2. [file elife-84319-fig1-figsupp1-data2.pdf]
