# Supplementary material for: Snf1/AMPK fine-tunes TORC1 signaling in response to glucose starvation
Source: eLife. 2023 Feb 7;12:e84319. doi: 10.7554/eLife.84319 (PMC9937656; doi:10.7554/eLife.84319)

Figure 2A

Loading order:

			2% Glc									
	Exp	-N -C	DMSO					2NM-PP1				
min		20	1	2	5	10	15	1	2	5	10	15

Anti-Sch9-pThr[737]

Replica 1

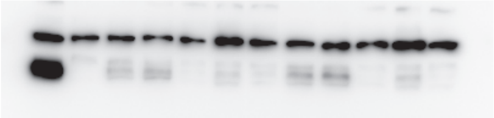

Replica 2

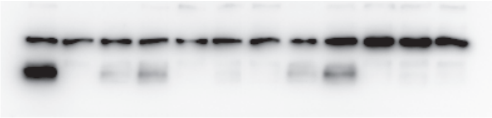

Replica 3 (Data shown in Figure 2A)

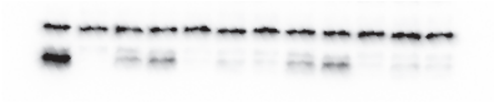

Anti-Sch9

Replica 1
Replica 2

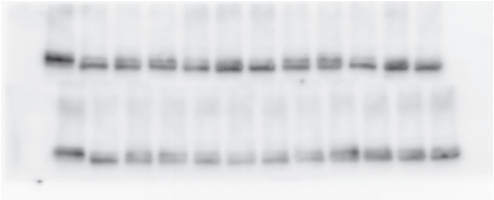

Replica 3 (Data shown in Figure 2A)

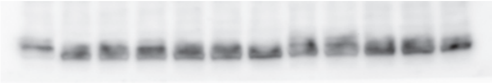

Anti-Snf1-pThr[210]

Replica 1
Replica 2

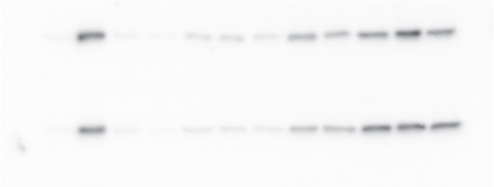

Replica 3 (Data shown in Figure 2A)

Anti-His_6

Replica 1
Replica 2

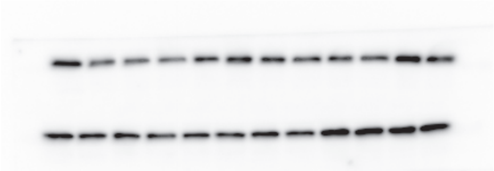

Replica 3 (Data shown in Figure 2A)

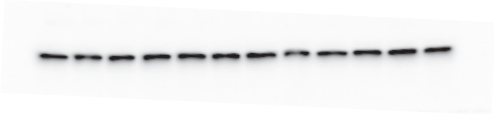

Figure 2C

Loading order:

2% Glc + 3.3 mM Gln

	Exp	-N -C	DMSO					2NM-PP1				
min		20	1	2	5	10	15	1	2	5	10	15

Anti-Sch9-pThr^{737}

Replica 1

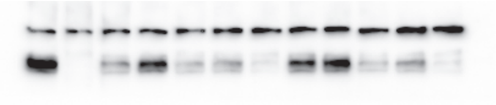

Replica 2
Replica 3 (Data shown in Figure 2A)

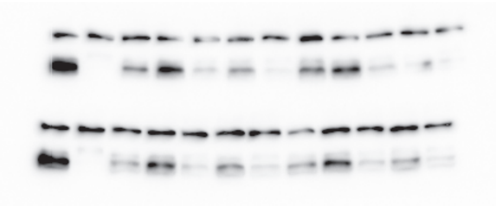

Anti-Sch9

Replica 1

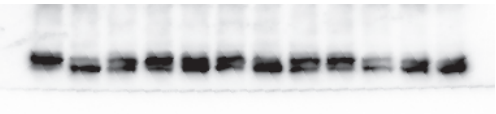

Replica 2
Replica 3 (Data shown in Figure 2A)

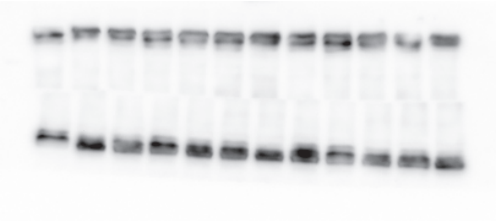

Anti-Snf1-pThr^{210}

Replica 1

Replica 2
Replica 3 (Data shown in Figure 2A)

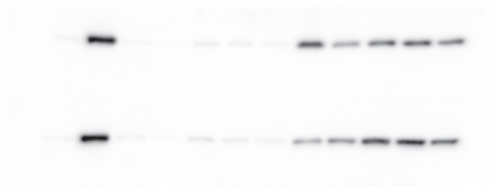

Anti-His$_6$

Replica 1

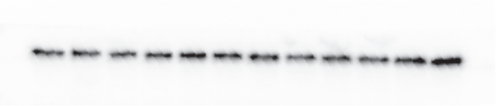

Replica 2
Replica 3 (Data shown in Figure 2A)

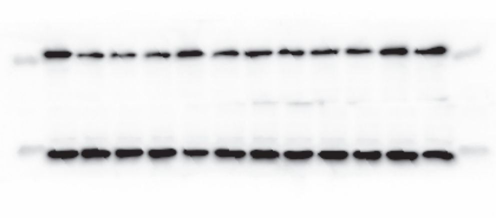

Figure 2E

Loading order:

3.3 mM Gln

Exp | -N -C | DMSO | 2NM-PP1

min | 20 | 1 2 5 10 15 | 1 2 5 10 15

Anti-Sch9-pThr^{737}

Replica 1

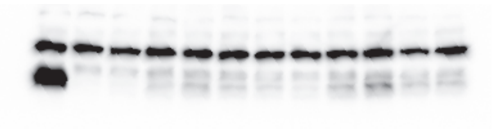

Replica 2

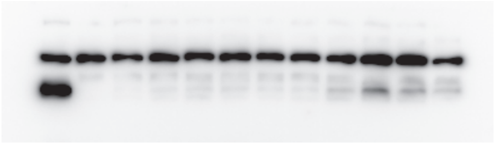

Replica 3 (Data shown in Figure 2A)

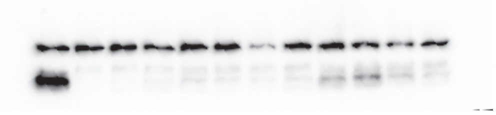

Anti-Sch9

Replica 1

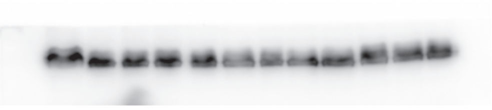

Replica 2

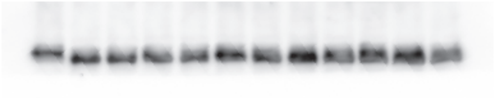

Replica 3 (Data shown in Figure 2A)

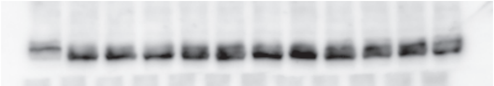

Anti-Snf1-pThr^{210}

Replica 1

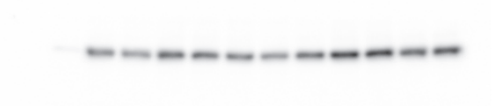

Replica 2

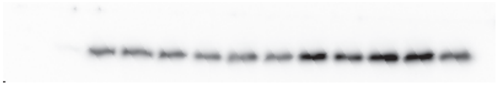

Replica 3 (Data shown in Figure 2A)

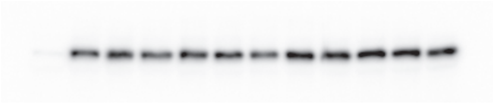

Anti-His_6

Replica 1

Replica 2

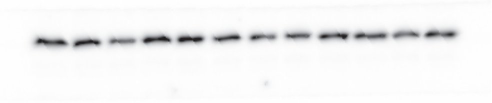

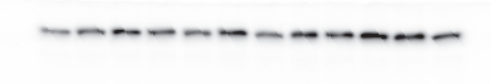

Replica 3 (Data shown in Figure 2A)

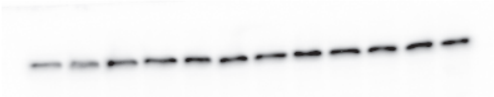

Supplement: Figure 2—source data 2. [file elife-84319-fig2-data2.pdf]
