# Supplementary material for: Snf1/AMPK fine-tunes TORC1 signaling in response to glucose starvation
Source: eLife. 2023 Feb 7;12:e84319. doi: 10.7554/eLife.84319 (PMC9937656; doi:10.7554/eLife.84319)

Figure 3-figure supplement 1C

Loading order:

	*snf1^as^*			*snf1^as^ lst4Δ*		
	Exp	-C		Exp	-C	
2NM-PP1		-	+		-	+

Anti-Sch9-pThr^{737}

Replica 1
Replica 2 (Data shown in Figure 3-figure supplement 1C)
Replica 3

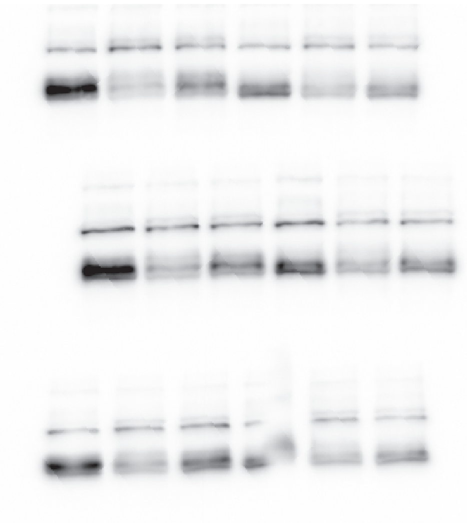

Anti-Sch9

Replica 1
Replica 2 (Data shown in Figure 3-figure supplement 1C)
Replica 3

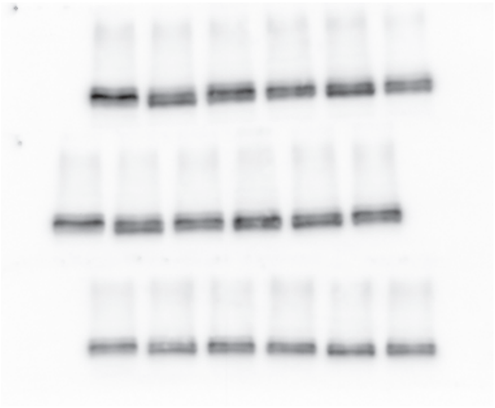

Anti-Snf1-pThr^{210}

Replica 1
Replica 2 (Data shown in Figure 3-figure supplement 1C)
Replica 3

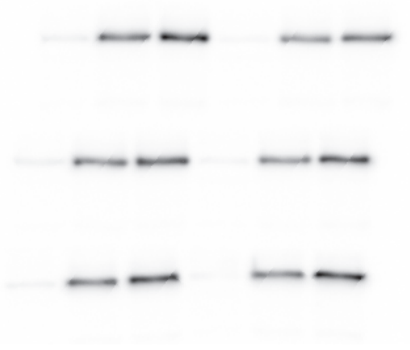

Anti-His$_6$

Replica 1
Replica 2 (Data shown in Figure 3-figure supplement 1C)
Replica 3

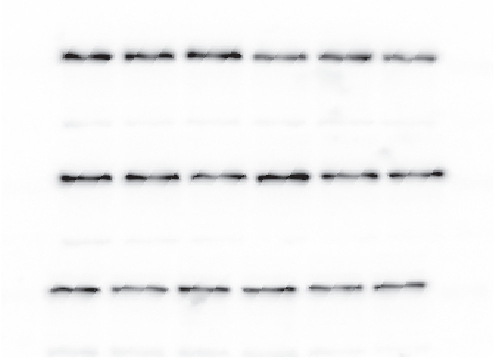

Supplement: Figure 3—figure supplement 1—source data 2. [file elife-84319-fig3-figsupp1-data2.pdf]
