# Supplementary material for: Snf1/AMPK fine-tunes TORC1 signaling in response to glucose starvation
Source: eLife. 2023 Feb 7;12:e84319. doi: 10.7554/eLife.84319 (PMC9937656; doi:10.7554/eLife.84319)

Figure 4D

Loading order:

His$_6$-Pib2$^{221\text{-}635}$	-		WT		S268A		S309A		SASA	
Snf1	WT	TA	WT	TA	WT	TA	WT	TA	WT	TA

^{32}P Autoradiography

Replica 1

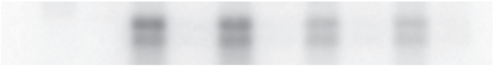

Replica 2

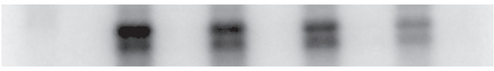

Replica 3

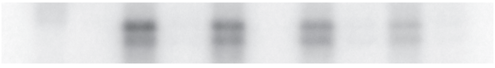

Sypro Ruby - Snf1 variants

Replica 1

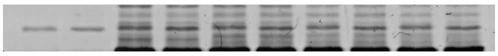

Replica 2

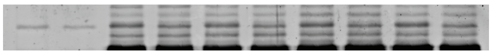

Replica 3

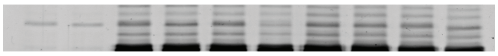

Sypro Ruby - Pib2 variants

Replica 1

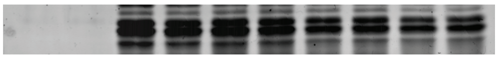

Replica 2

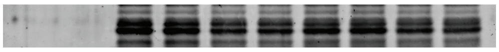

Replica 3

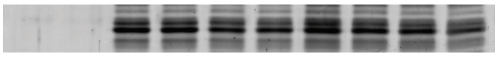

Figure 4F

Loading order:

	*snf1^as^*			*snf1^as^ pib2Δ*			*snf1^as^ pib2^SASA^*			*snf1^as^ pib2^SESE^*		
	Exp	-C		Exp	-C		Exp	-C		Exp	-C	
2NM-PP1		-	+		-	+		-	+		-	+

Anti-Sch9-pThr^{737}

Replica 1
Replica 2 (Data shown in Figure 4F)
Replica 3

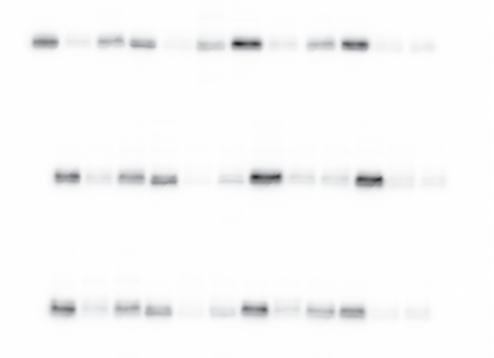

Replica 4

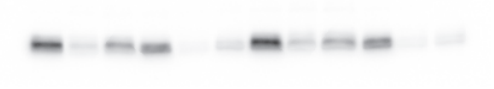

Anti-Sch9

Replica 1
Replica 2 (Data shown in Figure 4F)
Replica 3

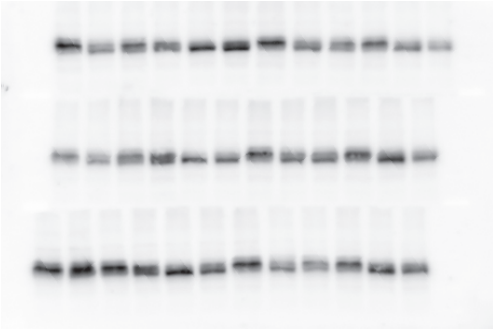

Replica 4

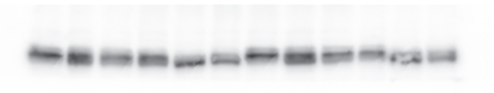

Anti-Snf1-pThr^{210}

Replica 1
Replica 2 (Data shown in Figure 4F)
Replica 3

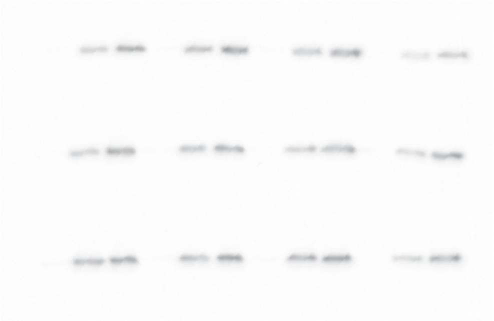

Replica 4

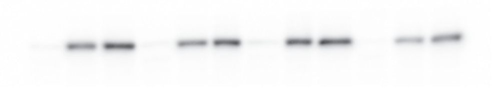

Anti-His_6

Replica 1
Replica 2 (Data shown in Figure 4F)
Replica 3

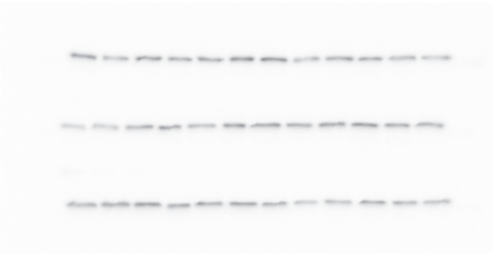

Replica 4

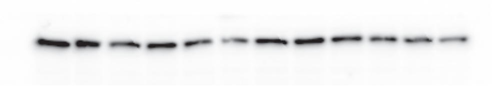

Figure 4H

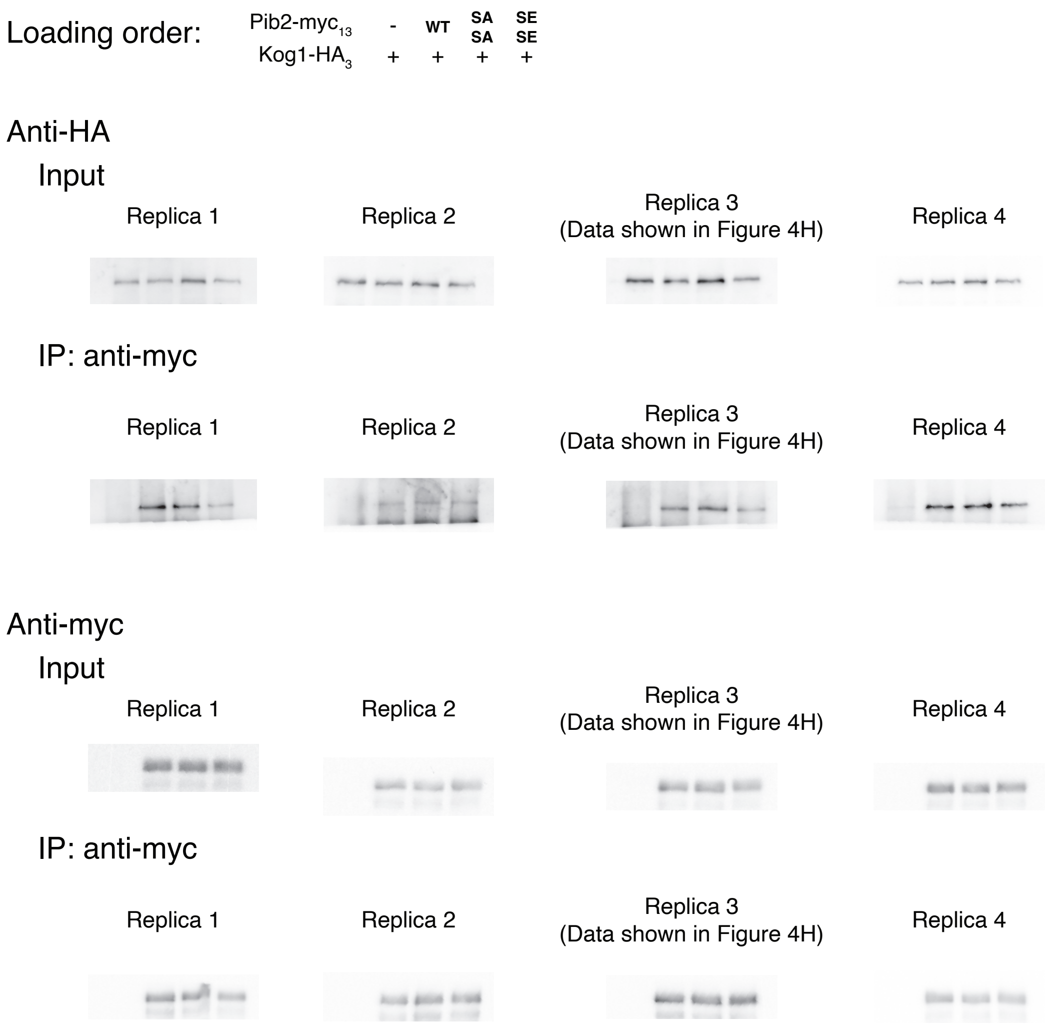
Loading order:
Pib2-myc$_{13}$ - WT SA SA SE SE
Kog1-HA$_3$ + + + +
Anti-HA
Input
Replica 1
Replica 2
Replica 3 (Data shown in Figure 4H)
Replica 4
IP: anti-myc
Replica 1
Replica 2
Replica 3 (Data shown in Figure 4H)
Replica 4
Anti-myc
Input
Replica 1
Replica 2
Replica 3 (Data shown in Figure 4H)
Replica 4
IP: anti-myc
Replica 1
Replica 2
Replica 3 (Data shown in Figure 4H)
Replica 4

Figure 4I

Loading order:

Pib2-myc$_{13}$ variant

	snf1^{as}	
WT	SA SA	SE SE

Anti-myc

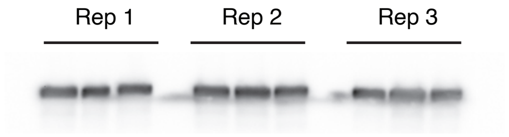

Anti-Adh1

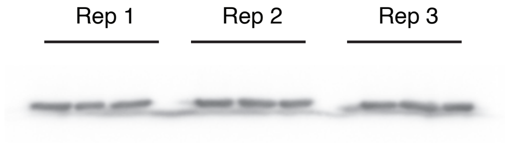

Supplement: Figure 4—source data 2. [file elife-84319-fig4-data2.pdf]
