# Supplementary figures and images for: Snf1/AMPK fine-tunes TORC1 signaling in response to glucose starvation (part 2 of 2)
Source: eLife. 2023 Feb 7;12:e84319. doi: 10.7554/eLife.84319 (PMC9937656; doi:10.7554/eLife.84319)

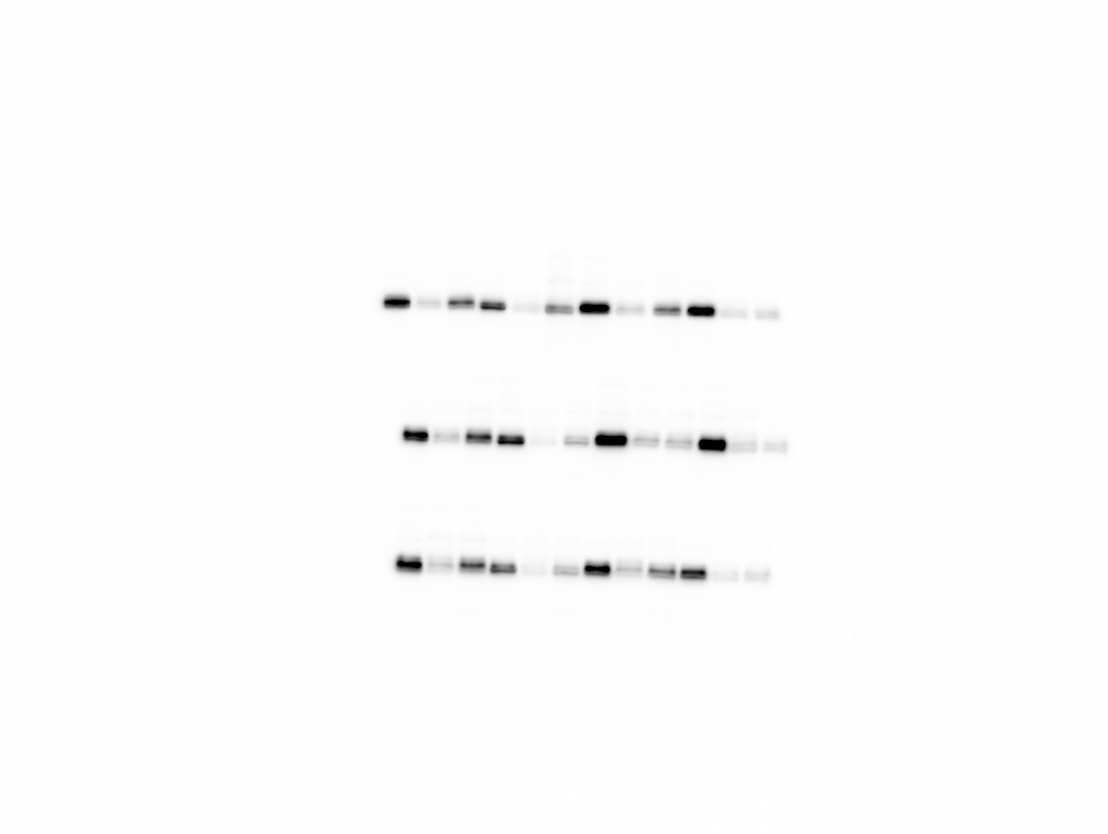

Supplement: Figure 4—source data 3. [file elife-84319-fig4-data3.zip › Figure 4ΓÇôSource Data 3/Figure 4F/Sch9-pThr737/Replica 1_2_3.tif]

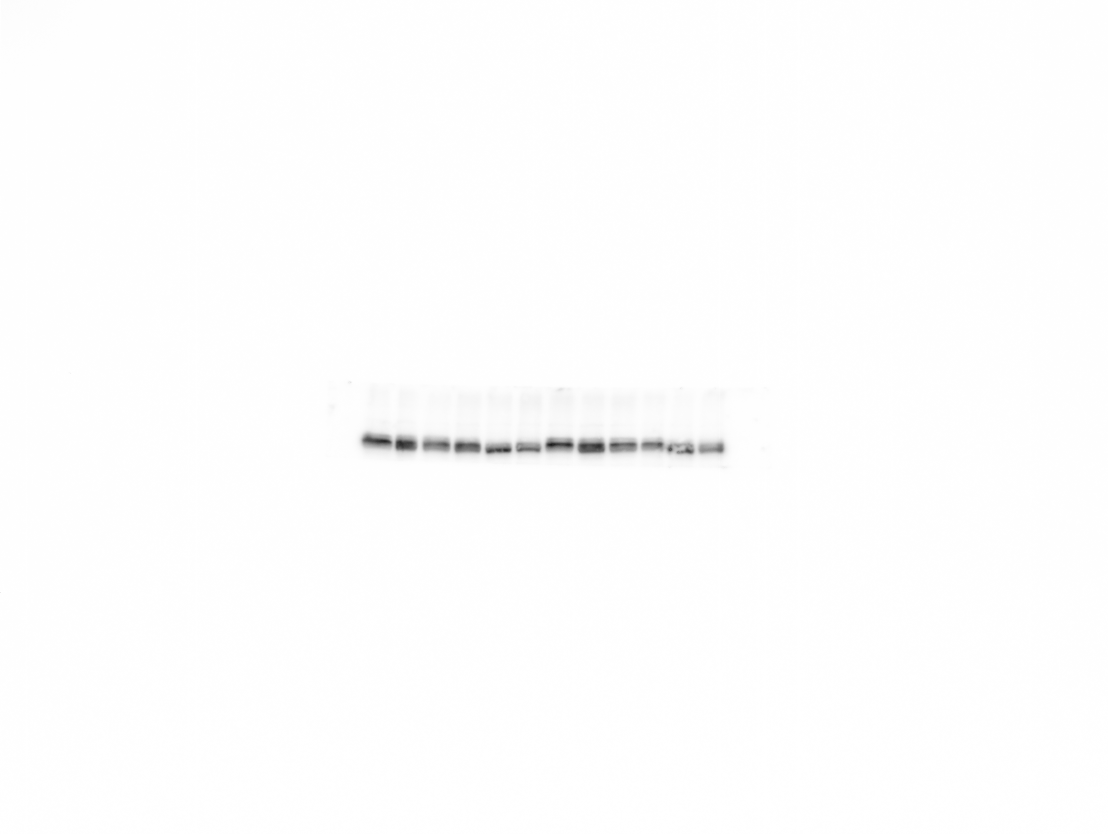

Supplement: Figure 4—source data 3. [file elife-84319-fig4-data3.zip › Figure 4ΓÇôSource Data 3/Figure 4F/Sch9/Replica 4.tif]

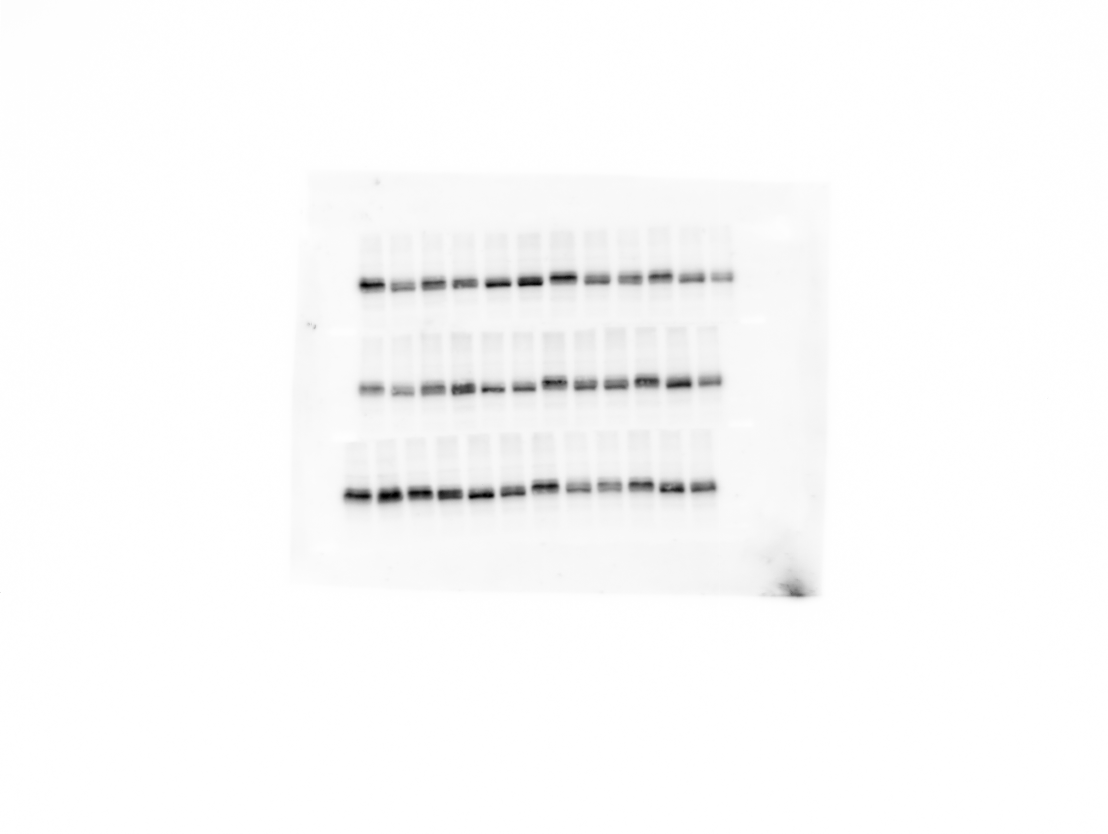

Supplement: Figure 4—source data 3. [file elife-84319-fig4-data3.zip › Figure 4ΓÇôSource Data 3/Figure 4F/Sch9/Replica 1_2_3.tif]

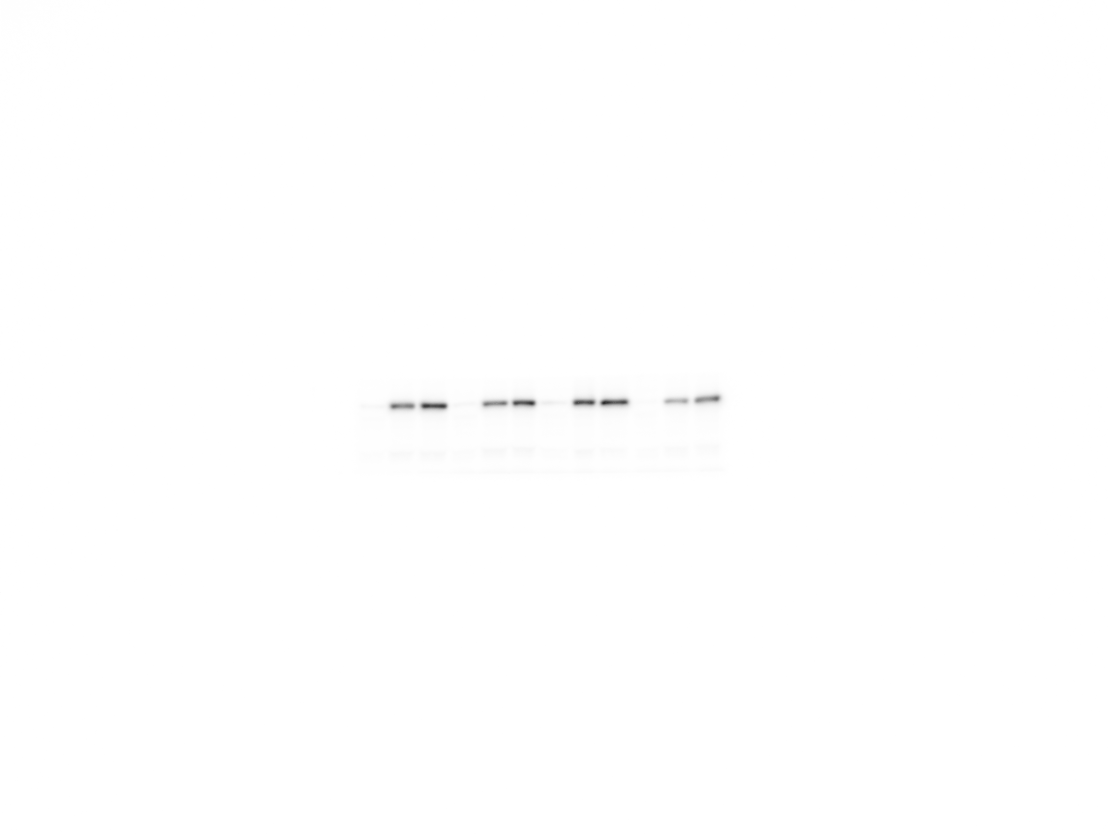

Supplement: Figure 4—source data 3. [file elife-84319-fig4-data3.zip › Figure 4ΓÇôSource Data 3/Figure 4F/Snf1-pThr210/Replica 4.tif]

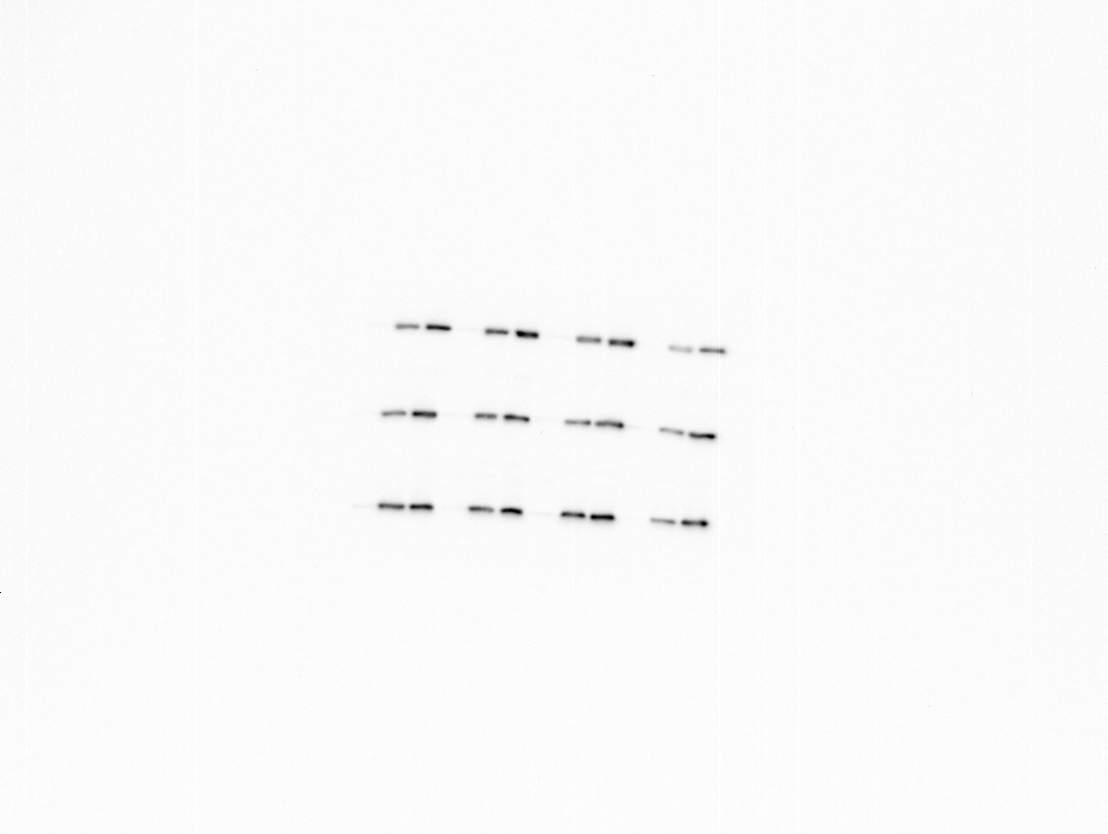

Supplement: Figure 4—source data 3. [file elife-84319-fig4-data3.zip › Figure 4ΓÇôSource Data 3/Figure 4F/Snf1-pThr210/Replica 1_2_3.tif]

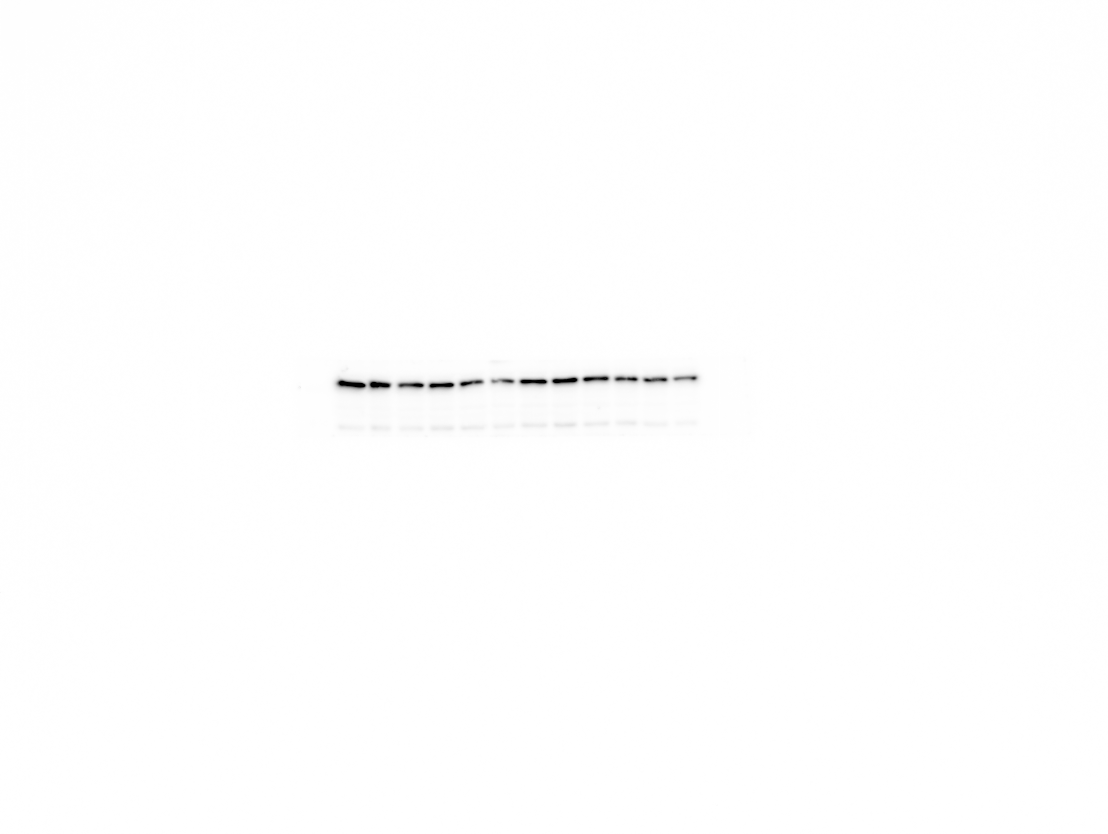

Supplement: Figure 4—source data 3. [file elife-84319-fig4-data3.zip › Figure 4ΓÇôSource Data 3/Figure 4F/His6/Replica 4.tif]

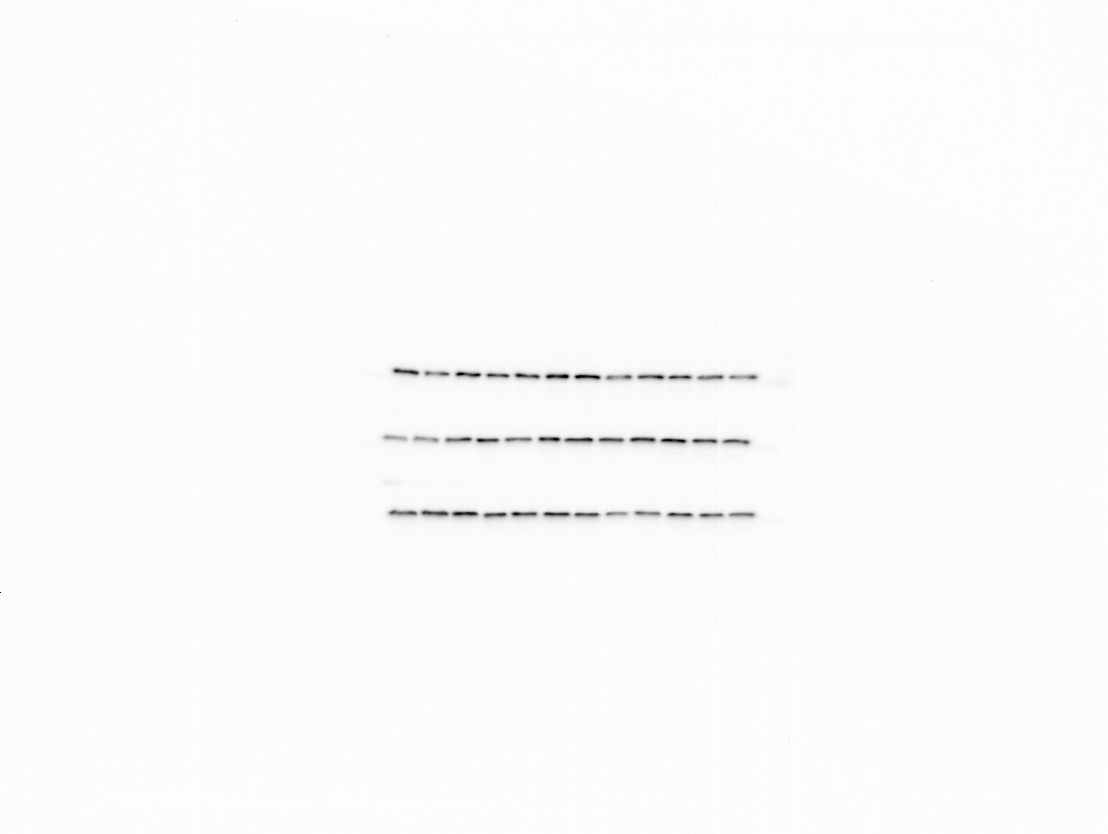

Supplement: Figure 4—source data 3. [file elife-84319-fig4-data3.zip › Figure 4ΓÇôSource Data 3/Figure 4F/His6/Replica 1_2_3.tif]

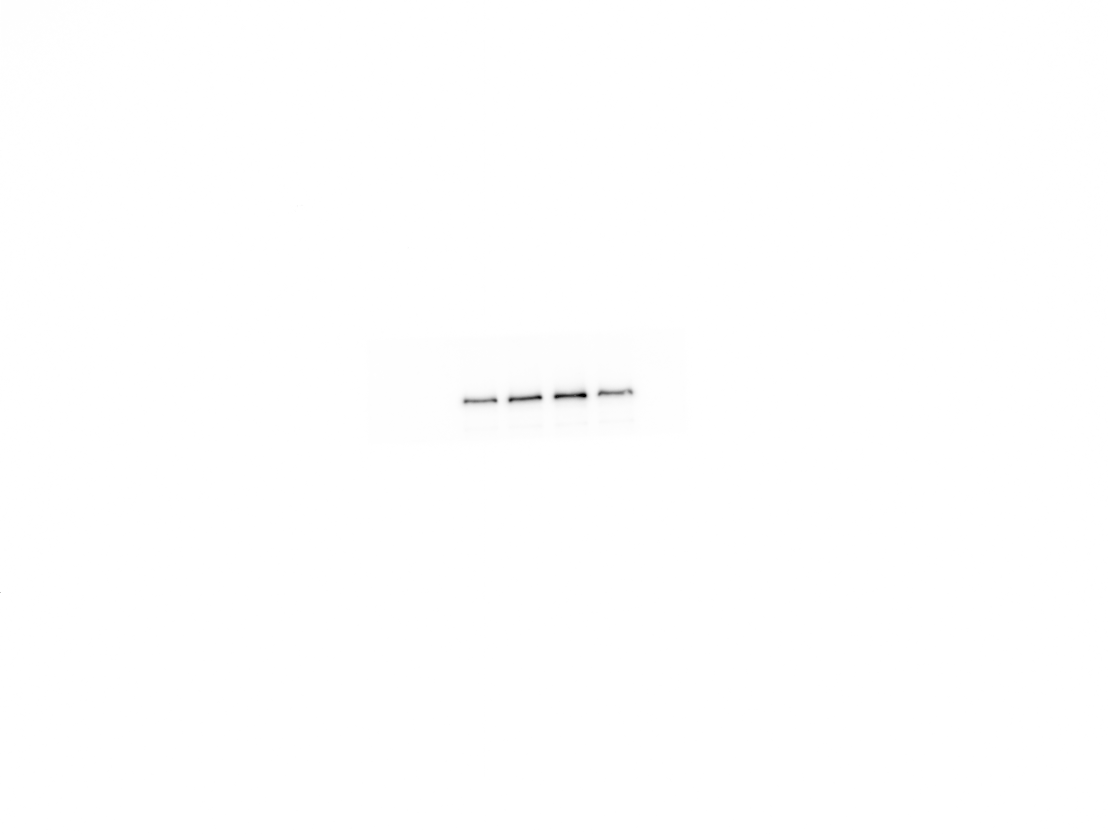

Supplement: Figure 4—source data 3. [file elife-84319-fig4-data3.zip › Figure 4ΓÇôSource Data 3/Figure 4H/Input HA/Replica 4.tif]

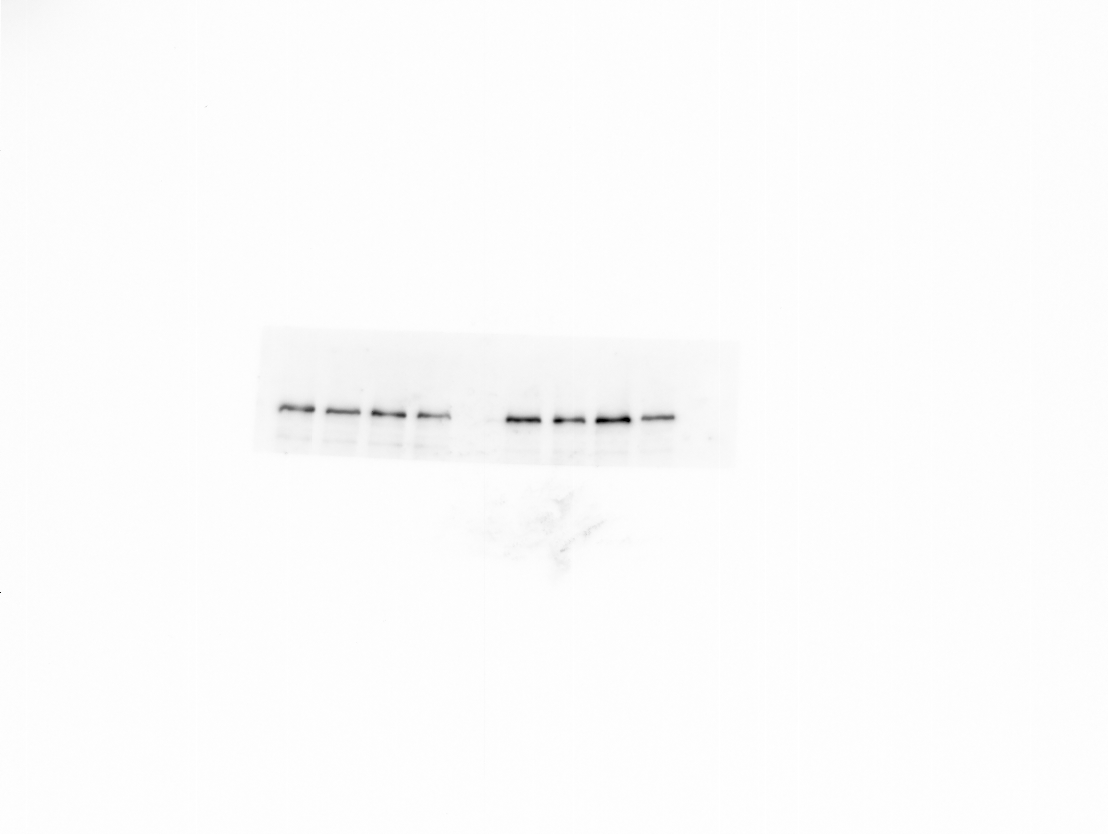

Supplement: Figure 4—source data 3. [file elife-84319-fig4-data3.zip › Figure 4ΓÇôSource Data 3/Figure 4H/Input HA/Replica 2_3.tif]

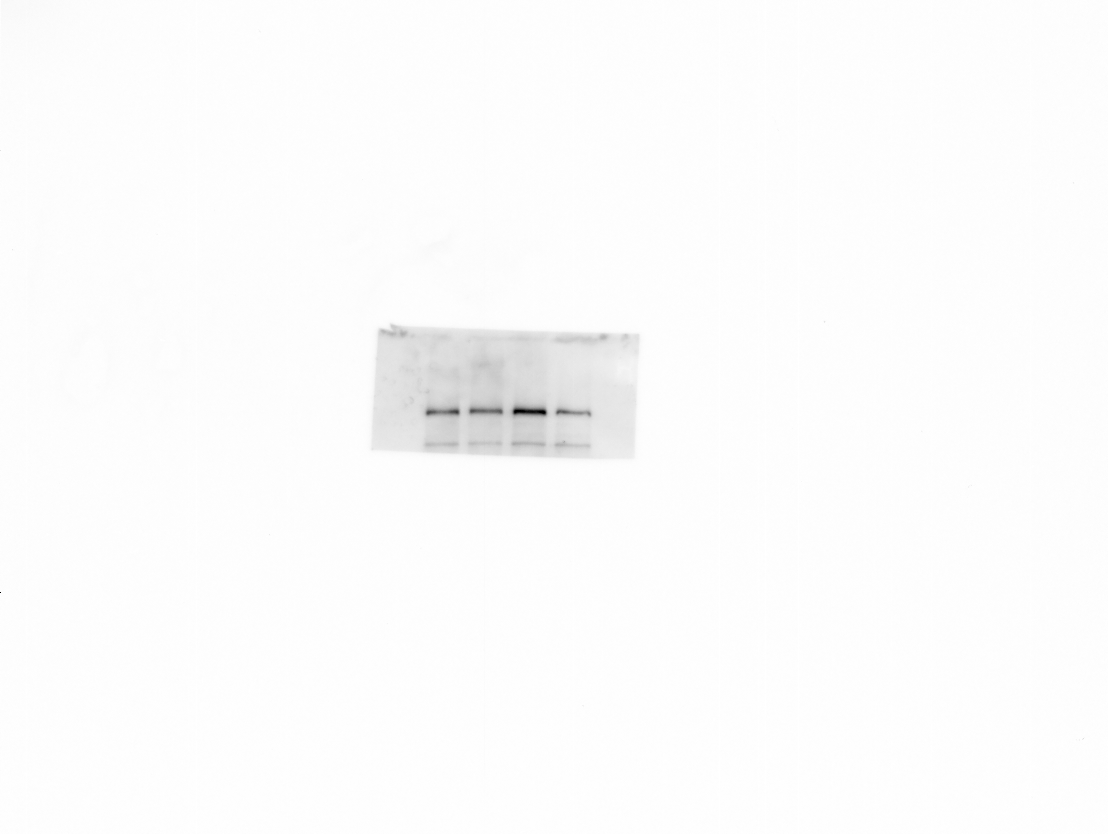

Supplement: Figure 4—source data 3. [file elife-84319-fig4-data3.zip › Figure 4ΓÇôSource Data 3/Figure 4H/Input HA/Replica 1.tif]

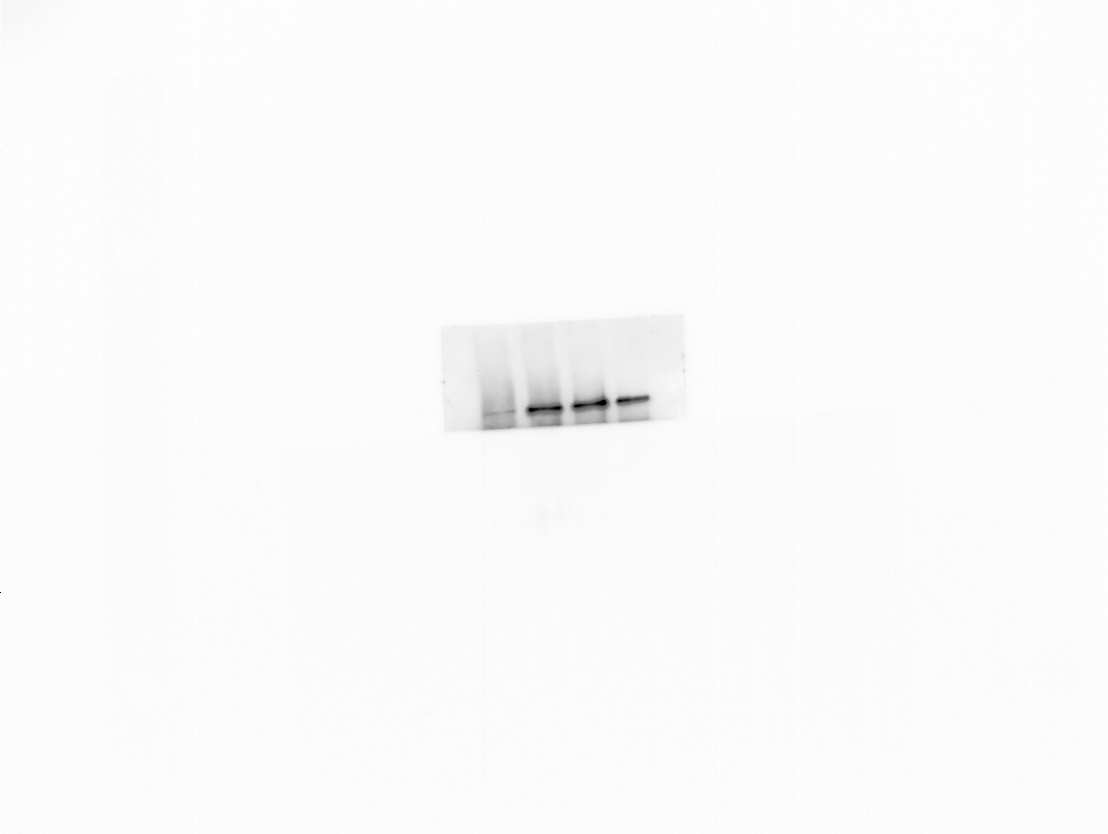

Supplement: Figure 4—source data 3. [file elife-84319-fig4-data3.zip › Figure 4ΓÇôSource Data 3/Figure 4H/IP HA/Replica 4.tif]

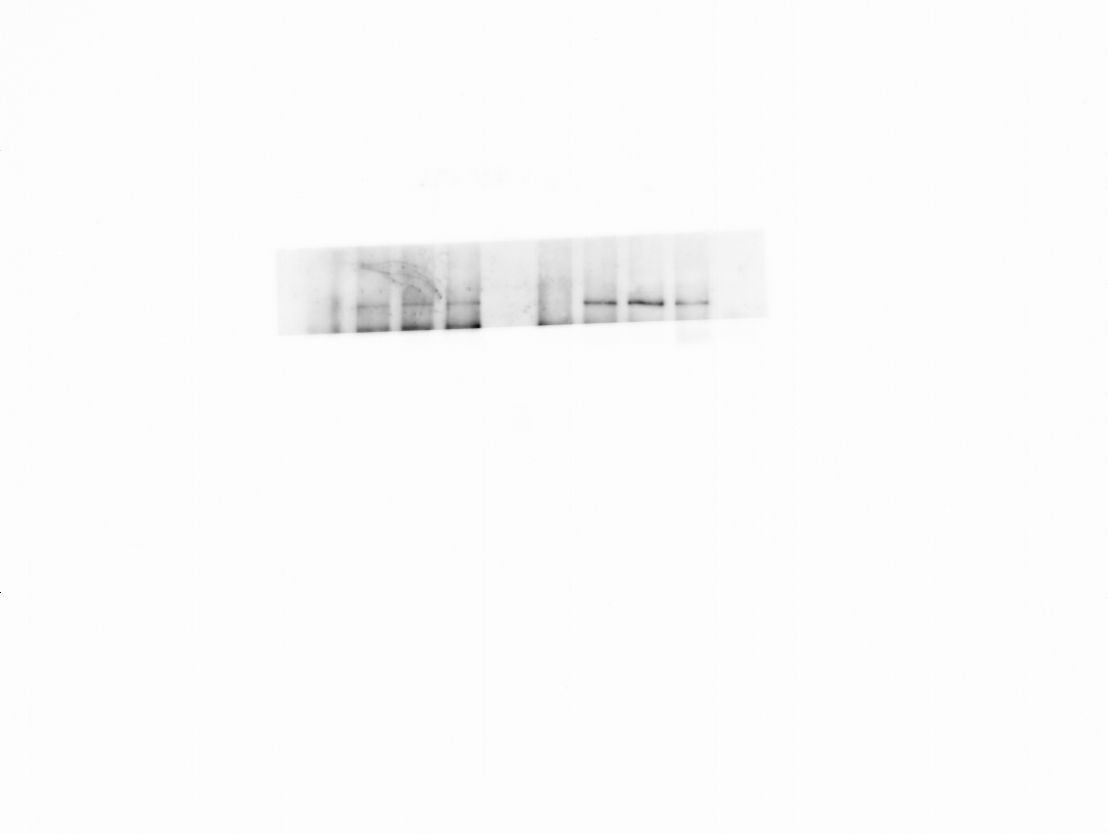

Supplement: Figure 4—source data 3. [file elife-84319-fig4-data3.zip › Figure 4ΓÇôSource Data 3/Figure 4H/IP HA/Replica 2_3.tif]

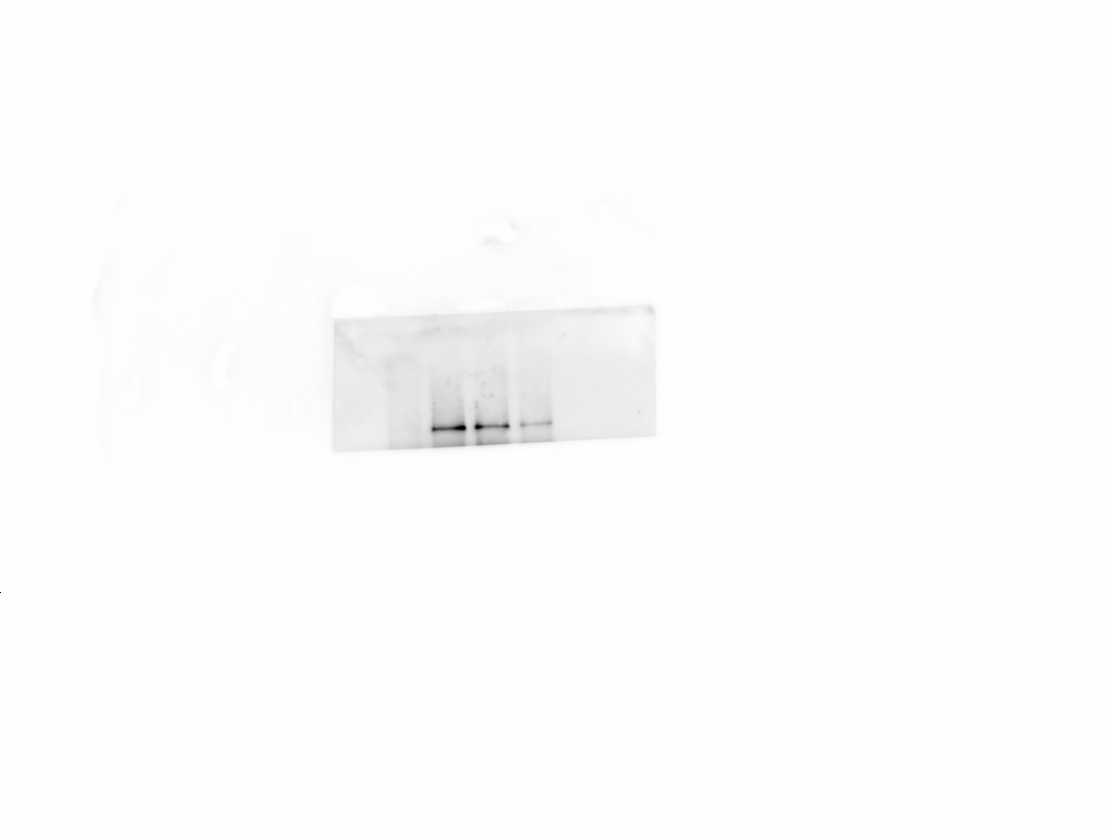

Supplement: Figure 4—source data 3. [file elife-84319-fig4-data3.zip › Figure 4ΓÇôSource Data 3/Figure 4H/IP HA/Replica 1.tif]

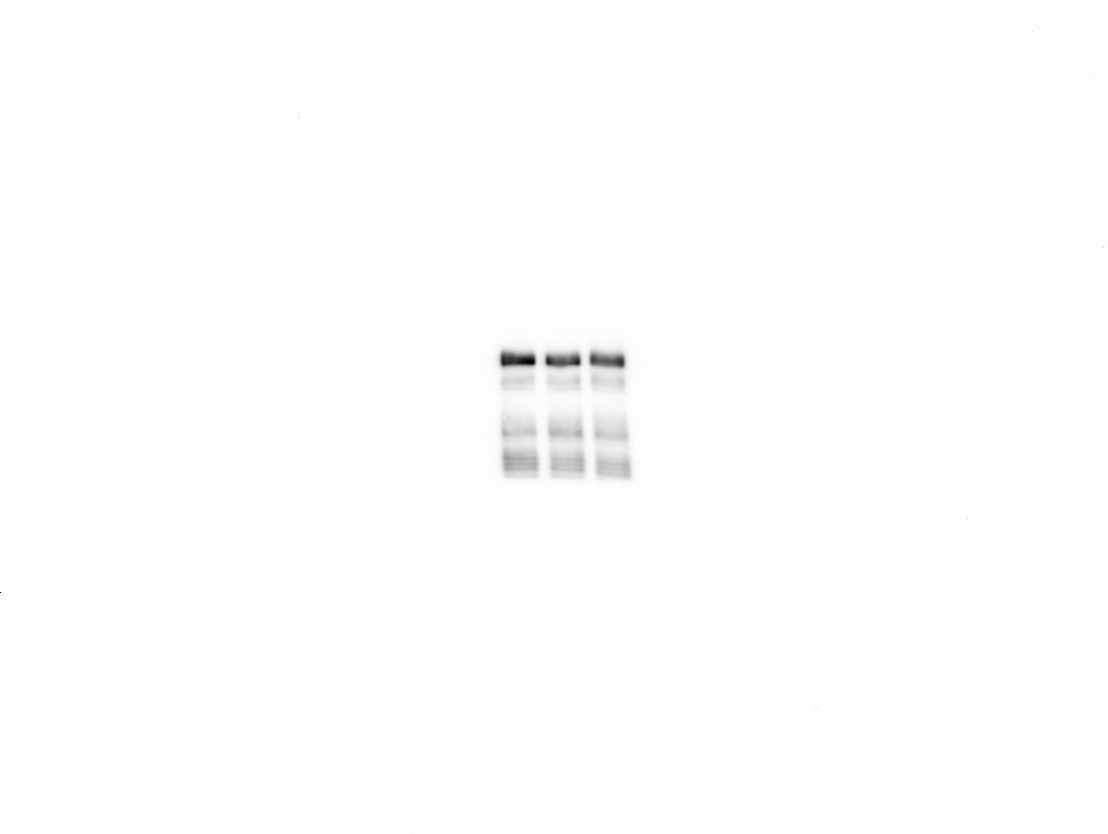

Supplement: Figure 4—source data 3. [file elife-84319-fig4-data3.zip › Figure 4ΓÇôSource Data 3/Figure 4H/IP myc/Replica 4.tif]

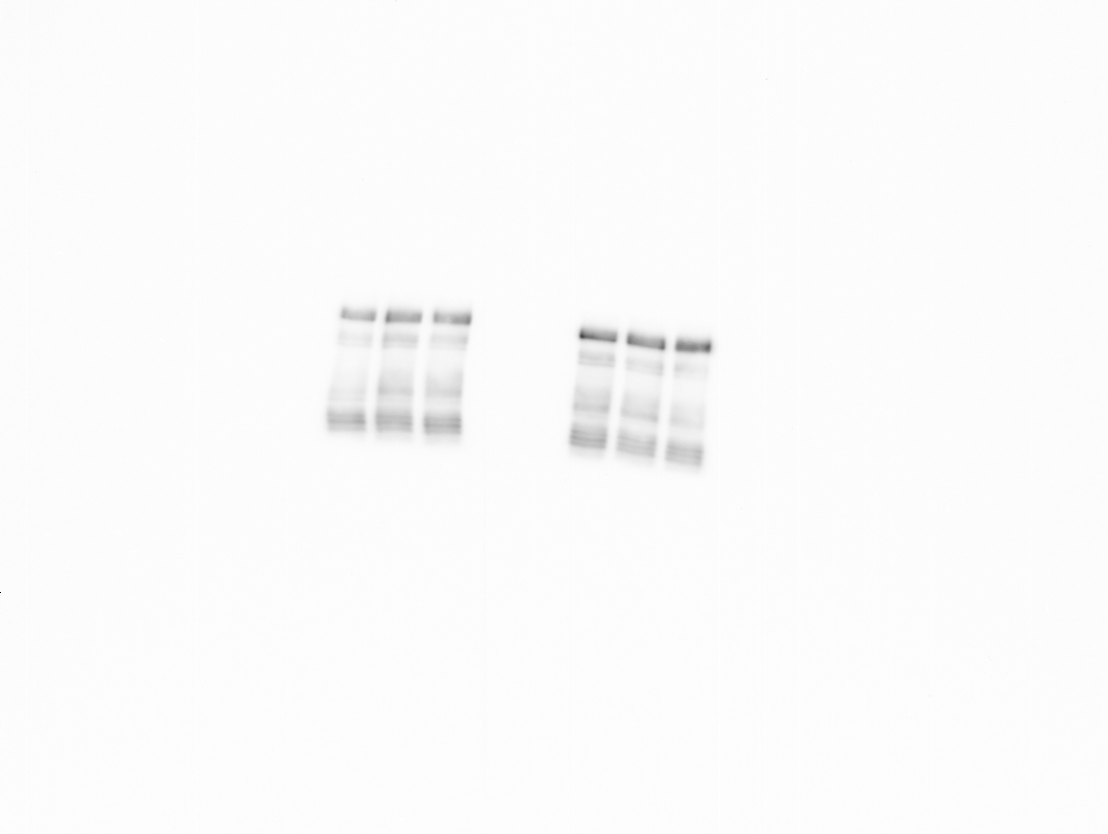

Supplement: Figure 4—source data 3. [file elife-84319-fig4-data3.zip › Figure 4ΓÇôSource Data 3/Figure 4H/IP myc/Replica 2_3.tif]

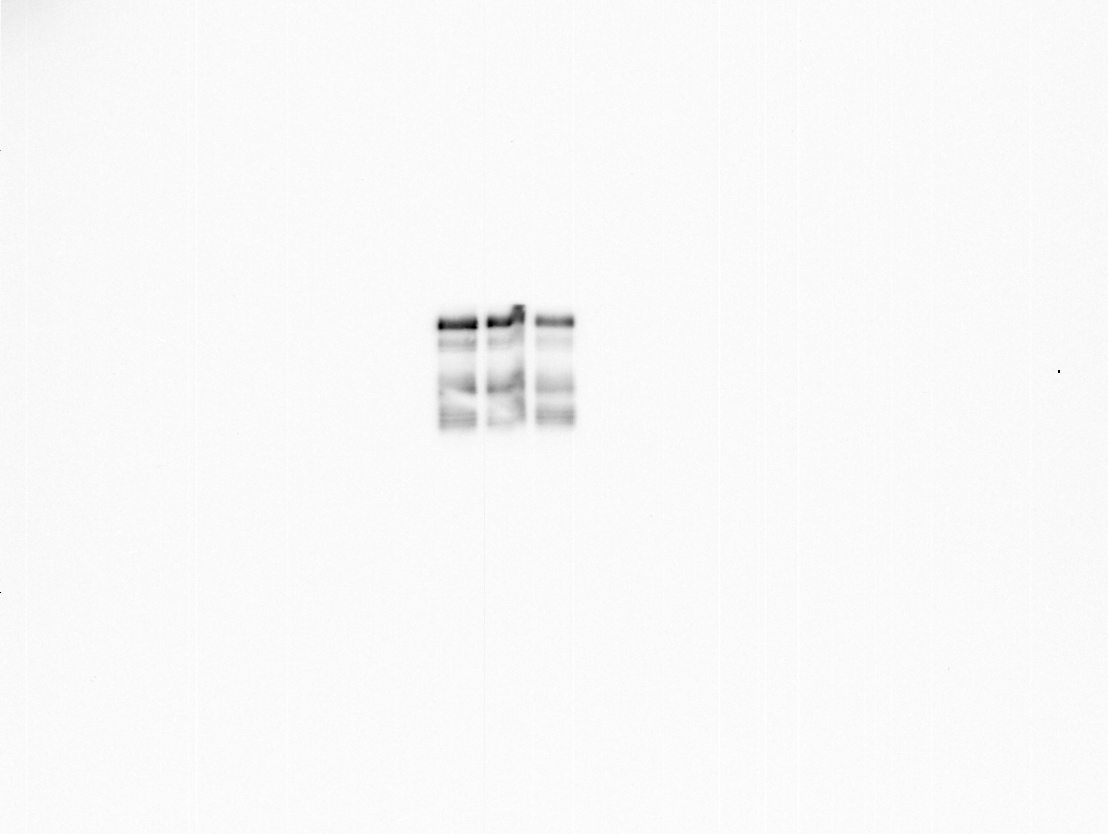

Supplement: Figure 4—source data 3. [file elife-84319-fig4-data3.zip › Figure 4ΓÇôSource Data 3/Figure 4H/IP myc/Replica 1.tif]

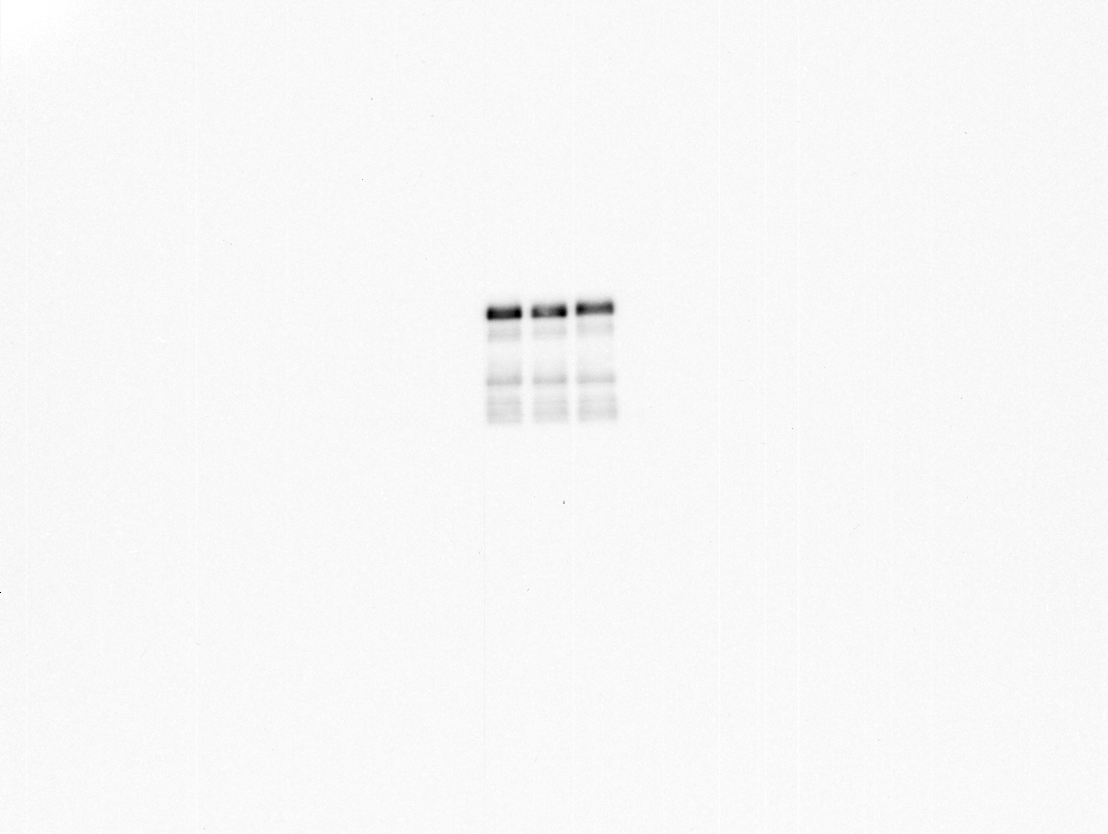

Supplement: Figure 4—source data 3. [file elife-84319-fig4-data3.zip › Figure 4ΓÇôSource Data 3/Figure 4H/Input myc/Replica 4.tif]

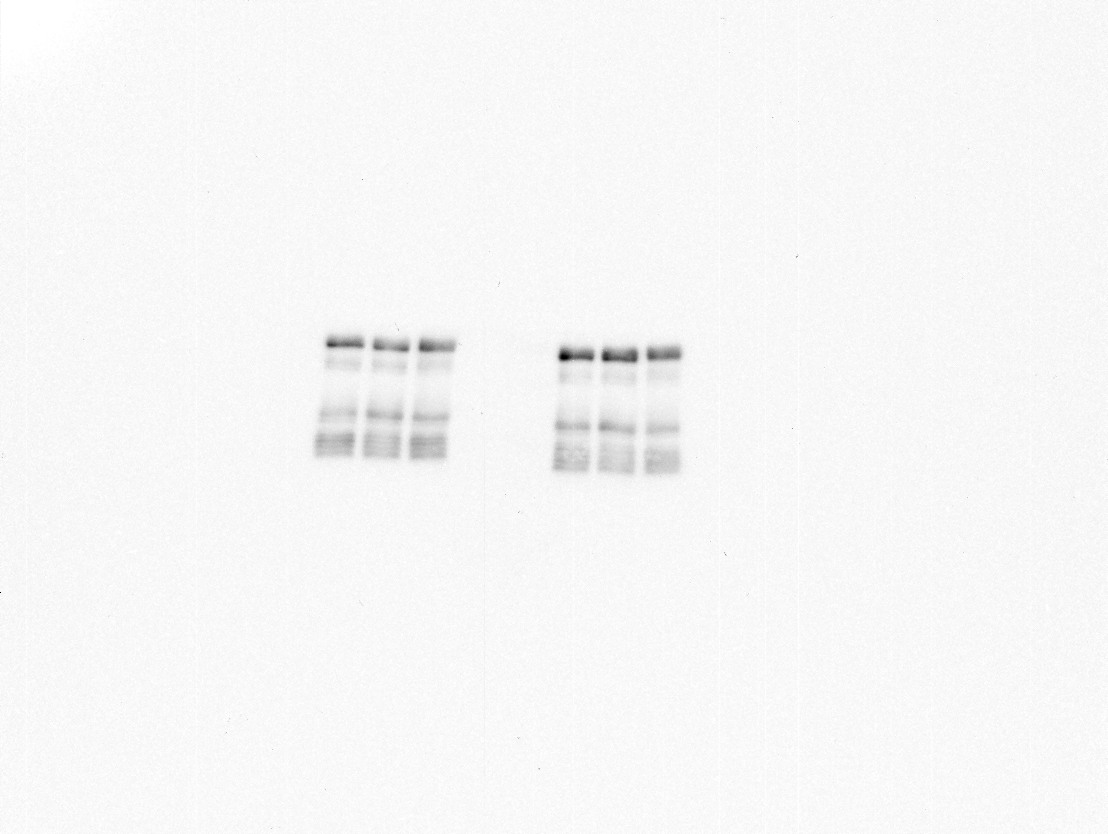

Supplement: Figure 4—source data 3. [file elife-84319-fig4-data3.zip › Figure 4ΓÇôSource Data 3/Figure 4H/Input myc/Replica 2_3.tif]

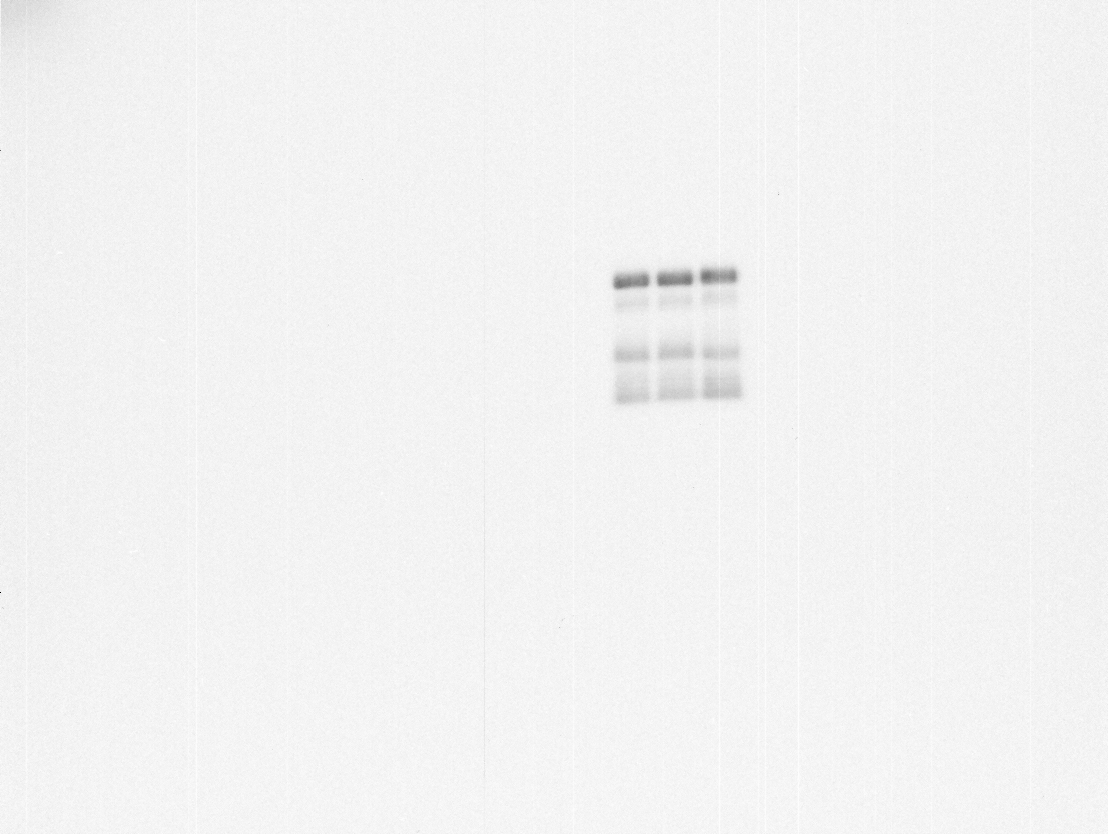

Supplement: Figure 4—source data 3. [file elife-84319-fig4-data3.zip › Figure 4ΓÇôSource Data 3/Figure 4H/Input myc/Replica 1.tif]

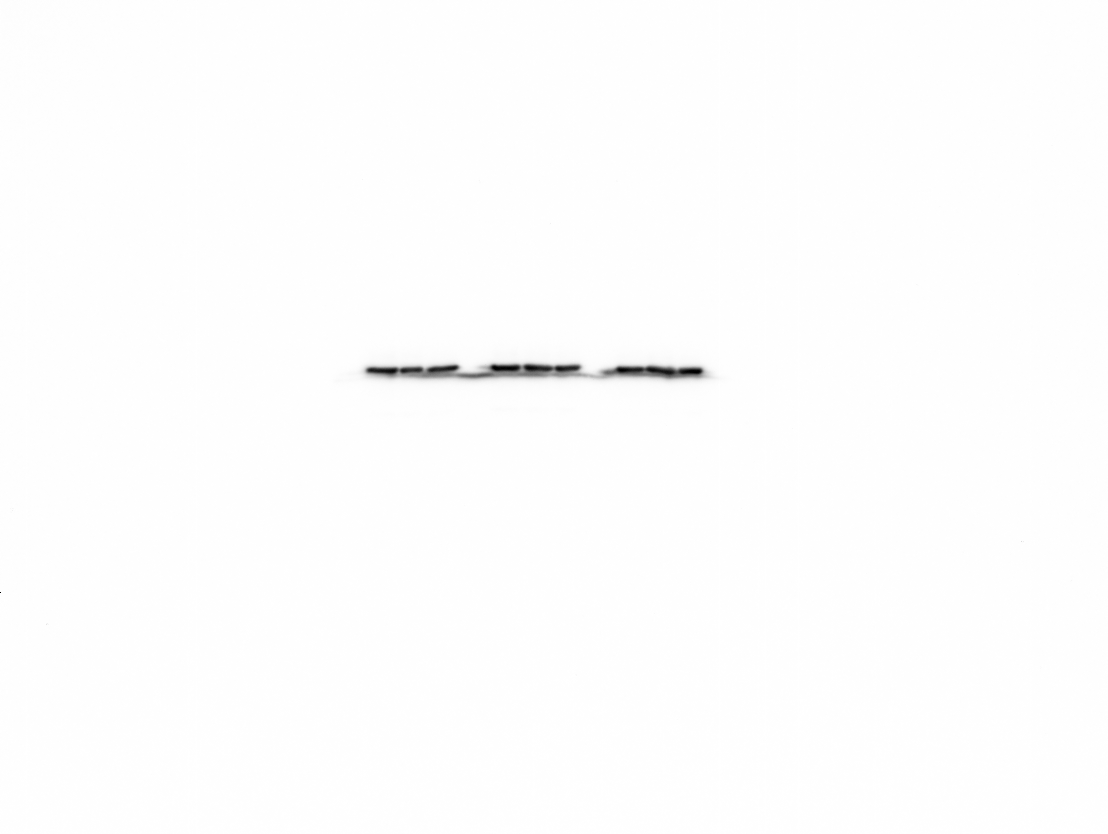

Supplement: Figure 4—source data 3. [file elife-84319-fig4-data3.zip › Figure 4ΓÇôSource Data 3/Figure 4I/Adh1/Replica 1_2_3.tif]

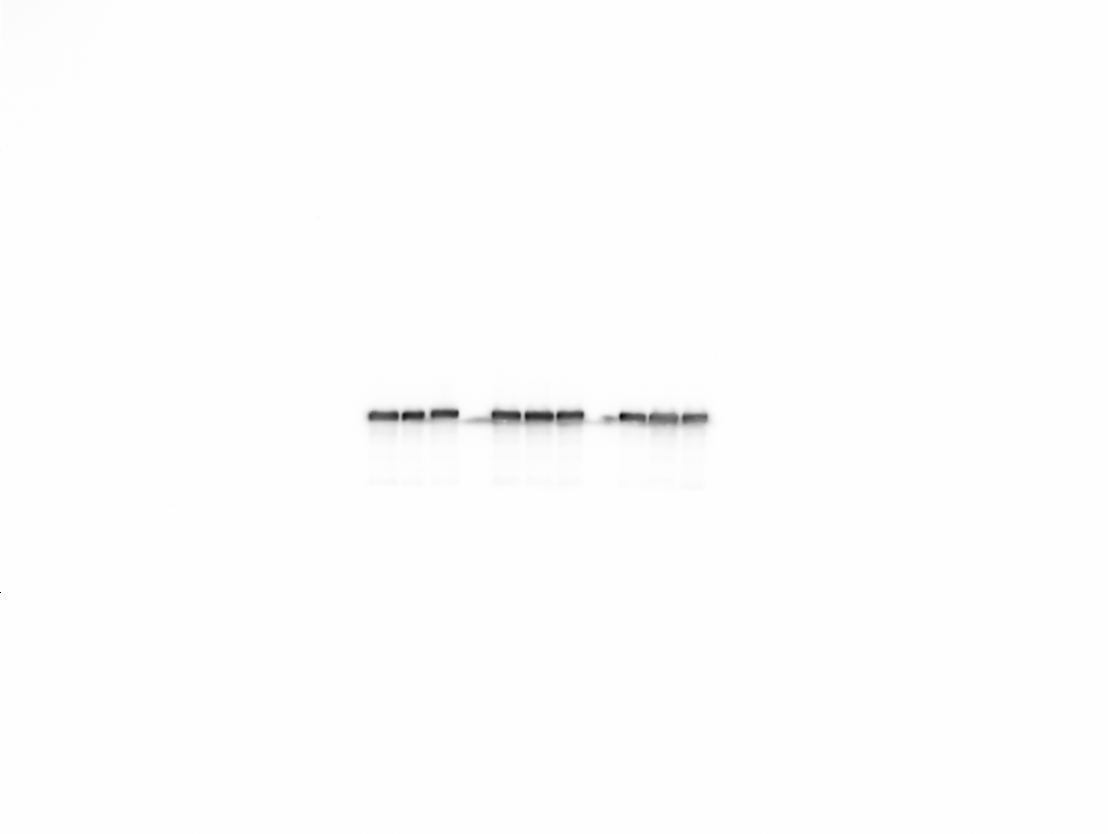

Supplement: Figure 4—source data 3. [file elife-84319-fig4-data3.zip › Figure 4ΓÇôSource Data 3/Figure 4I/myc/Replica 1_2_3.tif]

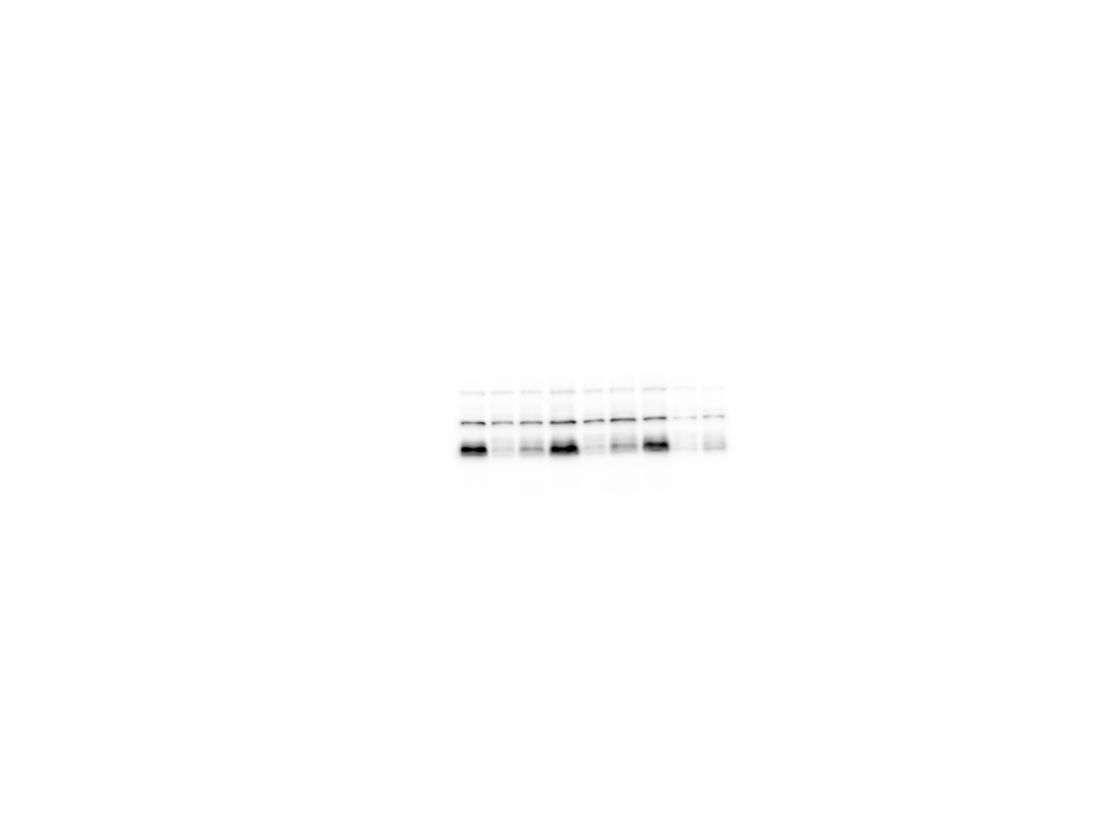

Supplement: Figure 5—source data 3. [file elife-84319-fig5-data3.zip › Figure 5ΓÇôSource Data 3/Figure 5F/Sch9-pThr737/Replica 6.tif]

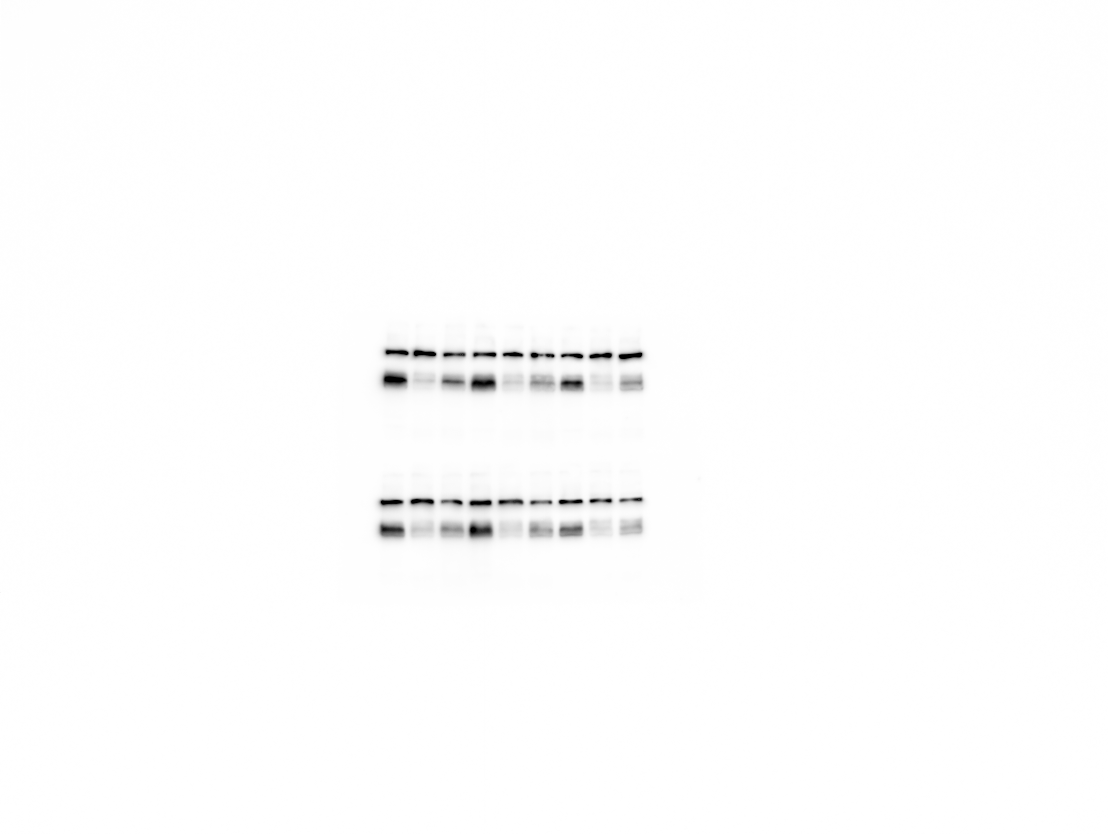

Supplement: Figure 5—source data 3. [file elife-84319-fig5-data3.zip › Figure 5ΓÇôSource Data 3/Figure 5F/Sch9-pThr737/Replica 4_5.tif]

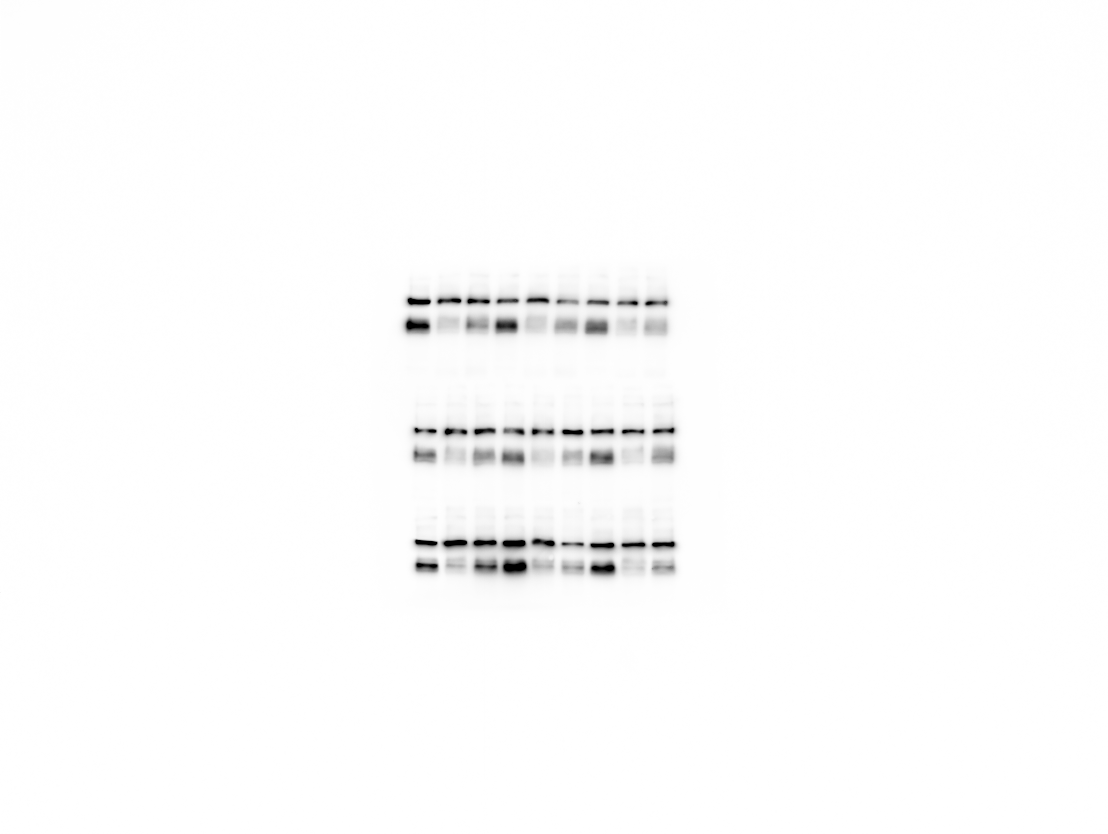

Supplement: Figure 5—source data 3. [file elife-84319-fig5-data3.zip › Figure 5ΓÇôSource Data 3/Figure 5F/Sch9-pThr737/Replica 1_2_3.tif]

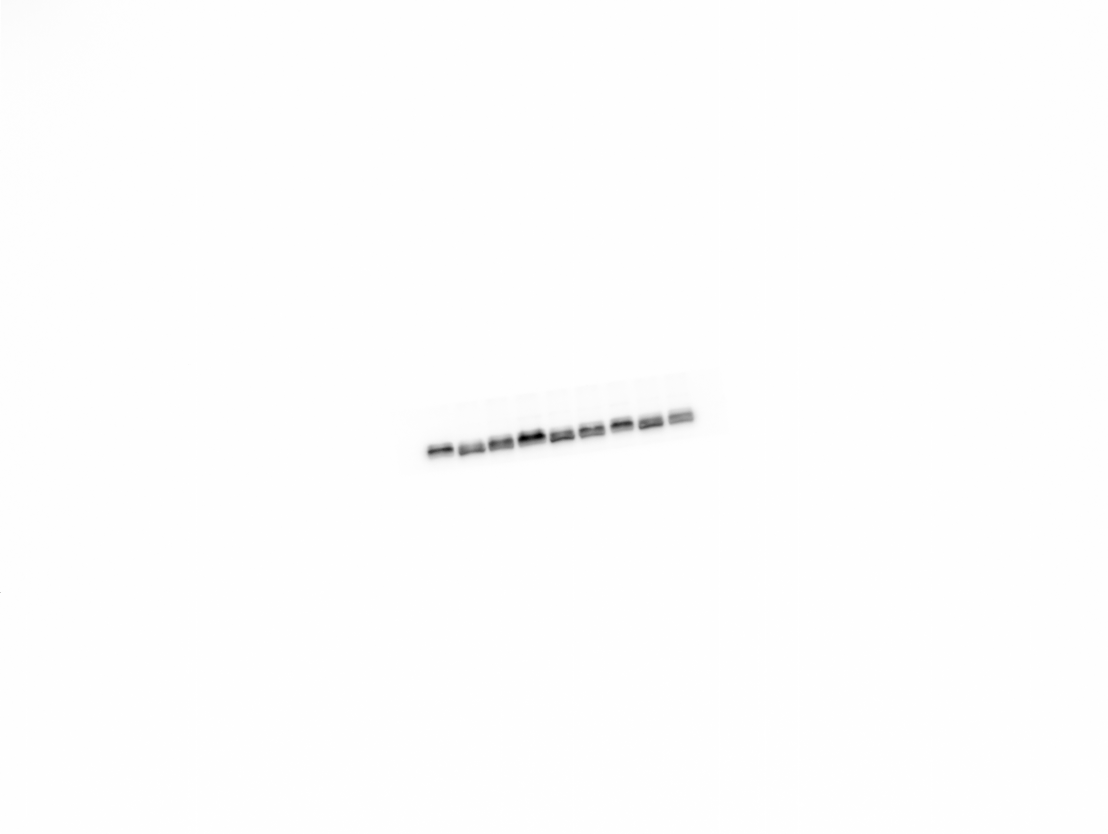

Supplement: Figure 5—source data 3. [file elife-84319-fig5-data3.zip › Figure 5ΓÇôSource Data 3/Figure 5F/Sch9/Replica 6.tif]

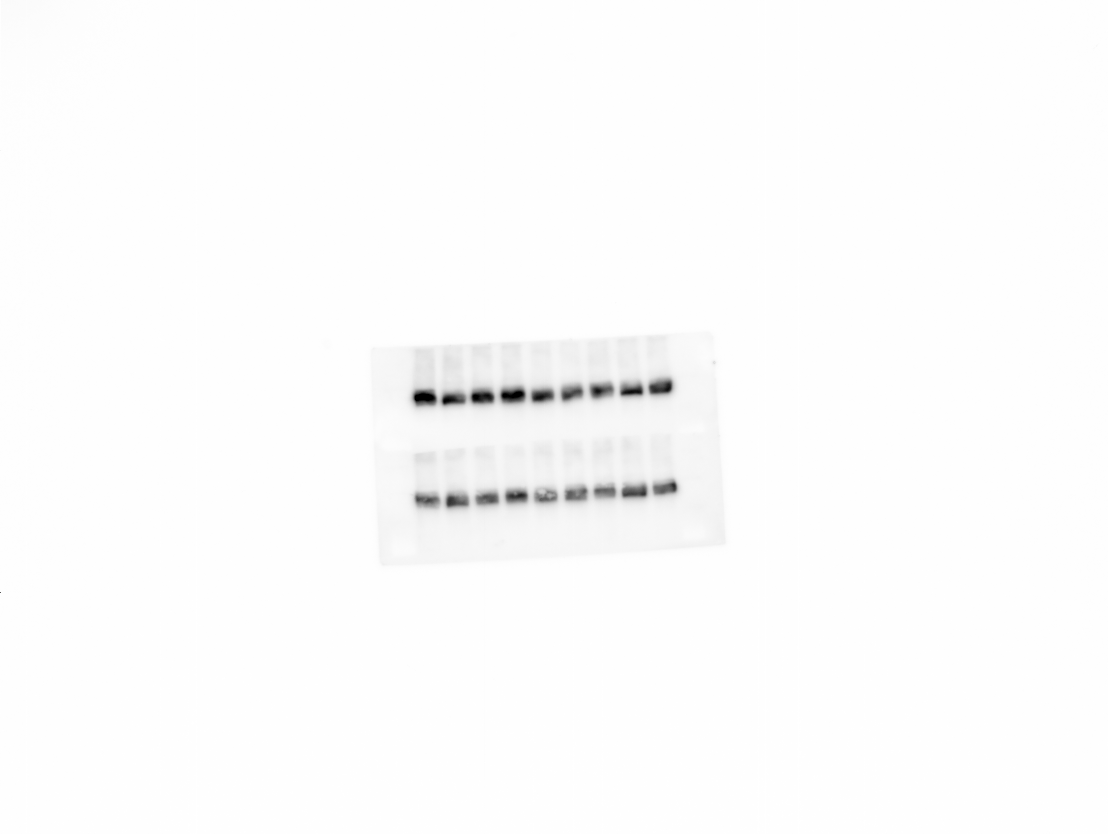

Supplement: Figure 5—source data 3. [file elife-84319-fig5-data3.zip › Figure 5ΓÇôSource Data 3/Figure 5F/Sch9/Replica 4_5.tif]

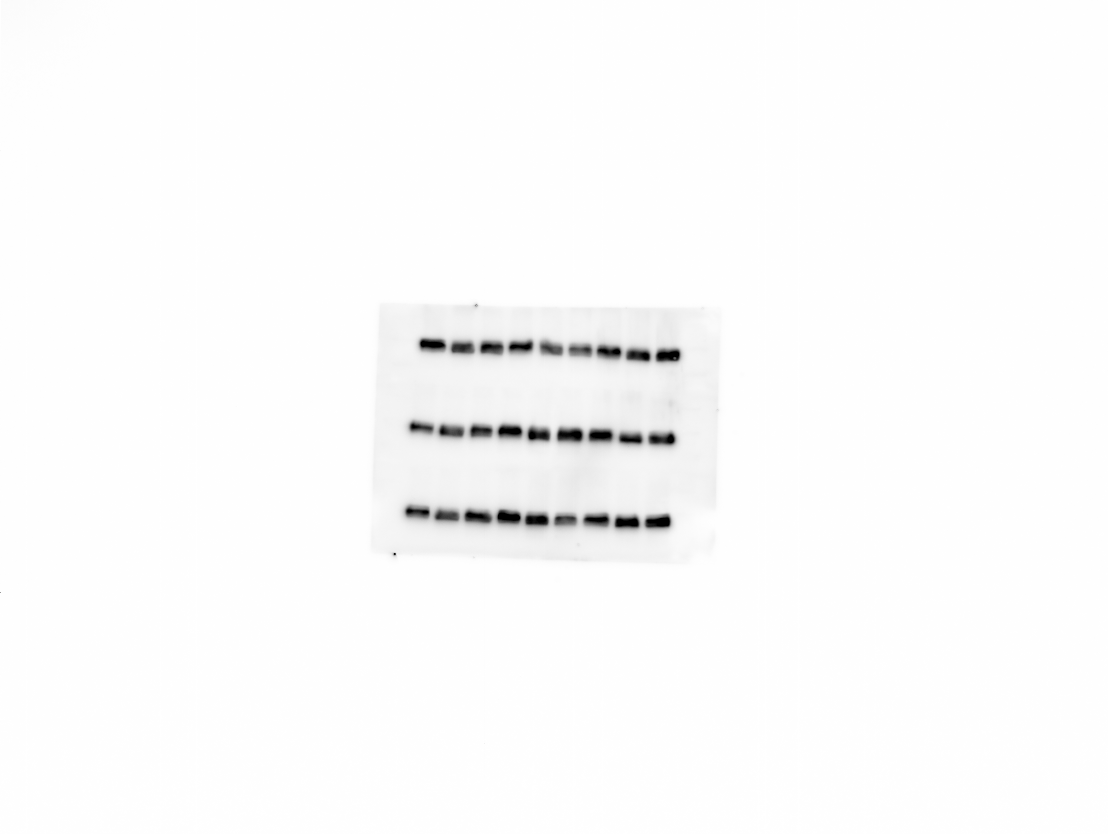

Supplement: Figure 5—source data 3. [file elife-84319-fig5-data3.zip › Figure 5ΓÇôSource Data 3/Figure 5F/Sch9/Replica 1_2_3.tif]

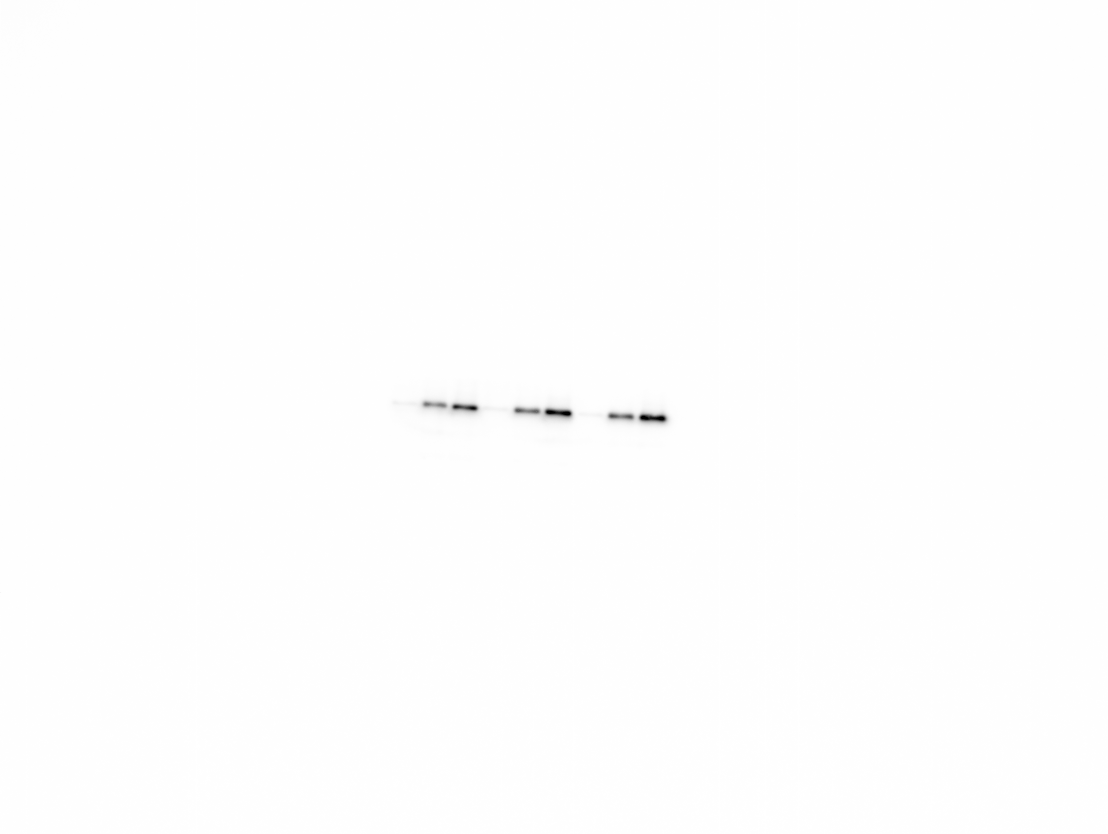

Supplement: Figure 5—source data 3. [file elife-84319-fig5-data3.zip › Figure 5ΓÇôSource Data 3/Figure 5F/Snf1-pThr210/Replica 6.tif]

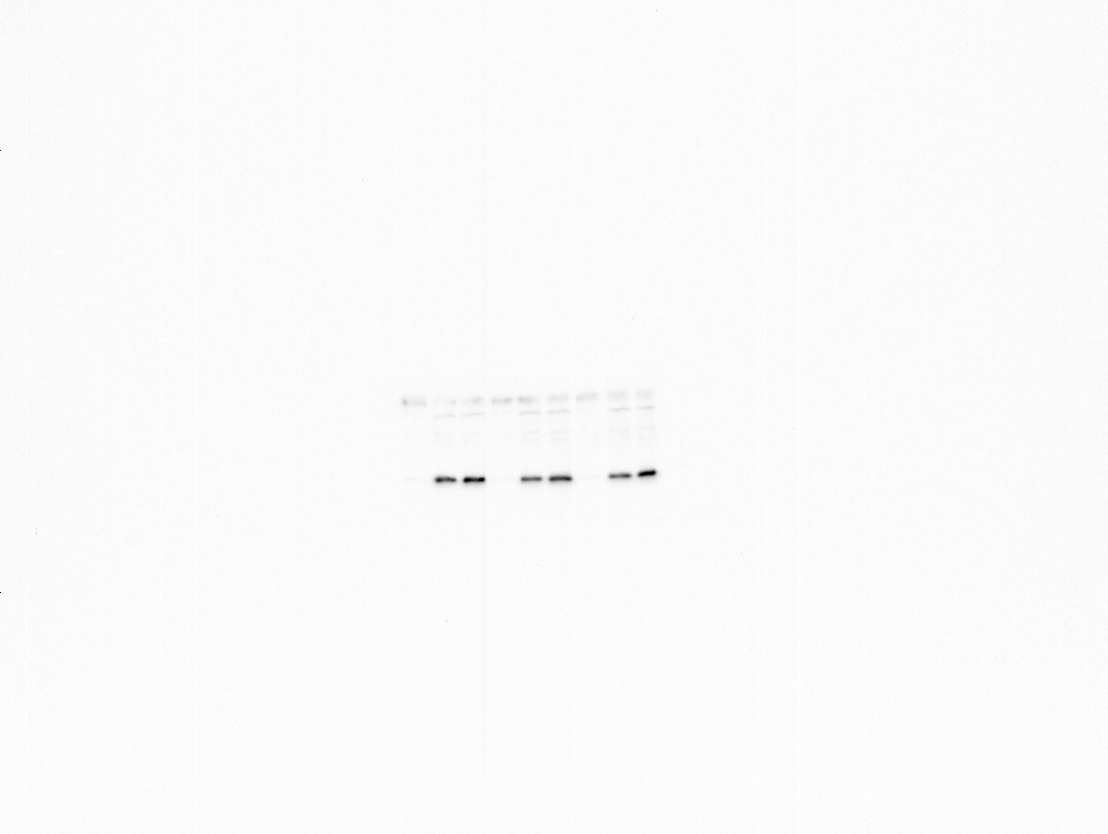

Supplement: Figure 5—source data 3. [file elife-84319-fig5-data3.zip › Figure 5ΓÇôSource Data 3/Figure 5F/Snf1-pThr210/Replica 1.tif]

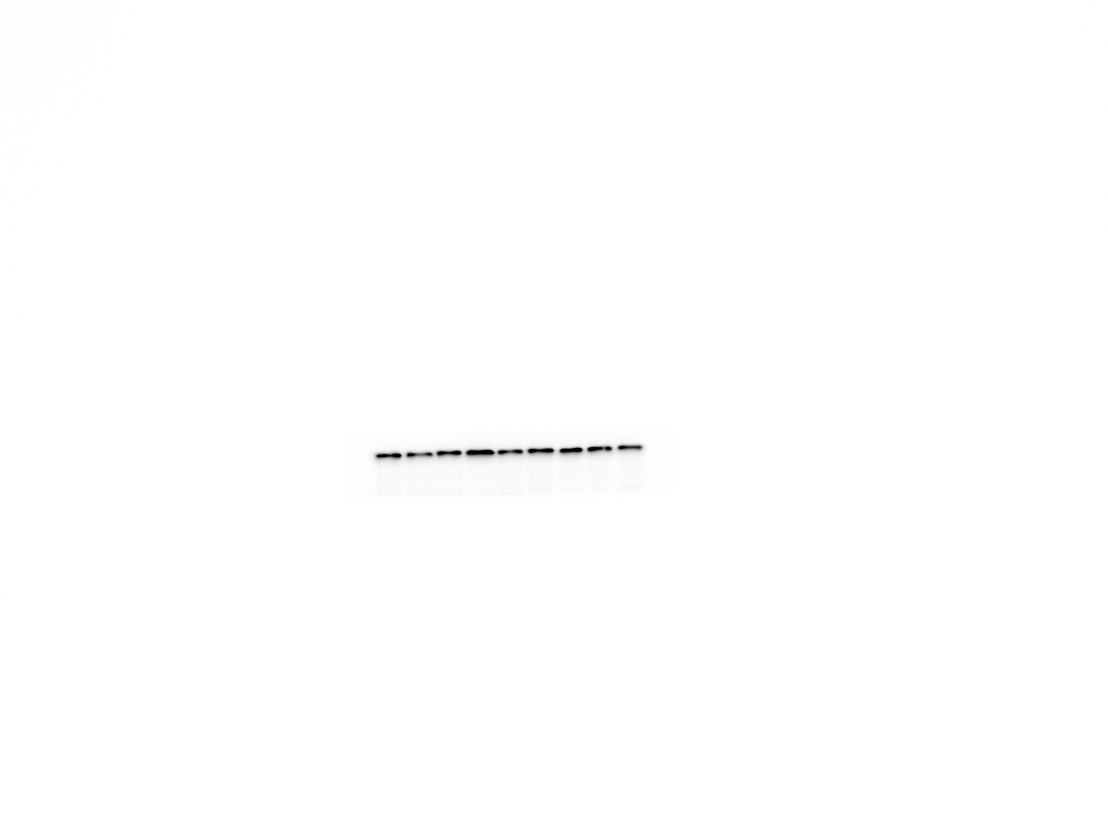

Supplement: Figure 5—source data 3. [file elife-84319-fig5-data3.zip › Figure 5ΓÇôSource Data 3/Figure 5F/His6/Replica 6.tif]

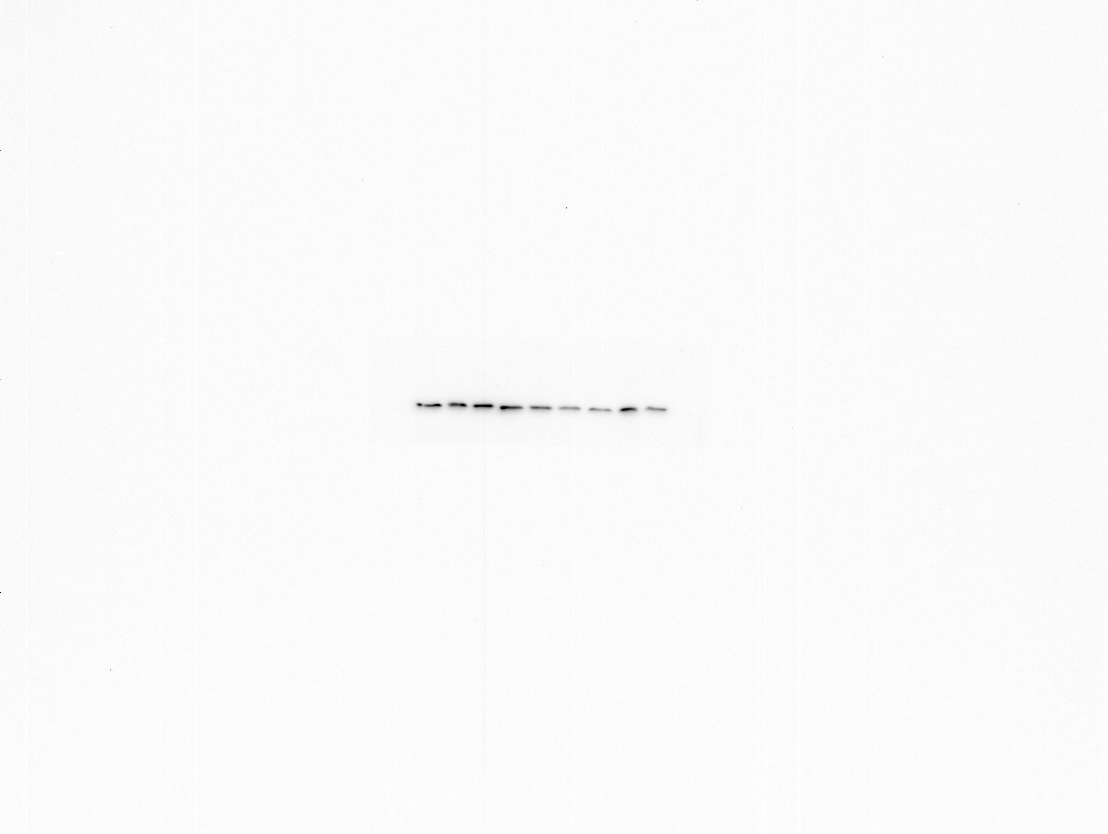

Supplement: Figure 5—source data 3. [file elife-84319-fig5-data3.zip › Figure 5ΓÇôSource Data 3/Figure 5F/His6/Replica 1.tif]

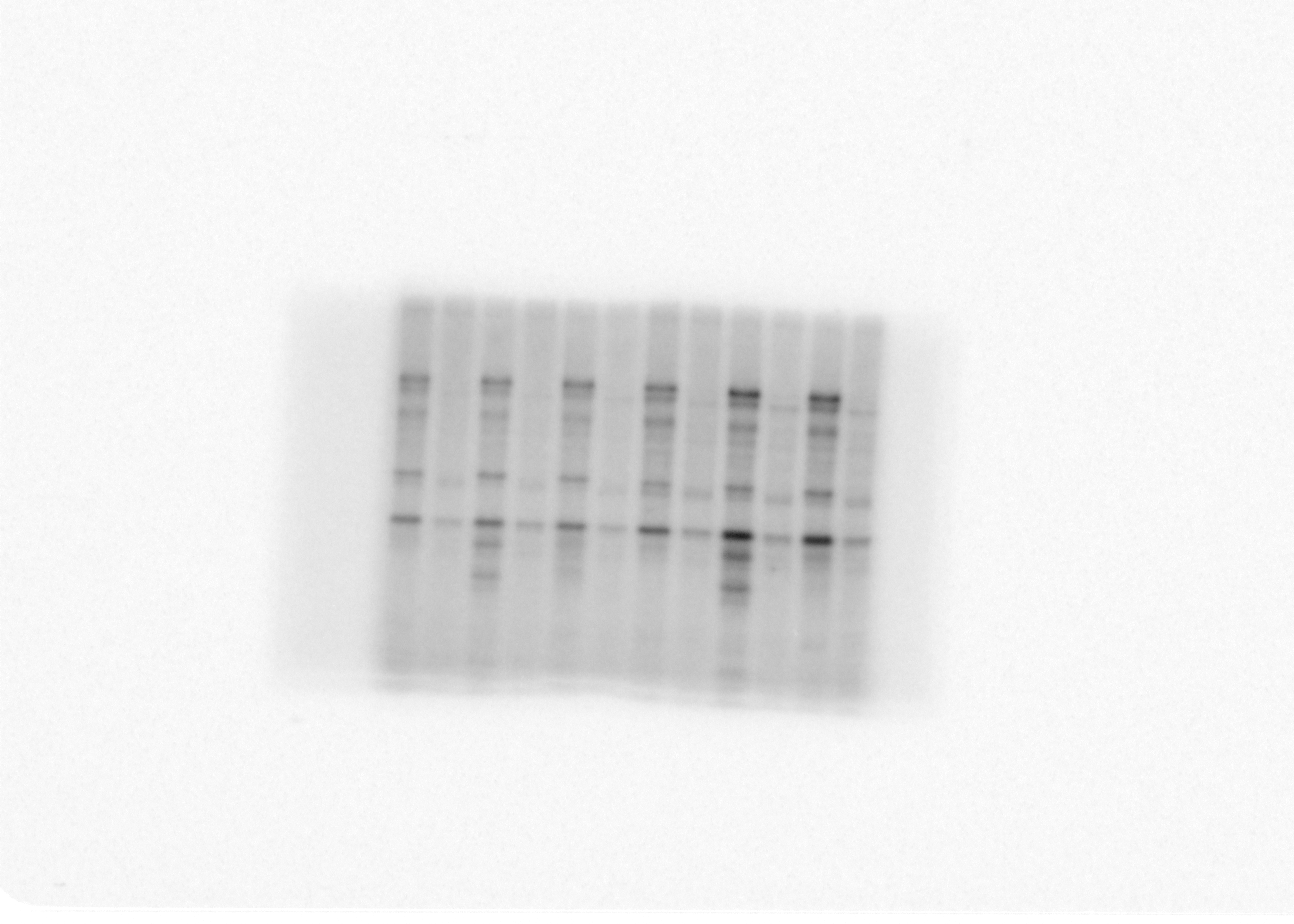

Supplement: Figure 5—source data 3. [file elife-84319-fig5-data3.zip › Figure 5ΓÇôSource Data 3/Figure 5C/32P/Replica 2_3.tif]

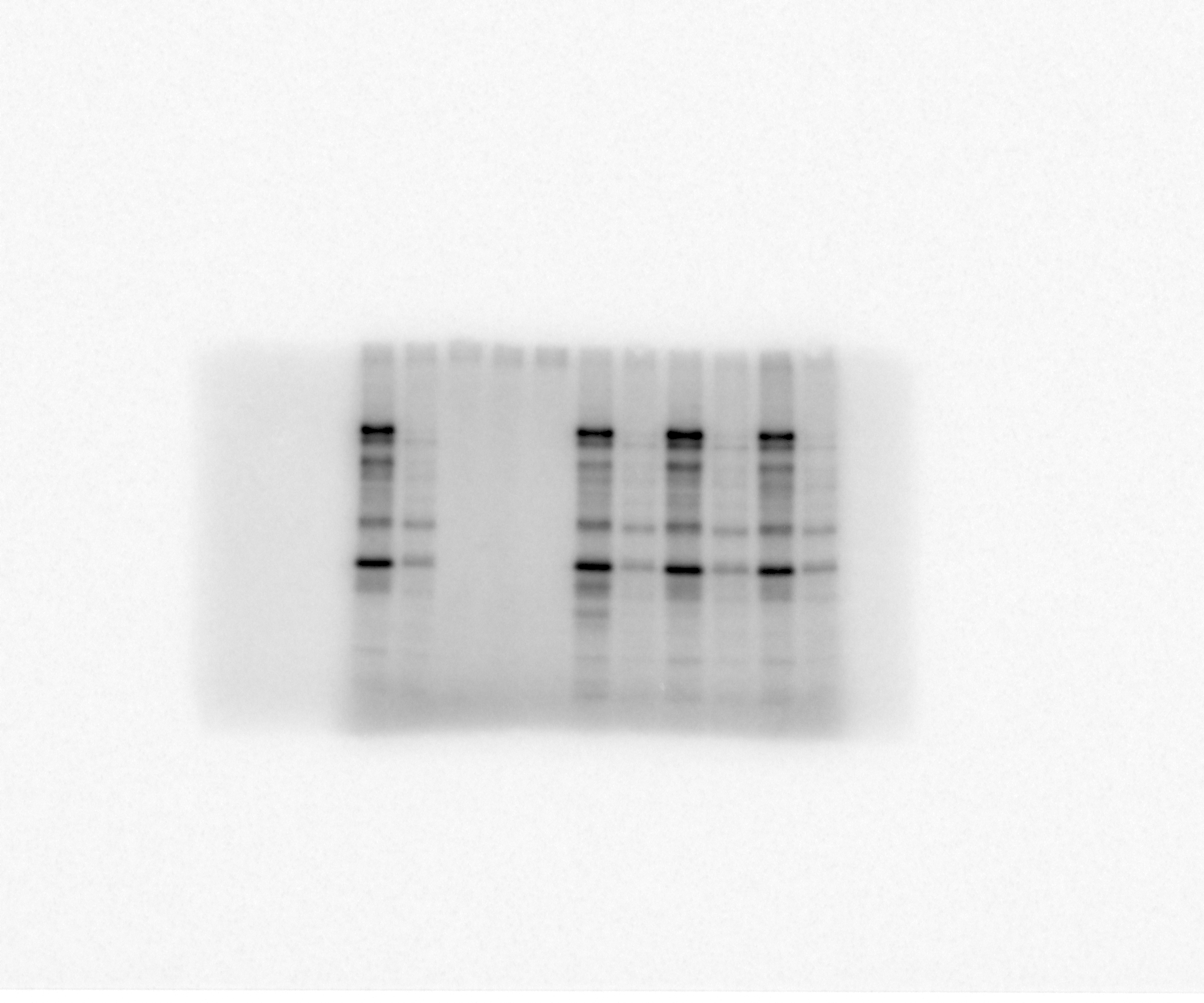

Supplement: Figure 5—source data 3. [file elife-84319-fig5-data3.zip › Figure 5ΓÇôSource Data 3/Figure 5C/32P/Replica 1.tif]

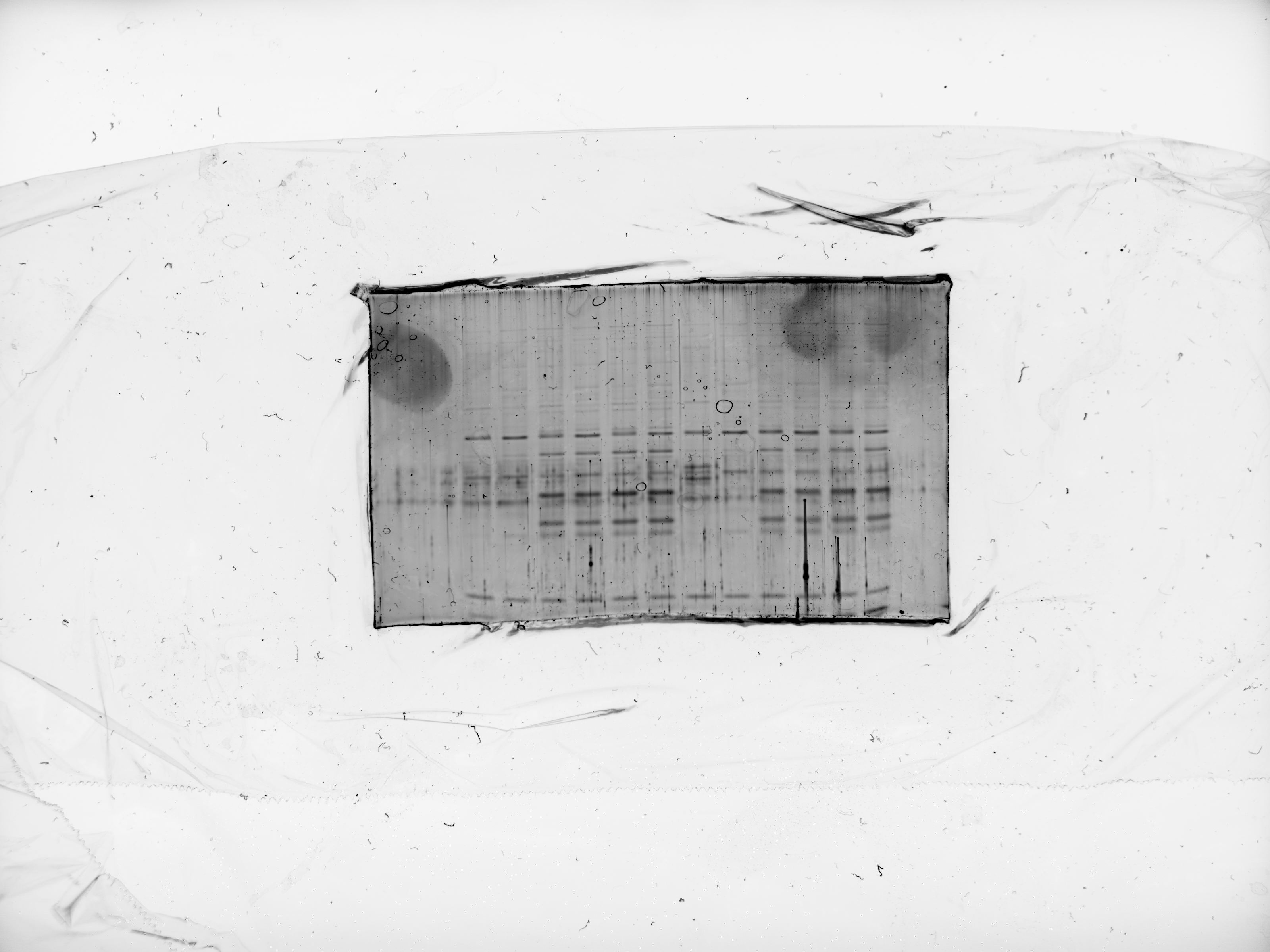

Supplement: Figure 5—source data 3. [file elife-84319-fig5-data3.zip › Figure 5ΓÇôSource Data 3/Figure 5C/Sypro Ruby/Replica 2_3.tif]

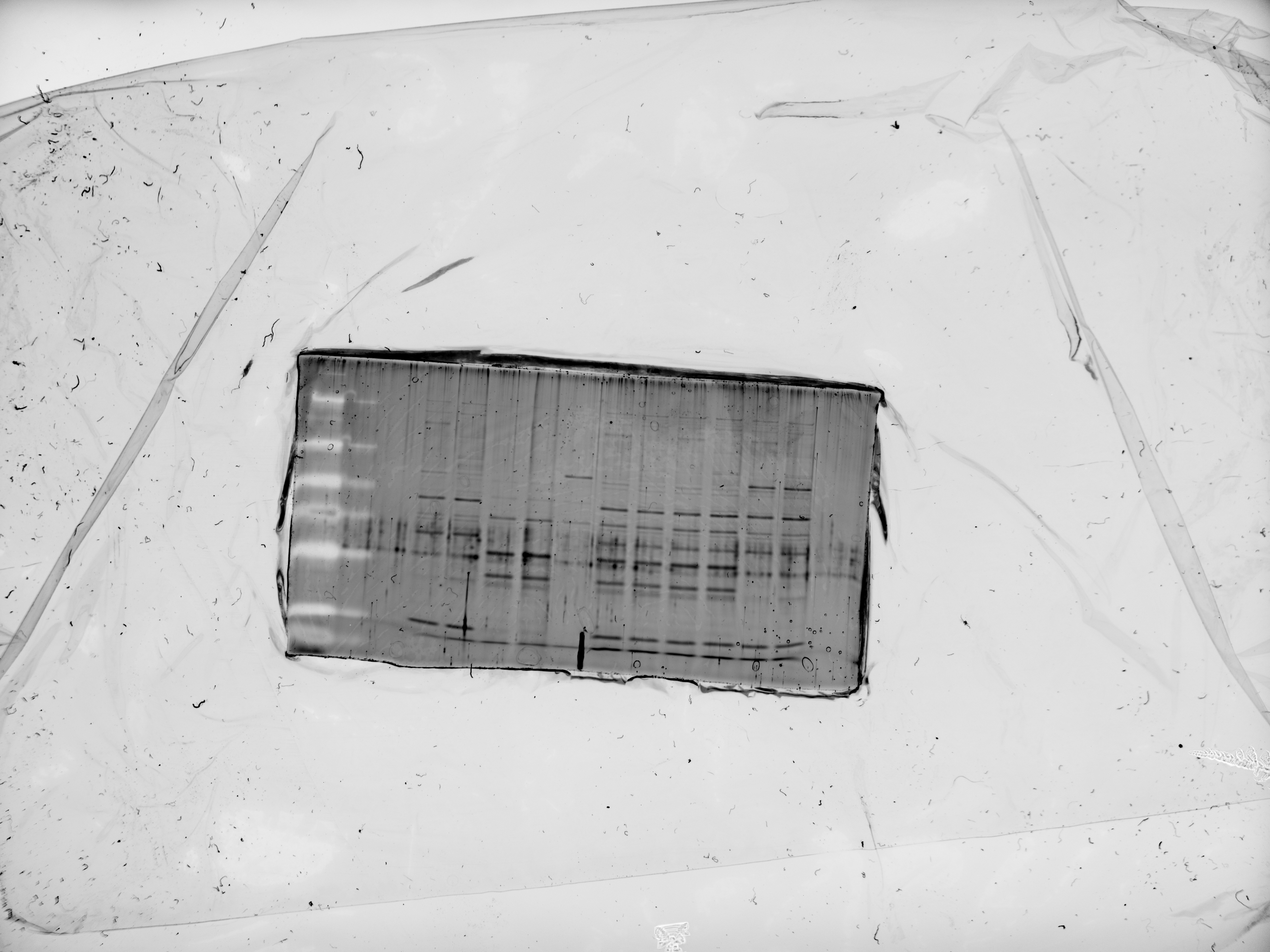

Supplement: Figure 5—source data 3. [file elife-84319-fig5-data3.zip › Figure 5ΓÇôSource Data 3/Figure 5C/Sypro Ruby/Replica 1.tif]

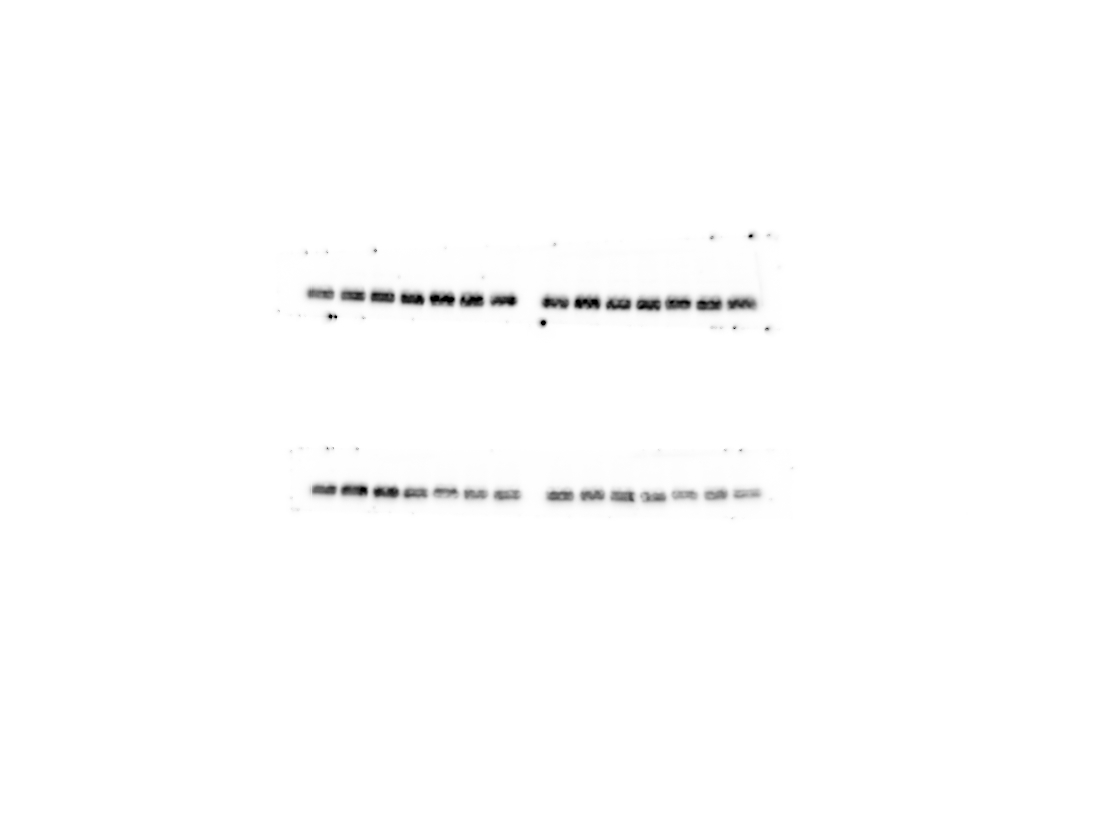

Supplement: Figure 5—source data 3. [file elife-84319-fig5-data3.zip › Figure 5ΓÇôSource Data 3/Figure 5D/Lower blots/Sch9/Replica 2.jpg]

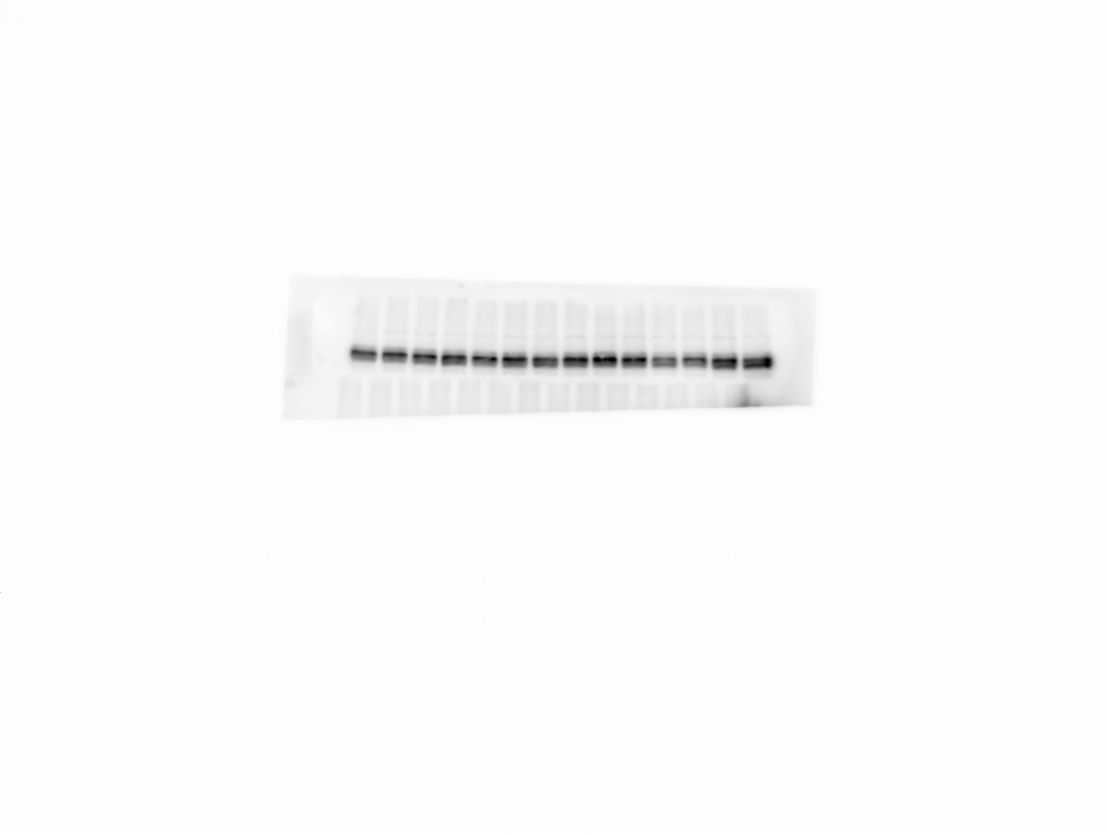

Supplement: Figure 5—source data 3. [file elife-84319-fig5-data3.zip › Figure 5ΓÇôSource Data 3/Figure 5D/Lower blots/Sch9/Replica 3_S288A.tif]

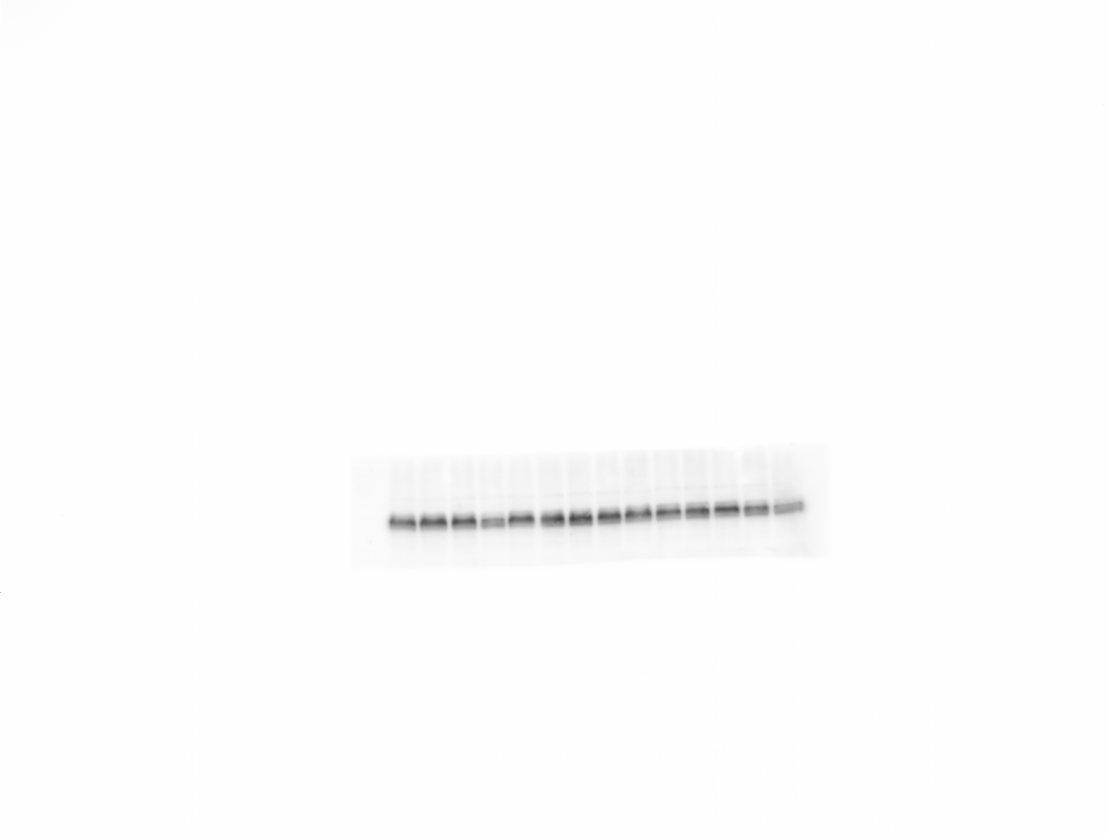

Supplement: Figure 5—source data 3. [file elife-84319-fig5-data3.zip › Figure 5ΓÇôSource Data 3/Figure 5D/Lower blots/Sch9/Replica 3_S288E.tif]

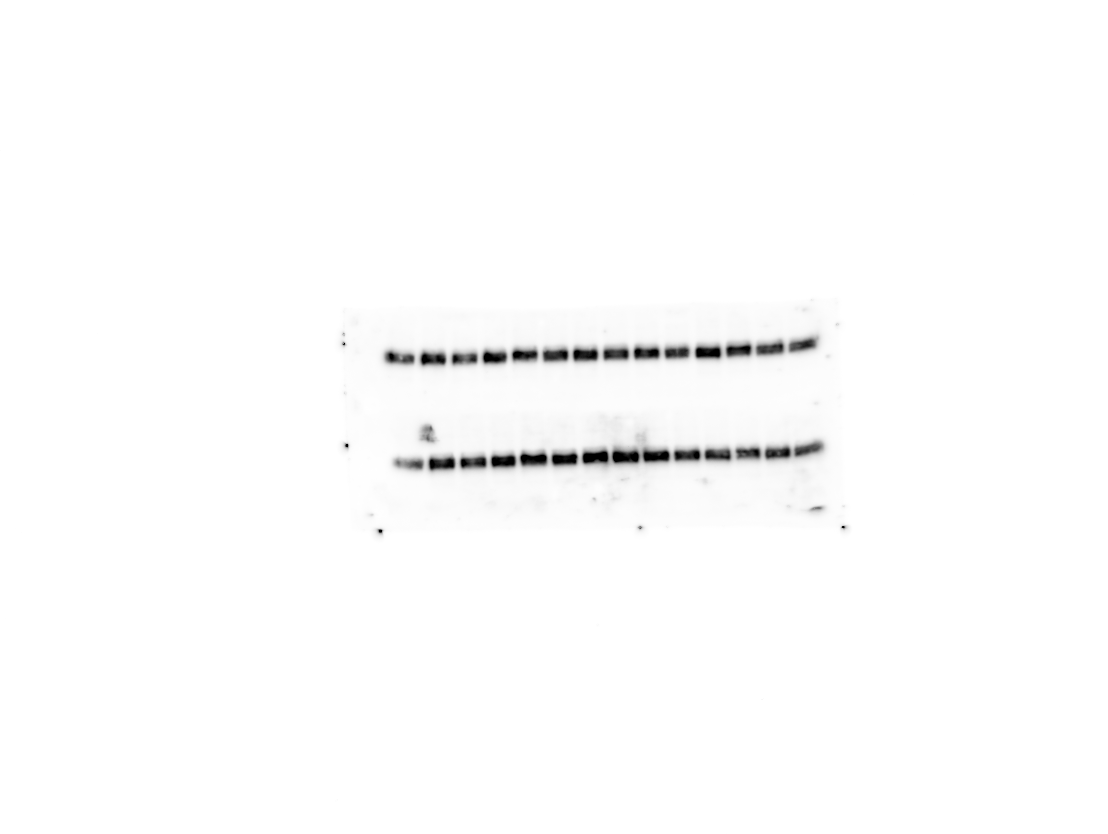

Supplement: Figure 5—source data 3. [file elife-84319-fig5-data3.zip › Figure 5ΓÇôSource Data 3/Figure 5D/Lower blots/Sch9/Replica 1.bmp]

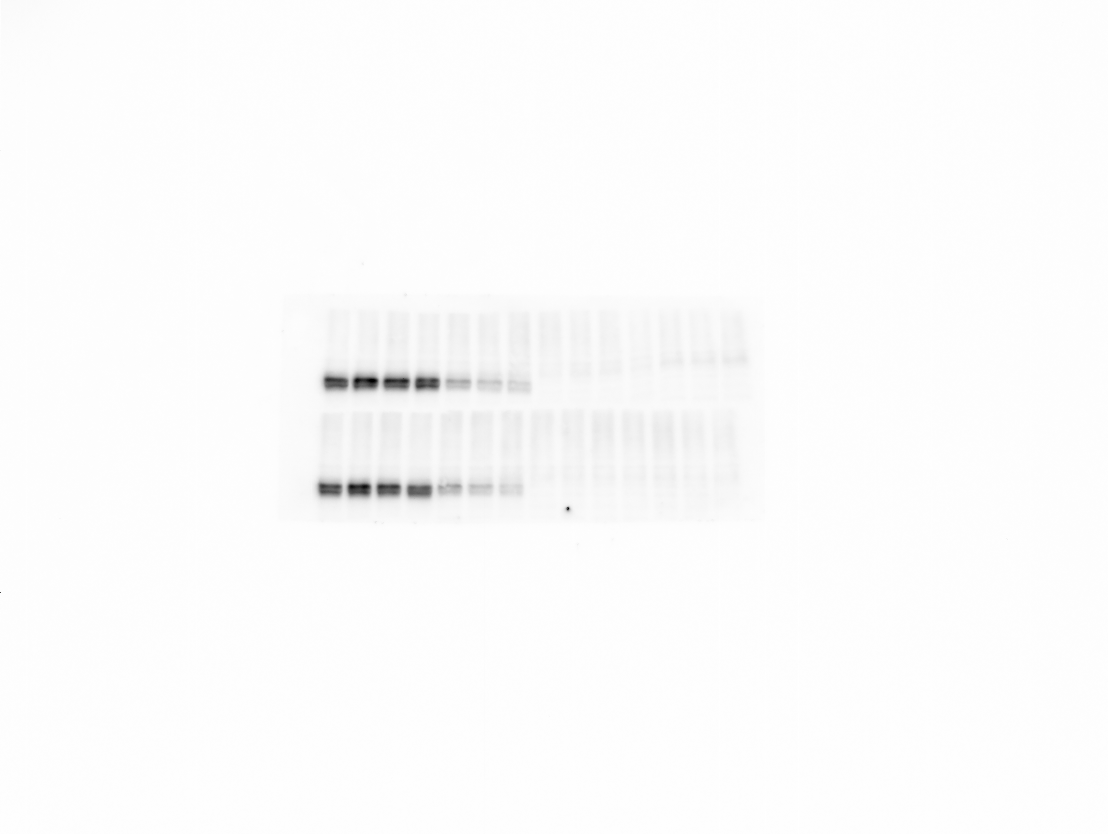

Supplement: Figure 5—source data 3. [file elife-84319-fig5-data3.zip › Figure 5ΓÇôSource Data 3/Figure 5D/Lower blots/Sch9-pSer288/Replica 3.tif]

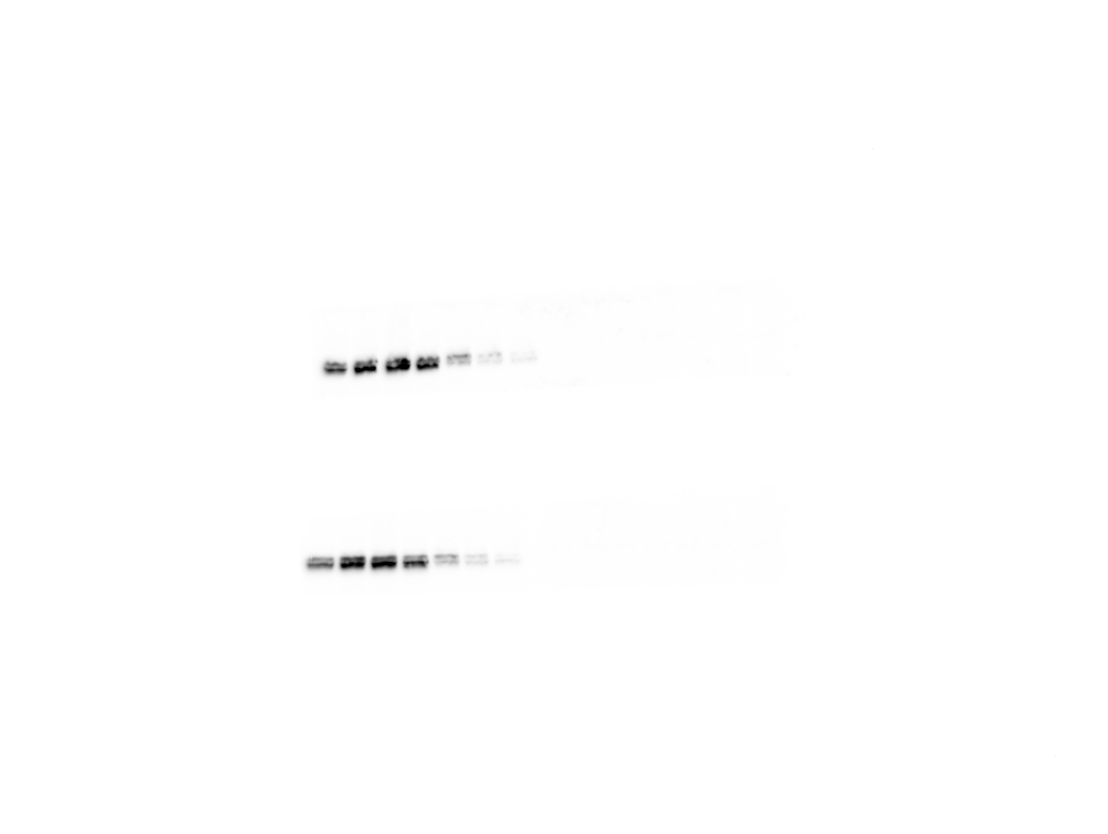

Supplement: Figure 5—source data 3. [file elife-84319-fig5-data3.zip › Figure 5ΓÇôSource Data 3/Figure 5D/Lower blots/Sch9-pSer288/Replica 2.jpg]

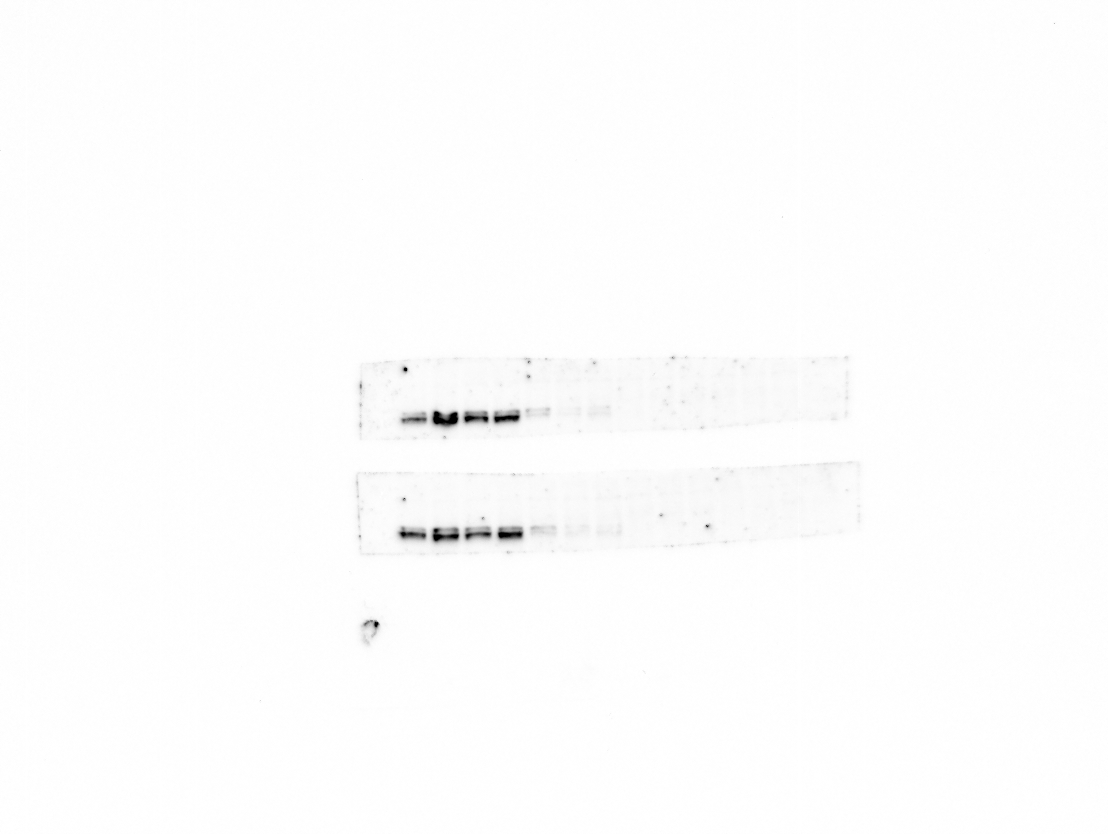

Supplement: Figure 5—source data 3. [file elife-84319-fig5-data3.zip › Figure 5ΓÇôSource Data 3/Figure 5D/Lower blots/Sch9-pSer288/Replica 1.bmp]

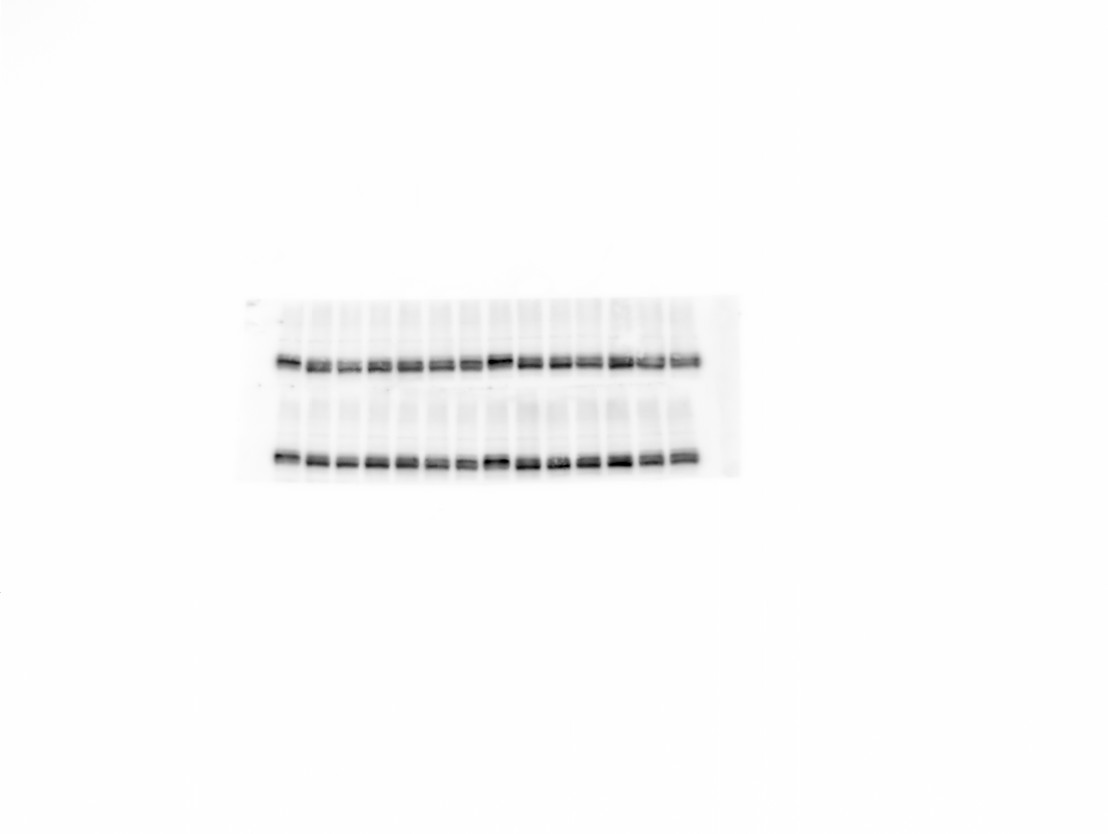

Supplement: Figure 5—source data 3. [file elife-84319-fig5-data3.zip › Figure 5ΓÇôSource Data 3/Figure 5D/Upper blots/Sch9/Replica 3.tif]

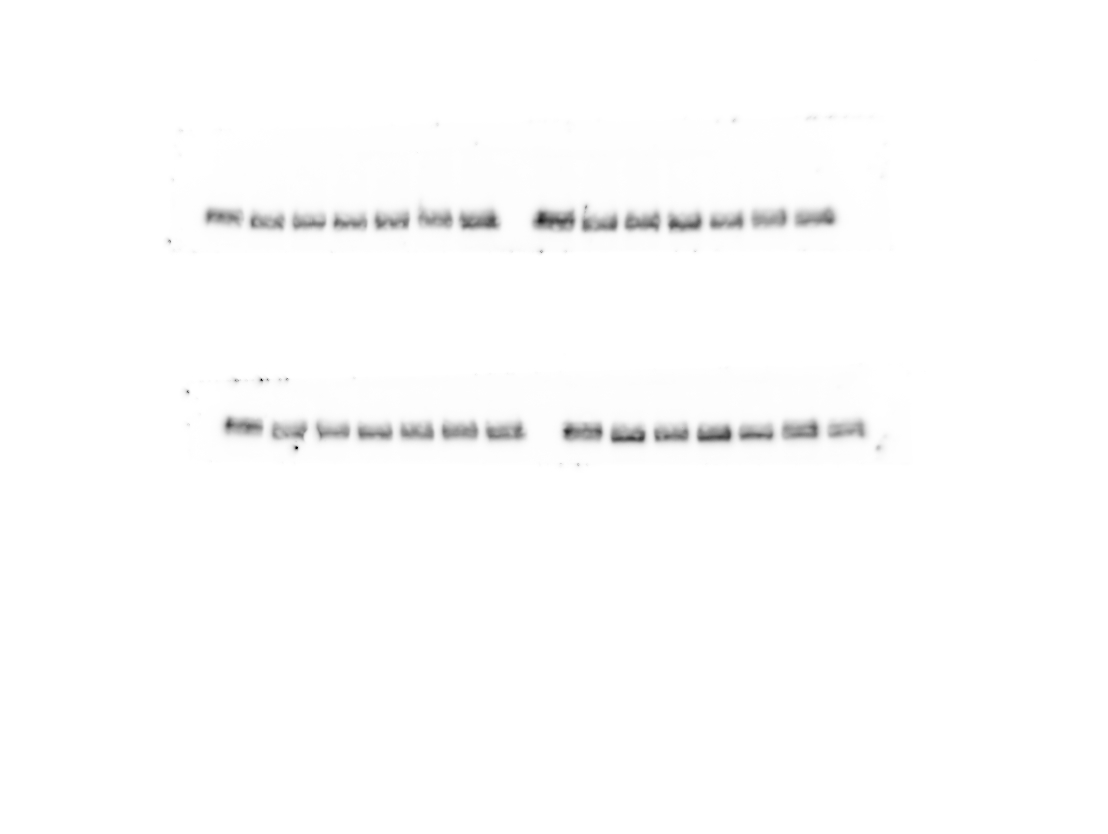

Supplement: Figure 5—source data 3. [file elife-84319-fig5-data3.zip › Figure 5ΓÇôSource Data 3/Figure 5D/Upper blots/Sch9/Replica 2.jpg]

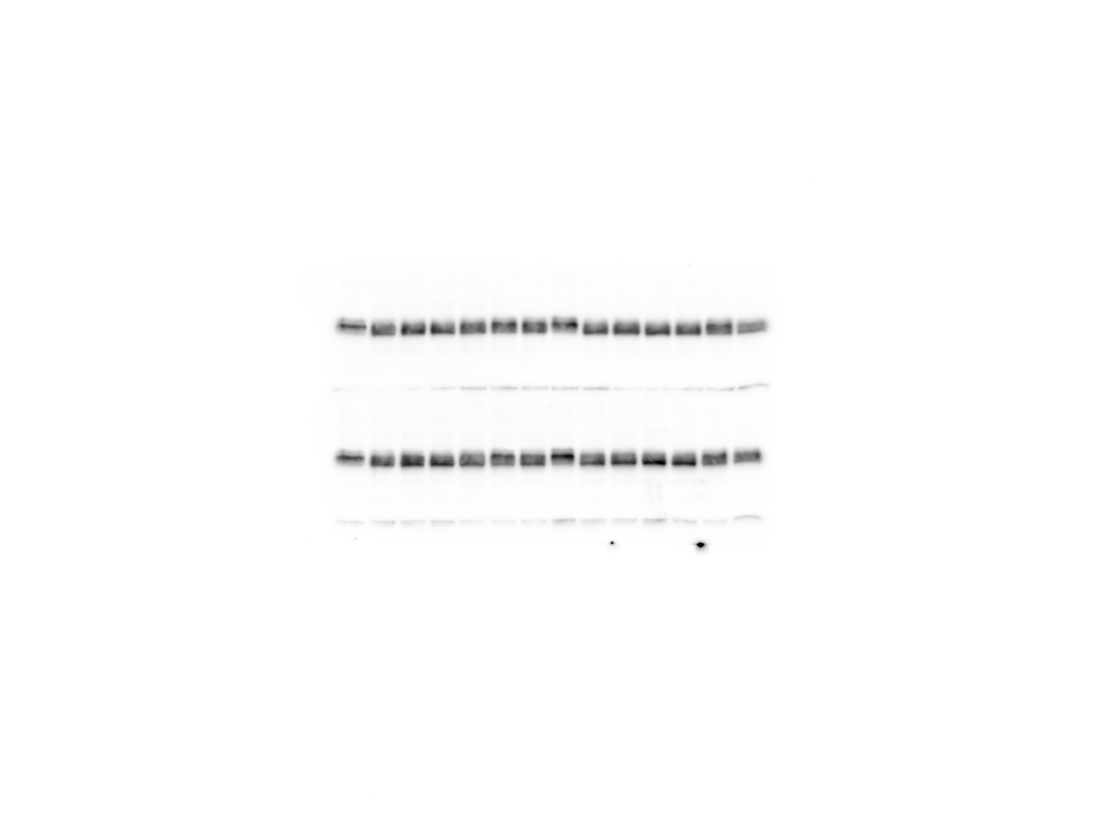

Supplement: Figure 5—source data 3. [file elife-84319-fig5-data3.zip › Figure 5ΓÇôSource Data 3/Figure 5D/Upper blots/Sch9/Replica 1.bmp]

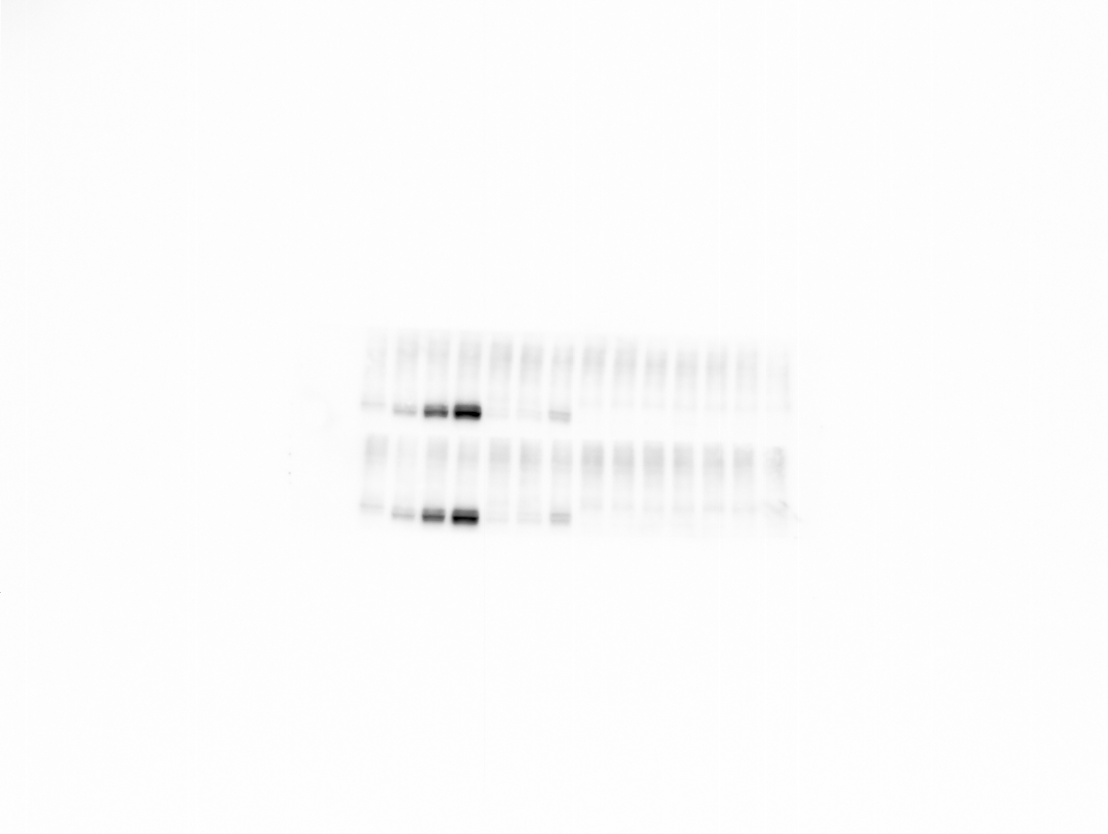

Supplement: Figure 5—source data 3. [file elife-84319-fig5-data3.zip › Figure 5ΓÇôSource Data 3/Figure 5D/Upper blots/Sch9-pSer288/Replica 3.tif]

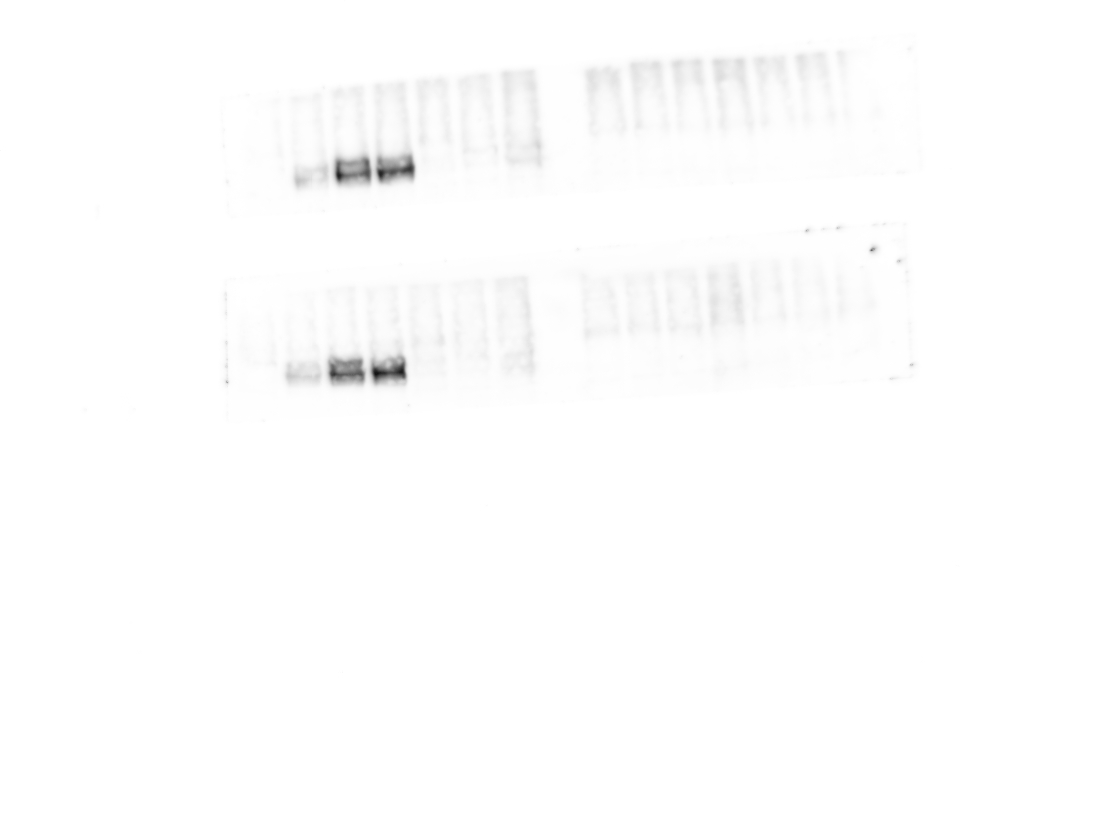

Supplement: Figure 5—source data 3. [file elife-84319-fig5-data3.zip › Figure 5ΓÇôSource Data 3/Figure 5D/Upper blots/Sch9-pSer288/Replica 2.jpg]

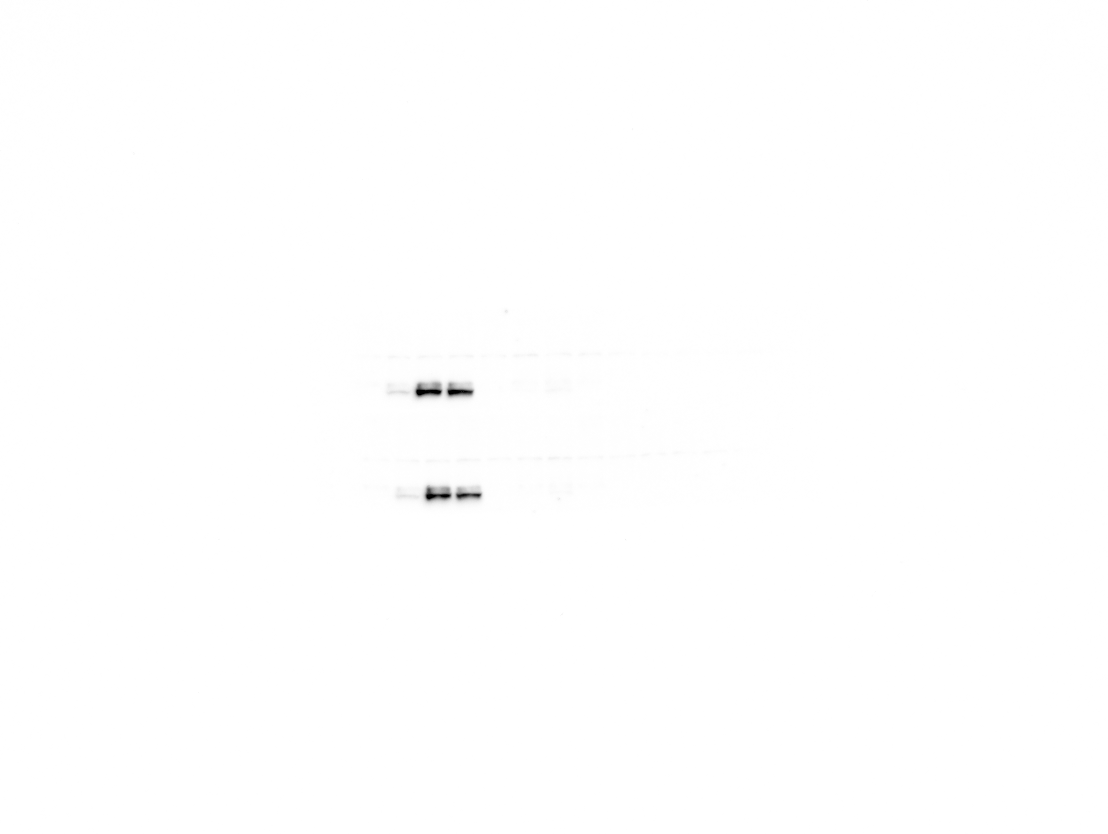

Supplement: Figure 5—source data 3. [file elife-84319-fig5-data3.zip › Figure 5ΓÇôSource Data 3/Figure 5D/Upper blots/Sch9-pSer288/Replica 1.bmp]

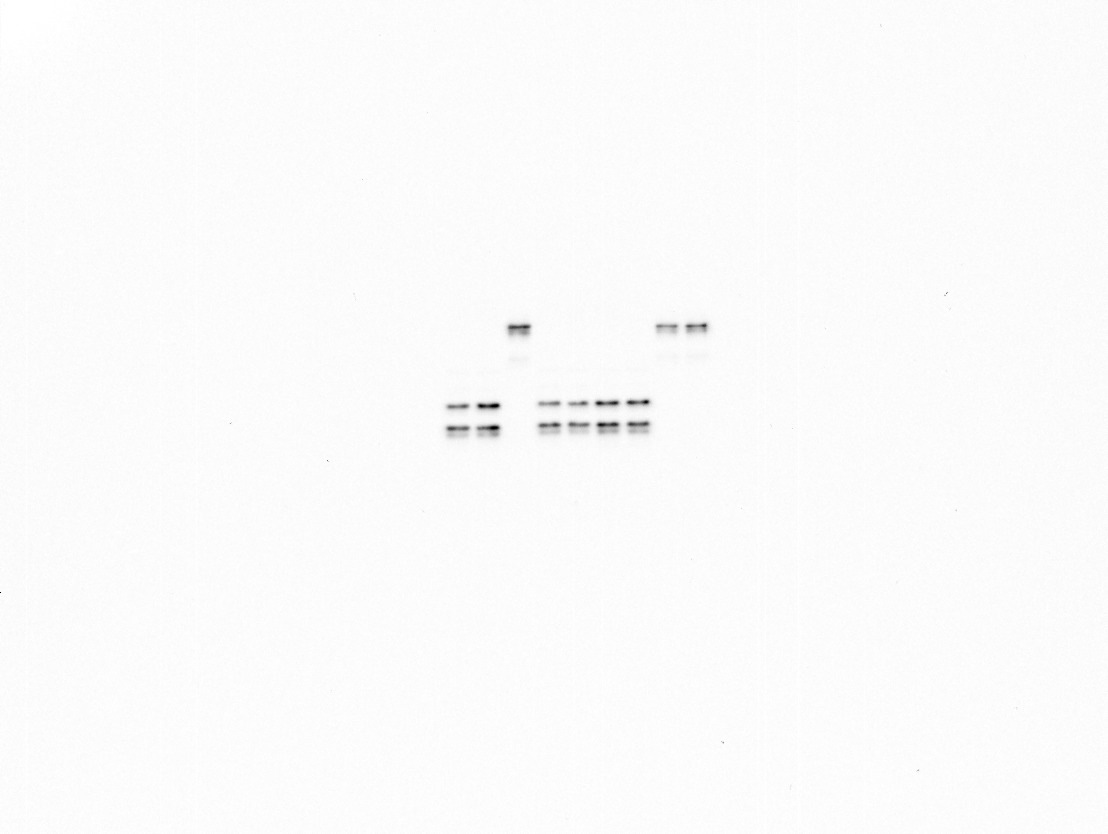

Supplement: Figure 5—figure supplement 1—source data 2. [file elife-84319-fig5-figsupp1-data2.zip › Figure 5ΓÇôSupplementary figure 3-Source Data 2/Supplementary figure 3/Sch9/Replica 2.tif]

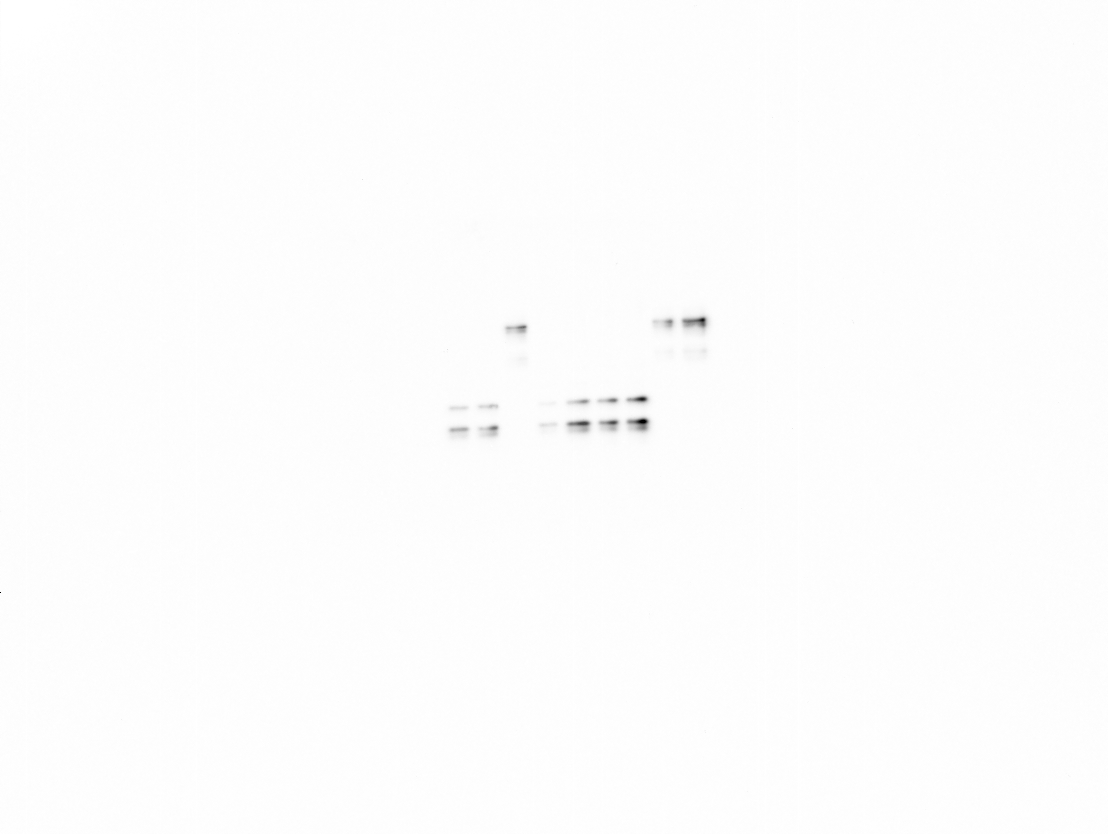

Supplement: Figure 5—figure supplement 1—source data 2. [file elife-84319-fig5-figsupp1-data2.zip › Figure 5ΓÇôSupplementary figure 3-Source Data 2/Supplementary figure 3/Sch9/Replica 1.tif]

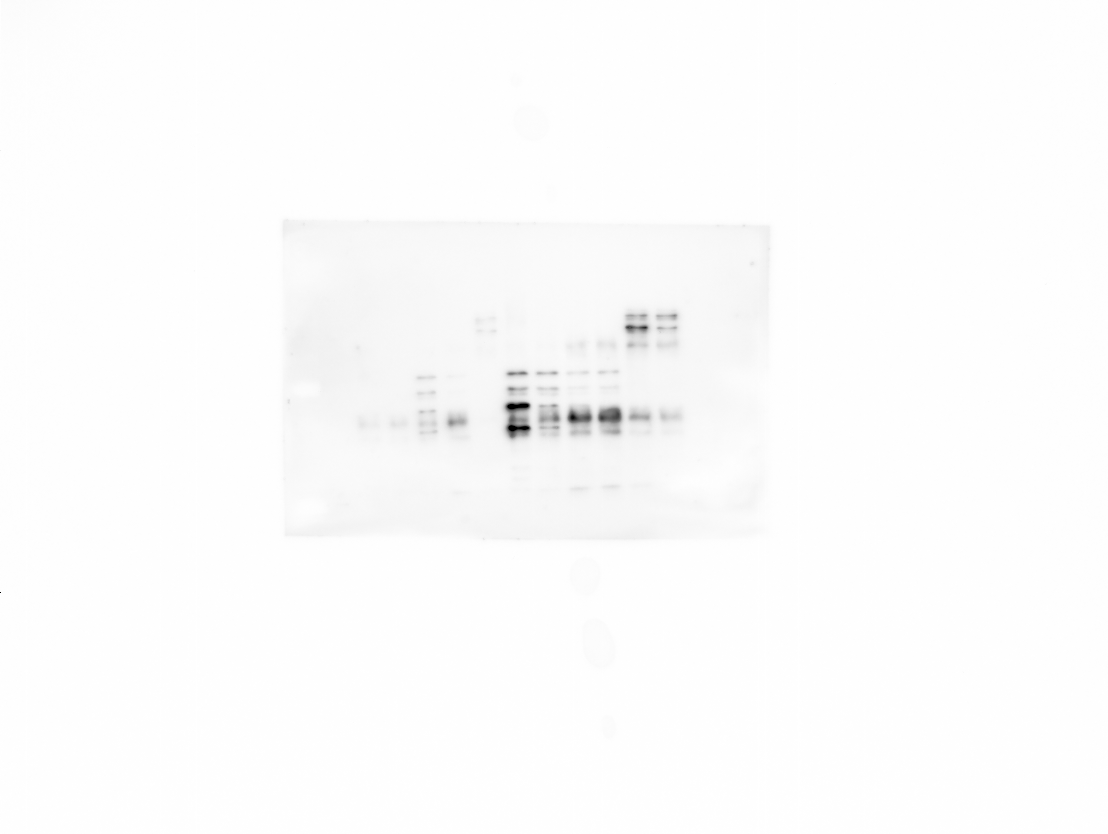

Supplement: Figure 5—figure supplement 1—source data 2. [file elife-84319-fig5-figsupp1-data2.zip › Figure 5ΓÇôSupplementary figure 3-Source Data 2/Supplementary figure 3/Sch9-pSer288/Replica 2.tif]

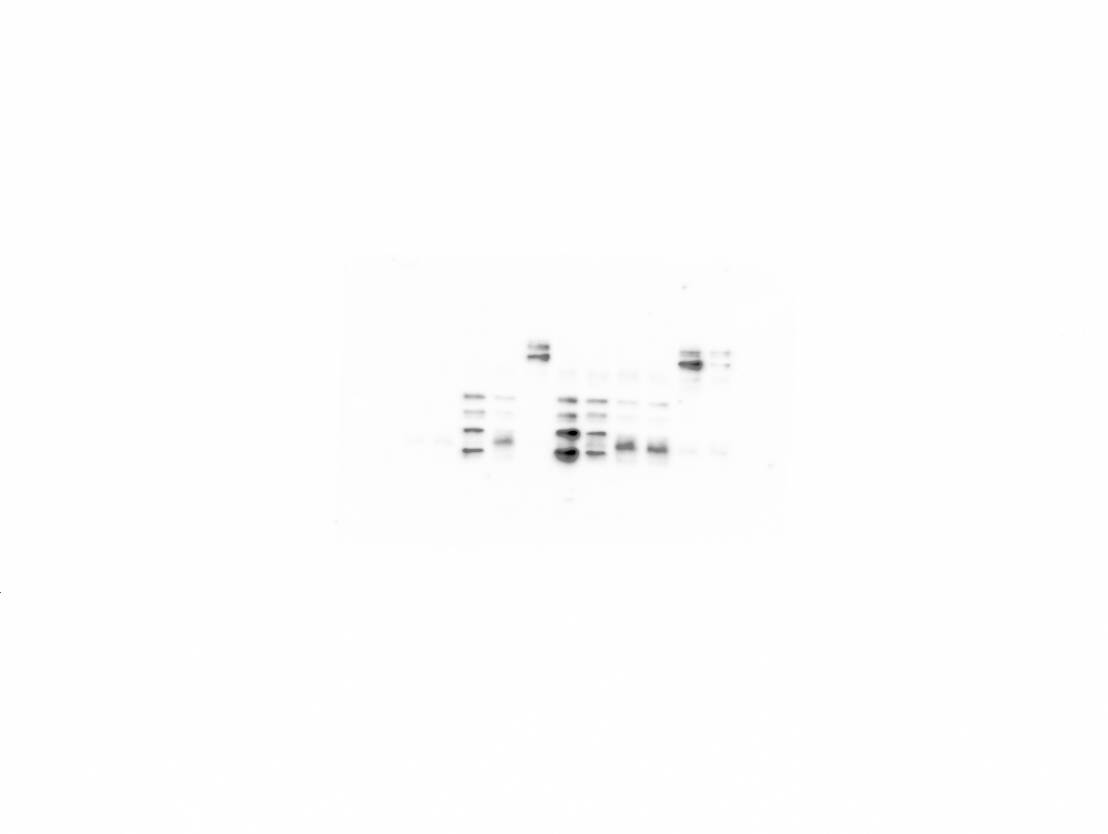

Supplement: Figure 5—figure supplement 1—source data 2. [file elife-84319-fig5-figsupp1-data2.zip › Figure 5ΓÇôSupplementary figure 3-Source Data 2/Supplementary figure 3/Sch9-pSer288/Replica 1.tif]

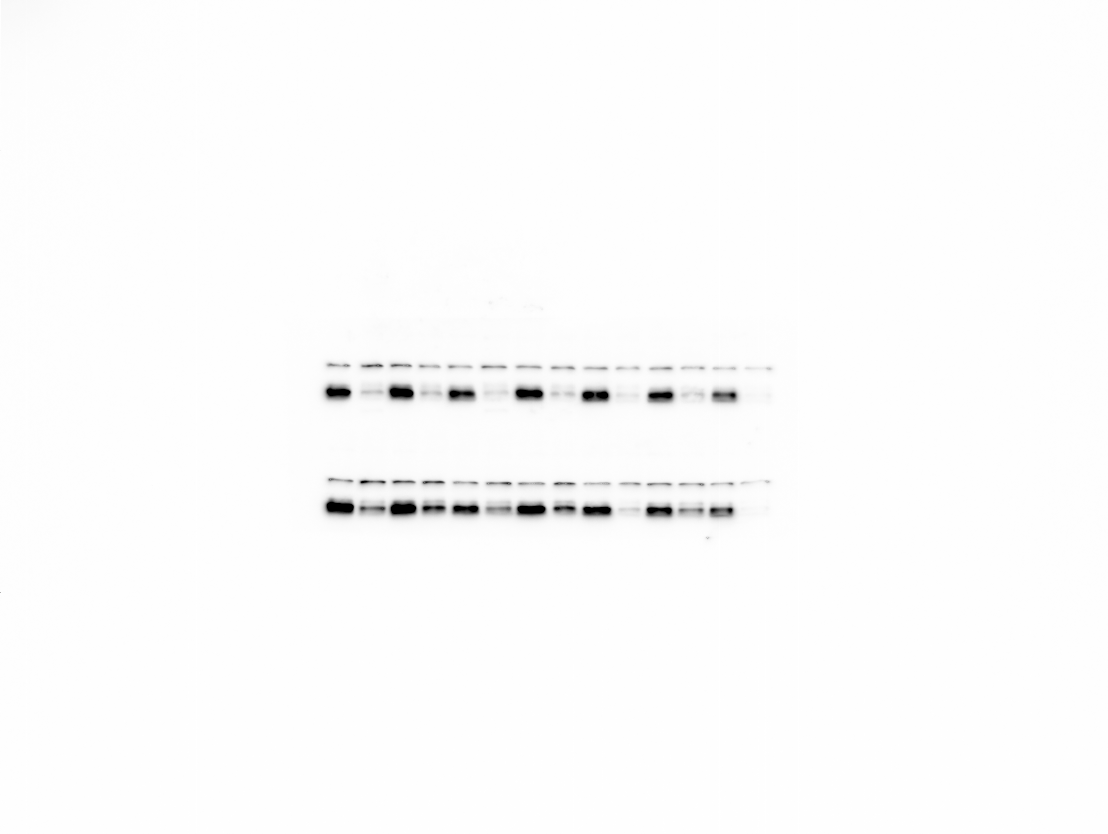

Supplement: Figure 6—source data 3. [file elife-84319-fig6-data3.zip › Figure 6ΓÇôsource data 3/Figure 6A/Sch9-pThr737/Replica 4.tif]

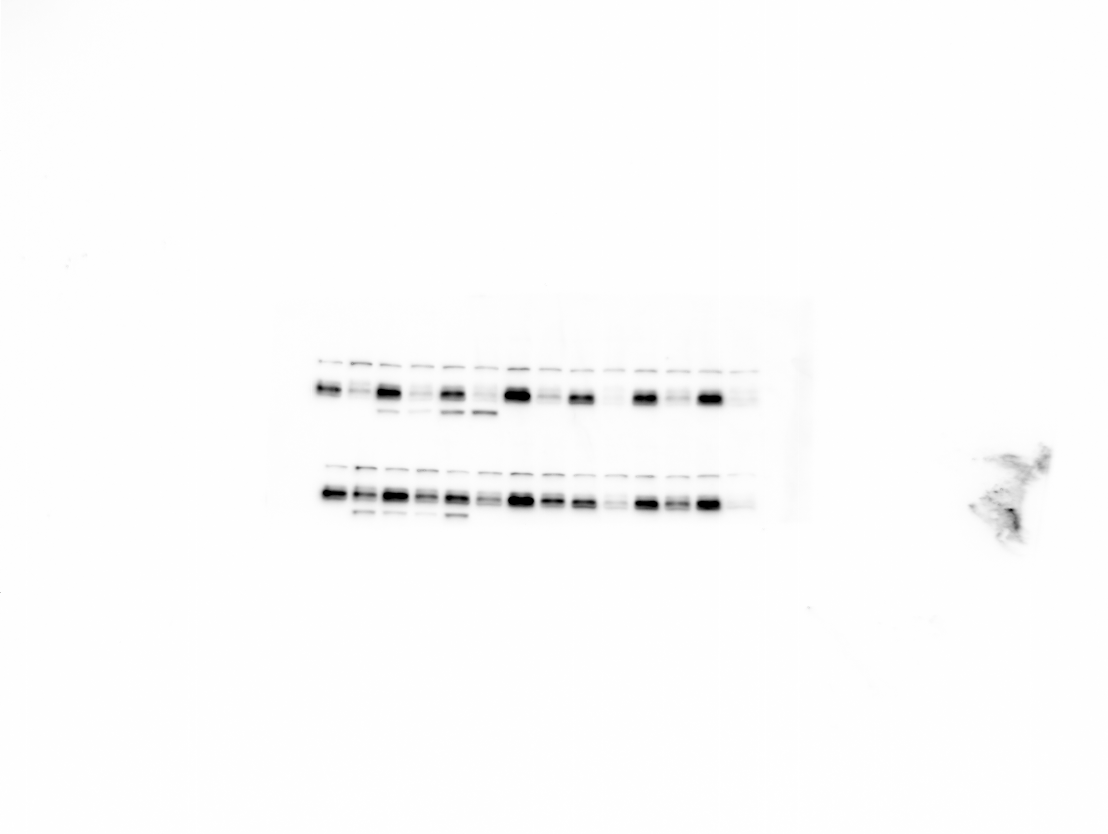

Supplement: Figure 6—source data 3. [file elife-84319-fig6-data3.zip › Figure 6ΓÇôsource data 3/Figure 6A/Sch9-pThr737/Replica 2.tif]

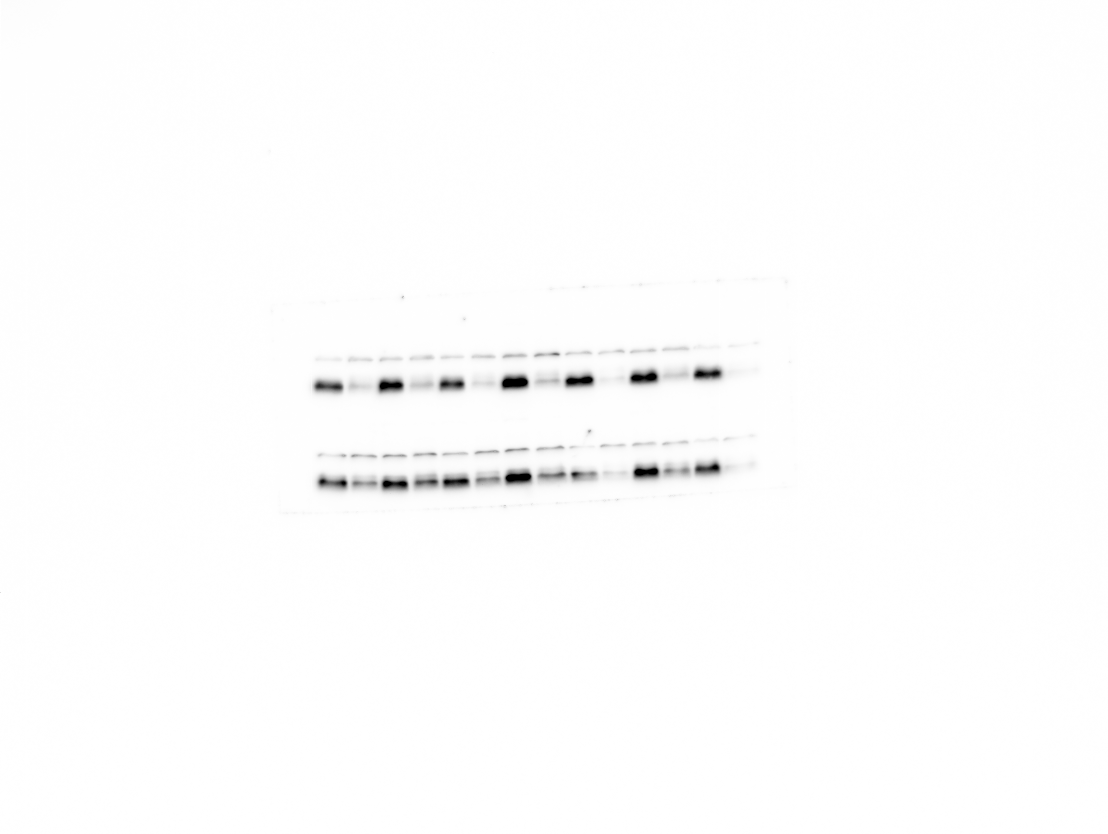

Supplement: Figure 6—source data 3. [file elife-84319-fig6-data3.zip › Figure 6ΓÇôsource data 3/Figure 6A/Sch9-pThr737/Replica 3.tif]

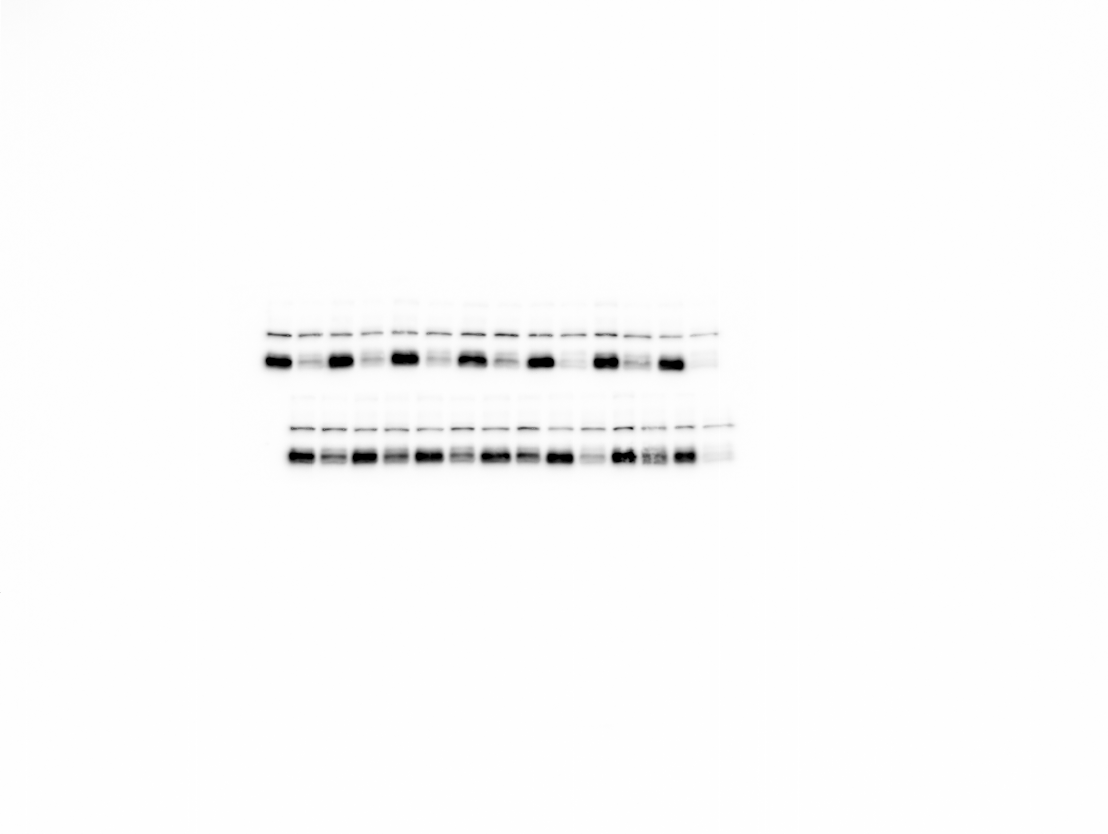

Supplement: Figure 6—source data 3. [file elife-84319-fig6-data3.zip › Figure 6ΓÇôsource data 3/Figure 6A/Sch9-pThr737/Replica 1.tif]

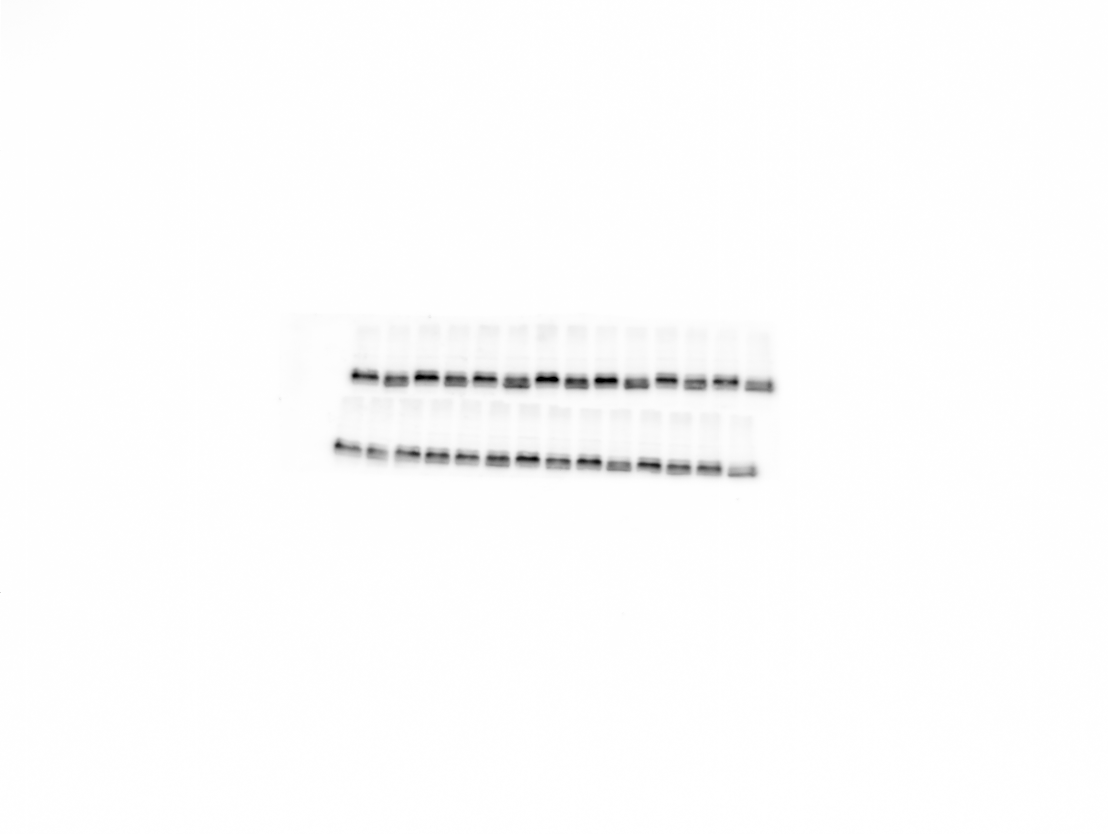

Supplement: Figure 6—source data 3. [file elife-84319-fig6-data3.zip › Figure 6ΓÇôsource data 3/Figure 6A/Sch9/Replica 4.tif]

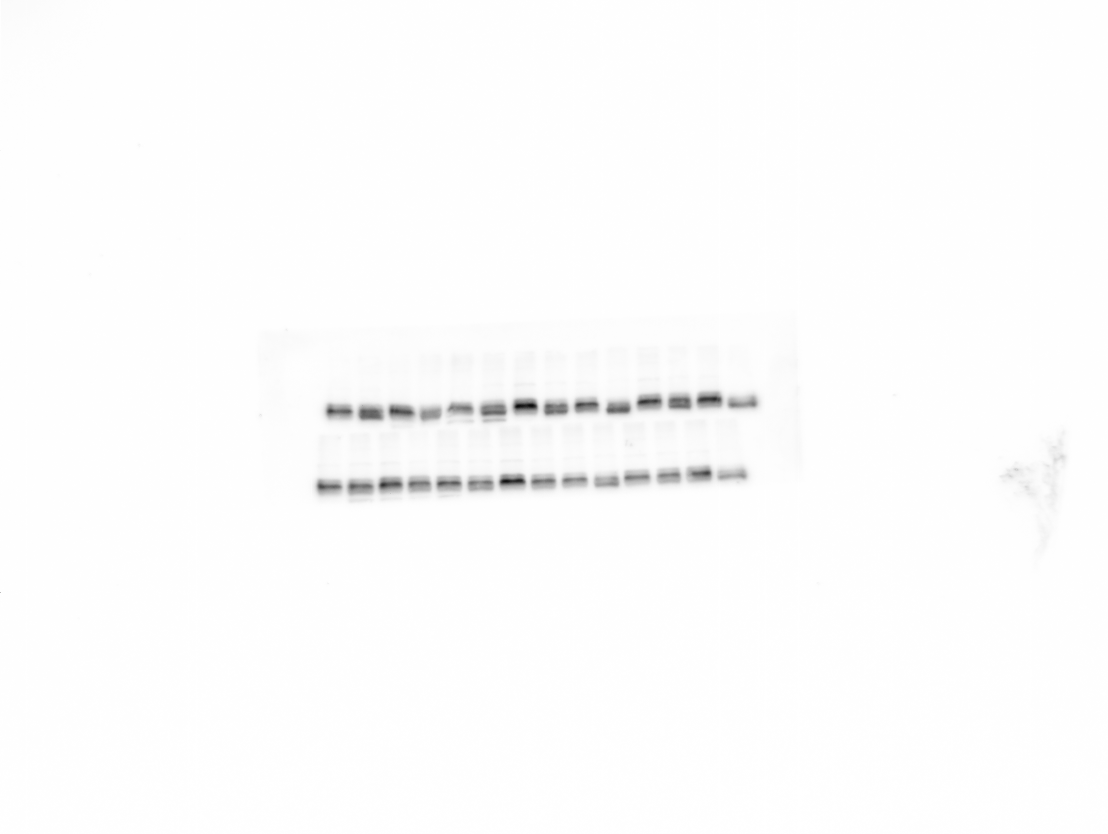

Supplement: Figure 6—source data 3. [file elife-84319-fig6-data3.zip › Figure 6ΓÇôsource data 3/Figure 6A/Sch9/Replica 2.tif]

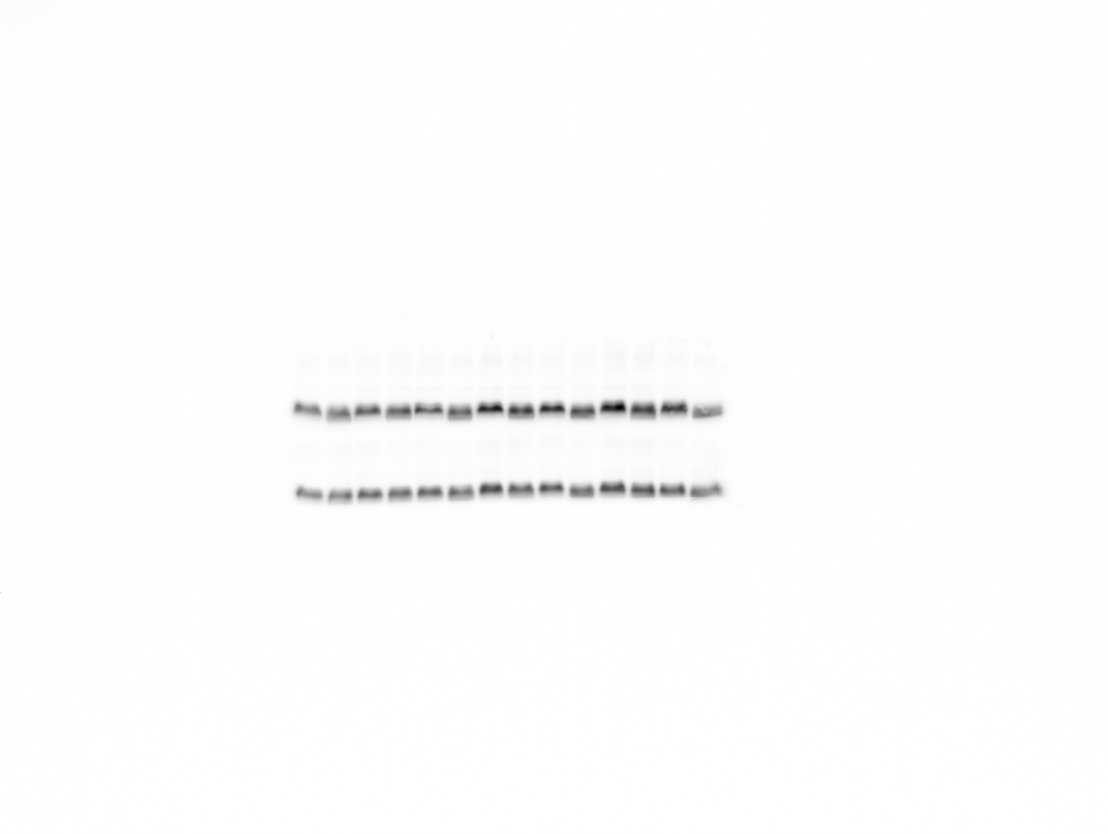

Supplement: Figure 6—source data 3. [file elife-84319-fig6-data3.zip › Figure 6ΓÇôsource data 3/Figure 6A/Sch9/Replica 3.tif]

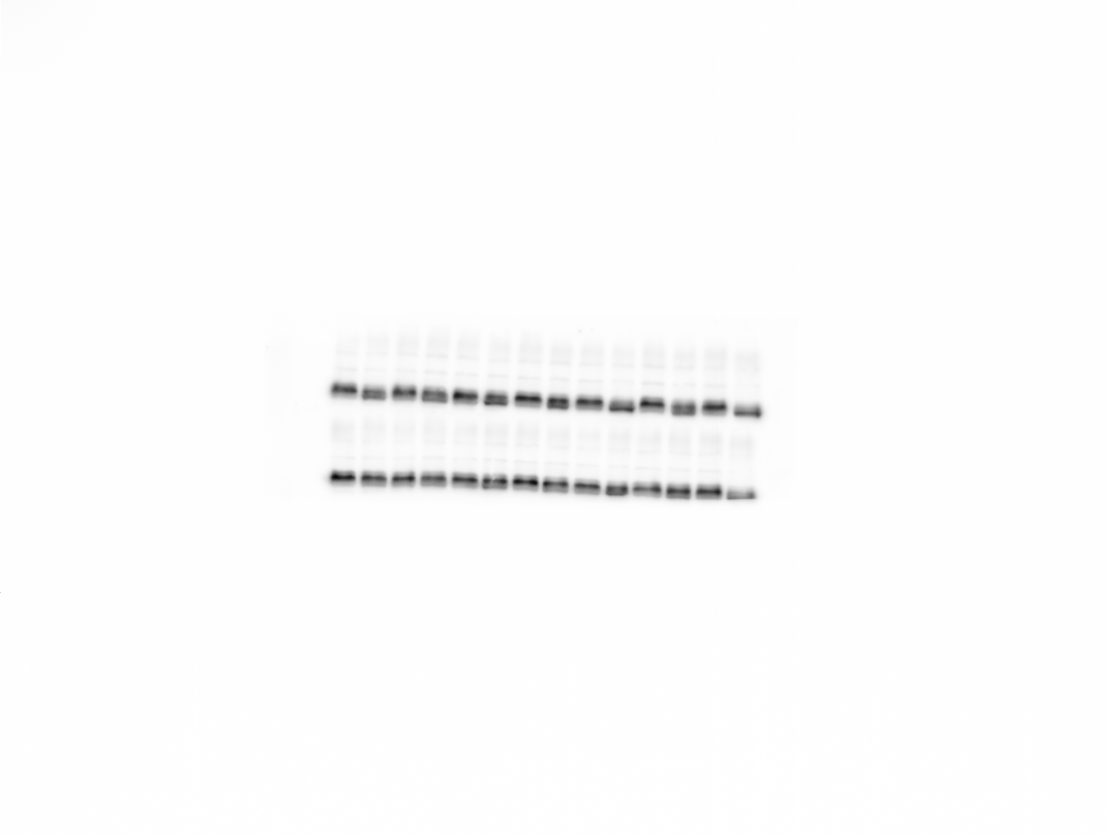

Supplement: Figure 6—source data 3. [file elife-84319-fig6-data3.zip › Figure 6ΓÇôsource data 3/Figure 6A/Sch9/Replica 1.tif]

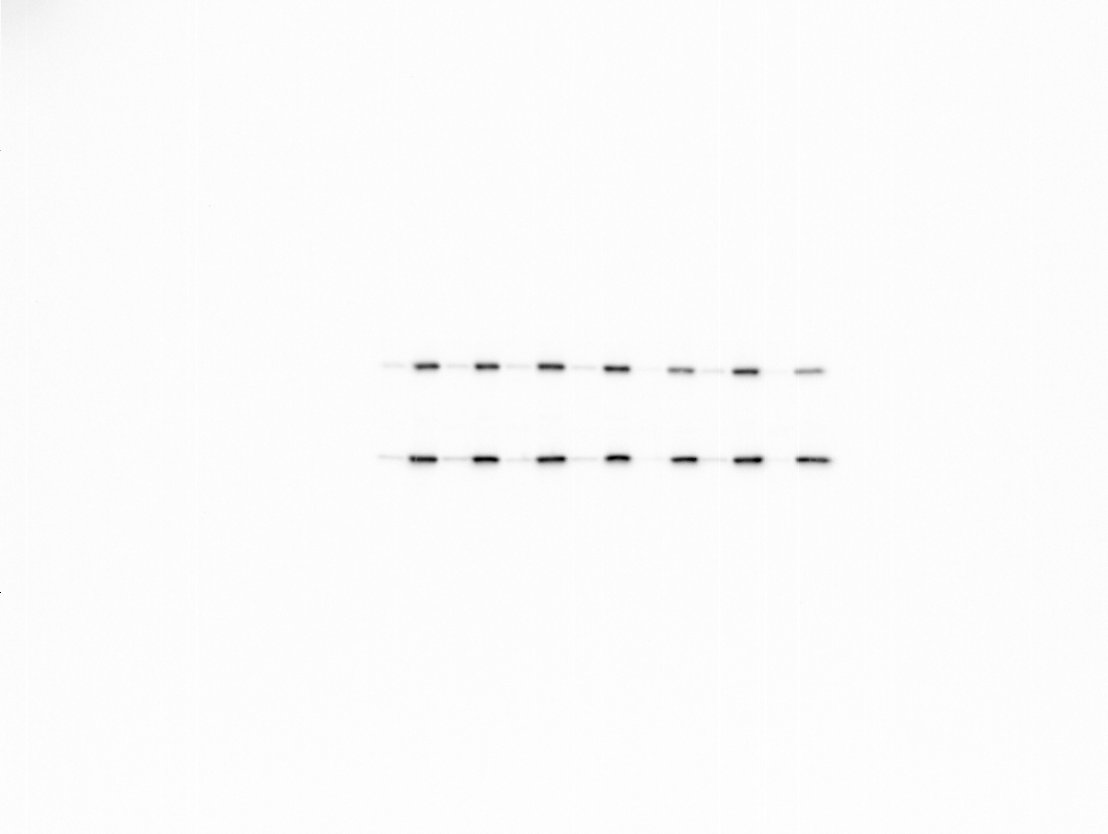

Supplement: Figure 6—source data 3. [file elife-84319-fig6-data3.zip › Figure 6ΓÇôsource data 3/Figure 6A/Snf1-pThr210/Replica 4.tif]

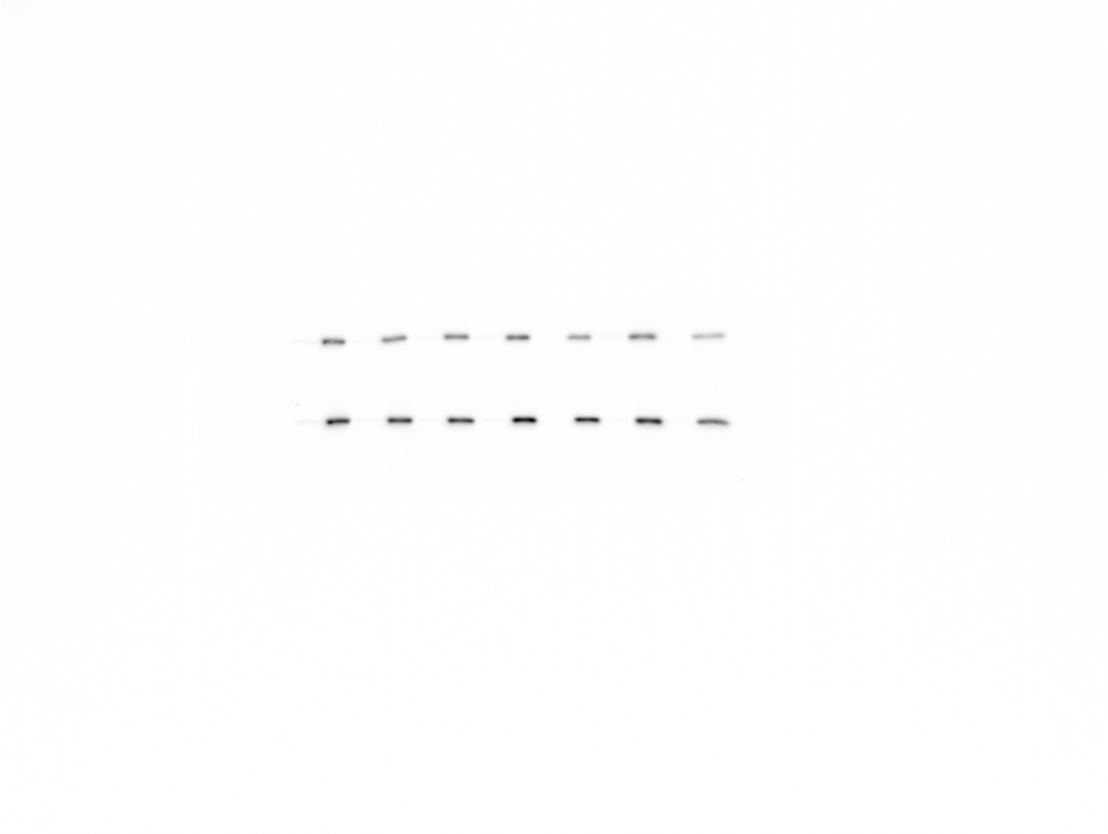

Supplement: Figure 6—source data 3. [file elife-84319-fig6-data3.zip › Figure 6ΓÇôsource data 3/Figure 6A/Snf1-pThr210/Replica 2.tif]

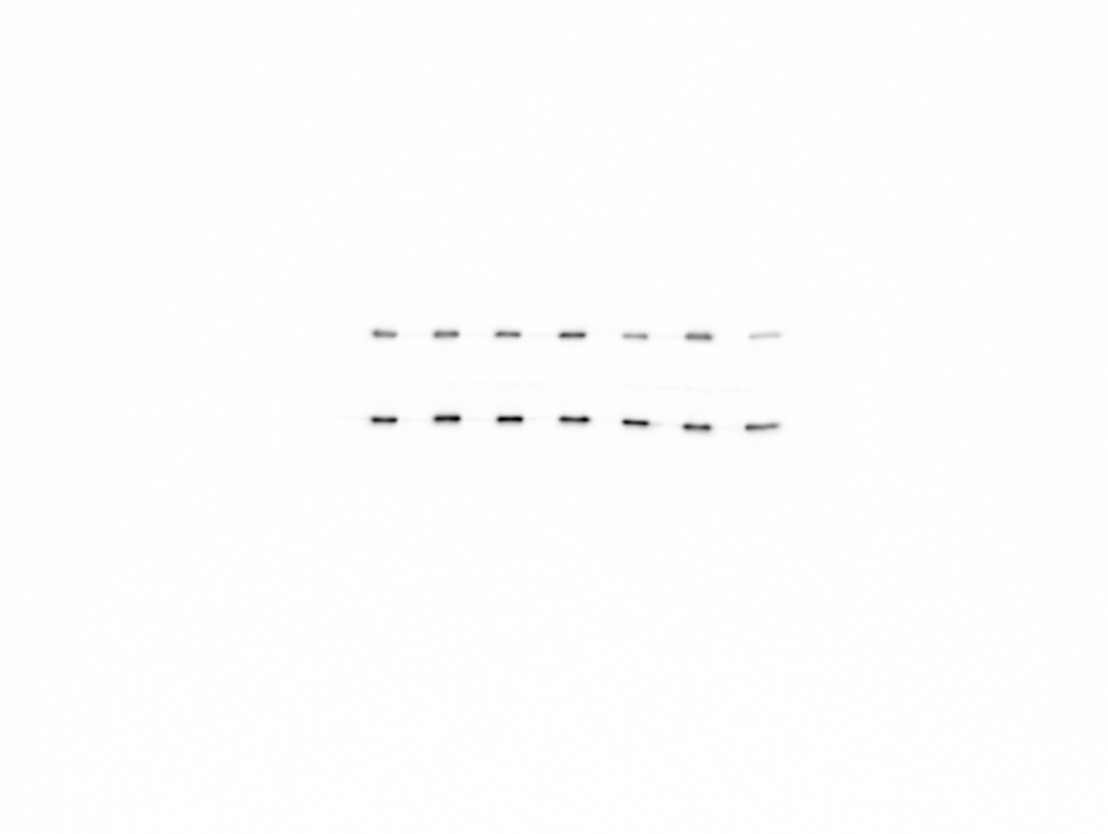

Supplement: Figure 6—source data 3. [file elife-84319-fig6-data3.zip › Figure 6ΓÇôsource data 3/Figure 6A/Snf1-pThr210/Replica 3.tif]

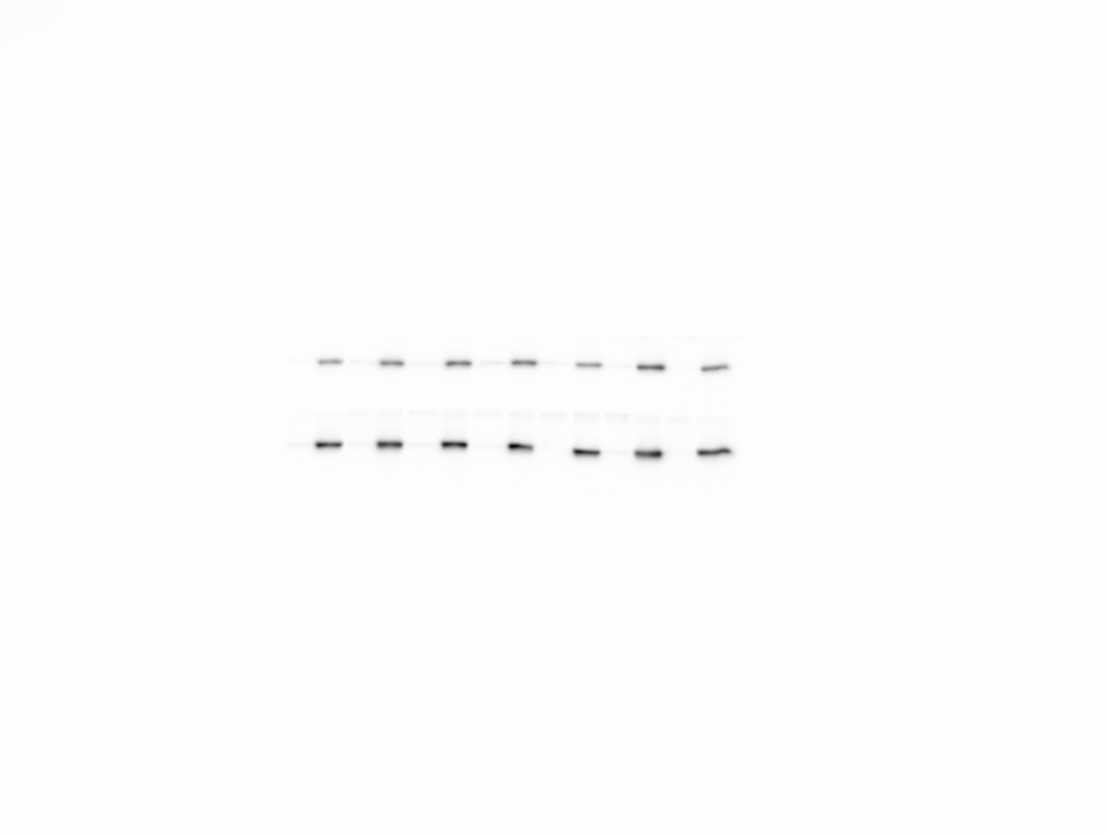

Supplement: Figure 6—source data 3. [file elife-84319-fig6-data3.zip › Figure 6ΓÇôsource data 3/Figure 6A/Snf1-pThr210/Replica 1.tif]

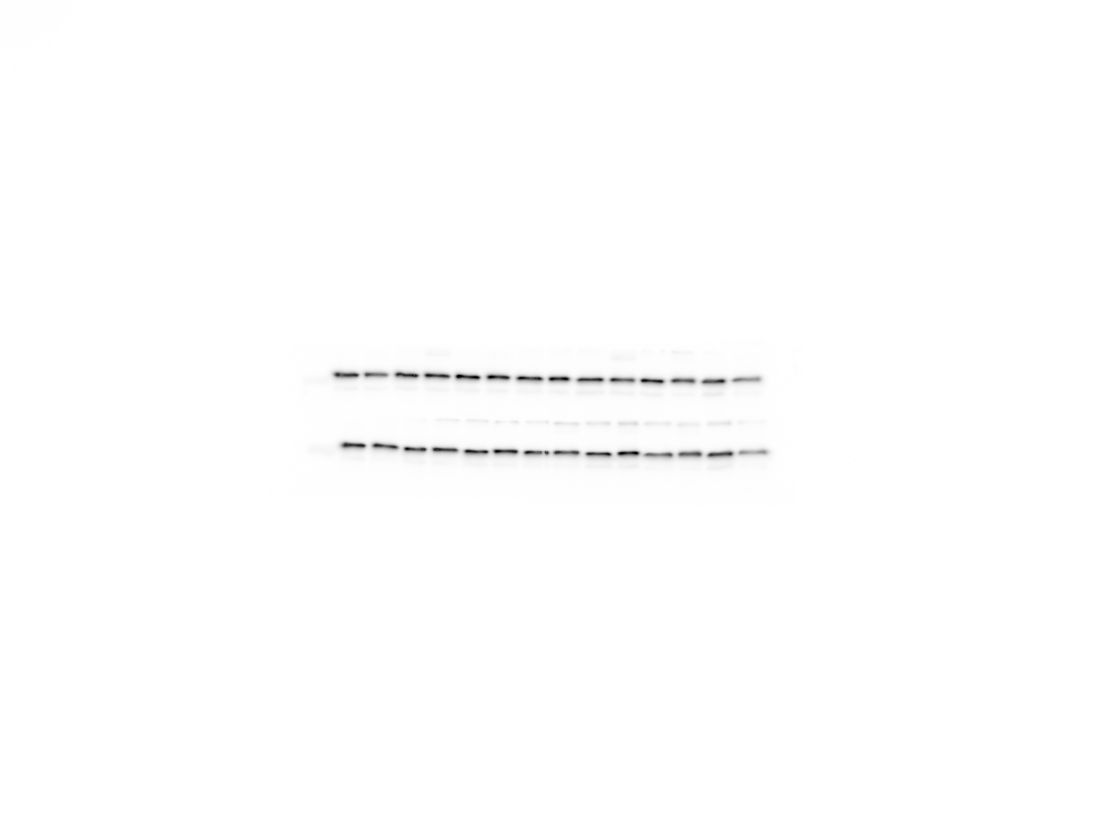

Supplement: Figure 6—source data 3. [file elife-84319-fig6-data3.zip › Figure 6ΓÇôsource data 3/Figure 6A/His6/Replica 4.tif]

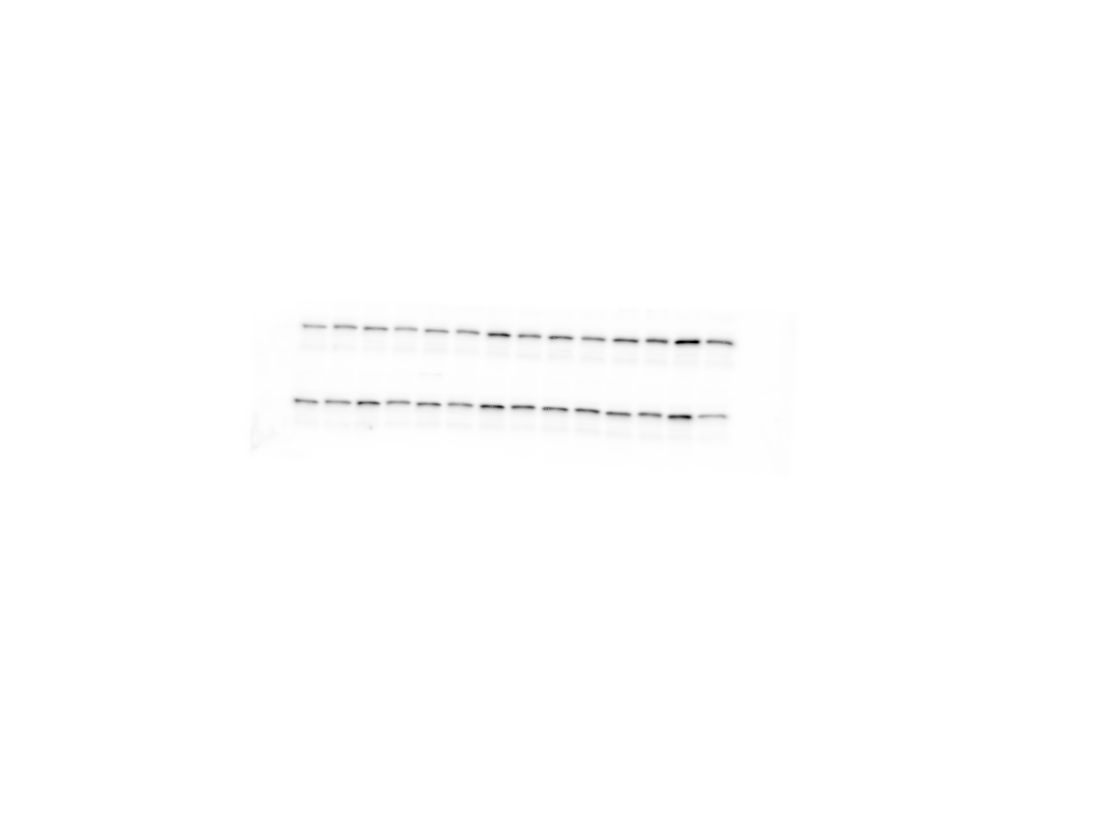

Supplement: Figure 6—source data 3. [file elife-84319-fig6-data3.zip › Figure 6ΓÇôsource data 3/Figure 6A/His6/Replica 2.tif]

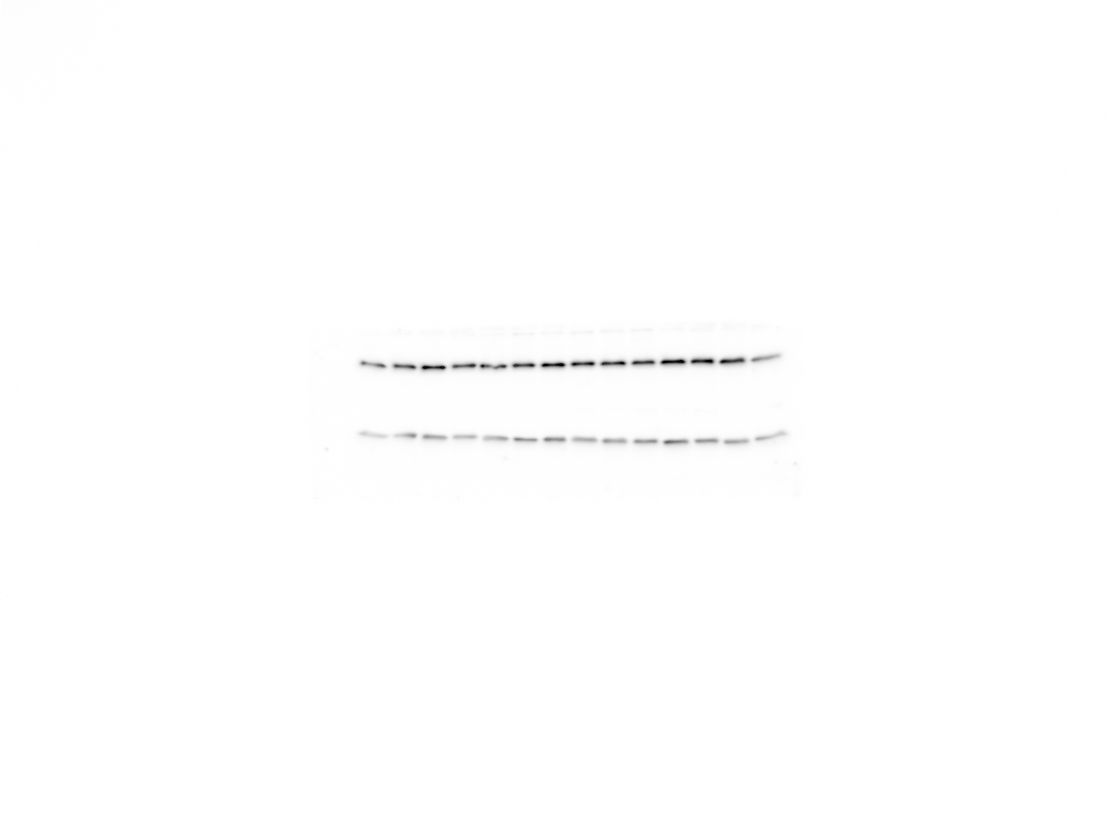

Supplement: Figure 6—source data 3. [file elife-84319-fig6-data3.zip › Figure 6ΓÇôsource data 3/Figure 6A/His6/Replica 3.tif]

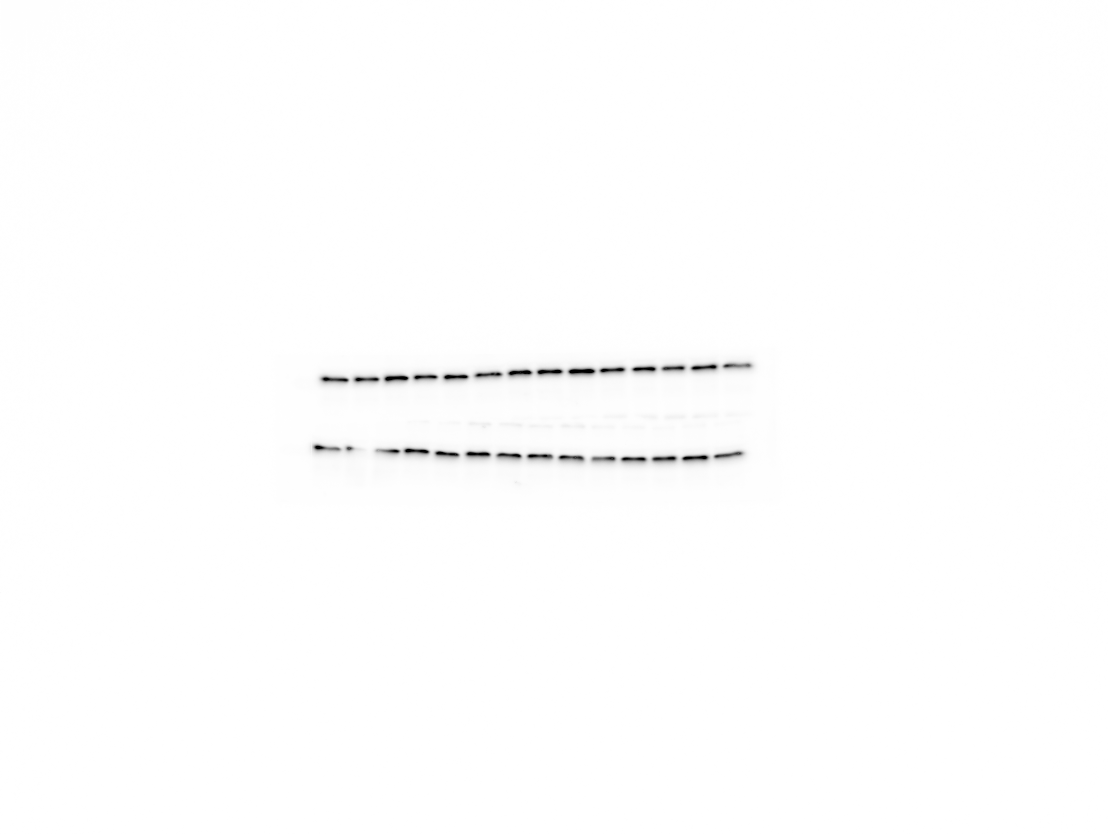

Supplement: Figure 6—source data 3. [file elife-84319-fig6-data3.zip › Figure 6ΓÇôsource data 3/Figure 6A/His6/Replica 1.tif]

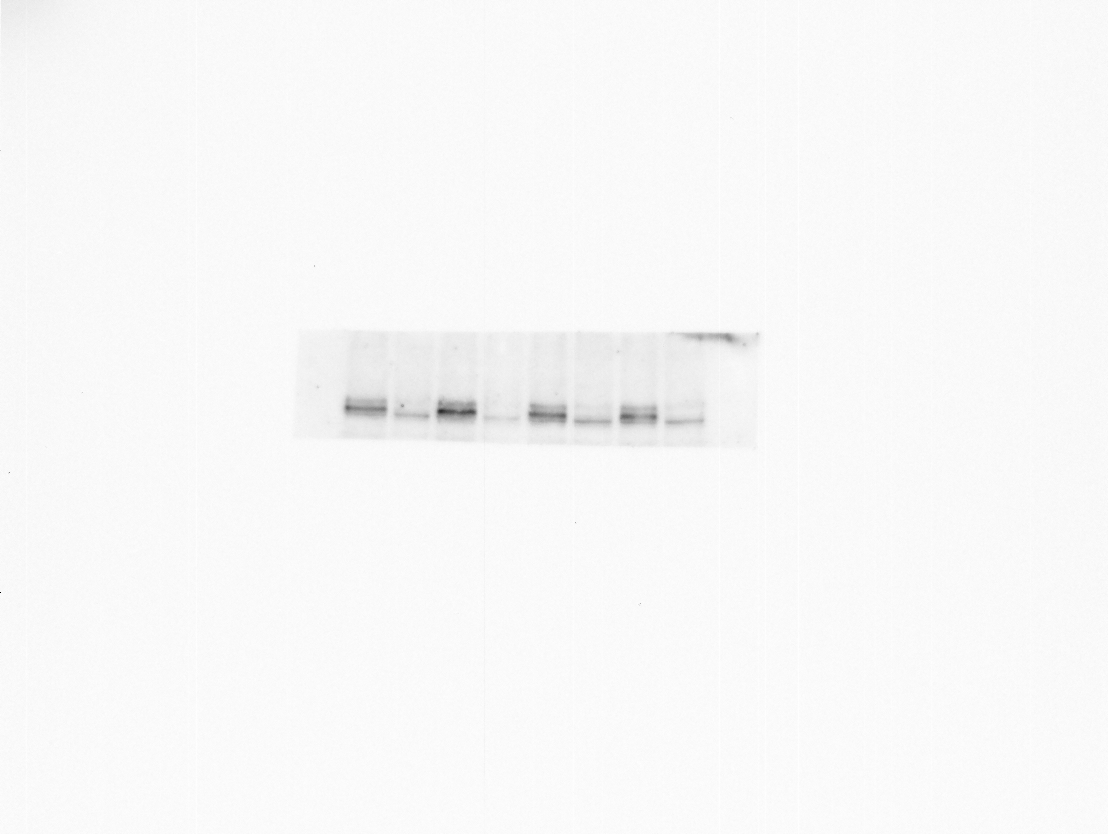

Supplement: Figure 6—source data 3. [file elife-84319-fig6-data3.zip › Figure 6ΓÇôsource data 3/Figure 6C/Sch9-pThr737/Replica 5_6_7_8.tif]

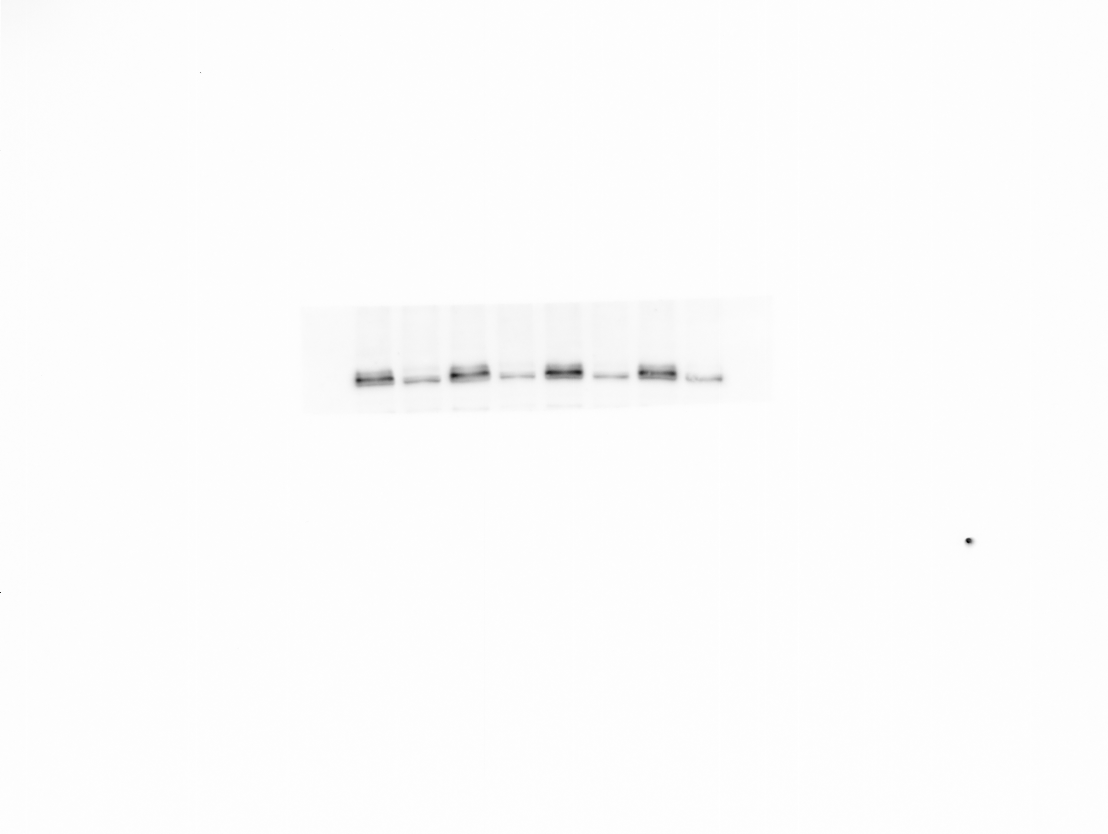

Supplement: Figure 6—source data 3. [file elife-84319-fig6-data3.zip › Figure 6ΓÇôsource data 3/Figure 6C/Sch9-pThr737/Replica 1_2_3_4.tif]

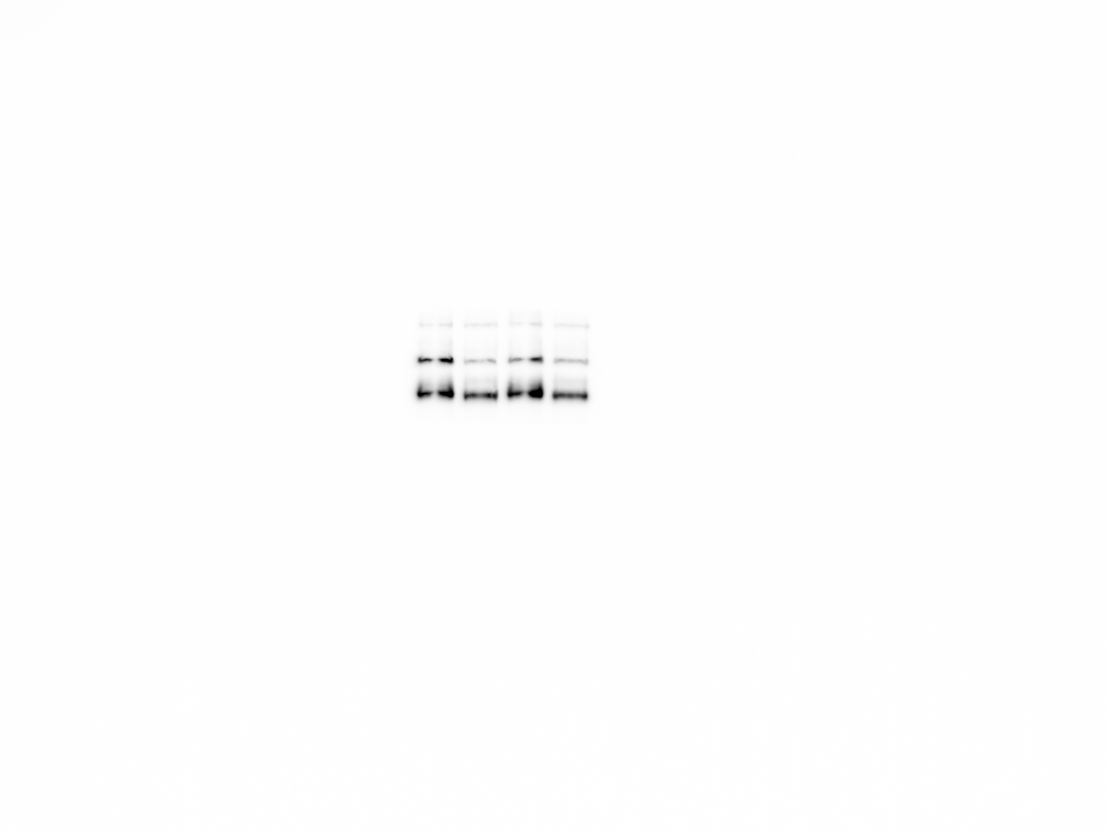

Supplement: Figure 6—source data 3. [file elife-84319-fig6-data3.zip › Figure 6ΓÇôsource data 3/Figure 6C/Sch9-pThr737/Replica 9_10.tif]

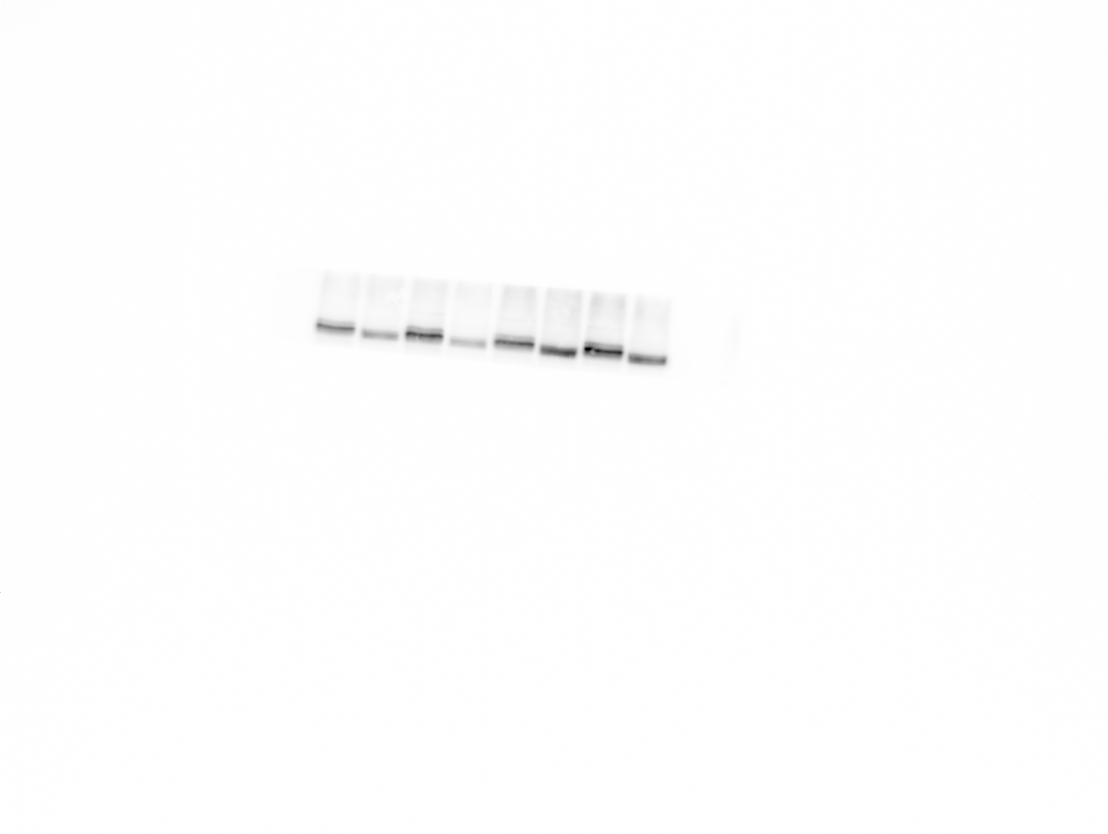

Supplement: Figure 6—source data 3. [file elife-84319-fig6-data3.zip › Figure 6ΓÇôsource data 3/Figure 6C/Sch9/Replica 5_6_7_8.tif]

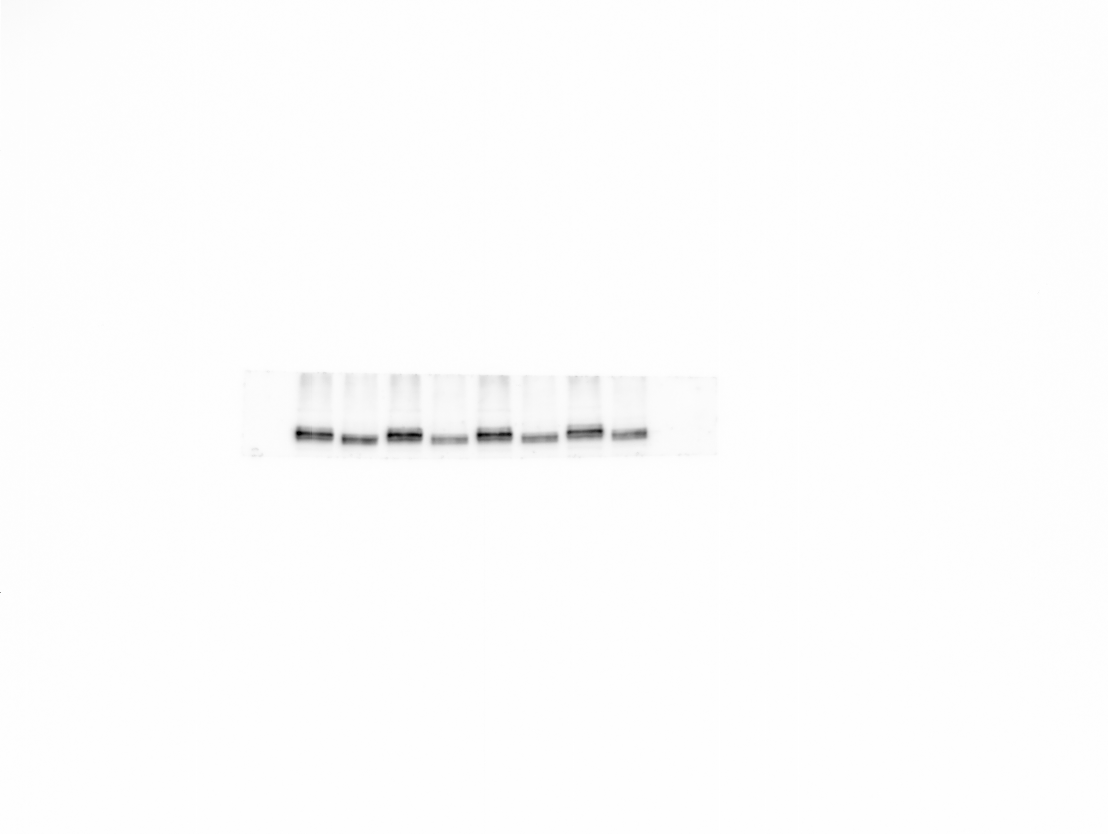

Supplement: Figure 6—source data 3. [file elife-84319-fig6-data3.zip › Figure 6ΓÇôsource data 3/Figure 6C/Sch9/Replica 1_2_3_4.tif]

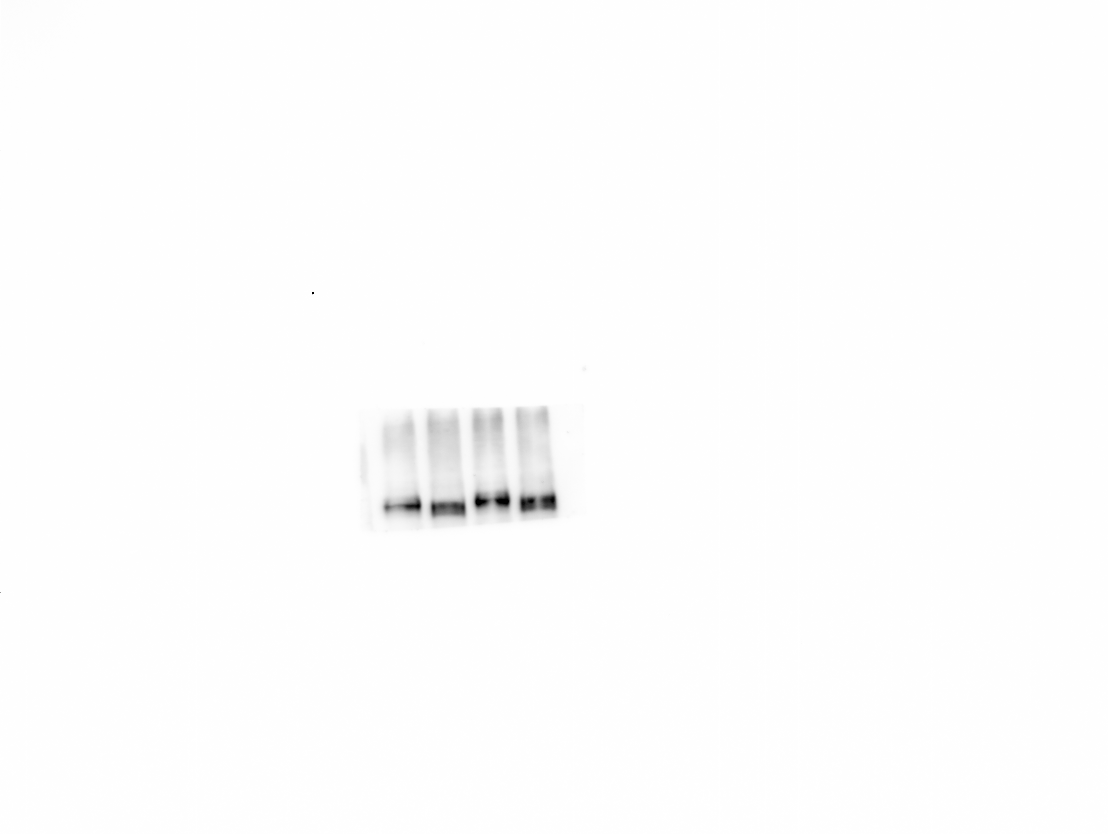

Supplement: Figure 6—source data 3. [file elife-84319-fig6-data3.zip › Figure 6ΓÇôsource data 3/Figure 6C/Sch9/Replica 9_10.tif]

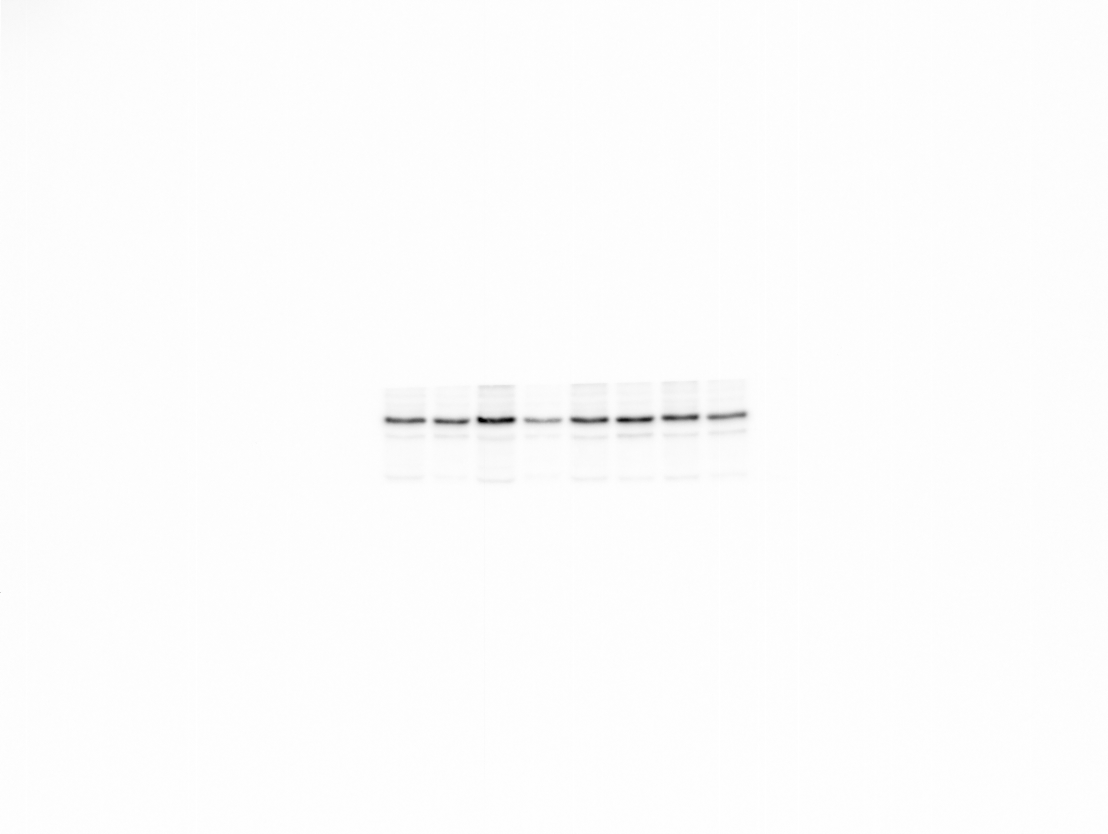

Supplement: Figure 6—source data 3. [file elife-84319-fig6-data3.zip › Figure 6ΓÇôsource data 3/Figure 6C/Snf1-pThr210/Replica 5_6_7_8.tif]

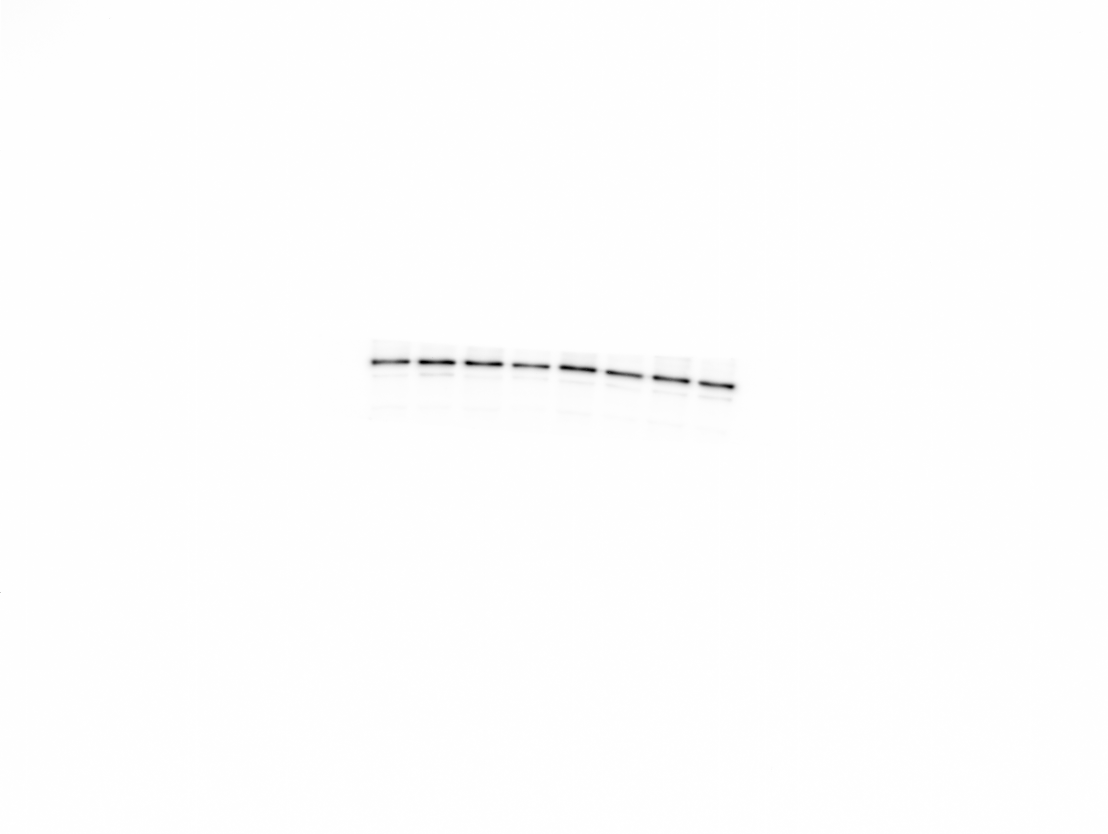

Supplement: Figure 6—source data 3. [file elife-84319-fig6-data3.zip › Figure 6ΓÇôsource data 3/Figure 6C/Snf1-pThr210/Replica 1_2_3_4.tif]

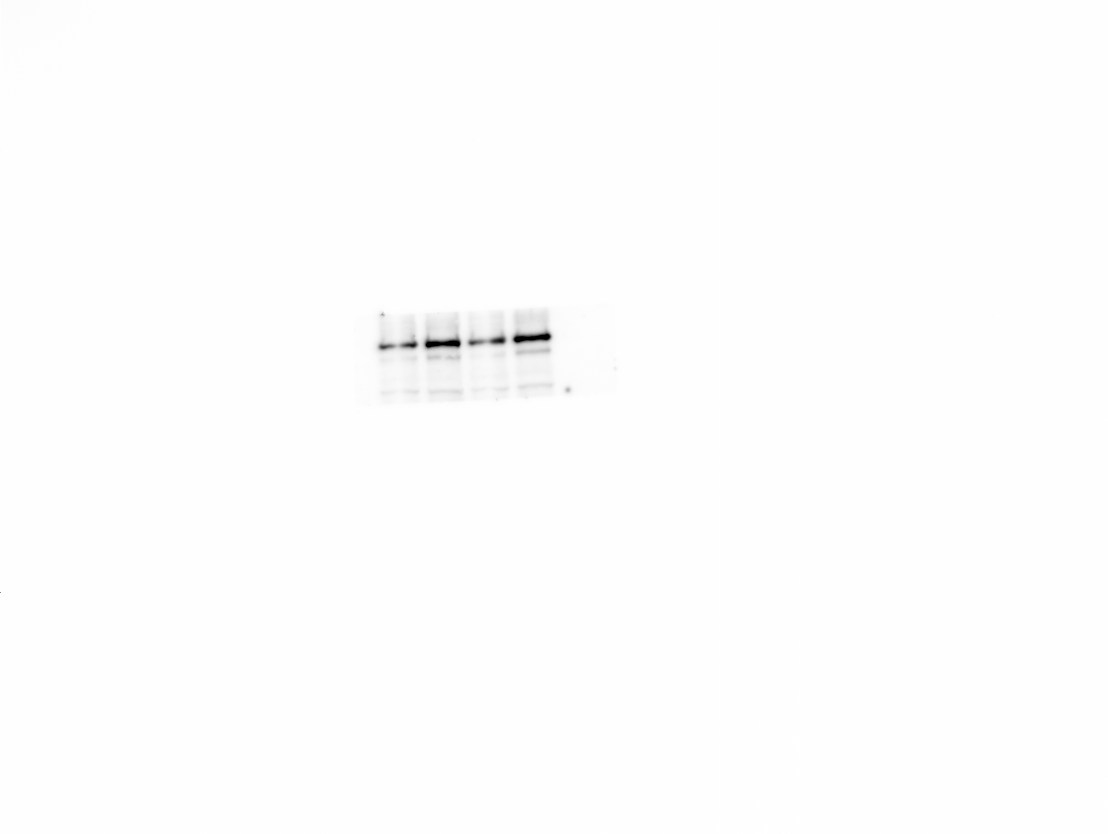

Supplement: Figure 6—source data 3. [file elife-84319-fig6-data3.zip › Figure 6ΓÇôsource data 3/Figure 6C/Snf1-pThr210/Replica 9_10.tif]

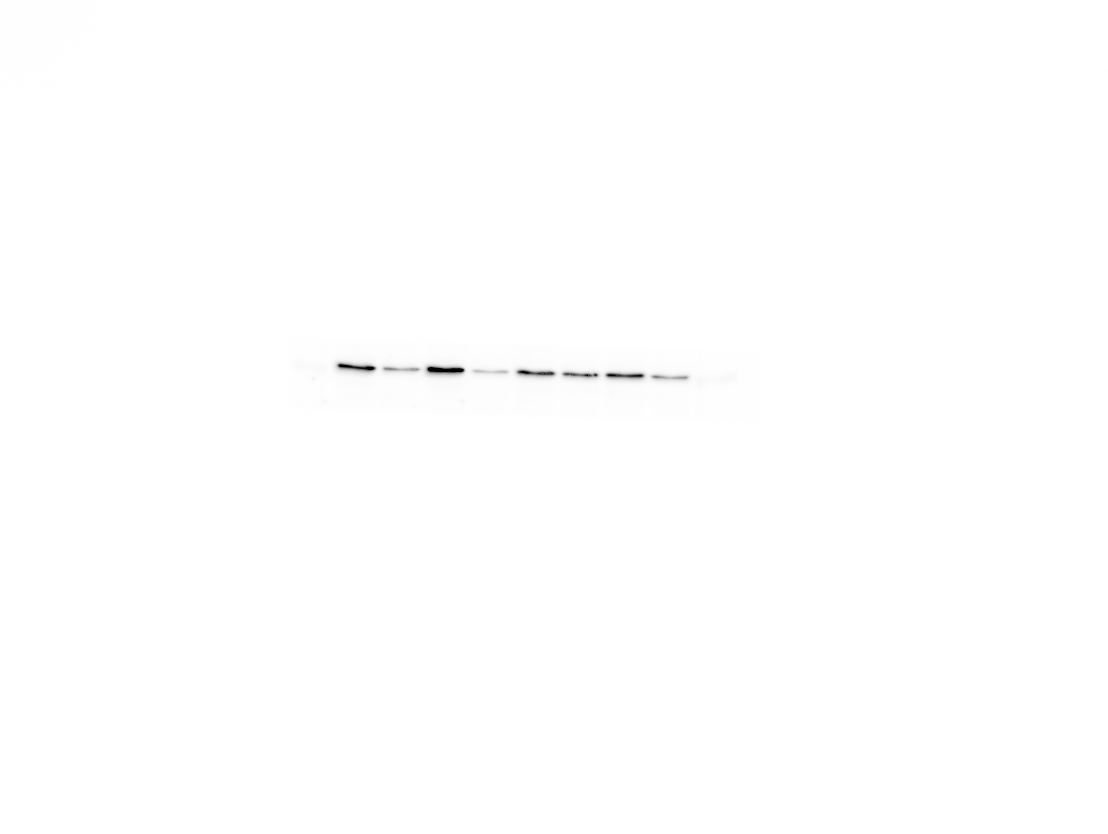

Supplement: Figure 6—source data 3. [file elife-84319-fig6-data3.zip › Figure 6ΓÇôsource data 3/Figure 6C/His6/Replica 5_6_7_8.tif]

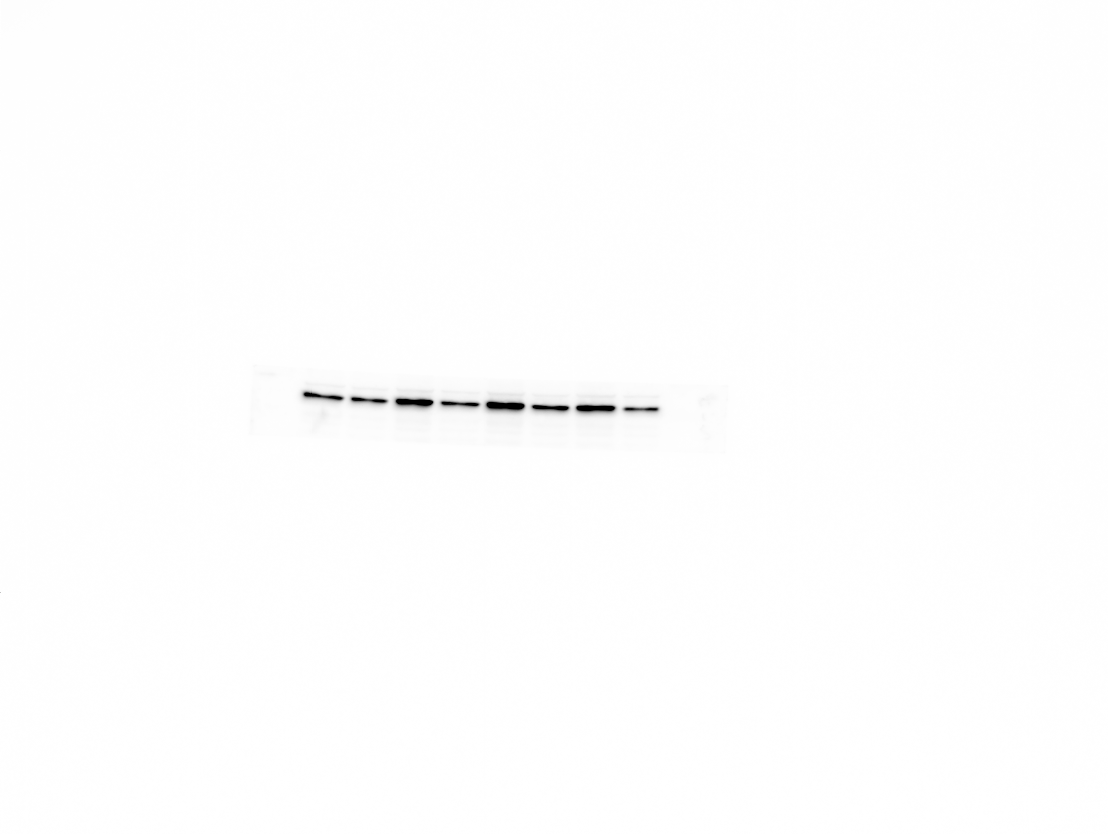

Supplement: Figure 6—source data 3. [file elife-84319-fig6-data3.zip › Figure 6ΓÇôsource data 3/Figure 6C/His6/Replica 1_2_3_4.tif]

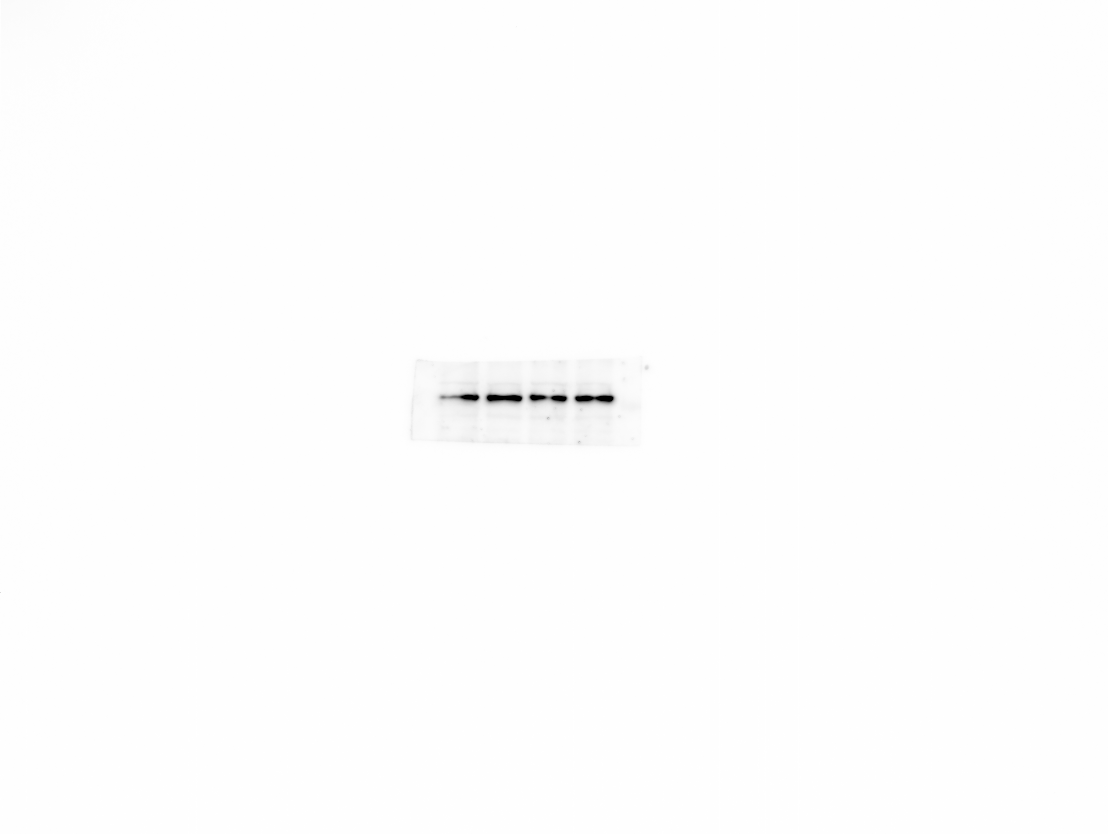

Supplement: Figure 6—source data 3. [file elife-84319-fig6-data3.zip › Figure 6ΓÇôsource data 3/Figure 6C/His6/Replica 9_10.tif]
