# Supplementary material for: Snf1/AMPK fine-tunes TORC1 signaling in response to glucose starvation
Source: eLife. 2023 Feb 7;12:e84319. doi: 10.7554/eLife.84319 (PMC9937656; doi:10.7554/eLife.84319)
Supplement: Figure 5—source data 2. [file elife-84319-fig5-data2.pdf]

Figure 5C

Loading order:

	^{32}P						Sypro Ruby					
Sch9$^{1\text{-}394}$	-		WT		S288A		-		WT		S288A	
Snf1	WT	TA	WT	TA	WT	TA	WT	TA	WT	TA	WT	TA

^{32}P Autoradiography

Replicate 1

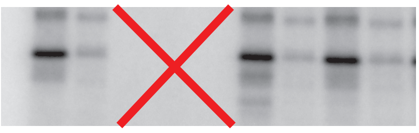

Replicate 2

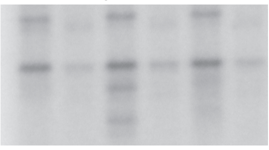

Replicate 3 (Data shown in Figure 5 C)

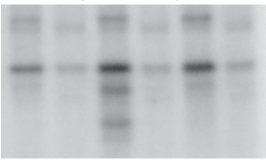

Sypro Ruby

Replicate 1

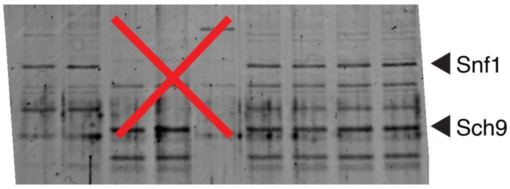

Replicate 2

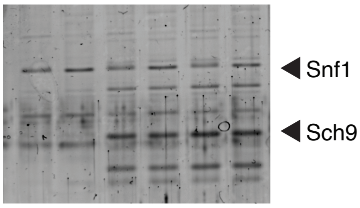

Replicate 3 (Data shown in Figure 5 C)

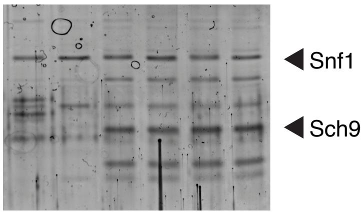

Replica 4 (Data shown in Figure 6A)

Figure 5D

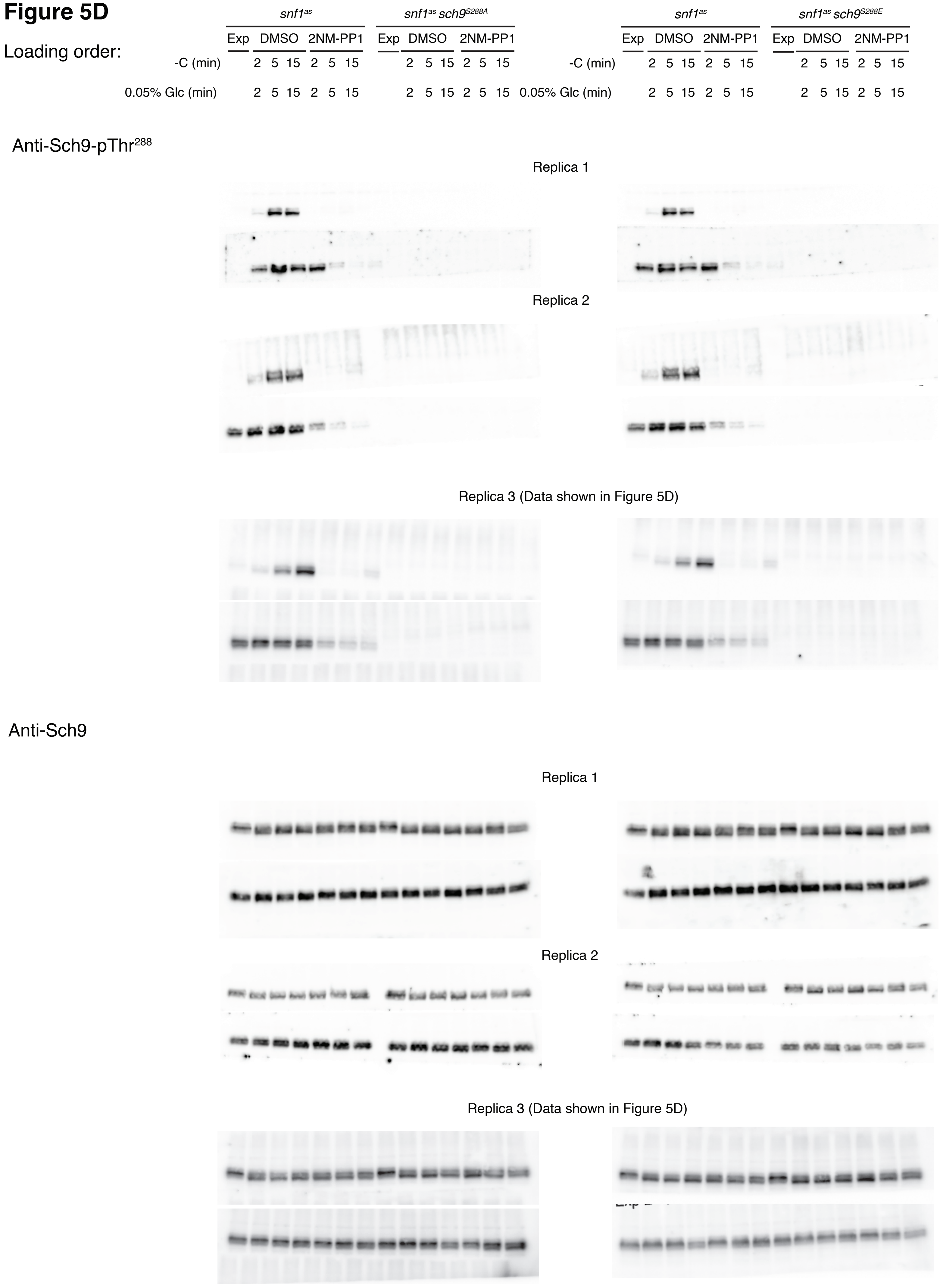
Loading order:
snf1as
snf1as sch9S288A
snf1as
snf1as sch9S288E
Exp DMSO 2NM-PP1 Exp DMSO 2NM-PP1
-C (min) 2 5 15 2 5 15 2 5 15 2 5 15
0.05% Glc (min) 2 5 15 2 5 15 2 5 15 2 5 15
-C (min) 2 5 15 2 5 15 2 5 15 2 5 15
0.05% Glc (min) 2 5 15 2 5 15 2 5 15 2 5 15
Anti-Sch9-pThr288
Replica 1
Replica 2
Replica 3 (Data shown in Figure 5D)
Anti-Sch9
Replica 1
Replica 2
Replica 3 (Data shown in Figure 5D)

Figure 5F

		*snf1^as^*		*snf1^as^ sch9^SA^*		*snf1^as^ sch9^SE^*	
		Exp	-C	Exp	-C	Exp	-C
Loading order:	2NM-PP1		- +		- +		- +

Anti-Sch9-pThr737

Replica 1

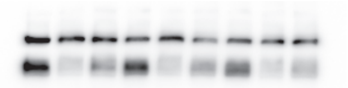

Replica 2

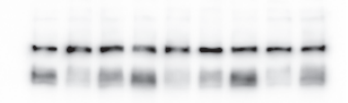

Replica 3

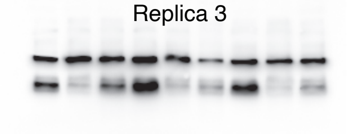

Replica 4

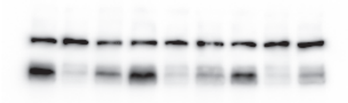

Replica 5

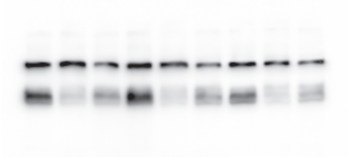

Replica 6 (Data shown in Figure 5F)

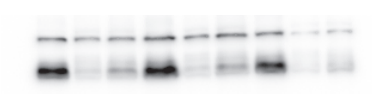

Anti-Sch9

Replica 1

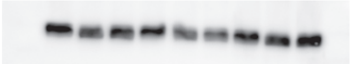

Replica 2

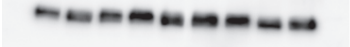

Replica 3

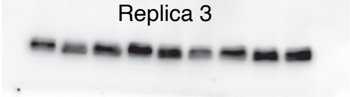

Replica 4

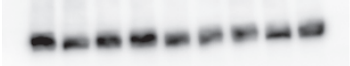

Replica 5

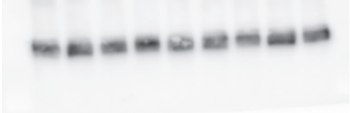

Replica 6 (Data shown in Figure 5F)

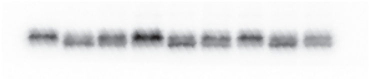

Anti-Snf1-pThr210

Replica 1

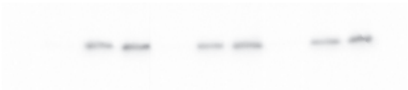

Replica 6 (Data shown in Figure 5F)

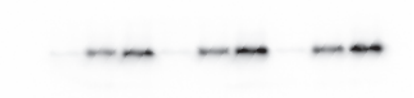

Anti-His$_6$

Replica 1

Replica 6 (Data shown in Figure 5F)
