# Supplementary material for: Snf1/AMPK fine-tunes TORC1 signaling in response to glucose starvation
Source: eLife. 2023 Feb 7;12:e84319. doi: 10.7554/eLife.84319 (PMC9937656; doi:10.7554/eLife.84319)

Figure 5-figure supplement 1

Loading order:

Snf1	WT	TA	-	-	-	WT	TA	WT	TA	WT	TA
Sch9$^{1-394}$	-	-	WT	SA	-	WT	WT	SA	SA	-	-
Sch9	-	-	-	-	KD	-	-	-	-	KD	KD

Anti-Sch9-pSer^{288}

Replica 1

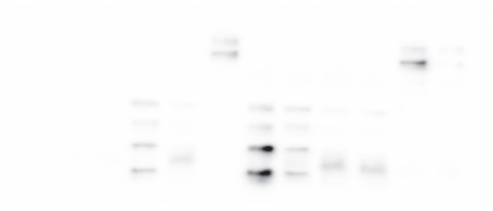

Replica 2 (Data shown in Figure 5-figure supplement 1)

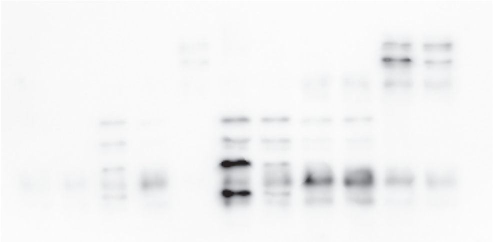

Anti-Sch9

Replica 1

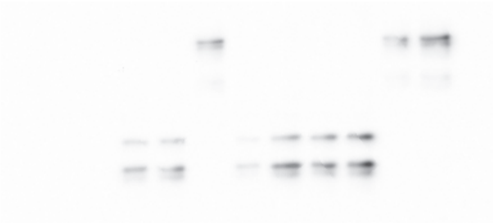

Replica 2 (Data shown in Figure 5-figure supplement 1)

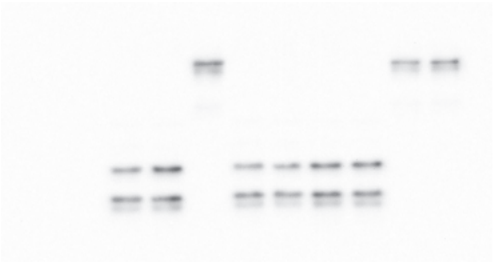

Supplement: Figure 5—figure supplement 1—source data 1. [file elife-84319-fig5-figsupp1-data1.pdf]
