# Supplementary material for: Snf1/AMPK fine-tunes TORC1 signaling in response to glucose starvation
Source: eLife. 2023 Feb 7;12:e84319. doi: 10.7554/eLife.84319 (PMC9937656; doi:10.7554/eLife.84319)

Figure 6A

Loading order:

	snf1^as						
Pib2 variant	**WT**	**WT**	**WT**	**SASA**	**SESE**	**SASA**	**SESE**
Sch9 variant	**WT**	**SA**	**SE**	**WT**	**WT**	**SA**	**SE**
	E -C	E -C	E -C	E -C	E -C	E -C	E -C
2NM-PP1	-	-	-	-	-	-	-
	E -C	E -C	E -C	E -C	E -C	E -C	E -C
2NM-PP1	+	+	+	+	+	+	+

Anti-Sch9-pThr^{737}

Replica 1

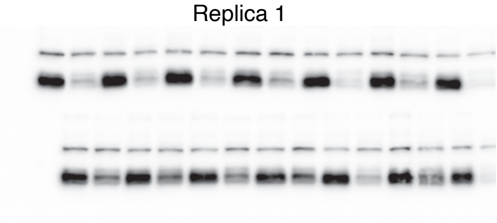

Replica 2

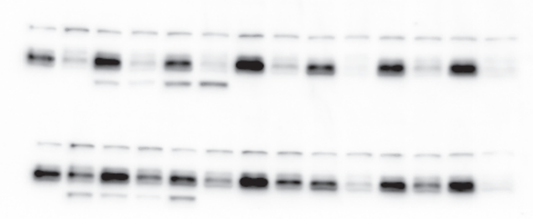

Replica 3

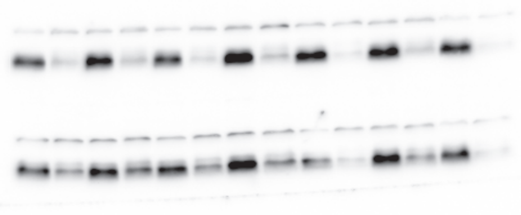

Replica 4 (Data shown in Figure 6A)

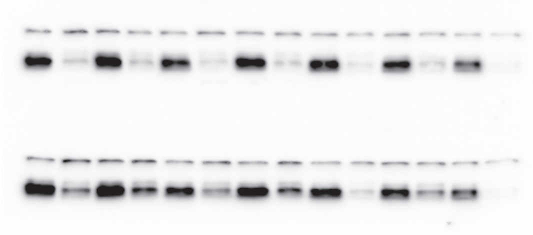

Anti-Sch9

Replica 1

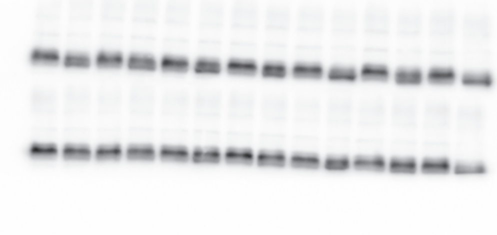

Replica 2

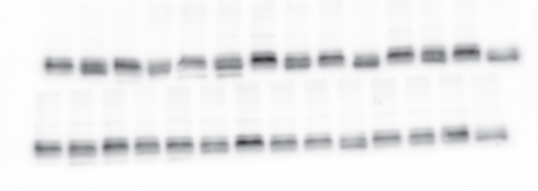

Replica 3

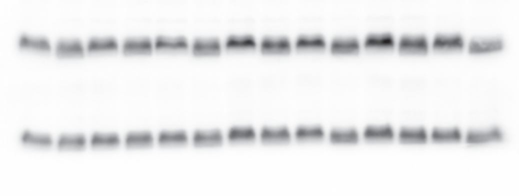

Replica 4 (Data shown in Figure 6A)

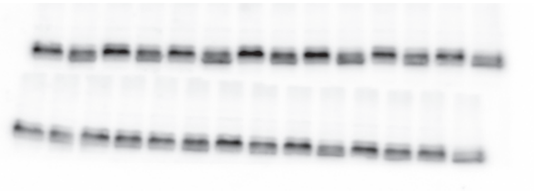

Anti-Snf1-pThr^{210}

Replica 1

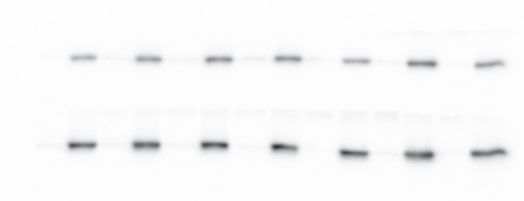

Replica 2

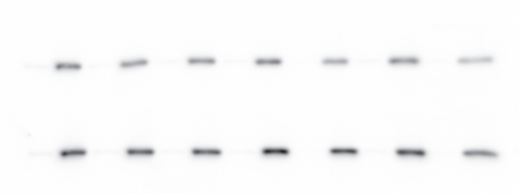

Replica 3

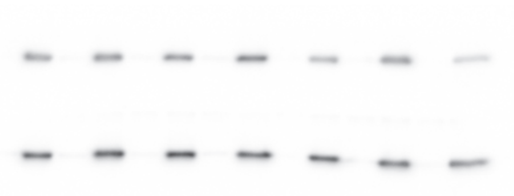

Replica 4 (Data shown in Figure 6A)

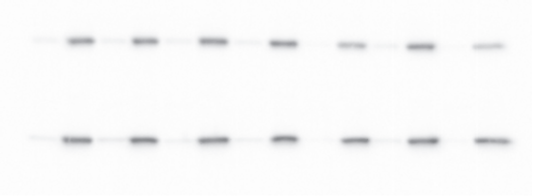

Anti-His$_6$

Replica 1

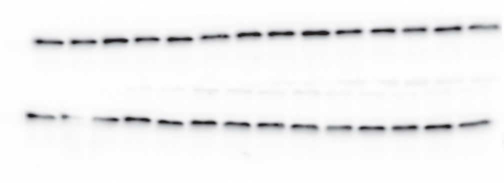

Replica 2

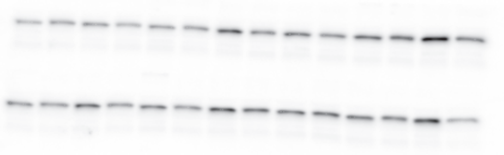

Replica 3

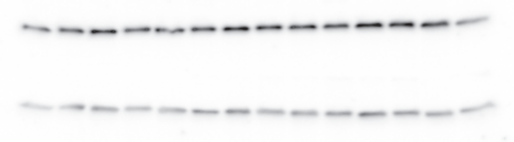

Replica 4 (Data shown in Figure 6A)

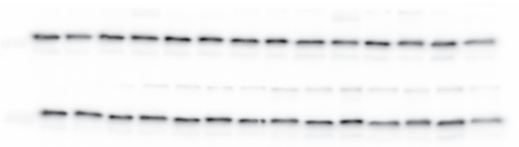

Figure 6C

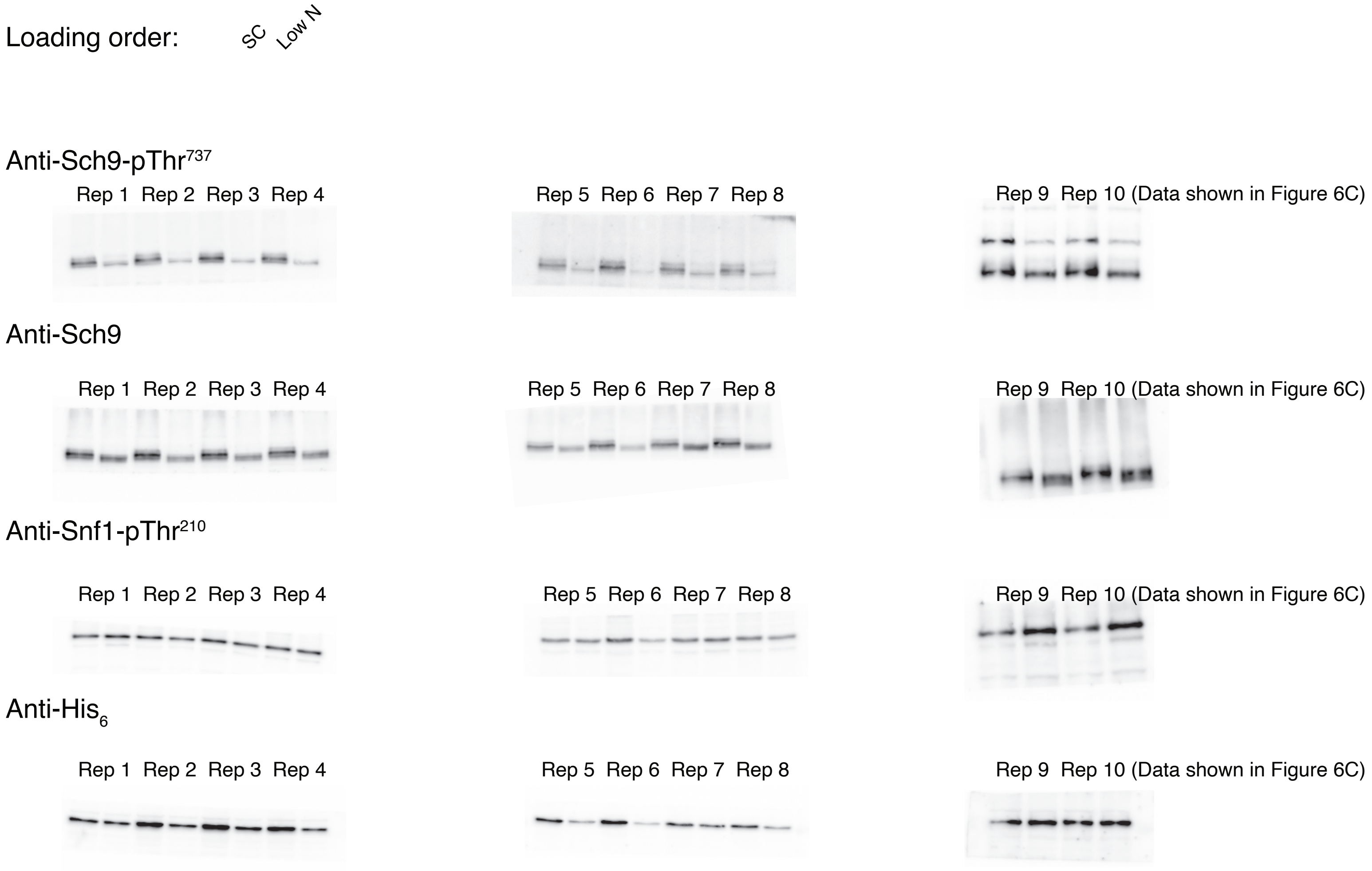

Replica 4 (Data shown in Figure 6A)

Figure 6D

Loading order:

	snf1^{as}						
Pib2 variant	WT	WT	WT	SASA	SESE	SASA	SESE
Sch9 variant	WT	SA	SE	WT	WT	SA	SE

Anti-Sch9-pThr^{737}

Replica 1 (Data shown in Figure 6D)

Replica 2

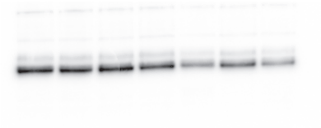

Replica 3

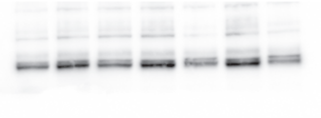

Replica 4

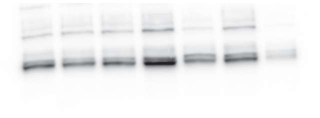

Anti-Sch9

Replica 1 (Data shown in Figure 6D)

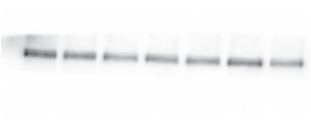

Replica 2

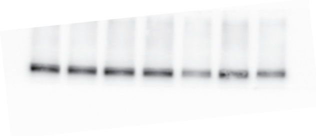

Replica 3

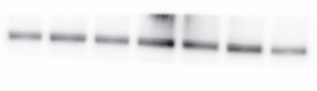

Replica 4

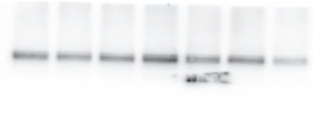

Anti-Snf1-pThr^{210}

Replica 1 (Data shown in Figure 6D)

Replica 2

Replica 3

Replica 4

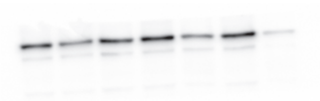

Anti-His_6

Replica 1 (Data shown in Figure 6D)

Replica 2

Replica 3

Replica 4

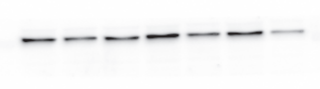

Anti-Adh1

Replica 1 (Data shown in Figure 6D)

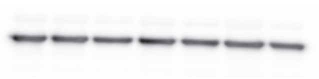

Replica 2

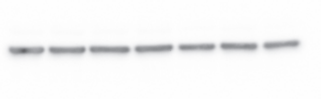

Replica 3

Replica 4

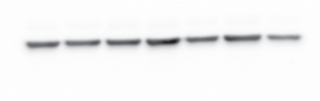

Figure 6E

Spotting order:

WT
$snf1^{as}$
pib2Δ
$sch9^{SA}$
$sch9^{SE}$
$pib2^{SASA}$
$pib2^{SESE}$
$sch9^{SA}$ $pib2^{SASA}$
$sch9^{SE}$ $pib2^{SESE}$

($snf1^{as}$ bracket spans *pib2Δ* through $sch9^{SE}$ $pib2^{SESE}$)

Control

Replica 1 (Data shown in Figure 6E) Replica 2

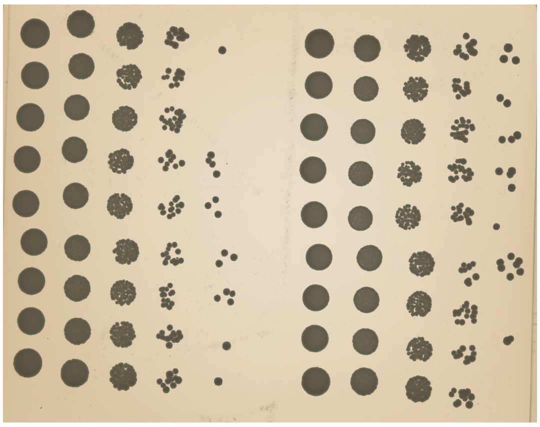

Replica 3 Replica 4

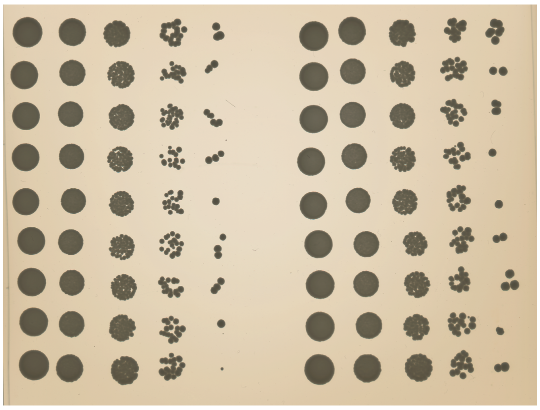

2.5 nM Rapamycin

Replica 1 (Data shown in Figure 6E) Replica 2

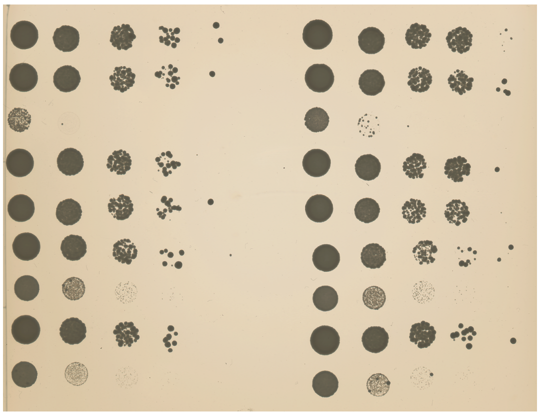

Replica 3 Replica 4

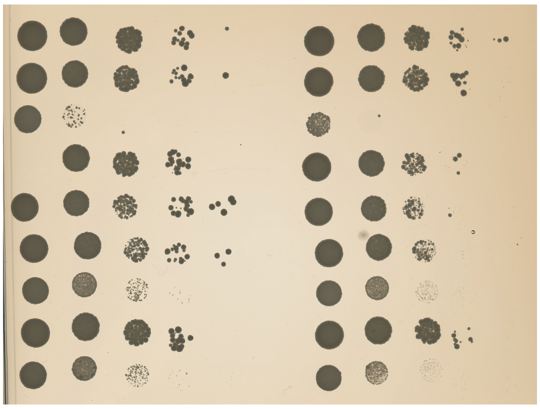

Supplement: Figure 6—source data 2. [file elife-84319-fig6-data2.pdf]
