# Supplementary material for: Snf1/AMPK fine-tunes TORC1 signaling in response to glucose starvation
Source: eLife. 2023 Feb 7;12:e84319. doi: 10.7554/eLife.84319 (PMC9937656; doi:10.7554/eLife.84319)
Supplement: Supplementary file 1. [file elife-84319-supp1.docx]

**Supplementary File 1A. Strains used in this study.**

| **Strain** | **Genotype** | **Source** | **Figure** |
| --- | --- | --- | --- |
| BY4741 | *MAT*a*;* *his3∆1, leu2∆0, met15∆0, ura3∆0* | Euroscarf |  |
| YL515 | [BY4741] *MATα; his3∆1, leu2∆0, ura3∆0* | ([Binda et al., 2009](#_ENREF_1)) | 1A; 1E; 1F; 6C; 6E; S1 |
| MC037 | [YL515] *MATα; snf11∆::HIS3MX6* | This study | 1A; 1F |
| MC012 | [YL515] *MATα; snf1^as^* | This study | 1A; 1C; 1F; 2A; 2C; 2E; 4F; 5D; 5F; 6A; 6D; 6E; S1; S2 |
| MC158 | [YL515] *MATα; reg1∆::kanMX* | This study | 1E; 1F |
| MB32 | [YL515] *MATα; gtr1∆::kanMX* | ([Binda *et al.*, 2009](#_ENREF_1)) | 1F |
| Snf1-TAP | [BY4741] *MAT*a*; SNF1-TAP:HIS3* | Open Biosystems([Powis et al., 2015](#_ENREF_3)) | 4D; 5C; S3 |
| MJ5682 | [YL515] *MATα;* *arg4∆::hisMX4 lys2∆::hphNT* | ([Hu et al., 2019](#_ENREF_2)) |  |
| NIC078 | [MJ5682] *MATα; snf1^as^* | This study | 3A |
| NIC103 | [BY4741] *MAT*a*; snf1^T210A^-TAP:HIS3* | This study | 4D; 5C; S3 |
| MC086 | [Snf1-TAP] *MAT*a*; SNF4-GFP:kanMX* | This study | 4E; 5E |
| MC013 | [MC012] *MATα; pib2∆::HIS3MX* | This study | 4F; 6E |
| MC058 | [MC012] *MATα; pib2^SASA^* | This study | 4F; 6A; 6D; 6E |
| MC059 | [MC012] *MATα; pib2^SESE^* | This study | 4F; 6A; 6D; 6E |
| MC145 | [MC012] *MATα; PIB2-myc_13_:kanMX* | This study | 4I |
| MC152 | [MC058] *MATα; pib2^SASA^-myc_13_:kanMX* | This study | 4I |
| MC153 | [MC059] *MATα; pib2^SESE^-myc_13_:kanMX* | This study | 4I |
| MC154 | [MC012] *MATα; KOG1-HA_3_:HIS3MX* | This study | 4H |
| MC155 | [MC145] *MATα; KOG1-HA_3_:HIS3MX* | This study | 4H |
| MC156 | [MC152] *MATα; KOG1-HA_3_:HIS3MX* | This study | 4H |
| MC157 | [MC153] *MATα; KOG1-HA_3_:HIS3MX* | This study | 4H |
| MC029 | [MC012] *MATα; sch9^S288A^* | This study | 5D; 5F; 6A; 6D; 6E |
| MC030 | [MC012] *MATα; sch9^S288E^* | This study | 5D; 5F; 6A; 6D; 6E |
| MC146 | [MC058] *MATα; sch9^S288A^* | This study | 6A; 6D; 6E |
| MC144 | [MC059] *MATα; sch9^S288E^* | This study | 6A; 6D; 6E |
| MC021 | [MC012] *MATα; lst4∆::HIS3MX* | This study | S2 |

**References**

Binda, M., Péli-Gulli, M.P., Bonfils, G., Panchaud, N., Urban, J., Sturgill, T.W., Loewith, R., and De Virgilio, C. (2009). The Vam6 GEF controls TORC1 by activating the EGO complex. Mol. Cell *35*, 563-573.

Hu, Z., Raucci, S., Jaquenoud, M., Hatakeyama, R., Stumpe, M., Rohr, R., Reggiori, F., De Virgilio, C., and Dengjel, J. (2019). Multilayered control of protein turnover by TORC1 and Atg1. Cell Rep. *28*, 3486-3496.

Powis, K., Zhang, T., Panchaud, N., Wang, R., De Virgilio, C., and Ding, J. (2015). Crystal structure of the Ego1-Ego2-Ego3 complex and its role in promoting Rag GTPase-dependent TORC1 signaling. Cell Res. *25*, 1043-1059.

**Supplementary File 1B. Plasmids used in this study.**

| **Plasmid** | **Genotype** | **Source** | **Figure** |
| --- | --- | --- | --- |
| pRS413 | *CEN, ARS, amp^R^, HIS3* | ([Brachmann et al., 1998](#_ENREF_1)) | 1A; 1C; 2A; 2C; 2E; 3A; 4F; 4H; 4I; 5D; 5F; 6A; 6C; 6D; 6E; S1; S2 |
| pRS415 | *CEN, ARS, amp^R^, LEU2* | ([Brachmann et al., 1998](#_ENREF_1)) | 1A; 1C; 2A; 2C; 2E; 3A; 4F; 4H; 4I; 5D; 5F; 6A; 6C; 6D; 6E; S1; S2 |
| pRS416 | *CEN, ARS, amp^R^, URA3* | ([Brachmann et al., 1998](#_ENREF_1)) | 1A; 1C; 2A; 2C; 2E; 3A; 4F; 4H; 4I; 5D; 5F; 6A; 6C; 6D; 6E; S1; S2 |
| pET-24d | *kan^R^, T7p, lacO* | Novagen |  |
| p3138 | [pET-24d] *His_6_-PIB2^221-635^* | This study | 4D; 4E |
| pMC030 | [pET-24d] *His_6_-pib2^221-635,S268A^* | This study | 4D |
| pMC031 | [pET-24d] *His_6_-pib2^221-635,S309A^* | This study | 4D |
| pMC032 | [pET-24d] *His_6_-pib2^221-635,S268,S309A^* | This study | 4D |
| YEplac195 | 2µ, *amp^R^, URA3* | ([Gietz and Sugino, 1988](#_ENREF_4)) |  |
| pMC013 | [YEplac195] *GAL1p-SCH9^1-394^-TAP* | This study | 5C; 5E; S3 |
| pMC016 | [YEplac195] *GAL1p-sch9^1-394,S288A^-TAP* | This study | 5C; S3 |
| pMC017 | [YEplac195] *GAL1p-sch9^K441A^-TAP* | This study | S3 |
| *pYX242-ACC1* | 2µ, *amp^R^, LEU2, TPI1p-ACC1-GFP-HA* | ([Deroover et al., 2016](#_ENREF_2)) | S1 |
| pRCC-K | 2µ, *amp^R^, kanMX, ROX3p-CAS9, SNR52p* | ([Generoso et al., 2016](#_ENREF_3)) |  |
| pNIC012 | [pRCC-K] *SNR52p-SNF1^I132^* (gRNA) | This study | 1A; 1C; 1F; 2A; 2C; 2E; 3A; 4F; 5D; 5F; 6A; 6D; 6E; S1; S2 |
| pNIC015 | [pRCC-K] *SNR52p-SNF1^T210^* (gRNA) | This study | 4D; 5C; S3 |
| pMC005 | [pRCC-K] *SNR52p-SCH9^S288^* (gRNA) | This study | 5D; 5F; 6A; 6D; 6E |
| pMC008 | [pRCC-K] *SNR52p-PIB2^S268^* (gRNA) | This study | 4F; 6A; 6D; 6E |
| pMC009 | [pRCC-K] *SNR52p-PIB2^S309^* (gRNA) | This study | 4F; 6A; 6D; 6E |

**References**

Brachmann, C.B., Davies, A., Cost, G.J., Caputo, E., Li, J., Hieter, P., and Boeke, J.D. (1998). Designer deletion strains derived from *Saccharomyces cerevisiae* S288C: a useful set of strains and plasmids for PCR-mediated gene disruption and other applications. Yeast *14*, 115-132.

Deroover, S., Ghillebert, R., Broeckx, T., Winderickx, J., and Rolland, F. (2016). Trehalose-6-phosphate synthesis controls yeast gluconeogenesis downstream and independent of SNF1. FEMS Yeast Res. *16*, fow036.

Generoso, W.C., Gottardi, M., Oreb, M., and Boles, E. (2016). Simplified CRISPR-Cas genome editing for *Saccharomyces cerevisiae*. J. Microbiol. Methods *127*, 203-205.

Gietz, R.D., and Sugino, A. (1988). New yeast-*Escherichia coli* shuttle vectors constructed with *in vitro* mutagenized yeast genes lacking six-base pair restriction sites. Gene *74*, 527-534.

**Supplementary File 1C. Oligonucleotides used in this study.**

| **Name** | **Orientation** | **Sequence** |
| --- | --- | --- |
| *snf1*^I132^ Proto-F | Forward | GAAATCATTATGGTTATAGAGTACGCCGTTTTAGAGCTAGAAATAGCAAGTTAAAATAAGG |
| *snf1*^I132^ Proto-R | Reverse | GGCGTACTCTATAACCATAATGATTTCGATCATTTATCTTTCACTGCGGAG |
| *snf1*^I132G^ Donor | Forward | TGATGTTATCAAATCCAAAGATGAAATCATTATGGTTGGAGAGTACGCCGGAAACGAATTGTTTGACTATATTGTTCAGA |
| *snf1*^T210^ Proto-F | Forward | GGTAATTTCTTAAAGACTTCTTGGTTTTAGAGCTAGAAATAGCAAGTTAAAATAAGG |
| *snf1*^T210^ Proto-R | Reverse | GAAGAAGTCTTTAAGAAATTACCGATCATTTATCTTTCACTGCGGAG |
| *sch9*^S288^ Proto-F | Forward | GAAGATGATCTGTGTGTATAAGTTTTAGAGCTAGAAATAGCAAGTTAAAATAAGG |
| *sch9*^S288^ Proto-R | Reverse | TTATACACACAGATCATCTTCGATCATTTATCTTTCACTGCGGAG |
| *sch9*^S288A^ Donor | Reverse | TACTGAAGAGCAAGAGTTTAGCTGATCTAATTGGGAAGATGCTCTGTGTGTATAAAGAGGTTTTTTCTTCAAGTGCTCTT |
| *sch9*^S288E^ Donor | Reverse | TACTGAAGAGCAAGAGTTTAGCTGATCTAATTGGGAAGATTCTCTGTGTGTATAAAGAGGTTTTTTCTTCAAGTGCTCTT |
| *pib2*^S268^ Proto-F | Forward | GAATTCTAGCTCGATGTCCCAACTGGTTTTAGAGCTAGAAATAGCAAGTTAAAATAAGG |
| *pib2*^S268^ Proto-R | Reverse | CAGTTGGGACATCGAGCTAGAATTCGATCATTTATCTTTCACTGCGGAG |
| *pib2*^S268A^ Donor | Forward | GAAAATATTGTCGACAAGCTGACTACAACGAATTCTAGCGCGATGTCCCAACTGCGATTTGGCAACACGAACGTCATTAT |
| *pib2*^S268E^ Donor | Forward | GAAAATATTGTCGACAAGCTGACTACAACGAATTCTAGCGAGATGTCCCAACTGCGATTTGGCAACACGAACGTCATTAT |
| *pib2*^S309^ Proto-F | Forward | GAATTAAAATTCAGATTAGTGCTCGAAGCGTTTTAGAGCTAGAAATAGCAAGTTAAAATAAGG |
| *pib2*^S309^ Proto-R | Reverse | GCTTCGAGCACTAATCTGAATTTTAATTCGATCATTTATCTTTCACTGCGGAG |
| *pib2*^S309A^ Donor | Reverse | GATTTATGTTTATTGGAATTAAAATTCAGATTAGTGCTCTCAGCAGGCTGCGGTAAAAATTCCAGCGAGGGTTTCCTTAG |
| *pib2*^S309E^ Donor | Reverse | GATTTATGTTTATTGGAATTAAAATTCAGATTAGTGCTCGAAGCAGGCTGCGGTAAAAATTCCAGCGAGGGTTTCCTTAG |
